# Construction of a high-density genetic map and mapping of a sex-linked locus for the brown alga *Undaria pinnatifida* (Phaeophyceae) based on large scale marker development by specific length amplified fragment (SLAF) sequencing

**DOI:** 10.1186/s12864-015-2184-y

**Published:** 2015-11-05

**Authors:** Tifeng Shan, Shaojun Pang, Jing Li, Xia Li, Li Su

**Affiliations:** Key Laboratory of Experimental Marine Biology, Institute of Oceanology, Chinese Academy of Sciences, Qingdao, 266071 PR China; Graduate University of Chinese Academy of Science, Beijing, 100049 PR China

**Keywords:** Genetic map, SNP, *Undaria pinnatifida*, Laminariales, Sex determination

## Abstract

**Background:**

*Undaria pinnatifida* is an important economic brown alga in East Asian countries. However, its genetic and genomic information is very scarce, which hinders further research in this species. A high-density genetic map is a basic tool for fundamental and applied research such as discovery of functional genes and mapping of quantitative trait loci (QTL). In this study the recently developed specific length amplified fragment sequencing (SLAF-seq) technology was employed to construct a high-density genetic linkage map and locate a sex determining locus for *U. pinnatifida.*

**Results:**

A total of 28.06 Gb data including 140.31 M pair-end reads was obtained. After linkage analysis 4626 SLAF markers were mapped onto the genetic map. After adding the sex linked simple sequence repeat (SSR) marker [GenBank:AY738602.1], the final genetic map was 1816.28 cM long, consisting of 30 linkage groups with an average distance of 0.39 cM between adjacent markers. The length of LGs ranged from 20.12 to 106.95 cM. A major sex associated QTL was mapped to LG22 within a window starting at 29.01 cM and ending at 68.81 cM with a total of 68 SLAF markers. The SSR marker and five SLAF markers (Marker6556, 19020, 43089, 60771 and 26359) were identified as tightly sex-linked markers, as indicated by the absence of recombination between them and the sex phenotype. These markers were located at the position of 59.50 cM, which was supposed to be the sex determining region.

**Conclusions:**

A high-density genetic linkage map was constructed using SLAF-seq technique and F1 gametophyte population for the first time in the economically important brown alga *U. pinnatifida*. For the first time, a major sex associated QTL suggesting a sex determining region was mapped to a single LG. This map will facilitate the further fundamental and applied research such as QTL mapping and map-based gene clone in *U. pinnatifida* and provide a reference for studies in other kelp species.

**Electronic supplementary material:**

The online version of this article (doi:10.1186/s12864-015-2184-y) contains supplementary material, which is available to authorized users.

## Background

*Undaria pinnatifida* (Harv.) Suringar is an important economic macroalga that has been used as sea vegetable for a long history in East Asian countries [1]. In China it has been farmed since 1980s, with the annual yield maintaining around 500,000 tons in wet weight in recent years, only second to *Saccharina japonica* in brown algae. It is also an invasive species that has spread to all continents except Antarctic and Africa [2–4] and many investigations have been conducted on its invasive mechanism [5–7]. Despite the economic and ecological importance and nutritional value, the genetic study of *U. pinnatifida* lags far behind that of *S. japonica*, for which the draft genome sequence has recently been sequenced and characterized [8]. Comparatively, the genetic exploration of *U. pinnatifida* is very limited, with only the transcriptome of gametophytes being sequenced till now [9]. *U. pinnatifida* has relatively complex and plastic morphology in comparison with other species of Laminariales (kelp) [10]; however, the underlying genomic basis remains unknown. A genetic linkage map is a basic and robust tool for genetic and genomic research, which can provide a foundation for identification of genomic loci linked to phenotypic variants, mapping of quantitative traits loci (QTL) and even anchoring genomic sequence scaffolds [11–13]. Unfortunately there has been no genetic linkage map for *U. pinnatifida*.

Genetic linkage maps have been extensively constructed and applied in land plants, animals and marine animals [14–16]. In contrast, much fewer genetic maps have ever been reported in macroalgae, so far only in *S. japonica*, *Ectocarpus siliculosus* and *Porphyra haitanensis* to our knowledge [13, 17–20]. As a model species of brown algae, the genome of *E. siliculosus* has been sequenced and a genetic map was constructed to support the assembly of the genome [13, 21]. QTL mapping and locating of sex-linked loci were conducted depending on the construction of genetic maps of *S. japonica* [18, 22]. In earlier time these genetic maps were constructed by conventional markers such as amplified fragment length polymorphism (AFLP), simple sequence repeat (SSR) and sequence-related amplified polymorphism (SRAP), thus limiting the density of the maps. A high-density genetic map of *S. japonica* has very recently been constructed based on single nucleotide polymorphisms (SNPs) [19], which are more robust markers than the above-mentioned ones because they are the most abundant form of genetic variation in the genome. However, SNPs used to be very expensive to develop; therefore they were only applied in model or very important species. With the advent of next generation sequencing technology, the sequencing cost has been dramatically reduced. High throughput sequencing technology provides novel strategies for SNPs development and genotyping. Sequencing of reduced representation library (RRL), which further reduces the sequencing cost by only sequencing representative parts of the complex genome, can rapidly detect thousands of SNP loci [23]. Accordingly it is very suitable for development of SNP markers, in particular in nonmodel species. The most common RRL sequencing methods include restriction site-associated DNA sequencing (RAD-seq) [24, 25], 2b-RAD [26], specific length amplified fragment sequencing (SLAF-seq) [27] and genotyping by sequencing (GBS) [28]. Among others, the advantage of SLAF-seq lies in that it can create a balance between higher genotyping accuracy and relatively lower sequencing cost [29, 30]. It has been effectively applied in SNPs development, high-density genetic mapping and QTL mapping in organisms with or without reference genomic sequence [29–33]. It is thus expected that this method can also be suitable for SNPs development and construction of high-density genetic map in nonmodel macroalgal species such as *U. pinnatifida*.

As a kelp species, *U. pinnatifida* has a typical life cycle that alternates between microscopic haploid gametophyte and macroscopic diploid sporophyte. Male and female sexes are expressed after meiosis, at the haploid stage of the life cycle. This type of sexual system, which has recently been termed as UV, is in common with bryophytes and some other algae [34]. The chromosomes of kelp species are very small in size and droplet-shaped, making it difficult to conduct research on the karyotype [35, 36]. As a result, there has been a long time debate regarding the exact chromosome numbers of kelp species and whether they possess a sex chromosome. Recently, the chromosome number of the haploid gametophyte of *S. japonica* has been determined to be 31 with an improved chromosome preparation and staining method [37]. Moreover, a female-related FRML-494 marker (494-bp female-related marker of *S. japonica*) was localized on a unique chromosome of the female gametophyte and sporophyte [38]. In spite of this progress, the sex determining mechanism of kelp species still remains largely unknown. Genetic mapping can be used to locate the sex-linked locus, thus being very helpful to decipher the sex determining mechanism, as shown by the result that high-density genetic mapping suggested a ZW sex determining system in Chinese mitten crab *Eriocheir sinensis* [39]. Similarly, it has recently been revealed that sex of *Ectocarpus* sp. is determined at the haploid stage by a nonrecombining region on linkage group (LG) 30 [40]. This sex determining region (SDR) was then sequenced and characterized, which suggested a distinct evolutionary history in brown algae as compared to the XY and ZW sexual system. The sex determining locus was also mapped to a unique LG (LG 2) on the high-density genetic map of *S. japonica* [19]. These results imply that kelp species might have a similar sex determining mechanism to that of *Ectocarpus* sp. The haploid chromosome number of *U. pinnatifida* was preliminarily determined to be 30 by traditional staining method [41] and its genome size was estimated to be 560 Mbp [42]. A sex-linked SSR marker (UP-AC-2A8) [GenBank:AY738602.1] was identified only present in female gametophytes [43]. However, there is no further analysis on either the inheritance pattern or the chromosome location of this marker. Furthermore, unusual monoecious zoospore-derived gametophytes were found in *U. pinnatifida* [44]; however, the genetic basis of this phenomenon is unknown. Construction of a high-density genetic linkage map is expected to be an essential step towards the understanding of sex determining mechanism in *U. pinnatifida*.

In this study, the SLAF-seq technique was employed to construct a high-density genetic linkage map for *U. pinnatifida*, aiming at providing a basic genetic tool for further fundamental and applied research as well as mapping the sex determining locus in this species.

## Results

### Analysis of SLAF-seq data and SLAF markers

After SLAF library construction and high throughput sequencing, a total of 28.06 Gb data including 140.31 M pair-end reads was obtained. Among them, the total read number for maternal and paternal parent was10.92 M and 10.31 M, respectively; while the average number of reads per progeny was 1.18 M. The Q30 (means a sequencing quality score of 30, indicating a 0.1 % chance of an error, and thus 99.9 % confidence) percentage was 89.79 % and guanine-cytosine (GC) content was 51.45 % on average. The raw data of SLAF-seq have been submitted to NCBI SRA database under a bioproject accession number SRP060486.

**Fig. 1:**
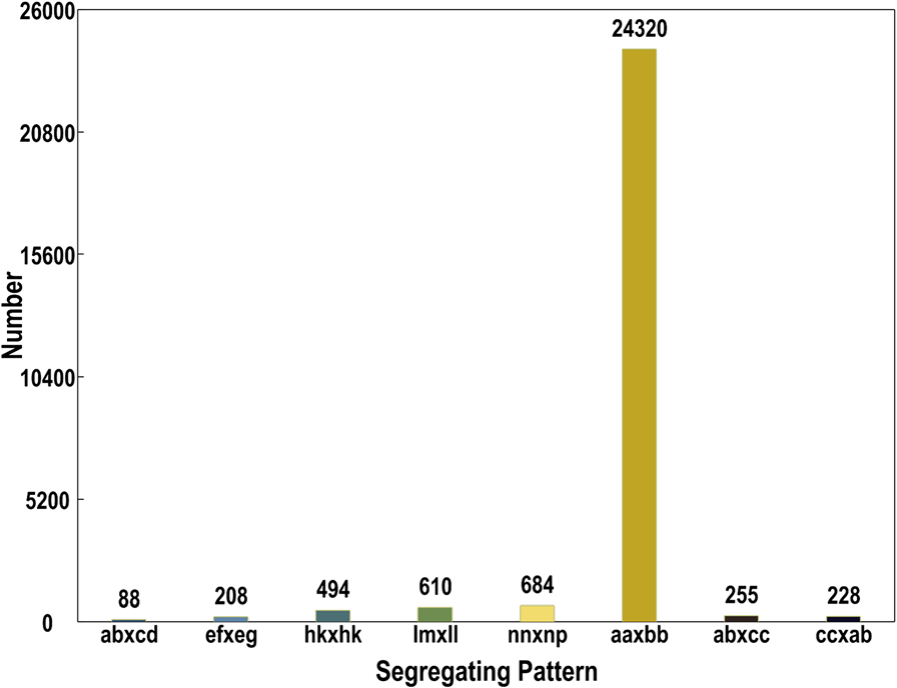
The number of markers in each of eight segregation patterns

The SLAFs number of maternal and paternal parent was 134,975 and 139,873, with an average coverage of 50.88 and 54.39 fold, respectively. The SLAFs number in the offspring was 104,831, with an average coverage of 8.16 fold (Table 1). In total, 202,572 high-quality SLAFs were developed and 33,093 of them were polymorphic, with a polymorphism percentage of 16.34 % (Table 2). Among the polymorphic SLAFs, 26,887 were classified into eight segregation patterns. Because the mapping population was the F1 haploid population, only SLAFs with the aa × bb segregation pattern was used for genetic map construction. A total of 24,320 SLAFs fell into this class (Fig. 1). After filtering out the SLAFs with the sequencing coverage less than 10 fold in parents, or integrity less than 70 %, or that showing significant segregation distortion (*P* < 0.05), 4821 SLAFs were obtained and used for construction of the linkage map.

**Table 1:** Summary of the developed specific length amplified fragment (SLAF) markers

Samples	SLAF number	Total depth	Average depth
Maternal parent	134,975	6,868,171	50.88×
Paternal parent	139,873	7,607,425	54.39×
Offspring	104,831	855,106	8.16×

**Table 2:** Polymorphism analysis results of the SLAF markers

SLAF Type	Polymorphic	Non-polymorphic	Repetitive	Total
Number	33,093	169,220	259	202,572
Percentage (%)	16.34	83.54	0.13	100

### High-density genetic linkage map construction and mapping of a sex-linked locus

**Fig. 2:**
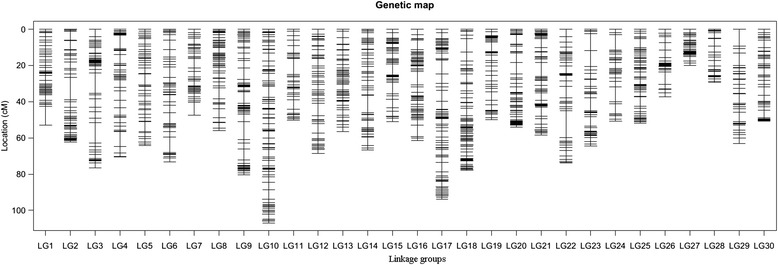
Distribution of SLAF markers on 30 linkage groups of *Undaria pinnatifida*. A black bar indicates a SLAF marker. The x-axis indicates linkage group number and the y-axis the map distance (cM)

A total of 4626 SLAFs were mapped onto the genetic map after linkage analysis. The SLAF sequences and the genotype of all mapping samples were given in Additional file 1. The mean coverage of these markers was 63.96 fold in the maternal parent, 92.53 fold in the paternal parent and 13.93 fold in the offspring on average. After adding the SSR marker (UP-AC-2A8), the final genetic map was 1816.28 cM in length, consisting of 30 LGs with an average distance of 0.39 cM between adjacent markers (Table 3). The largest LG was LG10, with a length of 106.95 cM and an average maker interval of 0.48 cM, while the smallest LG was LG27, with a length of 20.12 cM and an average maker interval of 0.22 cM (Fig. 2). The largest gap was located on LG22, with a marker interval of 13.17 cM (Table 3 and Additional file 2). The ratio of double crossover was less than 3 % as evaluated by the haplotype map, suggesting a good quality of maker linear order on LGs. No evident recombination hotspot was found in the haplotype map (Additional file 3). Neither did heat maps demonstrate apparent regions of frequent recombination, indicating the LGs performed well in general (Additional file 4).

**Table 3:** The characteristics of the 30 linkage groups constructed in *Undaria pinnatifida*

Linkage group	No. of markers	Total distance(cM)	Average distance(cM)	Maximum gap
1	244	53.02	0.22	10.55
2	135	62.24	0.46	12.28
3	183	76.61	0.42	11.03
4	159	70.43	0.44	8.18
5	126	64.27	0.51	5.97
6	160	73.15	0.46	8.61
7	110	47.42	0.43	6.97
8	231	55.96	0.24	6.17
9	172	80.33	0.47	9.81
10	221	106.95	0.48	6.22
11	98	50.26	0.51	8.62
12	128	68.67	0.54	6.94
13	126	56.63	0.45	3.75
14	126	66.57	0.53	9.72
15	193	51.05	0.26	6.32
16	136	61.57	0.45	7.46
17	274	93.97	0.34	7.44
18	292	77.93	0.27	7.43
19	145	49.90	0.34	5.15
20	181	53.97	0.30	6.26
21	164	58.40	0.36	7.43
22	100	68.81	0.69	13.17
23	121	64.51	0.53	9.31
24	105	50.76	0.48	8.61
25	206	51.83	0.25	5.21
26	96	37.41	0.39	6.88
27	93	20.12	0.22	2.48
28	93	29.46	0.32	4.12
29	107	63.22	0.59	9.33
30	102	50.86	0.50	4.73
Total	4,627	1,816.28	0.39	13.17

**Fig. 3:**
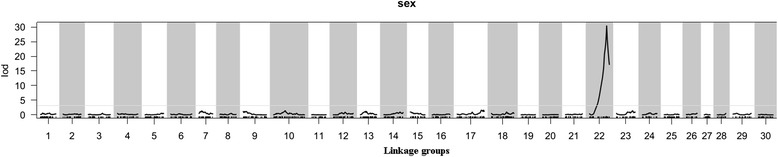
QTL analysis of the sex phenotype on linkage groups. The x-axis indicates the linkage groups and the marker order. The horizontal gray line indicates the threshold of the LOD score (4.1) for significance (*P* = 0.01) at the whole genomic level

**Fig. 4:**
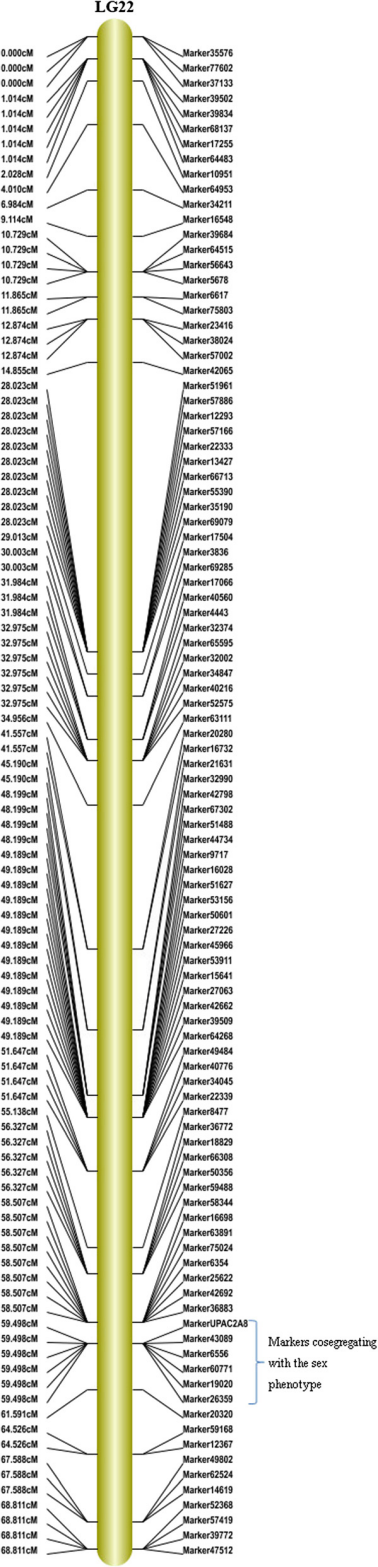
Linkage group 22 and the location of cosegregating markers with the sex phenotype

In the QTL linkage analysis of sex phenotype, the threshold of LOD was determined to be 4.1 (*P* = 0.01). A major sex associated locus was mapped to LG22 within a window starting at 29.01 cM and ending at 68.81 cM with a total of 68 SLAF markers (Figs. 3, 4 and Additional file 5). The SSR marker (UP-AC-2A8) and five SLAF markers (Marker 6556, 19020, 43089, 60771 and 26359) were identified as tightly sex-linked markers, as indicated by the zero recombination rate between them and sex phenotype. These markers were located at the position of 59.50 cM (Fig. 4). In blast search against unigene database of *U. pinnatifida*, Marker 60771 showed significant similarity to the unigene CL11961Contig1, which was annotated as an unnamed protein product of *Chondrus crispus* (Additional files 6 and 7). Marker 43089 had significant similarities to CL134Contig1 and CL1827Contig1, with the former being annotated as a hypothetical protein of *E.siliculosus* and the latter unannotated.

## Discussion

DNA markers and mapping population are indispensable elements for construction of the genetic linkage map [11, 45]. SSRs have been the traditional markers of choice for genetic map construction due to their codominant nature, high polymorphism and good transferability [46]. However, there are only 20 genomic microsatellite markers available for *U. pinnatifida* [47]. Although a large number of tentative EST-SSRs have been developed recently [9], their polymorphism and reliability have not been tested. With the reduction of sequencing cost brought by advanced generation sequencing approach, SNPs are becoming the first choice for construction of high-density genetic linkage map, in which the average marker interval can reach < 1 cM, thus providing more accurate foundation for discovery of functional genes and analysis of genome structure [48]. In this study, a large number of SLAF markers were developed and a high-density genetic map was constructed in *U. pinnatifida*. The quality and quantity of the developed markers were enough to meet the requirements for construction of a high density genetic map. In total, 4626 SLAF and one SSR markers were assigned to 30 LGs, which equals the chromosome number identified by Yabu et al [41]. The average distance between adjacent markers was 0.39 cM, very similar to the marker density (0.36 cM) in the genetic map of *S. japonica* [19]. Evaluation with haplotype map and heat map indicated a high quality of the genetic map. To our knowledge, this is the first genetic linkage map of *U. pinnatifida* to date. With the high-density map and draft genomic sequence of *S. japonica* being available [8], it is possible to conduct comparative analysis between *U. pinnatifida* and *S. japonica* in the next step [49].

In this research, F1 haploid population was used to construct the genetic linkage map. Similar to bryophyte, the haploid gametophyte of kelp species can propagate vegetatively by mitosis, making it feasible to establish F1 haploid population, which is the progeny of one segregating meiosis. As the gametophytes can be preserved for a long time without genetic variation, F1 haploid population provides a perpetual resource that can be used for genetic mapping and the further research. Genetic markers can be continually added to the map in future studies and the mapping population or the corresponding DNA can be shared with different labs for additional analysis. In this sense, F1 haploid population is genetically identical to double haploid (DH) population, except for the difference in ploidy. Moreover, since the sex trait of kelp species is expressed in haploid gametophyte stage, only gametophyte population can be used to map sex linked loci. F1 haploid populations have been used for construction of genetic map and mapping of sex determining locus in the moss *Ceratodon purpureus* and the brown algae *S. japonica* and *Ectocarpus* sp. [19, 40, 50]. Because of the single meiosis event it has gone through, F1 haploid population suffered from relatively low recombination rate that might be the reason for the short map distance of some LGs in this study such as LG27, in which the recombination rate between most adjacent markers was less than 1 %. This problem is expected to be resolved to a certain extent by constructing a larger F1 haploid population using parents with more heterozygosity.

We also associated the mapped SLAF markers to sex trait. A major QTL involved in sex determination was detected in LG 22, with a location spanning a region of around 40 cM. This result preliminarily implied that LG 22 is the potential sex chromosome for *U. pinnatifida*. In *S. japonica*, a sex determining locus was mapped to LG 2 in a window about 9.0 cM in width [19]. In *Eriocheir sinensis*, all the sex linked markers in the mapping family were located on a single linkage group, LG 60 within a size of 15.15 cM by using QTL mapping of the sex phenotype [39]. In *Hippoglossus hippoglossus*, a major sex associated QTL was also found spanning a region around 22 cM [51]. Genomic regions with reduced recombination with sex trait are a common characteristic of sex chromosomes. These regions are supposed to contain genes for sex determination. Fortunately, five SLAF and one SSR markers were found to be completely linked to sex trait in this study, therefore these markers will be of special importance in characterizing the structure of the sex determining region (SDR). In previous studies, the SSR marker (UP-AC-2A8) has been identified to be tightly linked to quite a number of female gametophytes that were originated from geographically isolated populations [43, 44, 52]. This study further confirmed the sex linked characteristics of this marker in a segregating family. In the high-density genetic map of *Carica papaya*, a total of 225 AFLP markers were found to be cosegregating with sex determination locus [53]. Similarly, 30 markers cosegregated with sex in the genetic map of *Ceratodon purpureus* [50]. These results showed that recombination was severely suppressed in the region surrounding the sex determining locus. The large number of cosegregating markers in *Carica papaya* and *Ceratodon purpureus* suggested extensive sequence divergence at the sex determining locus. In comparison, only six cosegregating markers were identified in *U. pinnatifida*. The male and female SDR scaffolds of *Ectocarpus* sp. have been identified to be approximately 920 kilobase pairs, in which four completely sex linked SSR markers were found [40]. These results suggest that the SDR of *U. pinnatifida* might be similar to that of *Ectocarpus* sp., being of small size but highly diverged. Mapping of the sex determining locus was just the first step towards deciphering the sex determining mechanism of *U. pinnatifida*. The physical structure of the SDR needs to be analyzed and characterized in the future. Besides the research on genomic level, transcriptome analysis is also necessary to compare the differential gene expression between the two sexes to fully understand the genetic basis of sexual dimorphism. Such kind of studies has recently been conducted in *Fucus* and *Ectocarpus* [54, 55]. Fine mapping in combination with transcriptome analysis will provide new insight into the sex determining mechanism as demonstrated in recent studies [39].

The high-density genetic map built in this study is supposed to provide a robust tool for further fundamental and applied research in *U. pinnatifida*. However, the mapping population consisted of microscopic gametophytes, thus they cannot be used directly for QTL mapping of important economic traits such as length, width and weight. Instead, mapping populations composed of sporophytes must be established for QTL research, which will be another focus in our future study.

## Conclusions

A high-density genetic map was constructed for the first time in the economic brown alga *Undaria pinnatifida* by using SLAF-seq technology. A total of 4626 SLAFs and one SSR marker (UP-AC-2A8) were mapped onto the genetic map, which was 1816.28 cM long, consisting of 30 LGs with an average distance of 0.39 cM between adjacent markers. The length of the LGs ranged from 20.12 to 106.95 cM. A sex associated locus was mapped to LG22 within a window starting at 29.01 cM and ending at 68.81 cM with a total of 68 SLAF markers. The SSR marker and five SLAF markers (Marker 6556, 19020, 43089, 60771 and 26359) were identified as sex linked markers, as indicated by the zero recombination rate between sex phenotype and them. These markers are located at the position of 59.50 cM, which was supposed to be the sex determining region. This map will serve as a basic genetic tool for further fundamental and applied research of *U. pinnatifida* and may provide a reference for studies in other kelp species.

## Methods

### Establishment of the mapping population and DNA isolation

Recombinant first filial (F1) haploid gametophytes were used as mapping population in this study. The paternal and maternal unialgal gametophyte clones were respectively 10#F1-2-5 M and 5# F1-2-5 F, which were originated from the cultivated populations in Dalian in 2009. Sporophytes were obtained by crossing the parental gametophytes and cultured in indoor tanks with tumbling natural seawater. After maturity, zoospores were released from one sporophyte and unialgal gametophyte clones were isolated as described previously [56]. Sixty gametophyte clones of each sex were randomly selected after the sex could be discriminated under microscope. They were cultured in flasks with 50 mL of sterilized Provasoli enriched seawater (PES) [57] in irradiance of 4 μmol photons m^-2^s^-1^, 20 °C, where they were kept in a vegetative phase. Ultimately, 50 female and 51 male gametophytes survived and grew to enough quantity for DNA isolation. The sex of these gametophytes was further confirmed by gametogenesis experiment, in which the sex was able to be determined by the formation of oogonia or antheridia. These gametophytes constituted the mapping population for construction of high-density genetic linkage map. Genomic DNA was extracted by using DNeasy Plant Mini Kit (Qiagen). DNA quality and quantity were assessed by electrophoresis in 0.8 % agarose gel and a Nanodrop 2000 spectrophotometer (Thermo).

### SLAF library construction and high throughput sequencing

An improved SLAF-seq method was used to genotype the parents and 101 progenies as previous described, with minor modifications [27, 32]. Briefly, a SLAF pre-design in silico simulation experiment was conducted with the genome of *E. siliculosus* as the reference to establish an optimum enzyme digestion scheme. Then the SLAF library was constructed according to the pre-designed scheme. Genomic DNA was completely digested with *Hae*III (New England Biolabs, NEB). An adenine nucleotide (A) overhang was added to the digested fragments using the Klenow Fragment (3´ → 5´ exo–) (NEB) and dATP at 37 °C, and then the duplex tag-labeled sequencing adapters (PAGE purified, Life Technologies) were ligated to the A-tailed DNA fragments with T4 DNA ligase [58]. Polymerase chain reaction (PCR) was performed using diluted restriction-ligation samples, dNTP, Q5® High-Fidelity DNA Polymerase and PCR primers: AATGATACGGCGACCACCGA and CAAGCAGAAGACGGCATACG (PAGE purified, Life Technologies). The PCR products were purified by using Agencourt AMPure XP beads (Beckman Coulter, High Wycombe, UK) and pooled. The pooled sample was separated by electrophoresis in a 2 % agarose gel. Fragments of 400 to 450 bp (including indexes and adaptors) in size were excised, purified using QIAquick Gel Extraction Kit (Qiagen, Hilden, Germany). The diluted gel-purified products were submitted to pair-end sequencing (each end 100 bp) on the Illumina HiSeq 2500 system (Illumina, Inc; San Diego, CA, U.S.) according to the manufacturer’s instructions. The indices sequences and the codes for each sample were shown in Additional file 8.

### SLAF-seq data grouping and genotyping

Grouping and genotyping of SLAF markers were performed according to the procedures previously described [27, 32]. In brief, low-quality reads with a quality score < Q20 (means a sequencing quality score of 20, indicating a 1 % chance of an error, and thus 99 % confidence) were discarded and all SLAF pair-end reads with clear index information were clustered depending on sequence similarity by using BLAT (−tileSize = 10 -stepSize = 5). Sequences with over 95 % identity were grouped in one SLAF locus. SNP loci of each SLAF locus were then detected between parents, and SLAFs with more than 3 SNPs were filtered out firstly. Alleles were defined in each SLAF by the minor allele frequency (MAF) evaluation. Since a haploid population was used in this study, only the SLAF markers whose segregation patterns were aa × bb were used for genetic map construction. Moreover, because at most two SLAF tags existed in one locus, groups containing more than two tags were filtered out as repetitive SLAFs. SLAFs with 2 tags were identified as potential polymorphic SLAF markers.

### Genotyping of the sex-linked SSR marker

PCR amplification and genotyping of the sex-linked SSR marker UP-AC-2A8 was conducted according to the previously described procedure [43, 47] in the mapping population.

### Linkage map construction and mapping of the sex-determining locus

The SLAF markers were partitioned into LGs based on a pairwise modified logarithm of odds (MLOD) scores. Markers with MLOD scores < 5 were abandoned prior to ordering. HighMap software was employed to order the SLAF markers and correct genotyping errors within LGs [59]. Briefly, recombination frequencies and LOD scores were calculated by two-point analysis. Then, a combination of enhanced Gibbs sampling, spatial sampling and simulated annealing algorithms was used to perform an iterative process of marker ordering. The error correction strategy of SMOOTH was conducted according to parental contribution of genotypes, and the k-nearest neighbor algorithm was applied to impute missing genotypes. Markers with significant segregation distortion (*P* < 0.05) were excluded from map construction. Kosambi mapping function was utilized to estimate the map distances. Haplotype map and heat map were used to evaluate the quality of the genetic map [60]. “Draw_haplotype-map.pl” and “draw_heatmap.pl” were used to construct haplotype and heat map, respectively. Haplotype map was used to look for the possible double crossover, which suggested the genotyping and marker-order errors. Haplotype maps were generated for each of the F1 haploid individuals and the parental controls using the 4626 SLAF markers. Heat maps not only evaluate the quality of the genetic map using the pair-wise recombination values for all the mapped SLAF markers, but also reflect the relationship of recombination between markers from one single LG. Both of them were programmed by Beijing Biomarker Technologies Corporation, and can be downloaded at http://highmap.biomarker.com.cn/. The sex-linked microsatellite marker was integrated on the genetic map using the same method.

Finding of sex associated loci was conducted with R/QTL software [61] using interval mapping method based on the high-density linkage map. The sex phenotype was regarded as a binary trait (1 for females and 0 for males). The significance of each QTL interval was tested by a likelihood-ratio statistic (LOD). The threshold of the LOD score for significance (*P* = 0.01) at the whole genomic level was determined using 1000 permutation test for the trait. Similarity of sex-linked SLAF markers to the unigenes of *U. pinnatifida* was searched by blast analysis [9, 62].

## Additional files

Additional file 1:
**Sequences of the mapped SLAF markers and genotype of the mapping samples.** (PDF 9946 kb) Additional file 2:
**High-density genetic linkage map of** ***Undaria pinnatifida*** **constructed in this study.** (XLSX 1104 kb) Additional file 3:
**Haplotype map of the genetic map. Blue and red represent markers originating from maternal and paternal parent, respectively.** Gray represents missing data. Rows indicate the markers on the linkage group and columns the genotype of an individual. (PDF 27195 kb) Additional file 4:
**Heat map of the genetic map: Each cell represents the recombination rate between two markers.** The color change from yellow through red to purple indicates the change of recombination rate from low to high. (PDF 23911 kb) Additional file 5:
**The SLAF markers encompassed in the major sex associated locus as revealed by QTL analysis.** (XLSX 13 kb) Additional file 6:
**Result of the blast analysis of the tightly** **sex-linked** **markers against the unigene database of** ***Undaria pinntifida*** **.** (DOCX 18 kb) Additional file 7:
**The sequence and annotation of unigenes showing significant similarities to sex-linked SLAF markers of** ***Undaria pinnatifida*** **.** (XLSX 9 kb) Additional file 8:
**The indices sequences and the codes for each sample of the mapping population.** (XLSX 13 kb)

## Abbreviations

SLAF: Specific length amplified fragment
QTL: Quantitative trait loci
SSR: Simple sequence repeat
AFLP: Amplified fragment length polymorphism
SRAP: Sequence-related amplified polymorphism
SNP: Single nucleotide polymorphism
RRL: Reduced representation library
RAD: Restriction site-associated DNA
GBS: Genotyping by sequencing
LG: Linkage group
PES: Provasoli enriched seawater
PCR: Polymerase chain reaction
LOD: Logarithm of odds
SDR: Sex determining region
