# Supplementary material for: Construction of a high-density genetic map and mapping of a sex-linked locus for the brown alga Undaria pinnatifida (Phaeophyceae) based on large scale marker development by specific length amplified fragment (SLAF) sequencing
Source: BMC Genomics. 2015 Nov 5;16:902. doi: 10.1186/s12864-015-2184-y (PMC4635539; doi:10.1186/s12864-015-2184-y)
Supplement: Additional file 1: — Sequences of the mapped SLAF markers and genotype of the mapping samples. (PDF 9946 kb) [file 12864_2015_2184_MOESM1_ESM.pdf]

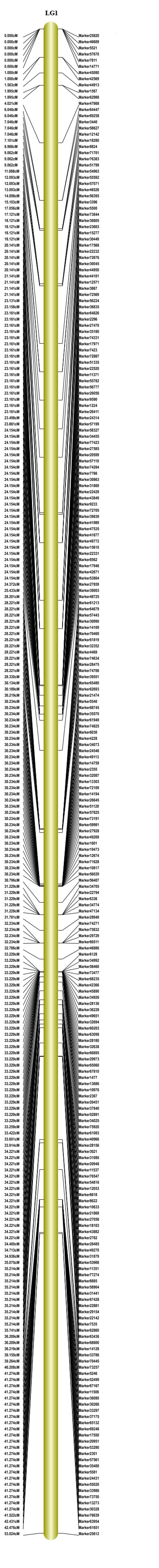
LG1

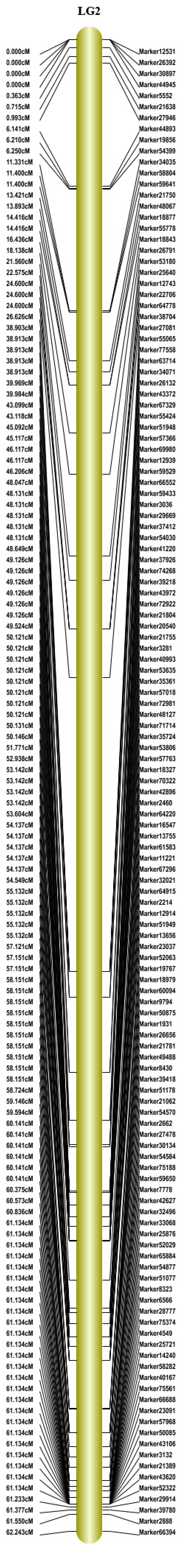
LG2
0.000cM Marker12531
0.000cM Marker26392
0.000cM Marker30897
0.000cM Marker44945
0.363cM Marker5552
0.715cM Marker21638
0.993cM Marker27946
6.141cM Marker44893
6.210cM Marker19856
6.250cM Marker54399
11.331cM Marker34035
11.400cM Marker58804
11.400cM Marker59641
13.421cM Marker21750
13.893cM Marker48067
14.416cM Marker18877
14.416cM Marker55778
16.436cM Marker18843
18.138cM Marker26791
21.560cM Marker53180
22.575cM Marker25640
24.600cM Marker12743
24.600cM Marker22706
24.600cM Marker64778
26.626cM Marker38704
38.903cM Marker27081
38.913cM Marker55065
38.913cM Marker77558
38.913cM Marker63714
38.913cM Marker34071
39.969cM Marker26132
39.984cM Marker43372
43.099cM Marker67329
43.118cM Marker55424
45.092cM Marker51948
45.117cM Marker57366
46.117cM Marker69980
46.117cM Marker12939
46.206cM Marker59529
48.047cM Marker66552
48.131cM Marker59433
48.131cM Marker3036
48.131cM Marker29669
48.131cM Marker37412
48.131cM Marker54030
48.649cM Marker41220
49.126cM Marker37926
49.126cM Marker74268
49.126cM Marker39218
49.126cM Marker43972
49.126cM Marker72922
49.126cM Marker21804
49.524cM Marker20540
50.121cM Marker21755
50.121cM Marker3281
50.121cM Marker40993
50.121cM Marker53635
50.121cM Marker35361
50.121cM Marker57018
50.121cM Marker72981
50.121cM Marker48127
50.131cM Marker71714
50.146cM Marker35724
51.771cM Marker53806
52.938cM Marker57763
53.142cM Marker18327
53.142cM Marker70322
53.142cM Marker42896
53.142cM Marker2460
53.604cM Marker64220
54.137cM Marker16547
54.137cM Marker13755
54.137cM Marker61583
54.137cM Marker11221
54.137cM Marker67296
54.549cM Marker32021
55.132cM Marker64915
55.132cM Marker2214
55.132cM Marker12914
55.132cM Marker51949
55.132cM Marker13656
57.121cM Marker23037
57.151cM Marker52063
57.151cM Marker19767
58.151cM Marker18979
58.151cM Marker60094
58.151cM Marker9794
58.151cM Marker50875
58.151cM Marker1931
58.151cM Marker26656
58.151cM Marker21781
58.151cM Marker49488
58.151cM Marker8430
58.151cM Marker39418
58.724cM Marker51178
59.146cM Marker21062
59.594cM Marker54570
60.141cM Marker2662
60.141cM Marker27478
60.141cM Marker30134
60.141cM Marker54584
60.141cM Marker75188
60.141cM Marker59650
60.375cM Marker7778
60.573cM Marker42627
60.836cM Marker32496
61.134cM Marker33068
61.134cM Marker25876
61.134cM Marker52029
61.134cM Marker65884
61.134cM Marker54877
61.134cM Marker51077
61.134cM Marker8323
61.134cM Marker6566
61.134cM Marker28777
61.134cM Marker75374
61.134cM Marker4549
61.134cM Marker25721
61.134cM Marker14240
61.134cM Marker58282
61.134cM Marker40167
61.134cM Marker75561
61.134cM Marker66688
61.134cM Marker23091
61.134cM Marker57968
61.134cM Marker50085
61.134cM Marker43106
61.134cM Marker3132
61.134cM Marker21389
61.134cM Marker43620
61.134cM Marker52322
61.233cM Marker29914
61.377cM Marker39780
61.550cM Marker2888
62.243cM Marker66394

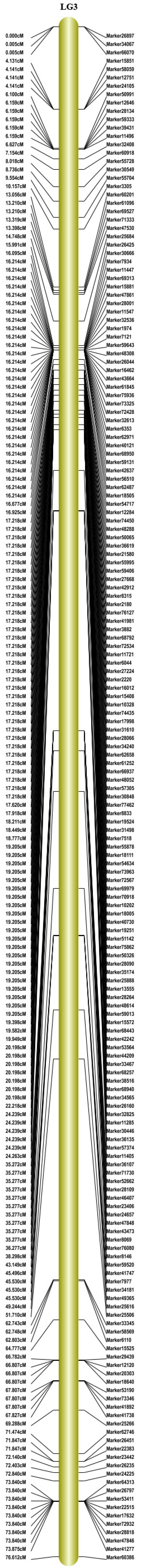
LG3
0.000cM Marker26897
0.005cM Marker34067
0.005cM Marker66070
4.131cM Marker15851
4.141cM Marker58059
4.141cM Marker12751
4.141cM Marker24105
6.100cM Marker50991
6.159cM Marker12646
6.159cM Marker28134
6.159cM Marker59333
6.159cM Marker39431
6.159cM Marker11496
6.627cM Marker32408
7.154cM Marker60918
8.018cM Marker55728
8.736cM Marker30549
9.554cM Marker55704
10.157cM Marker3305
13.056cM Marker60201
13.210cM Marker61096
13.210cM Marker69527
13.319cM Marker71333
13.398cM Marker47530
14.748cM Marker25684
15.991cM Marker26425
16.095cM Marker30666
16.214cM Marker7934
16.214cM Marker11447
16.214cM Marker69313
16.214cM Marker15881
16.214cM Marker47861
16.214cM Marker28001
16.214cM Marker11547
16.214cM Marker32536
16.214cM Marker1974
16.214cM Marker7121
16.214cM Marker59643
16.214cM Marker48308
16.214cM Marker26044
16.214cM Marker16462
16.214cM Marker43664
16.214cM Marker61845
16.214cM Marker75936
16.214cM Marker73325
16.214cM Marker72428
16.214cM Marker32613
16.214cM Marker6353
16.214cM Marker62971
16.214cM Marker40121
16.214cM Marker68950
16.214cM Marker59131
16.214cM Marker42637
16.214cM Marker56510
16.214cM Marker62487
16.214cM Marker18505
16.677cM Marker54717
16.925cM Marker12284
17.218cM Marker74450
17.218cM Marker48288
17.218cM Marker50065
17.218cM Marker36619
17.218cM Marker21580
17.218cM Marker55995
17.218cM Marker59406
17.218cM Marker27668
17.218cM Marker42912
17.218cM Marker8315
17.218cM Marker2180
17.218cM Marker76127
17.218cM Marker41981
17.218cM Marker3882
17.218cM Marker68792
17.218cM Marker72534
17.218cM Marker11721
17.218cM Marker6044
17.218cM Marker27224
17.218cM Marker2220
17.218cM Marker16012
17.218cM Marker15408
17.218cM Marker10328
17.218cM Marker74435
17.218cM Marker17998
17.218cM Marker31610
17.218cM Marker28066
17.218cM Marker34240
17.218cM Marker62658
17.218cM Marker61252
17.218cM Marker66937
17.218cM Marker48052
17.218cM Marker57305
17.218cM Marker30848
17.620cM Marker77462
17.918cM Marker8833
18.211cM Marker19524
18.449cM Marker31498
18.777cM Marker7518
19.205cM Marker55878
19.205cM Marker18111
19.205cM Marker54634
19.205cM Marker73963
19.205cM Marker72567
19.205cM Marker69979
19.205cM Marker70918
19.205cM Marker10202
19.205cM Marker18005
19.205cM Marker40730
19.205cM Marker19251
19.205cM Marker51142
19.205cM Marker75862
19.205cM Marker50326
19.205cM Marker28090
19.205cM Marker35174
19.205cM Marker25888
19.205cM Marker13555
19.205cM Marker28264
19.205cM Marker48614
19.205cM Marker59013
19.398cM Marker15572
19.582cM Marker68443
19.949cM Marker42242
20.198cM Marker53564
20.198cM Marker44209
20.198cM Marker33467
20.198cM Marker68257
20.198cM Marker38516
20.198cM Marker68940
20.198cM Marker34565
22.218cM Marker26160
24.239cM Marker32825
24.239cM Marker11285
24.239cM Marker30446
24.239cM Marker36135
24.239cM Marker57374
24.263cM Marker11405
35.272cM Marker36107
35.277cM Marker71730
35.277cM Marker52662
35.277cM Marker28109
35.277cM Marker46407
35.277cM Marker23406
35.277cM Marker24657
35.277cM Marker47848
35.277cM Marker43473
35.277cM Marker8069
36.277cM Marker76080
38.298cM Marker8146
43.149cM Marker59520
45.496cM Marker41747
45.530cM Marker7977
45.530cM Marker34181
45.530cM Marker49365
49.244cM Marker25616
51.710cM Marker25506
62.743cM Marker33345
62.748cM Marker58569
62.803cM Marker6110
64.777cM Marker15525
66.782cM Marker29439
66.807cM Marker12120
66.807cM Marker20303
66.807cM Marker18640
67.807cM Marker53190
67.807cM Marker73346
67.807cM Marker41892
67.827cM Marker41738
69.288cM Marker25266
71.474cM Marker62746
71.847cM Marker26451
71.847cM Marker22383
72.140cM Marker23442
72.403cM Marker26235
72.840cM Marker24225
73.840cM Marker64313
73.840cM Marker26797
73.840cM Marker53411
73.840cM Marker22515
73.840cM Marker17632
73.840cM Marker72932
73.840cM Marker28818
73.840cM Marker47846
73.875cM Marker41277
76.612cM Marker60386

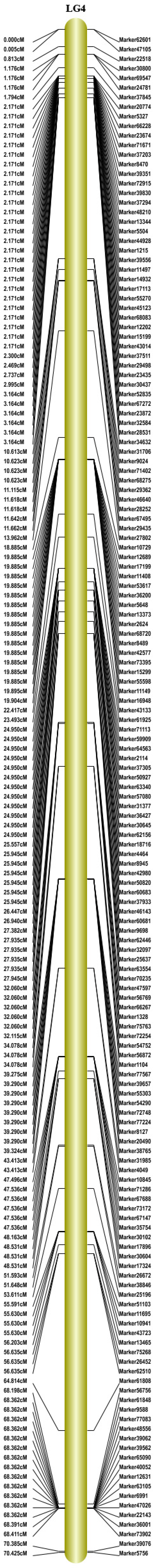
LG4
0.000cM Marker62601
0.005cM Marker47105
0.813cM Marker22518
1.176cM Marker30800
1.176cM Marker69547
1.176cM Marker24781
1.794cM Marker37845
2.171cM Marker20774
2.171cM Marker5327
2.171cM Marker66228
2.171cM Marker23674
2.171cM Marker71671
2.171cM Marker37203
2.171cM Marker8470
2.171cM Marker39351
2.171cM Marker72915
2.171cM Marker39830
2.171cM Marker37294
2.171cM Marker48210
2.171cM Marker13344
2.171cM Marker5504
2.171cM Marker44928
2.171cM Marker1215
2.171cM Marker39556
2.171cM Marker11497
2.171cM Marker14932
2.171cM Marker17113
2.171cM Marker55270
2.171cM Marker45123
2.171cM Marker68083
2.171cM Marker12202
2.171cM Marker15199
2.171cM Marker43014
2.300cM Marker37511
2.469cM Marker29498
2.737cM Marker23435
2.995cM Marker30437
3.164cM Marker52835
3.164cM Marker67272
3.164cM Marker23872
3.164cM Marker32584
3.164cM Marker28531
3.164cM Marker34632
10.613cM Marker31706
10.623cM Marker9024
10.623cM Marker71402
10.623cM Marker68275
11.115cM Marker29362
11.618cM Marker46640
11.618cM Marker28252
11.642cM Marker67495
11.662cM Marker29435
13.962cM Marker27802
18.885cM Marker10729
19.885cM Marker12689
19.885cM Marker17199
19.885cM Marker11408
19.885cM Marker53617
19.885cM Marker36200
19.885cM Marker5648
19.885cM Marker13373
19.885cM Marker2624
19.885cM Marker68720
19.885cM Marker8489
19.885cM Marker42577
19.885cM Marker73395
19.885cM Marker15299
19.885cM Marker55598
19.895cM Marker11149
19.904cM Marker16948
22.417cM Marker43133
23.493cM Marker61925
24.950cM Marker71113
24.950cM Marker59909
24.950cM Marker64563
24.950cM Marker2114
24.950cM Marker37305
24.950cM Marker50927
24.950cM Marker63340
24.950cM Marker57080
24.950cM Marker31377
24.950cM Marker36427
24.950cM Marker30645
24.950cM Marker62156
25.557cM Marker18716
25.945cM Marker4464
25.945cM Marker8945
25.945cM Marker42980
25.945cM Marker50820
25.945cM Marker60683
25.945cM Marker37933
26.447cM Marker46143
26.940cM Marker60681
27.382cM Marker9698
27.935cM Marker62446
27.935cM Marker32097
27.935cM Marker25637
27.935cM Marker63554
27.945cM Marker70235
32.060cM Marker47597
32.060cM Marker56769
32.060cM Marker66267
32.060cM Marker1328
32.060cM Marker75763
32.115cM Marker72254
34.078cM Marker54752
34.078cM Marker56872
34.078cM Marker1104
39.275cM Marker77567
39.290cM Marker39657
39.290cM Marker55303
39.290cM Marker54290
39.290cM Marker72748
39.290cM Marker77224
39.290cM Marker8127
39.290cM Marker20490
39.324cM Marker38765
43.413cM Marker31985
43.413cM Marker4049
47.496cM Marker10845
47.536cM Marker71286
47.536cM Marker67688
47.536cM Marker73172
47.536cM Marker67147
47.536cM Marker35754
48.163cM Marker30102
48.531cM Marker17896
48.531cM Marker30604
48.531cM Marker17324
51.593cM Marker26672
51.648cM Marker38846
53.611cM Marker25196
55.591cM Marker51103
55.630cM Marker11695
55.630cM Marker10941
55.630cM Marker43723
56.203cM Marker13465
56.635cM Marker75268
56.635cM Marker26452
56.635cM Marker62510
64.814cM Marker61808
68.198cM Marker56756
68.362cM Marker61848
68.362cM Marker9588
68.362cM Marker77083
68.362cM Marker48556
68.362cM Marker39062
68.362cM Marker39562
68.362cM Marker65090
68.362cM Marker40052
68.362cM Marker12631
68.362cM Marker63105
68.362cM Marker6991
68.362cM Marker47026
68.362cM Marker22143
68.391cM Marker36001
68.411cM Marker73902
70.385cM Marker39076
70.425cM Marker5756

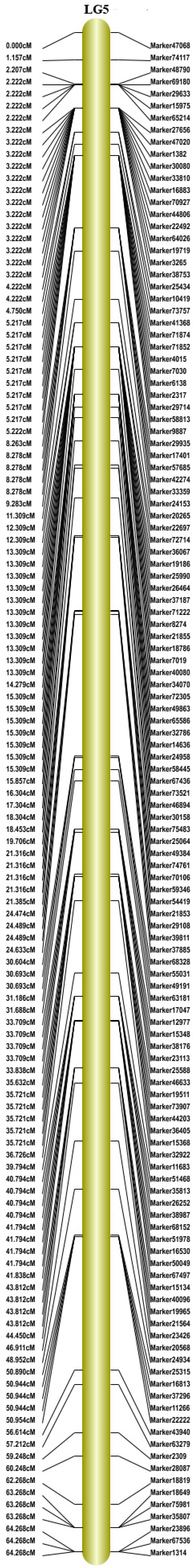
LG5
0.000cM
1.157cM
2.207cM
2.222cM
2.222cM
2.222cM
2.222cM
3.222cM
3.222cM
3.222cM
3.222cM
3.222cM
3.222cM
3.222cM
3.222cM
3.222cM
3.222cM
3.222cM
3.222cM
3.222cM
4.222cM
4.222cM
4.750cM
5.217cM
5.217cM
5.217cM
5.217cM
5.217cM
5.217cM
5.217cM
5.217cM
5.217cM
5.222cM
8.263cM
8.278cM
8.278cM
8.278cM
8.278cM
9.283cM
11.309cM
12.309cM
12.309cM
13.309cM
13.309cM
13.309cM
13.309cM
13.309cM
13.309cM
13.309cM
13.309cM
13.309cM
13.309cM
13.309cM
14.279cM
15.309cM
15.309cM
15.309cM
15.309cM
15.309cM
15.309cM
15.309cM
15.857cM
16.304cM
17.304cM
18.304cM
18.453cM
19.706cM
21.316cM
21.316cM
21.316cM
21.316cM
21.385cM
24.474cM
24.489cM
24.489cM
24.633cM
30.604cM
30.693cM
30.693cM
31.186cM
31.688cM
33.709cM
33.709cM
33.709cM
33.709cM
33.838cM
35.632cM
35.721cM
35.721cM
35.721cM
35.721cM
35.721cM
36.726cM
39.794cM
40.794cM
40.794cM
40.794cM
40.794cM
41.794cM
41.794cM
41.794cM
41.794cM
41.838cM
43.812cM
43.812cM
43.812cM
43.812cM
44.450cM
46.911cM
48.952cM
50.890cM
50.944cM
50.944cM
50.944cM
50.954cM
56.614cM
57.212cM
59.248cM
60.248cM
62.268cM
63.268cM
63.268cM
63.268cM
64.268cM
64.268cM
64.268cM
Marker47068
Marker74117
Marker48790
Marker69180
Marker29633
Marker15975
Marker65214
Marker27656
Marker47020
Marker1382
Marker30080
Marker33810
Marker16883
Marker70927
Marker44806
Marker22492
Marker64026
Marker19719
Marker3265
Marker38753
Marker25434
Marker10419
Marker73757
Marker41368
Marker71874
Marker71852
Marker4015
Marker7030
Marker6138
Marker2317
Marker29714
Marker58813
Marker9887
Marker29935
Marker17401
Marker57685
Marker42274
Marker33359
Marker24153
Marker20265
Marker22697
Marker72714
Marker36067
Marker19186
Marker25990
Marker26464
Marker37187
Marker71222
Marker8274
Marker21855
Marker18786
Marker7019
Marker40080
Marker34070
Marker72305
Marker49863
Marker65586
Marker32786
Marker14636
Marker24958
Marker58445
Marker67436
Marker73521
Marker46894
Marker30158
Marker75483
Marker25064
Marker49384
Marker74761
Marker70106
Marker59346
Marker54419
Marker21853
Marker29108
Marker39811
Marker37885
Marker68328
Marker55031
Marker49191
Marker63181
Marker17047
Marker12977
Marker15348
Marker38176
Marker23113
Marker25588
Marker46633
Marker19511
Marker73907
Marker44203
Marker36405
Marker15368
Marker32922
Marker11683
Marker51468
Marker35813
Marker26252
Marker38987
Marker68152
Marker51978
Marker16530
Marker50049
Marker67497
Marker15134
Marker40096
Marker19965
Marker21564
Marker23426
Marker20568
Marker24934
Marker25315
Marker16813
Marker37296
Marker11266
Marker22222
Marker43940
Marker63279
Marker2309
Marker28087
Marker18819
Marker18649
Marker75981
Marker35807
Marker23896
Marker67536
Marker1314

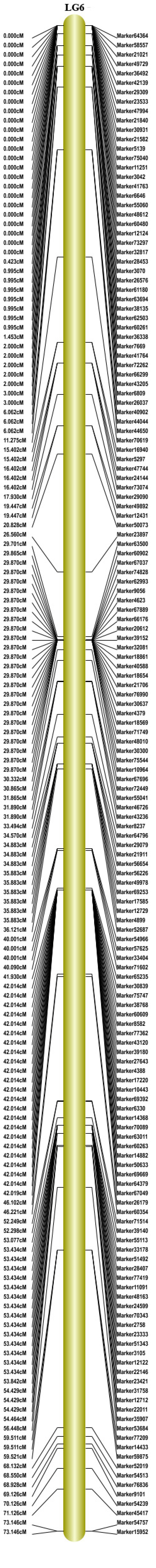
LG6
0.000cM Marker64364
0.000cM Marker58557
0.000cM Marker21021
0.000cM Marker49729
0.000cM Marker36492
0.000cM Marker42139
0.000cM Marker29309
0.000cM Marker23533
0.000cM Marker47994
0.000cM Marker21840
0.000cM Marker30931
0.000cM Marker21582
0.000cM Marker5139
0.000cM Marker75040
0.000cM Marker11251
0.000cM Marker3042
0.000cM Marker41763
0.000cM Marker6646
0.000cM Marker55060
0.000cM Marker48612
0.000cM Marker60480
0.000cM Marker12124
0.000cM Marker73297
0.000cM Marker32817
0.423cM Marker28453
0.995cM Marker3070
0.995cM Marker26576
0.995cM Marker61180
0.995cM Marker63694
0.995cM Marker38135
0.995cM Marker62503
0.995cM Marker60261
1.453cM Marker36338
2.000cM Marker7669
2.000cM Marker41764
2.000cM Marker72262
2.000cM Marker66299
2.000cM Marker43205
3.000cM Marker6809
3.000cM Marker26037
6.062cM Marker40902
6.062cM Marker44044
6.062cM Marker44650
11.275cM Marker70619
15.402cM Marker16940
15.402cM Marker5297
16.402cM Marker47744
16.402cM Marker24144
16.402cM Marker73074
17.930cM Marker29090
19.447cM Marker49892
19.447cM Marker12431
20.828cM Marker50073
26.560cM Marker23897
29.701cM Marker63500
29.865cM Marker60902
29.870cM Marker67037
29.870cM Marker74828
29.870cM Marker62993
29.870cM Marker9056
29.870cM Marker4623
29.870cM Marker67889
29.870cM Marker66176
29.870cM Marker20612
29.870cM Marker39152
29.870cM Marker32081
29.870cM Marker18861
29.870cM Marker40588
29.870cM Marker18654
29.870cM Marker21706
29.870cM Marker76990
29.870cM Marker30637
29.870cM Marker4379
29.870cM Marker18569
29.870cM Marker71749
29.870cM Marker48010
29.870cM Marker30300
29.870cM Marker75544
29.870cM Marker10964
30.332cM Marker67696
30.865cM Marker72449
31.865cM Marker55041
31.890cM Marker46726
31.890cM Marker43236
33.494cM Marker8237
34.570cM Marker64796
34.883cM Marker29079
34.883cM Marker21911
34.883cM Marker56654
35.883cM Marker56226
35.883cM Marker49978
35.883cM Marker69253
35.883cM Marker17585
35.883cM Marker12729
35.883cM Marker4899
36.121cM Marker52687
40.001cM Marker54966
40.001cM Marker57625
40.001cM Marker33404
40.090cM Marker71602
41.930cM Marker65235
42.014cM Marker30839
42.014cM Marker75747
42.014cM Marker38768
42.014cM Marker60609
42.014cM Marker8582
42.014cM Marker77362
42.014cM Marker43120
42.014cM Marker39180
42.014cM Marker27643
42.014cM Marker4388
42.014cM Marker17220
42.014cM Marker10443
42.014cM Marker69392
42.014cM Marker6330
42.014cM Marker14368
42.014cM Marker70089
42.014cM Marker63011
42.014cM Marker60263
42.014cM Marker14882
42.014cM Marker50633
42.014cM Marker69669
42.014cM Marker64379
42.019cM Marker67049
46.102cM Marker26179
46.221cM Marker60354
52.249cM Marker71514
52.298cM Marker39140
53.077cM Marker55113
53.434cM Marker33178
53.434cM Marker51492
53.434cM Marker28407
53.434cM Marker77419
53.434cM Marker11091
53.434cM Marker48163
53.434cM Marker24599
53.434cM Marker70343
53.434cM Marker2758
53.434cM Marker23333
53.434cM Marker51343
53.434cM Marker3105
53.434cM Marker12122
53.434cM Marker22146
53.842cM Marker23421
54.429cM Marker31758
54.429cM Marker12712
54.429cM Marker22011
54.464cM Marker35907
56.448cM Marker53684
59.511cM Marker77209
59.511cM Marker14433
59.521cM Marker59875
68.132cM Marker52019
68.550cM Marker54513
68.928cM Marker76836
69.126cM Marker9101
70.126cM Marker54239
71.126cM Marker45417
73.146cM Marker54757
73.146cM Marker15952

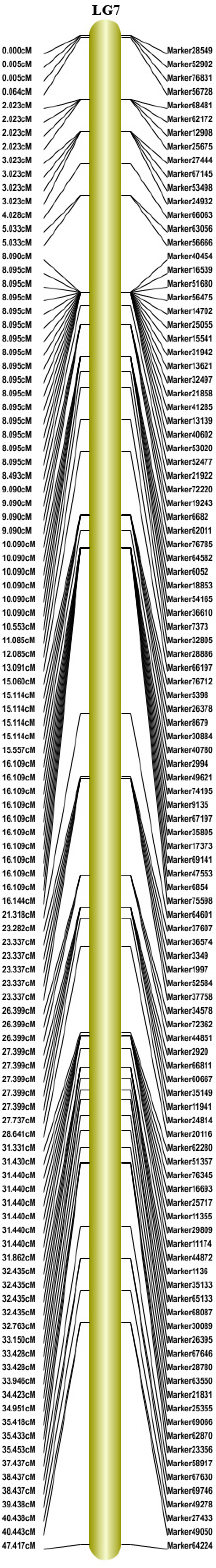
LG7
0.000cM
0.005cM
0.005cM
0.064cM
2.023cM
2.023cM
2.023cM
2.023cM
3.023cM
3.023cM
3.023cM
3.023cM
4.028cM
5.033cM
5.033cM
8.090cM
8.095cM
8.095cM
8.095cM
8.095cM
8.095cM
8.095cM
8.095cM
8.095cM
8.095cM
8.095cM
8.095cM
8.095cM
8.095cM
8.095cM
8.095cM
8.493cM
9.090cM
9.090cM
9.090cM
9.090cM
10.090cM
10.090cM
10.090cM
10.090cM
10.090cM
10.090cM
10.553cM
11.085cM
12.085cM
13.091cM
15.060cM
15.114cM
15.114cM
15.114cM
15.114cM
15.557cM
16.109cM
16.109cM
16.109cM
16.109cM
16.109cM
16.109cM
16.109cM
16.109cM
16.109cM
16.109cM
16.144cM
21.318cM
23.282cM
23.337cM
23.337cM
23.337cM
23.337cM
23.337cM
26.399cM
26.399cM
26.399cM
27.399cM
27.399cM
27.399cM
27.399cM
27.399cM
27.737cM
28.641cM
31.331cM
31.430cM
31.440cM
31.440cM
31.440cM
31.440cM
31.440cM
31.440cM
31.862cM
32.435cM
32.435cM
32.435cM
32.435cM
32.763cM
33.150cM
33.428cM
33.428cM
33.946cM
34.423cM
34.951cM
35.418cM
35.433cM
35.453cM
37.437cM
38.437cM
38.437cM
39.438cM
40.438cM
40.443cM
47.417cM
Marker28549
Marker52902
Marker76831
Marker56728
Marker68481
Marker62172
Marker12908
Marker25675
Marker27444
Marker67145
Marker53498
Marker24932
Marker66063
Marker63056
Marker56666
Marker40454
Marker16539
Marker51680
Marker56475
Marker14702
Marker25055
Marker15541
Marker31942
Marker13621
Marker32497
Marker21858
Marker41285
Marker13139
Marker40602
Marker53020
Marker52477
Marker21922
Marker72220
Marker19243
Marker6682
Marker62011
Marker76785
Marker64582
Marker6052
Marker18853
Marker54165
Marker36610
Marker7373
Marker32805
Marker28886
Marker66197
Marker76712
Marker5398
Marker26378
Marker8679
Marker30884
Marker40780
Marker2994
Marker49621
Marker74195
Marker9135
Marker67197
Marker35805
Marker17373
Marker69141
Marker47553
Marker6854
Marker75598
Marker64601
Marker37607
Marker36574
Marker3349
Marker1997
Marker52584
Marker37758
Marker34578
Marker72362
Marker44851
Marker2920
Marker66811
Marker60667
Marker35149
Marker11941
Marker24814
Marker20116
Marker62280
Marker51357
Marker76345
Marker16693
Marker25717
Marker11355
Marker29809
Marker11174
Marker44872
Marker1136
Marker35133
Marker65133
Marker68087
Marker30089
Marker26395
Marker67646
Marker28780
Marker63550
Marker21831
Marker25355
Marker69066
Marker62870
Marker23356
Marker58917
Marker67630
Marker69746
Marker49278
Marker27433
Marker49050
Marker64224

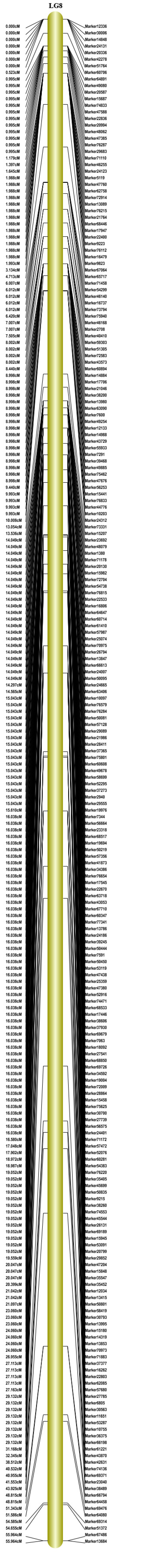
LG8
0.000cM Marker12336
0.000cM Marker30006
0.000cM Marker14848
0.000cM Marker24131
0.000cM Marker20336
0.000cM Marker42278
0.000cM Marker51764
0.523cM Marker60706
0.995cM Marker64891
0.995cM Marker49080
0.995cM Marker20587
0.995cM Marker15687
0.995cM Marker74833
0.995cM Marker47588
0.995cM Marker22836
0.995cM Marker20994
0.995cM Marker48062
0.995cM Marker47385
0.995cM Marker76287
0.995cM Marker29683
1.179cM Marker71110
1.397cM Marker46255
1.645cM Marker24123
1.988cM Marker5119
1.988cM Marker47760
1.988cM Marker62758
1.988cM Marker72914
1.988cM Marker13089
1.988cM Marker76215
1.988cM Marker21764
1.988cM Marker68446
1.988cM Marker17947
1.988cM Marker22400
1.988cM Marker9223
1.988cM Marker76112
1.988cM Marker16479
1.993cM Marker9823
3.134cM Marker67064
4.713cM Marker65717
6.007cM Marker71458
6.012cM Marker54299
6.012cM Marker46140
6.012cM Marker16737
6.012cM Marker73794
6.429cM Marker75940
7.007cM Marker46168
7.007cM Marker2708
7.505cM Marker40410
8.002cM Marker59303
8.002cM Marker51305
8.002cM Marker72583
8.002cM Marker43573
8.440cM Marker60894
8.998cM Marker14884
8.998cM Marker17706
8.998cM Marker21046
8.998cM Marker38200
8.998cM Marker13980
8.998cM Marker63090
8.998cM Marker7600
8.998cM Marker49254
8.998cM Marker12133
8.998cM Marker14988
8.998cM Marker43729
8.998cM Marker55933
8.998cM Marker7291
8.998cM Marker39468
8.998cM Marker49885
8.998cM Marker75462
8.998cM Marker47676
9.440cM Marker56253
9.993cM Marker15441
9.993cM Marker76833
9.993cM Marker44776
9.993cM Marker10203
10.008cM Marker24312
13.054cM Marker73331
13.536cM Marker15207
14.049cM Marker23692
14.049cM Marker48079
14.049cM Marker1380
14.049cM Marker71178
14.049cM Marker20130
14.049cM Marker15982
14.049cM Marker72704
14.049cM Marker54738
14.049cM Marker76815
14.049cM Marker22533
14.049cM Marker16806
14.049cM Marker64647
14.049cM Marker60714
14.049cM Marker61410
14.049cM Marker57987
14.049cM Marker25074
14.049cM Marker70975
14.049cM Marker26794
14.049cM Marker13847
14.049cM Marker66613
14.049cM Marker24007
14.049cM Marker50095
14.297cM Marker24665
14.565cM Marker63406
15.043cM Marker10097
15.043cM Marker76579
15.043cM Marker76284
15.043cM Marker50081
15.043cM Marker57128
15.043cM Marker29089
15.043cM Marker21986
15.043cM Marker28411
15.043cM Marker37365
15.043cM Marker75801
15.043cM Marker60608
15.043cM Marker49678
15.043cM Marker58699
15.043cM Marker52295
15.043cM Marker37273
15.043cM Marker2940
15.043cM Marker29555
15.610cM Marker19976
16.038cM Marker7344
16.038cM Marker56664
16.038cM Marker23318
16.038cM Marker68517
16.038cM Marker19694
16.038cM Marker50219
16.038cM Marker57356
16.038cM Marker41873
16.038cM Marker34386
16.038cM Marker76654
16.038cM Marker17545
16.038cM Marker22670
16.038cM Marker63718
16.038cM Marker43053
16.038cM Marker67710
16.038cM Marker60347
16.038cM Marker77341
16.038cM Marker13786
16.038cM Marker24186
16.038cM Marker39245
16.038cM Marker50444
16.038cM Marker7591
16.038cM Marker50450
16.038cM Marker53119
16.038cM Marker47438
16.038cM Marker25359
16.038cM Marker47380
16.038cM Marker52916
16.038cM Marker74471
16.038cM Marker68533
16.038cM Marker17446
16.038cM Marker38606
16.038cM Marker37930
16.038cM Marker69679
16.038cM Marker7063
16.038cM Marker18092
16.038cM Marker27541
16.038cM Marker68850
16.038cM Marker69726
16.038cM Marker34592
16.038cM Marker19004
16.038cM Marker72009
16.038cM Marker28864
16.038cM Marker15458
16.038cM Marker75625
16.038cM Marker30700
16.038cM Marker27739
16.038cM Marker56575
16.038cM Marker24401
16.580cM Marker71172
17.046cM Marker57472
17.902cM Marker52076
18.972cM Marker60281
18.987cM Marker54383
19.052cM Marker76220
19.052cM Marker35405
19.052cM Marker45699
19.052cM Marker50835
19.052cM Marker9215
19.052cM Marker38260
19.052cM Marker74553
19.052cM Marker45544
19.052cM Marker26131
19.052cM Marker69189
19.052cM Marker15945
19.052cM Marker53091
19.052cM Marker20799
19.559cM Marker29852
20.047cM Marker47204
20.047cM Marker15848
20.047cM Marker35547
20.399cM Marker35452
21.042cM Marker12034
21.042cM Marker13415
21.097cM Marker50801
23.060cM Marker58419
23.060cM Marker30793
23.060cM Marker13995
24.060cM Marker15180
24.060cM Marker14319
24.060cM Marker13853
24.060cM Marker70973
26.955cM Marker71883
27.113cM Marker37377
27.113cM Marker16262
27.113cM Marker22803
27.113cM Marker62085
27.163cM Marker57680
29.132cM Marker27785
29.132cM Marker6805
29.132cM Marker30563
29.132cM Marker11651
29.132cM Marker53287
29.132cM Marker10755
29.132cM Marker36375
29.132cM Marker66198
31.168cM Marker61221
32.345cM Marker43870
36.512cM Marker42631
40.532cM Marker74136
40.955cM Marker69371
41.553cM Marker23040
43.925cM Marker38489
48.815cM Marker66794
48.815cM Marker64458
51.343cM Marker69476
51.586cM Marker64080
54.585cM Marker69314
54.655cM Marker51372
55.964cM Marker67486
55.964cM Marker13684

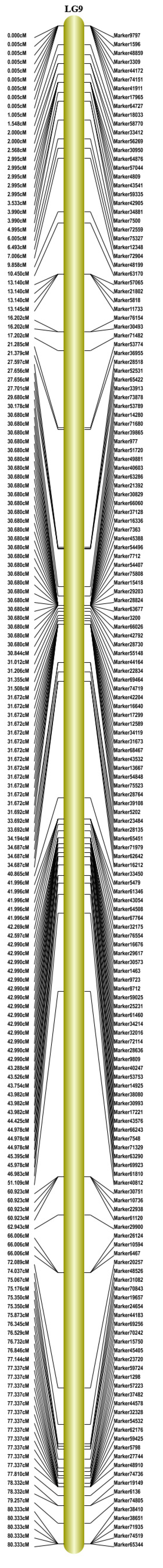
LG9
0.000cM Marker9797
0.005cM Marker1596
0.005cM Marker48859
0.005cM Marker3309
0.005cM Marker44172
0.005cM Marker74151
0.005cM Marker41911
0.005cM Marker17965
0.005cM Marker64727
1.005cM Marker18033
1.548cM Marker58770
2.000cM Marker33412
2.000cM Marker56269
2.568cM Marker30950
2.995cM Marker64876
2.995cM Marker57044
2.995cM Marker4809
2.995cM Marker43541
2.995cM Marker59335
3.533cM Marker42905
3.990cM Marker34881
3.990cM Marker7500
4.995cM Marker72559
6.005cM Marker75327
6.493cM Marker12348
7.006cM Marker72904
9.858cM Marker48199
10.450cM Marker63170
13.140cM Marker57065
13.140cM Marker21802
13.140cM Marker5818
13.145cM Marker11733
16.202cM Marker76154
16.202cM Marker30493
17.202cM Marker71482
21.285cM Marker53774
21.379cM Marker36955
27.597cM Marker28518
27.656cM Marker52531
27.656cM Marker65422
27.701cM Marker33913
29.680cM Marker73878
30.178cM Marker53789
30.680cM Marker14280
30.680cM Marker71680
30.680cM Marker39865
30.680cM Marker977
30.680cM Marker51720
30.680cM Marker49881
30.680cM Marker40603
30.680cM Marker63286
30.680cM Marker21392
30.680cM Marker30829
30.680cM Marker66060
30.680cM Marker37128
30.680cM Marker16336
30.680cM Marker7363
30.680cM Marker45388
30.680cM Marker54496
30.680cM Marker7712
30.680cM Marker54407
30.680cM Marker75808
30.680cM Marker15418
30.680cM Marker29203
30.680cM Marker28824
30.680cM Marker63677
30.680cM Marker3200
30.680cM Marker66026
30.680cM Marker42792
30.680cM Marker28730
30.844cM Marker55148
31.012cM Marker44164
31.206cM Marker22834
31.355cM Marker69464
31.508cM Marker74719
31.672cM Marker42204
31.672cM Marker16640
31.672cM Marker17299
31.672cM Marker12589
31.672cM Marker34119
31.672cM Marker31673
31.672cM Marker68467
31.672cM Marker43532
31.672cM Marker13667
31.672cM Marker54848
31.672cM Marker75523
31.672cM Marker28764
31.672cM Marker39108
31.692cM Marker5202
33.692cM Marker23484
33.692cM Marker28135
34.194cM Marker65451
34.687cM Marker71979
34.687cM Marker62642
34.687cM Marker16212
40.865cM Marker33450
41.996cM Marker5479
41.996cM Marker61346
41.996cM Marker43054
41.996cM Marker64508
41.996cM Marker67764
42.269cM Marker32175
42.597cM Marker76554
42.990cM Marker16676
42.990cM Marker29617
42.990cM Marker30573
42.990cM Marker1463
42.990cM Marker9723
42.990cM Marker8712
42.990cM Marker59025
42.990cM Marker25231
42.990cM Marker61460
42.990cM Marker34214
42.990cM Marker32016
42.990cM Marker72114
42.990cM Marker28636
42.990cM Marker9809
43.288cM Marker40247
43.526cM Marker53753
43.754cM Marker14925
43.982cM Marker38080
43.982cM Marker30993
43.982cM Marker17221
44.425cM Marker43576
44.978cM Marker66243
44.978cM Marker7548
44.978cM Marker71329
45.395cM Marker63290
45.978cM Marker69923
46.983cM Marker61810
51.109cM Marker40812
60.923cM Marker30751
60.923cM Marker10736
60.923cM Marker22938
60.923cM Marker61120
62.943cM Marker29900
66.006cM Marker26124
66.006cM Marker10594
66.006cM Marker6467
72.089cM Marker20257
74.037cM Marker48526
75.067cM Marker31082
75.176cM Marker70843
75.350cM Marker19657
75.350cM Marker24654
75.873cM Marker44183
76.345cM Marker69256
76.529cM Marker70242
76.732cM Marker15750
76.846cM Marker45405
77.144cM Marker23720
77.337cM Marker59724
77.337cM Marker1298
77.337cM Marker57223
77.337cM Marker37482
77.337cM Marker44578
77.337cM Marker32328
77.337cM Marker54532
77.337cM Marker62176
77.337cM Marker59425
77.337cM Marker5798
77.337cM Marker27744
77.337cM Marker48910
77.810cM Marker74736
78.332cM Marker19149
78.332cM Marker6136
79.257cM Marker74805
80.333cM Marker38410
80.333cM Marker38651
80.333cM Marker71935
80.333cM Marker74519
80.333cM Marker65344

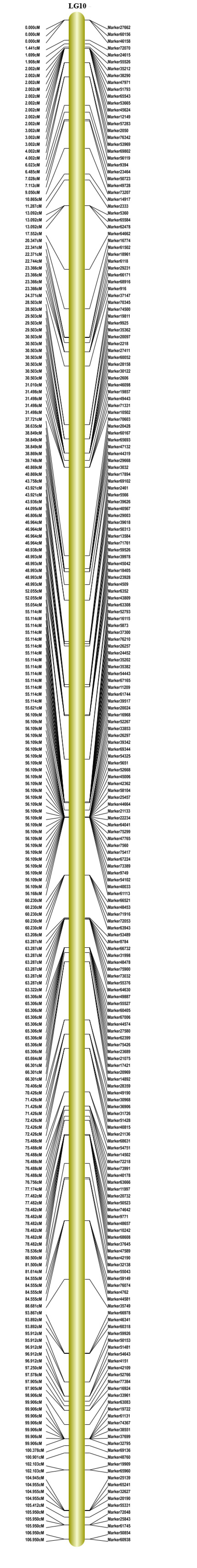
LG10
0.000cM Marker27662
0.000cM Marker60156
0.000cM Marker46158
1.441cM Marker72070
1.699cM Marker24615
1.908cM Marker55526
2.002cM Marker35212
2.002cM Marker38290
2.002cM Marker47971
2.002cM Marker51793
2.002cM Marker65543
2.002cM Marker53665
2.002cM Marker45624
2.002cM Marker12149
2.002cM Marker57283
3.002cM Marker2050
3.002cM Marker76342
3.002cM Marker53969
4.002cM Marker69802
4.002cM Marker56119
6.023cM Marker9394
6.485cM Marker23464
7.028cM Marker50723
7.112cM Marker49728
9.050cM Marker73207
10.865cM Marker14917
11.287cM Marker2333
13.092cM Marker5360
13.092cM Marker65584
13.092cM Marker62478
17.552cM Marker64662
20.347cM Marker16774
22.341cM Marker61502
22.371cM Marker18961
22.744cM Marker6118
23.366cM Marker29231
23.366cM Marker66171
23.366cM Marker68916
23.366cM Marker916
24.371cM Marker37147
28.503cM Marker70345
28.503cM Marker74500
29.503cM Marker19811
29.503cM Marker9925
29.503cM Marker35362
30.503cM Marker20097
30.503cM Marker2218
30.503cM Marker27411
30.503cM Marker60052
30.503cM Marker28158
30.503cM Marker30122
30.503cM Marker2606
31.010cM Marker46098
31.498cM Marker19857
31.498cM Marker49443
31.498cM Marker71331
31.498cM Marker10502
37.721cM Marker70603
38.635cM Marker20428
38.849cM Marker60167
38.849cM Marker65693
38.849cM Marker47132
38.869cM Marker44319
39.748cM Marker29668
40.869cM Marker3032
40.869cM Marker17894
43.758cM Marker69102
43.921cM Marker2461
43.921cM Marker5566
43.936cM Marker39626
44.095cM Marker40567
46.806cM Marker29003
46.964cM Marker39618
46.964cM Marker50313
46.964cM Marker13584
46.964cM Marker71761
48.938cM Marker59526
48.993cM Marker39978
48.993cM Marker45042
48.993cM Marker18405
48.993cM Marker23928
48.993cM Marker4509
52.055cM Marker6352
52.055cM Marker43809
55.054cM Marker63308
55.114cM Marker52793
55.114cM Marker16115
55.114cM Marker5873
55.114cM Marker37300
55.114cM Marker76210
55.114cM Marker26257
55.114cM Marker24452
55.114cM Marker35202
55.114cM Marker35382
55.114cM Marker54443
55.114cM Marker67165
55.114cM Marker11209
55.114cM Marker61744
55.114cM Marker39517
55.621cM Marker20024
56.109cM Marker16968
56.109cM Marker52267
56.109cM Marker33853
56.109cM Marker26297
56.109cM Marker39342
56.109cM Marker69344
56.109cM Marker54325
56.109cM Marker5651
56.109cM Marker52668
56.109cM Marker45006
56.109cM Marker42362
56.109cM Marker58104
56.109cM Marker25457
56.109cM Marker44664
56.109cM Marker21133
56.109cM Marker22234
56.109cM Marker64041
56.109cM Marker75299
56.109cM Marker47765
56.109cM Marker7560
56.109cM Marker75417
56.109cM Marker67224
56.109cM Marker73389
56.109cM Marker9749
56.109cM Marker54102
56.109cM Marker40033
56.168cM Marker61113
60.230cM Marker66521
60.230cM Marker48453
60.230cM Marker71916
60.230cM Marker72053
60.230cM Marker63943
63.208cM Marker53489
63.287cM Marker8784
63.287cM Marker66732
63.287cM Marker31998
63.287cM Marker48478
63.287cM Marker75900
63.287cM Marker73032
63.287cM Marker55376
63.322cM Marker64630
65.306cM Marker49887
65.306cM Marker55527
65.306cM Marker60405
65.306cM Marker67006
65.306cM Marker44574
65.306cM Marker27580
65.306cM Marker62399
65.306cM Marker75426
65.306cM Marker23689
65.664cM Marker21075
66.301cM Marker17421
66.301cM Marker20969
66.301cM Marker14892
70.406cM Marker28359
70.426cM Marker49190
71.426cM Marker30968
71.426cM Marker36906
71.426cM Marker31726
72.426cM Marker51428
72.426cM Marker40815
72.426cM Marker21136
75.488cM Marker68631
75.488cM Marker54751
76.488cM Marker14502
76.488cM Marker72218
76.488cM Marker73991
76.488cM Marker40178
76.756cM Marker63666
77.174cM Marker11997
77.482cM Marker20732
77.482cM Marker50523
78.482cM Marker74642
78.482cM Marker9771
78.482cM Marker48657
78.482cM Marker10242
78.482cM Marker68608
78.482cM Marker37645
78.536cM Marker47589
80.500cM Marker42190
81.500cM Marker32138
81.614cM Marker55043
84.555cM Marker59149
84.555cM Marker76074
84.555cM Marker4762
84.555cM Marker44581
88.681cM Marker35749
93.867cM Marker66978
93.892cM Marker46341
93.892cM Marker60318
95.912cM Marker59926
95.912cM Marker50153
96.912cM Marker51481
96.912cM Marker54643
96.912cM Marker4151
97.250cM Marker42109
97.578cM Marker52766
97.905cM Marker77384
97.905cM Marker16924
98.906cM Marker63961
99.906cM Marker63083
99.906cM Marker19722
99.906cM Marker61131
99.906cM Marker74367
99.906cM Marker38551
99.906cM Marker37699
99.906cM Marker32795
100.378cM Marker69136
100.901cM Marker48760
102.103cM Marker19909
102.103cM Marker65960
104.945cM Marker25139
104.955cM Marker63241
104.955cM Marker32627
104.955cM Marker20190
105.412cM Marker55331
105.950cM Marker72048
105.950cM Marker25843
105.950cM Marker61745
106.950cM Marker50854
106.950cM Marker60938

LG11

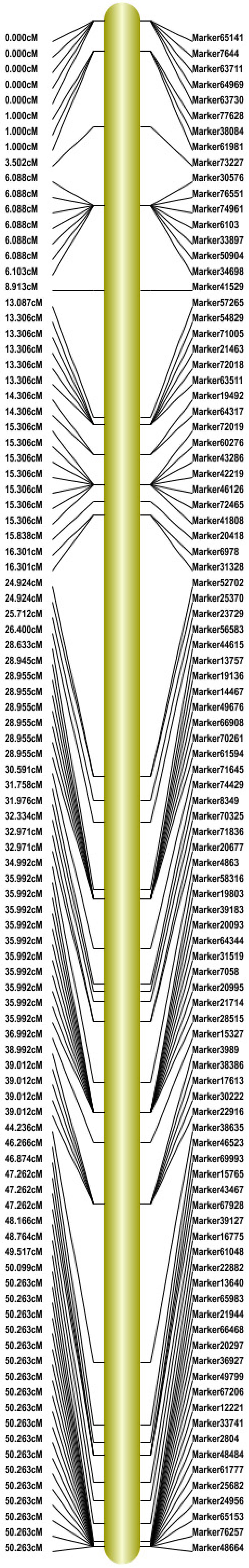
0.000cM
0.000cM
0.000cM
0.000cM
0.000cM
1.000cM
1.000cM
1.000cM
3.502cM
6.088cM
6.088cM
6.088cM
6.088cM
6.088cM
6.088cM
6.103cM
8.913cM
13.087cM
13.306cM
13.306cM
13.306cM
13.306cM
13.306cM
14.306cM
14.306cM
15.306cM
15.306cM
15.306cM
15.306cM
15.306cM
15.306cM
15.306cM
15.838cM
16.301cM
16.301cM
24.924cM
24.924cM
25.712cM
26.400cM
28.633cM
28.945cM
28.955cM
28.955cM
28.955cM
28.955cM
28.955cM
28.955cM
30.591cM
31.758cM
31.976cM
32.334cM
32.971cM
32.971cM
34.992cM
35.992cM
35.992cM
35.992cM
35.992cM
35.992cM
35.992cM
35.992cM
35.992cM
35.992cM
35.992cM
36.992cM
38.992cM
39.012cM
39.012cM
39.012cM
39.012cM
44.236cM
46.266cM
46.874cM
47.262cM
47.262cM
47.262cM
48.166cM
48.764cM
49.517cM
50.099cM
50.263cM
50.263cM
50.263cM
50.263cM
50.263cM
50.263cM
50.263cM
50.263cM
50.263cM
50.263cM
50.263cM
50.263cM
50.263cM
50.263cM
50.263cM
50.263cM
50.263cM
50.263cM
Marker65141
Marker7644
Marker63711
Marker64969
Marker63730
Marker77628
Marker38084
Marker61981
Marker73227
Marker30576
Marker76551
Marker74961
Marker6103
Marker33897
Marker50904
Marker34698
Marker41529
Marker57265
Marker54829
Marker71005
Marker21463
Marker72018
Marker63511
Marker19492
Marker64317
Marker72019
Marker60276
Marker43286
Marker42219
Marker46126
Marker72465
Marker41808
Marker20418
Marker6978
Marker31328
Marker52702
Marker25370
Marker23729
Marker56583
Marker44615
Marker13757
Marker19136
Marker14467
Marker49676
Marker66908
Marker70261
Marker61594
Marker71645
Marker74429
Marker8349
Marker70325
Marker71836
Marker20677
Marker4863
Marker58316
Marker19803
Marker39183
Marker20093
Marker64344
Marker31519
Marker7058
Marker20995
Marker21714
Marker28515
Marker15327
Marker3989
Marker38386
Marker17613
Marker30222
Marker22916
Marker38635
Marker46523
Marker69993
Marker15765
Marker43467
Marker67928
Marker39127
Marker16775
Marker61048
Marker22882
Marker13640
Marker65983
Marker21944
Marker66468
Marker20297
Marker36927
Marker49799
Marker67206
Marker12221
Marker33741
Marker2804
Marker48484
Marker61777
Marker25682
Marker24956
Marker65153
Marker76257
Marker48664

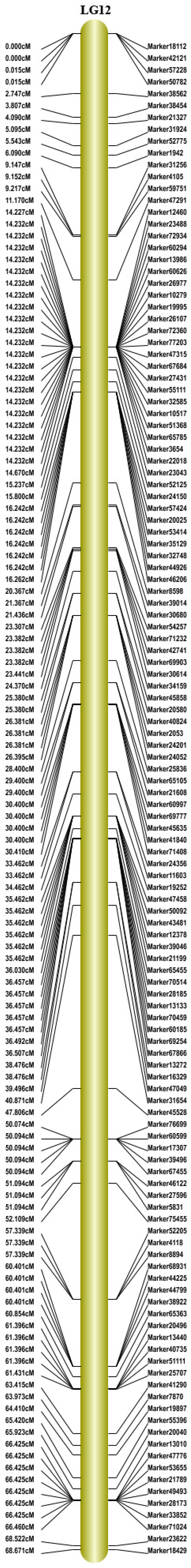
LG12
0.000cM Marker18112
0.000cM Marker42121
0.015cM Marker57228
0.015cM Marker50782
2.747cM Marker38562
3.807cM Marker38454
4.090cM Marker21327
5.095cM Marker31924
5.543cM Marker52775
6.090cM Marker1942
9.147cM Marker31256
9.152cM Marker4105
9.217cM Marker59751
11.170cM Marker47291
14.227cM Marker12460
14.232cM Marker23488
14.232cM Marker72934
14.232cM Marker60294
14.232cM Marker13986
14.232cM Marker60626
14.232cM Marker26977
14.232cM Marker10279
14.232cM Marker19995
14.232cM Marker26107
14.232cM Marker72360
14.232cM Marker77203
14.232cM Marker47315
14.232cM Marker67684
14.232cM Marker27431
14.232cM Marker55111
14.232cM Marker32585
14.232cM Marker10517
14.232cM Marker51368
14.232cM Marker65785
14.232cM Marker3654
14.232cM Marker22018
14.670cM Marker23043
15.237cM Marker52125
15.800cM Marker24150
16.242cM Marker57424
16.242cM Marker20025
16.242cM Marker53414
16.242cM Marker35129
16.242cM Marker32748
16.242cM Marker44926
16.262cM Marker46206
20.367cM Marker8598
21.367cM Marker39014
21.436cM Marker30680
23.307cM Marker54257
23.382cM Marker71232
23.382cM Marker42741
23.382cM Marker69903
23.441cM Marker30614
24.370cM Marker34159
25.380cM Marker45858
25.380cM Marker20580
26.381cM Marker40824
26.381cM Marker2053
26.381cM Marker24201
26.395cM Marker24052
28.400cM Marker25836
29.400cM Marker65105
29.400cM Marker21608
30.400cM Marker60997
30.400cM Marker69777
30.400cM Marker45635
30.400cM Marker41840
30.410cM Marker71408
33.462cM Marker24356
33.462cM Marker11603
34.462cM Marker19252
35.462cM Marker47458
35.462cM Marker50092
35.462cM Marker43481
35.462cM Marker12378
35.462cM Marker39046
35.462cM Marker21199
36.030cM Marker65455
36.457cM Marker70514
36.457cM Marker28185
36.457cM Marker13133
36.457cM Marker70459
36.457cM Marker60185
36.492cM Marker69254
36.507cM Marker67866
38.476cM Marker13272
38.476cM Marker16329
39.496cM Marker47049
40.871cM Marker31654
47.806cM Marker45528
50.074cM Marker76699
50.094cM Marker60599
50.094cM Marker17307
50.094cM Marker39496
50.094cM Marker67455
51.094cM Marker46122
51.094cM Marker27596
51.094cM Marker5831
52.109cM Marker75455
57.339cM Marker52205
57.339cM Marker4118
57.339cM Marker8894
60.401cM Marker68931
60.401cM Marker44225
60.401cM Marker44799
60.401cM Marker38922
60.854cM Marker65363
61.396cM Marker20496
61.396cM Marker13440
61.396cM Marker40735
61.396cM Marker51111
61.431cM Marker25707
63.415cM Marker41290
63.973cM Marker7870
64.410cM Marker19897
65.420cM Marker55396
65.923cM Marker20040
66.425cM Marker13010
66.425cM Marker47776
66.425cM Marker53655
66.425cM Marker21789
66.425cM Marker49493
66.425cM Marker28173
66.425cM Marker33852
66.460cM Marker71024
68.522cM Marker23622
68.671cM Marker18429

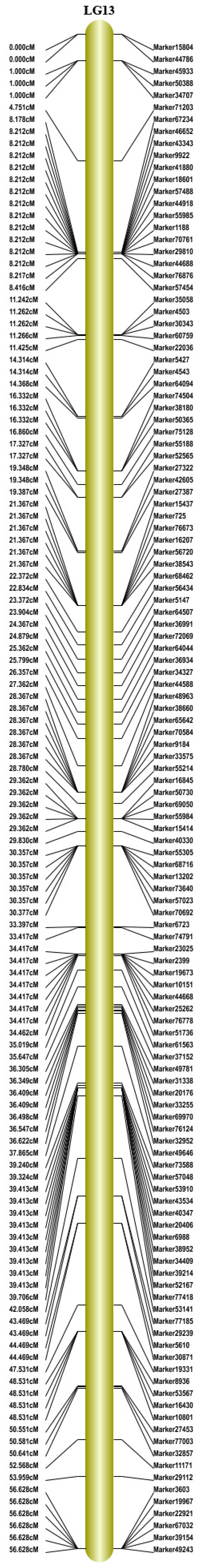
LG13
0.000cM
0.000cM
1.000cM
1.000cM
1.000cM
4.751cM
8.178cM
8.212cM
8.212cM
8.212cM
8.212cM
8.212cM
8.212cM
8.212cM
8.212cM
8.212cM
8.212cM
8.212cM
8.212cM
8.217cM
8.416cM
11.242cM
11.262cM
11.262cM
11.266cM
11.425cM
14.314cM
14.314cM
14.368cM
16.332cM
16.332cM
16.332cM
16.860cM
17.327cM
17.327cM
19.348cM
19.348cM
19.387cM
21.367cM
21.367cM
21.367cM
21.367cM
21.367cM
21.367cM
22.372cM
22.834cM
23.372cM
23.904cM
24.367cM
24.879cM
25.362cM
25.799cM
26.357cM
27.362cM
28.367cM
28.367cM
28.367cM
28.367cM
28.367cM
28.367cM
28.780cM
29.362cM
29.362cM
29.362cM
29.362cM
29.362cM
29.830cM
30.357cM
30.357cM
30.357cM
30.357cM
30.357cM
30.377cM
33.397cM
33.417cM
34.417cM
34.417cM
34.417cM
34.417cM
34.417cM
34.417cM
34.417cM
34.462cM
35.019cM
35.647cM
36.305cM
36.349cM
36.409cM
36.409cM
36.498cM
36.547cM
36.622cM
37.865cM
39.240cM
39.324cM
39.413cM
39.413cM
39.413cM
39.413cM
39.413cM
39.413cM
39.413cM
39.413cM
39.413cM
39.706cM
42.058cM
43.469cM
43.469cM
44.469cM
44.469cM
47.531cM
48.531cM
48.531cM
48.531cM
48.531cM
50.551cM
50.581cM
50.641cM
52.568cM
53.959cM
56.628cM
56.628cM
56.628cM
56.628cM
56.628cM
56.628cM
Marker15804
Marker44786
Marker45933
Marker50388
Marker34707
Marker71203
Marker67234
Marker46652
Marker43343
Marker9922
Marker41880
Marker18601
Marker57488
Marker44918
Marker55985
Marker1188
Marker70761
Marker29810
Marker44688
Marker76876
Marker57454
Marker35058
Marker4503
Marker30343
Marker60759
Marker22036
Marker5427
Marker4543
Marker64094
Marker74504
Marker38180
Marker50365
Marker75128
Marker55188
Marker52565
Marker27322
Marker42605
Marker27387
Marker15437
Marker725
Marker76673
Marker16207
Marker56720
Marker38543
Marker68462
Marker56434
Marker5147
Marker64507
Marker36991
Marker72069
Marker64044
Marker36934
Marker34327
Marker44588
Marker48963
Marker38660
Marker65642
Marker70584
Marker9184
Marker33575
Marker55214
Marker16845
Marker50730
Marker69050
Marker55984
Marker15414
Marker40330
Marker55305
Marker68716
Marker13202
Marker73640
Marker57023
Marker70692
Marker6723
Marker74791
Marker23025
Marker2399
Marker19673
Marker10151
Marker44668
Marker25262
Marker76778
Marker51736
Marker61563
Marker37152
Marker49781
Marker31338
Marker20176
Marker33255
Marker69970
Marker76124
Marker32952
Marker49646
Marker73588
Marker57048
Marker53910
Marker43534
Marker40347
Marker20406
Marker6988
Marker38952
Marker34409
Marker39214
Marker52167
Marker77418
Marker53141
Marker77185
Marker29239
Marker5610
Marker30871
Marker19331
Marker8936
Marker53567
Marker16430
Marker10801
Marker27453
Marker77003
Marker32857
Marker11171
Marker29112
Marker3603
Marker19967
Marker22921
Marker67032
Marker39154
Marker49243

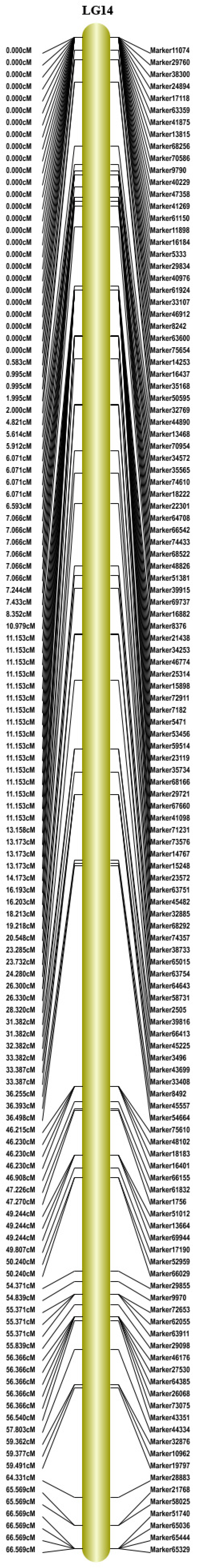
LG14
0.000cM Marker11074
0.000cM Marker29760
0.000cM Marker38300
0.000cM Marker24894
0.000cM Marker17118
0.000cM Marker63359
0.000cM Marker41875
0.000cM Marker13815
0.000cM Marker68256
0.000cM Marker70586
0.000cM Marker9790
0.000cM Marker40229
0.000cM Marker47358
0.000cM Marker41269
0.000cM Marker61150
0.000cM Marker11898
0.000cM Marker16184
0.000cM Marker5333
0.000cM Marker29834
0.000cM Marker40976
0.000cM Marker61924
0.000cM Marker33107
0.000cM Marker46912
0.000cM Marker8242
0.000cM Marker63600
0.000cM Marker75654
0.583cM Marker14253
0.995cM Marker16437
0.995cM Marker35168
1.995cM Marker50595
2.000cM Marker32769
4.821cM Marker44890
5.614cM Marker13468
5.912cM Marker70954
6.071cM Marker34572
6.071cM Marker35565
6.071cM Marker74610
6.071cM Marker18222
6.593cM Marker22301
7.066cM Marker64708
7.066cM Marker66542
7.066cM Marker74433
7.066cM Marker68522
7.066cM Marker48826
7.066cM Marker51381
7.244cM Marker39915
7.433cM Marker69737
8.352cM Marker16882
10.979cM Marker8376
11.153cM Marker21438
11.153cM Marker34253
11.153cM Marker46774
11.153cM Marker25314
11.153cM Marker15898
11.153cM Marker72911
11.153cM Marker7182
11.153cM Marker5471
11.153cM Marker53456
11.153cM Marker59514
11.153cM Marker23119
11.153cM Marker35734
11.153cM Marker68166
11.153cM Marker29721
11.153cM Marker67660
11.153cM Marker41098
13.158cM Marker71231
13.173cM Marker73576
13.173cM Marker14767
13.173cM Marker15248
14.173cM Marker23572
16.193cM Marker63751
16.203cM Marker45482
18.213cM Marker32885
19.218cM Marker68292
20.548cM Marker74357
23.285cM Marker38733
23.732cM Marker65015
24.280cM Marker63754
26.300cM Marker64643
26.330cM Marker58731
28.320cM Marker2505
31.382cM Marker39816
31.382cM Marker66413
32.382cM Marker45225
33.382cM Marker3496
33.387cM Marker43699
33.387cM Marker33408
36.255cM Marker8492
36.393cM Marker45557
36.498cM Marker54664
46.215cM Marker75610
46.230cM Marker48102
46.230cM Marker18183
46.230cM Marker16401
46.908cM Marker66155
47.226cM Marker61832
47.270cM Marker1756
49.244cM Marker51012
49.244cM Marker13664
49.244cM Marker69944
49.807cM Marker17190
50.240cM Marker52959
50.240cM Marker66029
54.371cM Marker29855
54.839cM Marker9970
55.371cM Marker72653
55.371cM Marker62055
55.371cM Marker63911
55.839cM Marker29098
56.366cM Marker46176
56.366cM Marker27530
56.366cM Marker64385
56.366cM Marker26068
56.366cM Marker73075
56.540cM Marker43351
57.803cM Marker44334
59.362cM Marker32876
59.377cM Marker10962
59.491cM Marker19797
64.331cM Marker28883
65.569cM Marker21768
65.569cM Marker58025
66.569cM Marker51740
66.569cM Marker65036
66.569cM Marker65444
66.569cM Marker65329

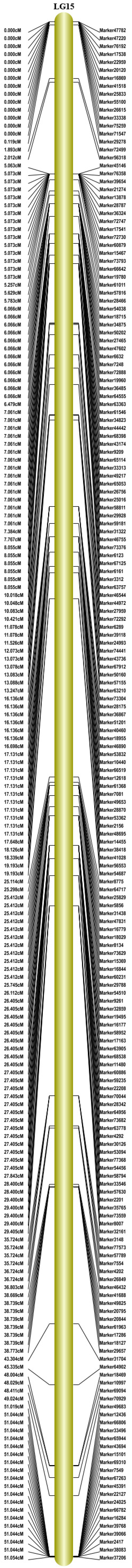
LG15
0.000cM Marker47782
0.000cM Marker47220
0.000cM Marker76192
0.000cM Marker17538
0.000cM Marker22959
0.000cM Marker20120
0.000cM Marker16869
0.000cM Marker41518
0.000cM Marker25833
0.000cM Marker55100
0.000cM Marker26615
0.000cM Marker33338
0.000cM Marker75259
0.000cM Marker71547
0.119cM Marker29278
1.893cM Marker72499
2.012cM Marker56318
5.063cM Marker45146
5.073cM Marker76358
5.073cM Marker39654
5.073cM Marker21274
5.073cM Marker13878
5.073cM Marker28787
5.073cM Marker36324
5.073cM Marker72747
5.073cM Marker17541
5.073cM Marker72730
5.073cM Marker60879
5.073cM Marker15467
5.073cM Marker73793
5.073cM Marker66642
5.073cM Marker19780
5.257cM Marker61011
5.629cM Marker57816
5.783cM Marker28466
6.066cM Marker54038
6.066cM Marker18715
6.066cM Marker34875
6.066cM Marker50202
6.066cM Marker27465
6.066cM Marker47602
6.066cM Marker6632
6.066cM Marker7248
6.066cM Marker72888
6.066cM Marker19960
6.066cM Marker36485
6.066cM Marker64555
6.479cM Marker63363
7.061cM Marker61546
7.061cM Marker34823
7.061cM Marker44442
7.061cM Marker68398
7.061cM Marker43174
7.061cM Marker9209
7.061cM Marker65114
7.061cM Marker33313
7.061cM Marker49217
7.061cM Marker65053
7.061cM Marker26756
7.061cM Marker25016
7.061cM Marker58811
7.061cM Marker29928
7.061cM Marker59181
7.384cM Marker31322
7.767cM Marker46755
8.055cM Marker73376
8.055cM Marker6123
8.055cM Marker67125
8.055cM Marker6161
8.055cM Marker3312
8.055cM Marker63757
10.018cM Marker46544
10.048cM Marker44972
10.083cM Marker27959
10.421cM Marker72292
11.078cM Marker6289
11.078cM Marker39118
11.526cM Marker24993
12.073cM Marker74441
13.073cM Marker43736
13.078cM Marker67912
13.083cM Marker50160
13.088cM Marker57155
13.247cM Marker63210
16.136cM Marker73304
16.136cM Marker28175
16.136cM Marker36867
16.136cM Marker51201
16.136cM Marker40460
16.136cM Marker18955
16.698cM Marker46890
17.131cM Marker53832
17.131cM Marker10440
17.131cM Marker66519
17.131cM Marker12618
17.131cM Marker61368
17.131cM Marker7081
17.131cM Marker49653
17.131cM Marker28870
17.131cM Marker53362
17.131cM Marker2156
17.131cM Marker48695
17.648cM Marker14455
18.126cM Marker38418
18.339cM Marker41028
19.193cM Marker56553
19.193cM Marker54687
25.114cM Marker8775
25.298cM Marker64717
25.412cM Marker25829
25.412cM Marker5856
25.412cM Marker31438
25.412cM Marker47831
25.412cM Marker16779
25.412cM Marker18029
25.412cM Marker8134
25.412cM Marker73629
25.412cM Marker15369
25.412cM Marker16844
25.412cM Marker60231
25.745cM Marker29788
26.112cM Marker54510
26.405cM Marker9261
26.405cM Marker32859
26.405cM Marker19495
26.405cM Marker16177
26.405cM Marker58952
26.405cM Marker17163
26.405cM Marker63905
26.405cM Marker68538
26.405cM Marker11480
27.405cM Marker60886
27.405cM Marker59235
27.405cM Marker22208
27.405cM Marker70044
27.405cM Marker28342
27.405cM Marker64956
27.405cM Marker73682
27.405cM Marker63778
27.405cM Marker4292
27.405cM Marker30126
27.405cM Marker53094
27.405cM Marker77368
27.405cM Marker54456
27.843cM Marker58794
28.400cM Marker33546
28.400cM Marker57630
29.400cM Marker2201
29.400cM Marker35765
29.400cM Marker73559
29.400cM Marker8007
29.405cM Marker32161
35.724cM Marker3148
35.724cM Marker77573
35.724cM Marker57789
35.724cM Marker7554
36.724cM Marker4202
36.724cM Marker26849
36.803cM Marker46432
38.669cM Marker41688
38.739cM Marker49825
38.739cM Marker20795
38.739cM Marker20844
38.739cM Marker61963
38.739cM Marker17286
38.739cM Marker18127
38.773cM Marker29657
43.304cM Marker31704
45.335cM Marker64902
48.004cM Marker18469
48.029cM Marker10997
48.411cM Marker69094
49.024cM Marker70929
51.019cM Marker49683
51.044cM Marker12436
51.044cM Marker66806
51.044cM Marker33496
51.044cM Marker65944
51.044cM Marker43694
51.044cM Marker15101
51.044cM Marker69310
51.044cM Marker7549
51.044cM Marker67263
51.044cM Marker45391
51.044cM Marker22127
51.044cM Marker24025
51.044cM Marker66782
51.044cM Marker16284
51.044cM Marker39768
51.044cM Marker39066
51.044cM Marker2417
51.044cM Marker38083
51.054cM Marker37206

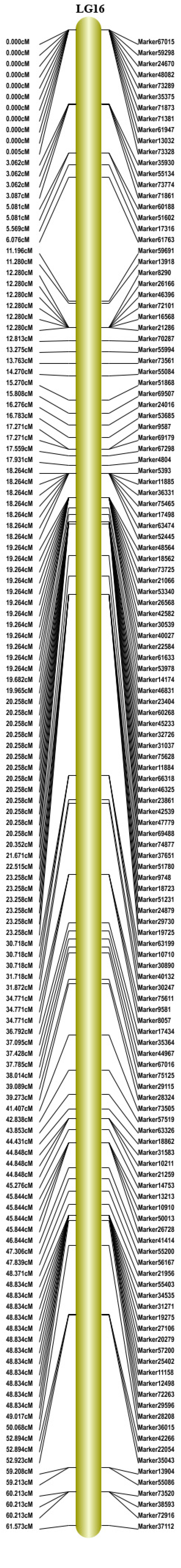
LG16
0.000cM Marker67015
0.000cM Marker59298
0.000cM Marker24670
0.000cM Marker48082
0.000cM Marker73289
0.000cM Marker35375
0.000cM Marker71873
0.000cM Marker71381
0.000cM Marker61947
0.000cM Marker13032
0.005cM Marker73328
3.062cM Marker35930
3.062cM Marker55134
3.062cM Marker73774
3.087cM Marker71861
5.081cM Marker60188
5.081cM Marker51602
5.569cM Marker17316
6.076cM Marker61763
11.196cM Marker59691
11.280cM Marker13918
12.280cM Marker8290
12.280cM Marker26166
12.280cM Marker46396
12.280cM Marker72101
12.280cM Marker16568
12.280cM Marker21286
12.813cM Marker70287
13.275cM Marker55994
13.763cM Marker73561
14.270cM Marker55084
15.270cM Marker51868
15.808cM Marker69507
16.276cM Marker24016
16.783cM Marker53685
17.271cM Marker9587
17.271cM Marker69179
17.559cM Marker67298
17.931cM Marker4804
18.264cM Marker5393
18.264cM Marker11885
18.264cM Marker36331
18.264cM Marker75465
18.264cM Marker17498
18.264cM Marker63474
18.264cM Marker52445
19.264cM Marker48564
19.264cM Marker18562
19.264cM Marker73725
19.264cM Marker21066
19.264cM Marker53340
19.264cM Marker26568
19.264cM Marker42582
19.264cM Marker30539
19.264cM Marker40027
19.264cM Marker22584
19.264cM Marker61633
19.264cM Marker53978
19.682cM Marker14174
19.965cM Marker46831
20.258cM Marker23404
20.258cM Marker60268
20.258cM Marker45233
20.258cM Marker32726
20.258cM Marker31037
20.258cM Marker75628
20.258cM Marker11884
20.258cM Marker66318
20.258cM Marker46325
20.258cM Marker23861
20.258cM Marker42539
20.258cM Marker47779
20.258cM Marker69488
20.352cM Marker74877
21.671cM Marker37651
22.515cM Marker51780
23.258cM Marker9748
23.258cM Marker18723
23.258cM Marker51231
23.258cM Marker24879
23.258cM Marker29730
23.258cM Marker19725
30.718cM Marker63199
30.718cM Marker10710
30.718cM Marker30890
31.718cM Marker40132
31.872cM Marker30247
34.771cM Marker75611
34.771cM Marker9581
34.771cM Marker8057
36.792cM Marker17434
37.095cM Marker35364
37.428cM Marker44967
37.785cM Marker67016
38.014cM Marker75125
39.089cM Marker29115
39.273cM Marker28324
41.407cM Marker73505
42.838cM Marker57519
43.853cM Marker63326
44.431cM Marker18862
44.848cM Marker31583
44.848cM Marker10211
44.848cM Marker21259
45.276cM Marker14753
45.844cM Marker13213
45.844cM Marker10910
45.844cM Marker50013
45.844cM Marker26728
46.844cM Marker41414
47.306cM Marker55200
47.839cM Marker56167
48.371cM Marker21956
48.834cM Marker55403
48.834cM Marker34535
48.834cM Marker31271
48.834cM Marker19275
48.834cM Marker27106
48.834cM Marker20279
48.834cM Marker57200
48.834cM Marker25402
48.834cM Marker11158
48.834cM Marker12498
48.834cM Marker72263
48.834cM Marker29596
49.017cM Marker28208
50.068cM Marker36015
52.894cM Marker42266
52.894cM Marker22054
52.923cM Marker35043
59.208cM Marker13904
59.213cM Marker55086
60.213cM Marker73520
60.213cM Marker38593
60.213cM Marker72916
61.573cM Marker37112

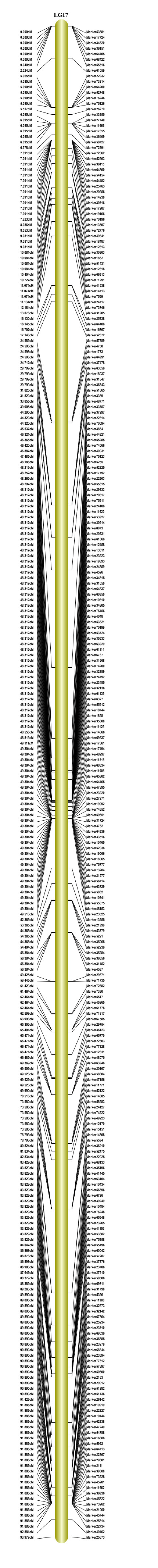
LG17

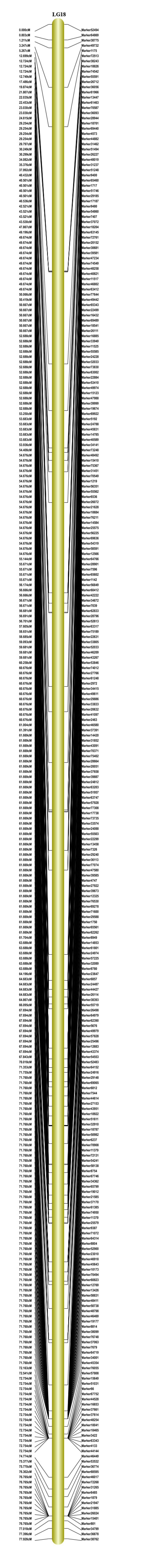
LG18
0.000cM Marker52494
0.803cM Marker64869
1.211cM Marker38775
3.247cM Marker49732
5.267cM Marker1175
12.699cM Marker72513
12.724cM Marker38243
12.724cM Marker18826
12.724cM Marker74542
12.749cM Marker55591
17.486cM Marker26712
19.874cM Marker36056
21.807cM Marker61966
22.035cM Marker13447
22.453cM Marker61463
23.030cM Marker70587
23.030cM Marker36093
24.615cM Marker28944
29.254cM Marker18701
29.254cM Marker69440
29.254cM Marker4573
29.254cM Marker44882
29.797cM Marker31462
30.249cM Marker51494
30.299cM Marker26227
34.082cM Marker48019
35.376cM Marker31237
37.992cM Marker51248
40.432cM Marker9400
40.501cM Marker65460
40.501cM Marker1717
40.501cM Marker51746
40.501cM Marker29185
40.536cM Marker17187
42.521cM Marker9480
43.521cM Marker54860
43.521cM Marker7407
43.530cM Marker37072
47.867cM Marker18204
49.196cM Marker63145
49.674cM Marker72761
49.674cM Marker20152
49.674cM Marker30681
49.674cM Marker39581
49.674cM Marker47234
49.674cM Marker74549
49.674cM Marker48258
49.674cM Marker40821
49.674cM Marker11517
49.674cM Marker46802
49.674cM Marker63412
50.066cM Marker77644
50.419cM Marker49442
50.667cM Marker65343
50.667cM Marker22499
50.667cM Marker16432
50.667cM Marker69489
50.667cM Marker18541
50.667cM Marker26111
52.688cM Marker16885
52.688cM Marker33949
52.688cM Marker11525
52.688cM Marker55585
52.688cM Marker24228
52.688cM Marker32033
52.688cM Marker73830
52.688cM Marker63002
52.688cM Marker22884
52.688cM Marker53410
52.688cM Marker49974
52.688cM Marker15123
52.688cM Marker47988
52.688cM Marker39999
52.688cM Marker19674
53.250cM Marker69922
53.683cM Marker5192
53.683cM Marker24788
53.683cM Marker45831
53.683cM Marker14785
53.683cM Marker40589
53.936cM Marker34141
54.408cM Marker73248
54.676cM Marker48492
54.676cM Marker15410
54.676cM Marker75367
54.676cM Marker31451
54.676cM Marker70549
54.676cM Marker1219
54.676cM Marker56351
54.676cM Marker55562
54.676cM Marker6536
54.676cM Marker26072
54.676cM Marker21628
54.676cM Marker10684
54.676cM Marker76211
54.676cM Marker14584
54.676cM Marker25575
54.676cM Marker56225
54.676cM Marker69636
54.676cM Marker54319
54.676cM Marker58591
54.676cM Marker12566
55.144cM Marker64766
55.671cM Marker28901
55.671cM Marker7596
55.671cM Marker65602
55.671cM Marker1142
56.174cM Marker56849
56.666cM Marker60412
56.666cM Marker42222
56.671cM Marker34672
56.671cM Marker7039
56.681cM Marker62833
56.691cM Marker28796
56.701cM Marker52613
57.605cM Marker63317
58.651cM Marker75189
58.685cM Marker22631
59.093cM Marker33805
59.681cM Marker52033
59.681cM Marker46209
59.681cM Marker43267
60.258cM Marker53846
60.676cM Marker74012
60.676cM Marker27766
60.676cM Marker61248
60.676cM Marker2972
60.676cM Marker34415
60.676cM Marker49611
60.676cM Marker29886
60.676cM Marker33833
60.676cM Marker20632
60.676cM Marker41097
60.676cM Marker2463
61.004cM Marker46580
61.391cM Marker37391
61.669cM Marker14420
61.669cM Marker31852
61.669cM Marker43091
61.669cM Marker70371
61.669cM Marker75402
61.669cM Marker28664
61.669cM Marker20051
61.669cM Marker37658
61.669cM Marker39887
61.669cM Marker24812
61.669cM Marker63203
61.669cM Marker51957
61.669cM Marker63747
61.669cM Marker57928
61.669cM Marker77308
61.669cM Marker17738
61.669cM Marker73735
61.669cM Marker33574
61.669cM Marker24088
61.669cM Marker55503
61.669cM Marker22299
61.669cM Marker13458
61.669cM Marker7326
61.669cM Marker29240
61.669cM Marker36113
61.669cM Marker77074
61.669cM Marker47580
61.669cM Marker39585
61.669cM Marker4747
61.669cM Marker27922
61.669cM Marker39673
61.669cM Marker12325
61.669cM Marker76530
61.669cM Marker69278
61.669cM Marker71600
61.669cM Marker29588
61.669cM Marker1750
61.669cM Marker65561
61.669cM Marker62262
61.704cM Marker8949
63.688cM Marker14853
63.688cM Marker61801
63.688cM Marker24974
63.688cM Marker57225
63.688cM Marker32089
63.688cM Marker8780
64.196cM Marker23647
64.683cM Marker6857
64.683cM Marker24487
64.683cM Marker44427
64.683cM Marker26114
64.867cM Marker38393
66.095cM Marker55719
67.694cM Marker26498
67.694cM Marker64979
67.694cM Marker62390
67.694cM Marker5676
67.694cM Marker49979
67.694cM Marker57828
67.694cM Marker25496
67.694cM Marker12683
67.694cM Marker43374
67.843cM Marker54553
70.018cM Marker52483
71.353cM Marker64152
71.755cM Marker24916
71.760cM Marker29148
71.760cM Marker69065
71.760cM Marker6812
71.760cM Marker7544
71.760cM Marker44614
71.760cM Marker27153
71.760cM Marker43951
71.760cM Marker10922
71.760cM Marker51611
71.760cM Marker32910
71.760cM Marker18787
71.760cM Marker50982
71.760cM Marker6237
71.760cM Marker70908
71.760cM Marker11379
71.760cM Marker72131
71.760cM Marker54241
71.760cM Marker58138
71.760cM Marker8754
71.760cM Marker67746
71.760cM Marker34362
71.760cM Marker65799
71.760cM Marker19012
71.760cM Marker21585
71.760cM Marker37170
71.760cM Marker61385
71.760cM Marker74950
71.760cM Marker11370
71.760cM Marker25579
71.760cM Marker9387
71.760cM Marker71072
71.760cM Marker64314
71.760cM Marker8804
71.760cM Marker52900
71.760cM Marker23010
71.760cM Marker46918
71.760cM Marker45643
71.760cM Marker10173
71.760cM Marker70494
71.760cM Marker60823
71.760cM Marker12769
71.760cM Marker13426
71.760cM Marker58831
71.760cM Marker48411
71.760cM Marker50738
71.760cM Marker48798
71.760cM Marker46469
71.760cM Marker19177
71.760cM Marker8814
71.760cM Marker38099
71.760cM Marker76748
71.760cM Marker37063
71.760cM Marker7679
71.760cM Marker64719
71.760cM Marker34001
71.760cM Marker45354
72.163cM Marker76055
72.541cM Marker57808
72.754cM Marker15649
72.754cM Marker51031
72.754cM Marker66
72.754cM Marker67752
72.754cM Marker44528
72.754cM Marker16653
72.754cM Marker37661
72.754cM Marker37614
72.754cM Marker48254
72.754cM Marker10041
72.754cM Marker18465
72.754cM Marker3422
72.754cM Marker63343
72.754cM Marker4133
72.754cM Marker44144
74.774cM Marker46449
75.377cM Marker53532
75.770cM Marker36774
76.302cM Marker68595
76.765cM Marker46017
76.765cM Marker72268
76.765cM Marker31265
76.765cM Marker6485
76.765cM Marker1879
76.765cM Marker21947
76.765cM Marker31885
76.765cM Marker26024
76.765cM Marker15401
76.765cM Marker801
77.018cM Marker34799
77.396cM Marker36678
77.928cM Marker38762

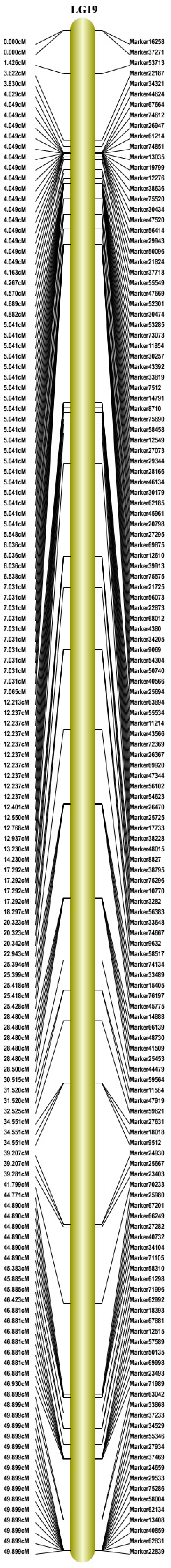
LG19
0.000cM Marker16258
0.000cM Marker37271
1.426cM Marker53713
3.622cM Marker22187
3.830cM Marker34321
4.029cM Marker44624
4.049cM Marker67664
4.049cM Marker74612
4.049cM Marker26947
4.049cM Marker61214
4.049cM Marker74851
4.049cM Marker13035
4.049cM Marker19799
4.049cM Marker12276
4.049cM Marker38636
4.049cM Marker75520
4.049cM Marker30434
4.049cM Marker47520
4.049cM Marker56414
4.049cM Marker29943
4.049cM Marker50096
4.049cM Marker21824
4.163cM Marker37718
4.267cM Marker55549
4.570cM Marker47669
4.689cM Marker52301
4.882cM Marker30474
5.041cM Marker53285
5.041cM Marker73073
5.041cM Marker11854
5.041cM Marker30257
5.041cM Marker43392
5.041cM Marker33819
5.041cM Marker7512
5.041cM Marker14791
5.041cM Marker8710
5.041cM Marker75690
5.041cM Marker58458
5.041cM Marker12549
5.041cM Marker27073
5.041cM Marker29344
5.041cM Marker28166
5.041cM Marker46134
5.041cM Marker30179
5.041cM Marker62185
5.041cM Marker45961
5.041cM Marker20798
5.548cM Marker27295
6.036cM Marker69875
6.036cM Marker12610
6.036cM Marker39913
6.538cM Marker75575
7.031cM Marker21725
7.031cM Marker56073
7.031cM Marker22873
7.031cM Marker68012
7.031cM Marker4380
7.031cM Marker34205
7.031cM Marker9069
7.031cM Marker54304
7.031cM Marker50740
7.031cM Marker40566
7.065cM Marker25694
12.213cM Marker63894
12.237cM Marker55534
12.237cM Marker11214
12.237cM Marker43566
12.237cM Marker72369
12.237cM Marker26367
12.237cM Marker69920
12.237cM Marker47344
12.237cM Marker56102
12.237cM Marker54623
12.401cM Marker26470
12.550cM Marker25725
12.768cM Marker17733
12.937cM Marker38228
13.230cM Marker48015
14.230cM Marker8827
17.292cM Marker38795
17.292cM Marker75296
17.292cM Marker10770
17.292cM Marker3282
18.297cM Marker56383
20.323cM Marker33648
20.323cM Marker74667
20.342cM Marker9632
22.943cM Marker58517
25.394cM Marker74134
25.399cM Marker33489
25.418cM Marker15405
25.418cM Marker76197
25.428cM Marker45775
28.480cM Marker14888
28.480cM Marker66139
28.480cM Marker48730
28.480cM Marker41509
28.480cM Marker25453
28.500cM Marker44479
30.515cM Marker59564
31.520cM Marker11584
31.520cM Marker47919
32.525cM Marker59621
34.551cM Marker27631
34.551cM Marker18018
34.551cM Marker9512
39.207cM Marker24930
39.207cM Marker25667
39.281cM Marker23403
41.799cM Marker70233
44.771cM Marker25980
44.890cM Marker67201
44.890cM Marker66249
44.890cM Marker27282
44.890cM Marker40732
44.890cM Marker34104
44.890cM Marker71105
45.383cM Marker58310
45.885cM Marker61298
45.885cM Marker71996
46.423cM Marker62992
46.881cM Marker18393
46.881cM Marker67881
46.881cM Marker12515
46.881cM Marker57589
46.881cM Marker50135
46.881cM Marker69998
46.881cM Marker23493
46.930cM Marker71989
48.899cM Marker63042
48.899cM Marker33868
49.899cM Marker37233
49.899cM Marker34529
49.899cM Marker55346
49.899cM Marker27934
49.899cM Marker37469
49.899cM Marker24659
49.899cM Marker29533
49.899cM Marker75286
49.899cM Marker58004
49.899cM Marker62134
49.899cM Marker13408
49.899cM Marker40859
49.899cM Marker62831
49.899cM Marker22839

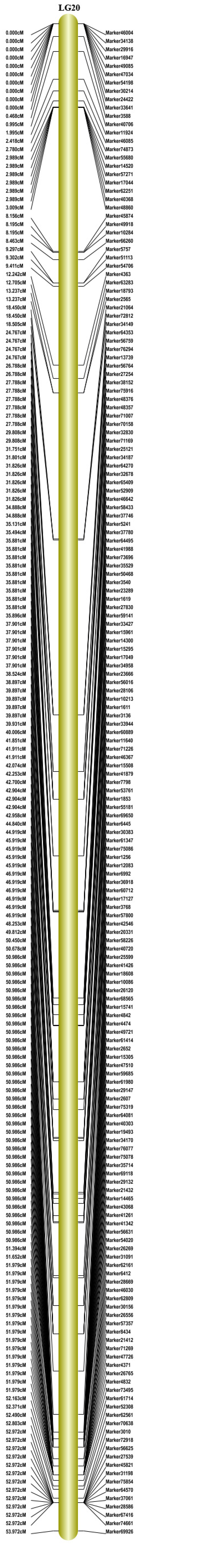
LG20
0.000cM Marker46004
0.000cM Marker34138
0.000cM Marker29916
0.000cM Marker16947
0.000cM Marker49085
0.000cM Marker47034
0.000cM Marker54198
0.000cM Marker30214
0.000cM Marker24422
0.000cM Marker33641
0.468cM Marker3588
0.995cM Marker40706
1.995cM Marker11924
2.418cM Marker46085
2.780cM Marker74873
2.989cM Marker55680
2.989cM Marker14520
2.989cM Marker57271
2.989cM Marker17044
2.989cM Marker62251
2.989cM Marker40368
3.009cM Marker48860
8.156cM Marker45874
8.195cM Marker49918
8.195cM Marker10284
8.463cM Marker66260
9.297cM Marker5757
9.302cM Marker51113
9.411cM Marker54706
12.242cM Marker4363
12.705cM Marker63283
13.237cM Marker18793
13.237cM Marker2565
18.450cM Marker21064
18.450cM Marker72812
18.505cM Marker34149
24.767cM Marker64353
24.767cM Marker56759
24.767cM Marker76294
24.767cM Marker13739
26.788cM Marker56764
26.788cM Marker27254
27.788cM Marker38152
27.788cM Marker75916
27.788cM Marker48376
27.788cM Marker48357
27.788cM Marker71007
27.788cM Marker70158
29.808cM Marker32830
29.808cM Marker71169
31.751cM Marker25121
31.801cM Marker34187
31.826cM Marker64270
31.826cM Marker32678
31.826cM Marker65409
31.826cM Marker52909
31.826cM Marker46642
34.888cM Marker58433
34.888cM Marker37746
35.131cM Marker5241
35.494cM Marker37780
35.881cM Marker64495
35.881cM Marker41988
35.881cM Marker73696
35.881cM Marker35529
35.881cM Marker50468
35.881cM Marker3540
35.881cM Marker23289
35.881cM Marker1619
35.881cM Marker27830
35.896cM Marker59141
37.901cM Marker33427
37.901cM Marker15961
37.901cM Marker14300
37.901cM Marker15295
37.901cM Marker17049
37.901cM Marker34958
38.524cM Marker23666
38.897cM Marker56016
39.897cM Marker28106
39.897cM Marker10213
39.897cM Marker1611
39.897cM Marker3136
39.931cM Marker33944
40.006cM Marker60889
41.851cM Marker11640
41.911cM Marker71226
41.911cM Marker46367
42.074cM Marker15508
42.253cM Marker41879
42.700cM Marker7798
42.904cM Marker53761
42.904cM Marker1853
42.904cM Marker55181
42.958cM Marker69650
44.840cM Marker6445
44.919cM Marker30383
45.919cM Marker61347
45.919cM Marker75086
45.919cM Marker1256
45.919cM Marker12083
46.919cM Marker6992
46.919cM Marker36918
46.919cM Marker60712
46.919cM Marker17127
46.919cM Marker3768
46.919cM Marker57800
48.253cM Marker42546
49.812cM Marker20331
50.450cM Marker58226
50.678cM Marker40720
50.986cM Marker25599
50.986cM Marker41426
50.986cM Marker18608
50.986cM Marker10086
50.986cM Marker26120
50.986cM Marker68565
50.986cM Marker15741
50.986cM Marker4842
50.986cM Marker4474
50.986cM Marker49721
50.986cM Marker61414
50.986cM Marker2652
50.986cM Marker15305
50.986cM Marker47510
50.986cM Marker59685
50.986cM Marker61980
50.986cM Marker29147
50.986cM Marker2607
50.986cM Marker75319
50.986cM Marker64081
50.986cM Marker40303
50.986cM Marker19493
50.986cM Marker34170
50.986cM Marker76077
50.986cM Marker75078
50.986cM Marker35714
50.986cM Marker69118
50.986cM Marker29132
50.986cM Marker21432
50.986cM Marker14465
50.986cM Marker43068
50.986cM Marker41261
50.986cM Marker41342
50.986cM Marker56631
50.986cM Marker54020
51.394cM Marker26269
51.652cM Marker31091
51.979cM Marker62161
51.979cM Marker6412
51.979cM Marker28669
51.979cM Marker46030
51.979cM Marker62809
51.979cM Marker30156
51.979cM Marker26556
51.979cM Marker57357
51.979cM Marker8434
51.979cM Marker21412
51.979cM Marker71269
51.979cM Marker47726
51.979cM Marker4371
51.979cM Marker26765
51.979cM Marker4832
51.979cM Marker73495
52.163cM Marker61714
52.371cM Marker52308
52.490cM Marker62561
52.803cM Marker70638
52.972cM Marker3010
52.972cM Marker72918
52.972cM Marker56625
52.972cM Marker27539
52.972cM Marker45821
52.972cM Marker31198
52.972cM Marker75854
52.972cM Marker64570
52.972cM Marker37061
52.972cM Marker28586
52.972cM Marker67416
52.972cM Marker74661
53.972cM Marker69926

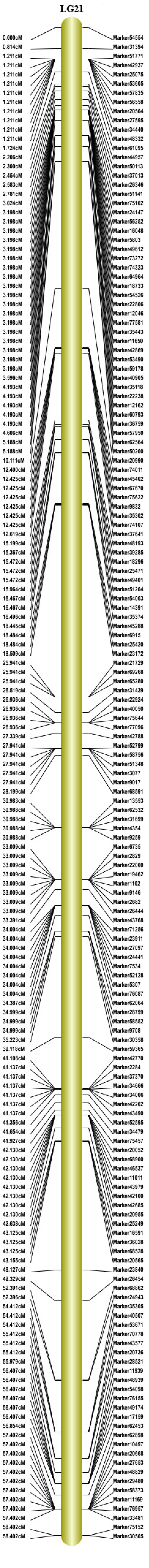
LG21
0.000cM Marker54554
0.814cM Marker31394
1.211cM Marker51771
1.211cM Marker42937
1.211cM Marker25075
1.211cM Marker53605
1.211cM Marker57835
1.211cM Marker56558
1.211cM Marker20504
1.211cM Marker27595
1.211cM Marker34440
1.211cM Marker48332
1.724cM Marker61095
2.206cM Marker44957
2.300cM Marker50113
2.454cM Marker37013
2.583cM Marker26346
2.781cM Marker51141
3.024cM Marker75102
3.198cM Marker24147
3.198cM Marker56252
3.198cM Marker16048
3.198cM Marker5803
3.198cM Marker49612
3.198cM Marker73272
3.198cM Marker74323
3.198cM Marker64964
3.198cM Marker18733
3.198cM Marker54526
3.198cM Marker22806
3.198cM Marker12046
3.198cM Marker77581
3.198cM Marker35443
3.198cM Marker11650
3.198cM Marker42869
3.198cM Marker53490
3.198cM Marker59178
3.596cM Marker40905
4.193cM Marker35118
4.193cM Marker22238
4.193cM Marker12162
4.193cM Marker60793
4.193cM Marker36759
4.606cM Marker57950
5.188cM Marker62564
5.188cM Marker50200
10.111cM Marker20990
12.400cM Marker74011
12.425cM Marker45402
12.425cM Marker67670
12.425cM Marker75622
12.425cM Marker9832
12.425cM Marker35302
12.425cM Marker74107
12.619cM Marker37641
15.199cM Marker48193
15.367cM Marker39285
15.472cM Marker18296
15.472cM Marker25471
15.472cM Marker49401
15.964cM Marker51204
16.467cM Marker54003
16.467cM Marker14391
16.496cM Marker35374
18.445cM Marker45288
18.484cM Marker6915
18.484cM Marker25420
18.509cM Marker23172
25.941cM Marker21729
25.941cM Marker69268
25.941cM Marker65280
26.519cM Marker31439
26.936cM Marker22924
26.936cM Marker40050
26.936cM Marker75644
26.936cM Marker77096
27.339cM Marker42788
27.941cM Marker52799
27.941cM Marker58756
27.941cM Marker51348
27.941cM Marker3077
27.941cM Marker9017
28.199cM Marker68591
30.983cM Marker13553
30.988cM Marker62532
30.988cM Marker31699
30.988cM Marker4354
30.988cM Marker9259
33.009cM Marker6735
33.009cM Marker2829
33.009cM Marker22000
33.009cM Marker19462
33.009cM Marker1102
33.009cM Marker9146
33.009cM Marker2682
33.009cM Marker26444
33.391cM Marker43768
34.004cM Marker71256
34.004cM Marker23911
34.004cM Marker27097
34.004cM Marker24441
34.004cM Marker7534
34.004cM Marker52128
34.004cM Marker5307
34.004cM Marker76087
34.387cM Marker62064
34.999cM Marker28799
34.999cM Marker58552
34.999cM Marker9708
35.223cM Marker30358
39.118cM Marker59365
41.108cM Marker42770
41.137cM Marker2284
41.137cM Marker37370
41.137cM Marker34666
41.137cM Marker34006
41.137cM Marker42202
41.137cM Marker43490
41.356cM Marker52595
41.654cM Marker34479
41.927cM Marker75457
42.130cM Marker20052
42.130cM Marker68900
42.130cM Marker46537
42.130cM Marker11011
42.130cM Marker43979
42.130cM Marker42100
42.130cM Marker42685
42.130cM Marker20955
42.638cM Marker25249
43.125cM Marker16591
43.125cM Marker36028
43.125cM Marker68528
43.155cM Marker20565
48.127cM Marker23840
49.329cM Marker26454
52.391cM Marker68862
52.396cM Marker24943
54.412cM Marker35305
54.412cM Marker40507
54.412cM Marker53671
55.412cM Marker70778
55.412cM Marker43577
55.412cM Marker20736
55.979cM Marker28521
56.407cM Marker11939
56.407cM Marker48939
56.407cM Marker54098
56.407cM Marker76155
56.407cM Marker49174
56.407cM Marker17159
56.854cM Marker62453
57.402cM Marker62898
57.402cM Marker10497
57.402cM Marker20668
57.402cM Marker27653
57.402cM Marker48829
57.402cM Marker29480
57.402cM Marker58373
57.402cM Marker11169
57.402cM Marker76957
57.402cM Marker33481
58.402cM Marker75152
58.402cM Marker30505

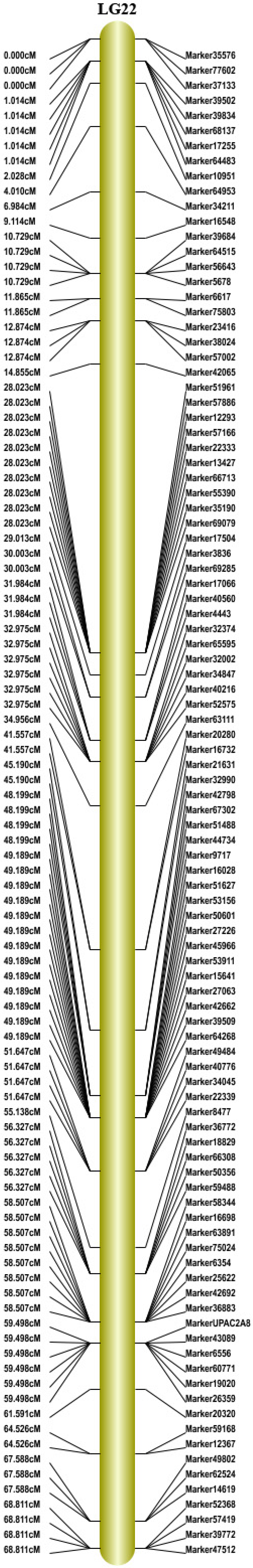
LG22
0.000cM Marker35576
0.000cM Marker77602
0.000cM Marker37133
1.014cM Marker39502
1.014cM Marker39834
1.014cM Marker68137
1.014cM Marker17255
1.014cM Marker64483
2.028cM Marker10951
4.010cM Marker64953
6.984cM Marker34211
9.114cM Marker16548
10.729cM Marker39684
10.729cM Marker64515
10.729cM Marker56643
10.729cM Marker5678
11.865cM Marker6617
11.865cM Marker75803
12.874cM Marker23416
12.874cM Marker38024
12.874cM Marker57002
14.855cM Marker42065
28.023cM Marker51961
28.023cM Marker57886
28.023cM Marker12293
28.023cM Marker57166
28.023cM Marker22333
28.023cM Marker13427
28.023cM Marker66713
28.023cM Marker55390
28.023cM Marker35190
28.023cM Marker69079
29.013cM Marker17504
30.003cM Marker3836
30.003cM Marker69285
31.984cM Marker17066
31.984cM Marker40560
31.984cM Marker4443
32.975cM Marker32374
32.975cM Marker65595
32.975cM Marker32002
32.975cM Marker34847
32.975cM Marker40216
32.975cM Marker52575
34.956cM Marker63111
41.557cM Marker20280
41.557cM Marker16732
45.190cM Marker21631
45.190cM Marker32990
48.199cM Marker42798
48.199cM Marker67302
48.199cM Marker51488
48.199cM Marker44734
49.189cM Marker9717
49.189cM Marker16028
49.189cM Marker51627
49.189cM Marker53156
49.189cM Marker50601
49.189cM Marker27226
49.189cM Marker45966
49.189cM Marker53911
49.189cM Marker15641
49.189cM Marker27063
49.189cM Marker42662
49.189cM Marker39509
49.189cM Marker64268
51.647cM Marker49484
51.647cM Marker40776
51.647cM Marker34045
51.647cM Marker22339
55.138cM Marker8477
56.327cM Marker36772
56.327cM Marker18829
56.327cM Marker66308
56.327cM Marker50356
56.327cM Marker59488
58.507cM Marker58344
58.507cM Marker16698
58.507cM Marker63891
58.507cM Marker75024
58.507cM Marker6354
58.507cM Marker25622
58.507cM Marker42692
58.507cM Marker36883
59.498cM MarkerUPAC2A8
59.498cM Marker43089
59.498cM Marker6556
59.498cM Marker60771
59.498cM Marker19020
59.498cM Marker26359
61.591cM Marker20320
64.526cM Marker59168
64.526cM Marker12367
67.588cM Marker49802
67.588cM Marker62524
67.588cM Marker14619
68.811cM Marker52368
68.811cM Marker57419
68.811cM Marker39772
68.811cM Marker47512

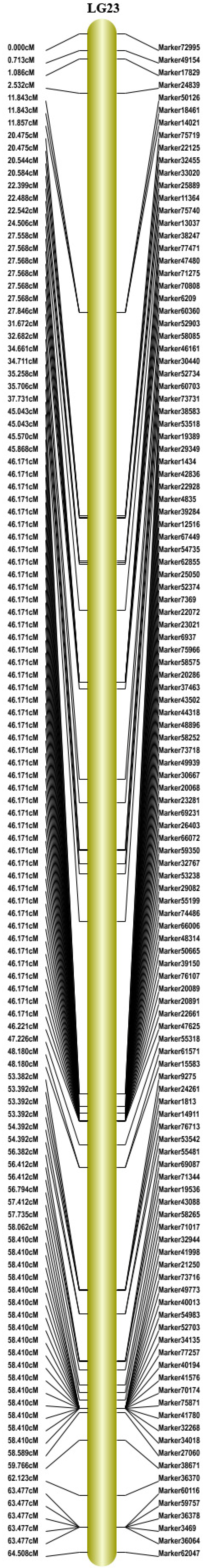
LG23
0.000cM Marker72995
0.713cM Marker49154
1.086cM Marker17829
2.532cM Marker24839
11.843cM Marker50126
11.843cM Marker18461
11.857cM Marker14021
20.475cM Marker75719
20.475cM Marker22125
20.544cM Marker32455
20.584cM Marker33020
22.399cM Marker25889
22.488cM Marker11364
22.542cM Marker75740
24.506cM Marker13037
27.558cM Marker38247
27.568cM Marker77471
27.568cM Marker47480
27.568cM Marker71275
27.568cM Marker70808
27.568cM Marker6209
27.846cM Marker60360
31.672cM Marker52903
32.682cM Marker58085
34.661cM Marker46161
34.711cM Marker30440
35.258cM Marker52734
35.706cM Marker60703
37.731cM Marker73731
45.043cM Marker38583
45.043cM Marker53518
45.570cM Marker19389
45.868cM Marker29349
46.171cM Marker1434
46.171cM Marker42836
46.171cM Marker22928
46.171cM Marker4835
46.171cM Marker39284
46.171cM Marker12516
46.171cM Marker67449
46.171cM Marker54735
46.171cM Marker62855
46.171cM Marker25050
46.171cM Marker52374
46.171cM Marker7369
46.171cM Marker22072
46.171cM Marker23021
46.171cM Marker6937
46.171cM Marker75966
46.171cM Marker58575
46.171cM Marker20286
46.171cM Marker37463
46.171cM Marker43502
46.171cM Marker44318
46.171cM Marker48896
46.171cM Marker58252
46.171cM Marker73718
46.171cM Marker49939
46.171cM Marker30667
46.171cM Marker20068
46.171cM Marker23281
46.171cM Marker69231
46.171cM Marker26403
46.171cM Marker66072
46.171cM Marker59350
46.171cM Marker32767
46.171cM Marker53238
46.171cM Marker29082
46.171cM Marker55199
46.171cM Marker74486
46.171cM Marker66006
46.171cM Marker48314
46.171cM Marker50665
46.171cM Marker39150
46.171cM Marker76107
46.171cM Marker20089
46.171cM Marker20891
46.171cM Marker22661
46.221cM Marker47625
47.226cM Marker55318
48.180cM Marker61571
48.180cM Marker15583
53.382cM Marker9275
53.392cM Marker24261
53.392cM Marker1813
53.392cM Marker14911
54.392cM Marker76713
54.392cM Marker53542
56.382cM Marker55481
56.412cM Marker69087
56.412cM Marker71344
56.794cM Marker19536
57.412cM Marker43088
57.735cM Marker58265
58.062cM Marker71017
58.410cM Marker32944
58.410cM Marker41998
58.410cM Marker21250
58.410cM Marker73716
58.410cM Marker49773
58.410cM Marker40013
58.410cM Marker54983
58.410cM Marker52703
58.410cM Marker34135
58.410cM Marker77257
58.410cM Marker40194
58.410cM Marker41576
58.410cM Marker70174
58.410cM Marker75871
58.410cM Marker41780
58.410cM Marker32268
58.410cM Marker34018
58.589cM Marker27060
59.766cM Marker38671
62.123cM Marker36370
63.477cM Marker60116
63.477cM Marker59757
63.477cM Marker36378
63.477cM Marker3469
63.477cM Marker36064
64.508cM Marker62047

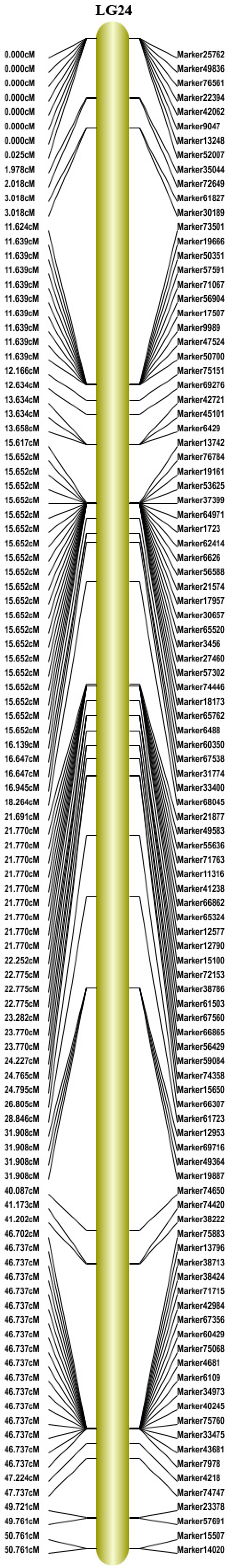
LG24
0.000cM Marker25762
0.000cM Marker49836
0.000cM Marker76561
0.000cM Marker22394
0.000cM Marker42062
0.000cM Marker9047
0.000cM Marker13248
0.025cM Marker52007
1.978cM Marker35044
2.018cM Marker72649
3.018cM Marker61827
3.018cM Marker30189
11.624cM Marker73501
11.639cM Marker19666
11.639cM Marker50351
11.639cM Marker57591
11.639cM Marker71067
11.639cM Marker56904
11.639cM Marker17507
11.639cM Marker9989
11.639cM Marker47524
11.639cM Marker50700
12.166cM Marker75151
12.634cM Marker69276
13.634cM Marker42721
13.634cM Marker45101
13.658cM Marker6429
15.617cM Marker13742
15.652cM Marker76784
15.652cM Marker19161
15.652cM Marker53625
15.652cM Marker37399
15.652cM Marker64971
15.652cM Marker1723
15.652cM Marker62414
15.652cM Marker6626
15.652cM Marker56588
15.652cM Marker21574
15.652cM Marker17957
15.652cM Marker30657
15.652cM Marker65520
15.652cM Marker3456
15.652cM Marker27460
15.652cM Marker57302
15.652cM Marker74446
15.652cM Marker18173
15.652cM Marker65762
15.652cM Marker6488
16.139cM Marker60350
16.647cM Marker67538
16.647cM Marker31774
16.945cM Marker33400
18.264cM Marker68045
21.691cM Marker21877
21.770cM Marker49583
21.770cM Marker55636
21.770cM Marker71763
21.770cM Marker11316
21.770cM Marker41238
21.770cM Marker66862
21.770cM Marker65324
21.770cM Marker12577
21.770cM Marker12790
22.252cM Marker15100
22.775cM Marker72153
22.775cM Marker38786
22.775cM Marker61503
23.282cM Marker67560
23.770cM Marker66865
23.770cM Marker56429
24.227cM Marker59084
24.765cM Marker74358
24.795cM Marker15650
26.805cM Marker66307
28.846cM Marker61723
31.908cM Marker12953
31.908cM Marker69716
31.908cM Marker49364
31.908cM Marker19887
40.087cM Marker74650
41.173cM Marker74420
41.202cM Marker38222
46.702cM Marker75883
46.737cM Marker13796
46.737cM Marker38713
46.737cM Marker38424
46.737cM Marker71715
46.737cM Marker42984
46.737cM Marker67356
46.737cM Marker60429
46.737cM Marker75068
46.737cM Marker4681
46.737cM Marker6109
46.737cM Marker34973
46.737cM Marker40245
46.737cM Marker75760
46.737cM Marker33475
46.737cM Marker43681
46.737cM Marker7978
47.224cM Marker4218
47.737cM Marker74747
49.721cM Marker23378
49.761cM Marker57691
50.761cM Marker15507
50.761cM Marker14020

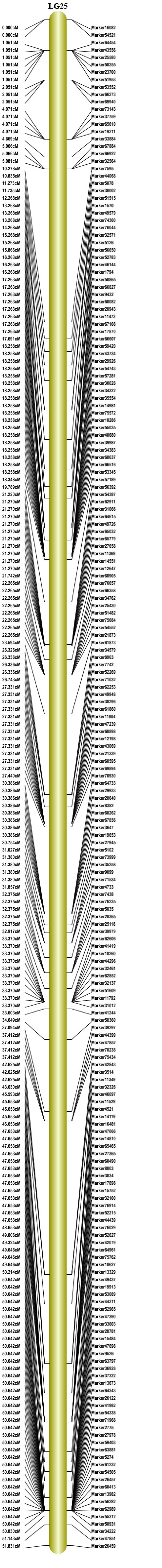
LG25
0.000cM Marker16082
0.000cM Marker54521
1.051cM Marker64454
1.051cM Marker43556
1.051cM Marker25580
1.051cM Marker58255
1.051cM Marker23700
1.051cM Marker51953
2.051cM Marker53552
2.051cM Marker66273
2.051cM Marker69940
4.071cM Marker73143
4.071cM Marker37759
4.071cM Marker65610
4.071cM Marker19211
4.669cM Marker33884
5.066cM Marker67884
5.066cM Marker66922
5.081cM Marker32564
10.278cM Marker7595
10.835cM Marker44068
11.273cM Marker5078
11.735cM Marker38002
12.268cM Marker51515
13.268cM Marker1570
13.268cM Marker49579
13.268cM Marker74300
14.268cM Marker76044
15.268cM Marker32571
15.268cM Marker5126
15.866cM Marker56650
16.263cM Marker52783
16.263cM Marker46144
16.263cM Marker1794
17.263cM Marker50865
17.263cM Marker66827
17.263cM Marker9432
17.263cM Marker60082
17.263cM Marker20943
17.263cM Marker11473
17.263cM Marker67108
17.263cM Marker17870
17.691cM Marker66607
18.258cM Marker59420
18.258cM Marker43734
18.258cM Marker29926
18.258cM Marker54743
18.258cM Marker57281
18.258cM Marker30028
18.258cM Marker34322
18.258cM Marker35554
18.258cM Marker14981
18.258cM Marker75572
18.258cM Marker10286
18.258cM Marker55035
18.258cM Marker40680
18.258cM Marker39987
18.258cM Marker34383
18.258cM Marker68637
18.258cM Marker66516
18.258cM Marker53345
18.348cM Marker57189
19.789cM Marker56392
21.220cM Marker54387
21.270cM Marker62911
21.270cM Marker31066
21.270cM Marker64615
21.270cM Marker49726
21.270cM Marker65032
21.270cM Marker65779
21.270cM Marker27658
21.270cM Marker11369
21.270cM Marker14551
21.270cM Marker12647
21.742cM Marker68905
22.265cM Marker76657
22.265cM Marker66358
22.265cM Marker34762
22.265cM Marker25430
22.265cM Marker51482
22.265cM Marker75684
22.265cM Marker54552
22.265cM Marker21873
23.594cM Marker61873
26.326cM Marker34579
26.336cM Marker8963
26.336cM Marker7742
26.336cM Marker52269
26.743cM Marker71032
27.331cM Marker62253
27.331cM Marker49948
27.331cM Marker38296
27.331cM Marker61860
27.331cM Marker11804
27.331cM Marker47239
27.331cM Marker68898
27.331cM Marker12198
27.331cM Marker43069
27.331cM Marker21339
27.331cM Marker60595
27.331cM Marker69894
27.440cM Marker70930
30.386cM Marker64733
30.386cM Marker29933
30.386cM Marker20640
30.386cM Marker8382
30.386cM Marker60262
30.386cM Marker67856
30.386cM Marker3647
30.386cM Marker19653
30.754cM Marker27945
31.027cM Marker5102
31.380cM Marker73999
31.380cM Marker35258
31.380cM Marker9099
31.380cM Marker71534
31.857cM Marker4733
32.375cM Marker7438
32.375cM Marker76235
32.375cM Marker5035
32.375cM Marker28365
32.375cM Marker25118
32.917cM Marker39979
33.370cM Marker62606
33.370cM Marker41419
33.370cM Marker10260
33.370cM Marker44296
33.370cM Marker32461
33.370cM Marker62852
33.370cM Marker32137
33.370cM Marker51609
33.370cM Marker11792
33.370cM Marker31012
33.603cM Marker41244
34.649cM Marker58360
37.094cM Marker39297
37.412cM Marker44399
37.412cM Marker47852
37.412cM Marker70238
37.412cM Marker75434
42.625cM Marker42843
42.625cM Marker3514
42.625cM Marker11349
43.630cM Marker32326
45.593cM Marker46097
45.653cM Marker11529
45.653cM Marker4521
45.653cM Marker14119
46.653cM Marker16481
47.653cM Marker47066
47.653cM Marker14810
47.653cM Marker65465
47.653cM Marker27365
47.653cM Marker60490
47.653cM Marker8803
47.653cM Marker3834
47.653cM Marker17898
47.653cM Marker15752
47.653cM Marker32100
47.653cM Marker76914
47.653cM Marker52215
47.653cM Marker44439
48.653cM Marker76029
49.006cM Marker52627
49.324cM Marker42079
49.646cM Marker64961
49.646cM Marker75762
49.646cM Marker18627
50.214cM Marker13329
50.642cM Marker49437
50.642cM Marker19913
50.642cM Marker53089
50.642cM Marker44311
50.642cM Marker52965
50.642cM Marker47390
50.642cM Marker33603
50.642cM Marker28781
50.642cM Marker15484
50.642cM Marker47698
50.642cM Marker9526
50.642cM Marker63797
50.642cM Marker36928
50.642cM Marker37322
50.642cM Marker13673
50.642cM Marker64343
50.642cM Marker26122
50.642cM Marker41982
50.642cM Marker54338
50.642cM Marker71968
50.642cM Marker2775
50.642cM Marker27978
50.642cM Marker59403
50.642cM Marker63881
50.642cM Marker5274
50.642cM Marker61232
50.642cM Marker54505
50.642cM Marker26457
50.642cM Marker60413
50.642cM Marker13982
50.642cM Marker56282
50.642cM Marker62989
50.642cM Marker55312
50.642cM Marker50931
50.830cM Marker34222
51.143cM Marker47851
51.831cM Marker26459

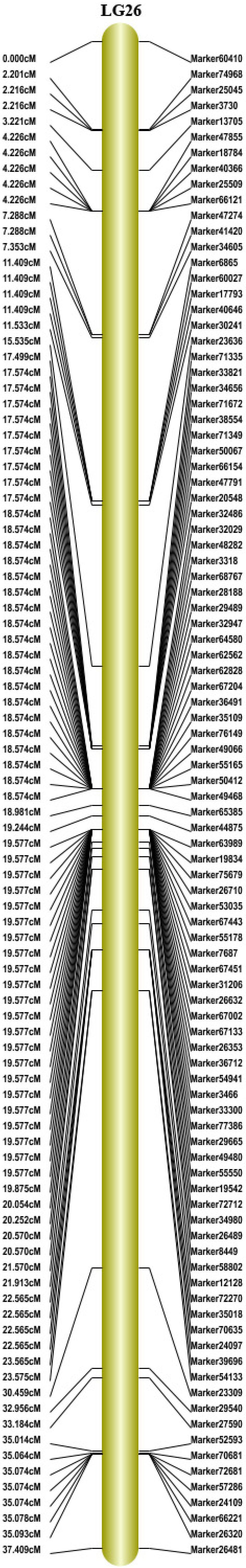
LG26
0.000cM Marker60410
2.201cM Marker74968
2.216cM Marker25045
2.216cM Marker3730
3.221cM Marker13705
4.226cM Marker47855
4.226cM Marker18784
4.226cM Marker40366
4.226cM Marker25509
4.226cM Marker66121
7.288cM Marker47274
7.288cM Marker41420
7.353cM Marker34605
11.409cM Marker6865
11.409cM Marker60027
11.409cM Marker17793
11.409cM Marker40646
11.533cM Marker30241
15.535cM Marker23636
17.499cM Marker71335
17.574cM Marker33821
17.574cM Marker34656
17.574cM Marker71672
17.574cM Marker38554
17.574cM Marker71349
17.574cM Marker50067
17.574cM Marker66154
17.574cM Marker47791
17.574cM Marker20548
18.574cM Marker32486
18.574cM Marker32029
18.574cM Marker48282
18.574cM Marker3318
18.574cM Marker68767
18.574cM Marker28188
18.574cM Marker29489
18.574cM Marker32947
18.574cM Marker64580
18.574cM Marker62562
18.574cM Marker62828
18.574cM Marker67204
18.574cM Marker36491
18.574cM Marker35109
18.574cM Marker76149
18.574cM Marker49066
18.574cM Marker55165
18.574cM Marker50412
18.574cM Marker49468
18.981cM Marker65385
19.244cM Marker44875
19.577cM Marker63989
19.577cM Marker19834
19.577cM Marker75679
19.577cM Marker26710
19.577cM Marker53035
19.577cM Marker67443
19.577cM Marker55178
19.577cM Marker7687
19.577cM Marker67451
19.577cM Marker31206
19.577cM Marker26632
19.577cM Marker67002
19.577cM Marker67133
19.577cM Marker26353
19.577cM Marker36712
19.577cM Marker54941
19.577cM Marker3466
19.577cM Marker33300
19.577cM Marker77386
19.577cM Marker29665
19.577cM Marker49480
19.577cM Marker55550
19.875cM Marker19542
20.054cM Marker72712
20.252cM Marker34980
20.570cM Marker26489
20.570cM Marker8449
21.570cM Marker58802
21.913cM Marker12128
22.565cM Marker72270
22.565cM Marker35018
22.565cM Marker70635
22.565cM Marker24097
23.565cM Marker39696
23.575cM Marker54133
30.459cM Marker23309
32.956cM Marker29540
33.184cM Marker27590
35.014cM Marker52593
35.064cM Marker70681
35.074cM Marker72681
35.074cM Marker57286
35.074cM Marker24109
35.078cM Marker66221
35.093cM Marker26320
37.409cM Marker26481

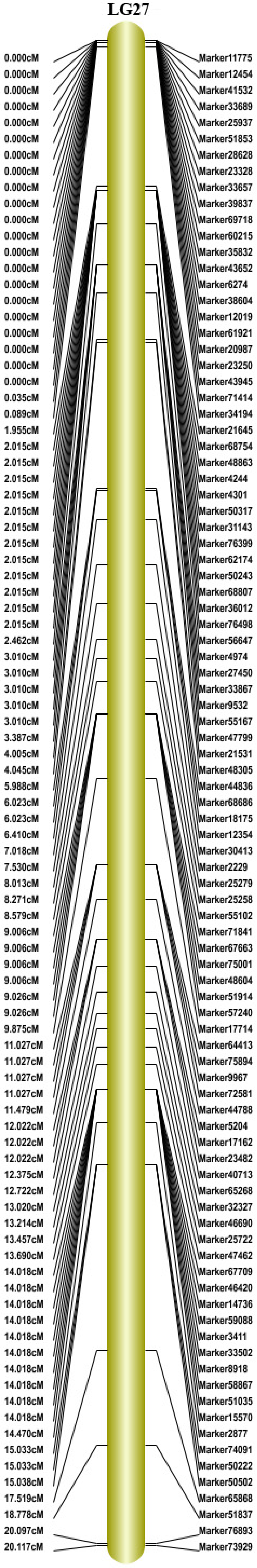
LG27
0.000cM Marker11775
0.000cM Marker12454
0.000cM Marker41532
0.000cM Marker33689
0.000cM Marker25937
0.000cM Marker51853
0.000cM Marker28628
0.000cM Marker23328
0.000cM Marker33657
0.000cM Marker39837
0.000cM Marker69718
0.000cM Marker60215
0.000cM Marker35832
0.000cM Marker43652
0.000cM Marker6274
0.000cM Marker38604
0.000cM Marker12019
0.000cM Marker61921
0.000cM Marker20987
0.000cM Marker23250
0.000cM Marker43945
0.035cM Marker71414
0.089cM Marker34194
1.955cM Marker21645
2.015cM Marker68754
2.015cM Marker48863
2.015cM Marker4244
2.015cM Marker4301
2.015cM Marker50317
2.015cM Marker31143
2.015cM Marker76399
2.015cM Marker62174
2.015cM Marker50243
2.015cM Marker68807
2.015cM Marker36012
2.015cM Marker76498
2.462cM Marker56647
3.010cM Marker4974
3.010cM Marker27450
3.010cM Marker33867
3.010cM Marker9532
3.010cM Marker55167
3.387cM Marker47799
4.005cM Marker21531
4.045cM Marker48305
5.988cM Marker44836
6.023cM Marker68686
6.023cM Marker18175
6.410cM Marker12354
7.018cM Marker30413
7.530cM Marker2229
8.013cM Marker25279
8.271cM Marker25258
8.579cM Marker55102
9.006cM Marker71841
9.006cM Marker67663
9.006cM Marker75001
9.006cM Marker48604
9.026cM Marker51914
9.026cM Marker57240
9.875cM Marker17714
11.027cM Marker64413
11.027cM Marker75894
11.027cM Marker9967
11.027cM Marker72581
11.479cM Marker44788
12.022cM Marker5204
12.022cM Marker17162
12.022cM Marker23482
12.375cM Marker40713
12.722cM Marker65268
13.020cM Marker32327
13.214cM Marker46690
13.457cM Marker25722
13.690cM Marker47462
14.018cM Marker67709
14.018cM Marker46420
14.018cM Marker14736
14.018cM Marker59088
14.018cM Marker3411
14.018cM Marker33502
14.018cM Marker8918
14.018cM Marker58867
14.018cM Marker51035
14.018cM Marker15570
14.470cM Marker2877
15.033cM Marker74091
15.033cM Marker50222
15.038cM Marker50502
17.519cM Marker65868
18.778cM Marker51837
20.097cM Marker76893
20.117cM Marker73929

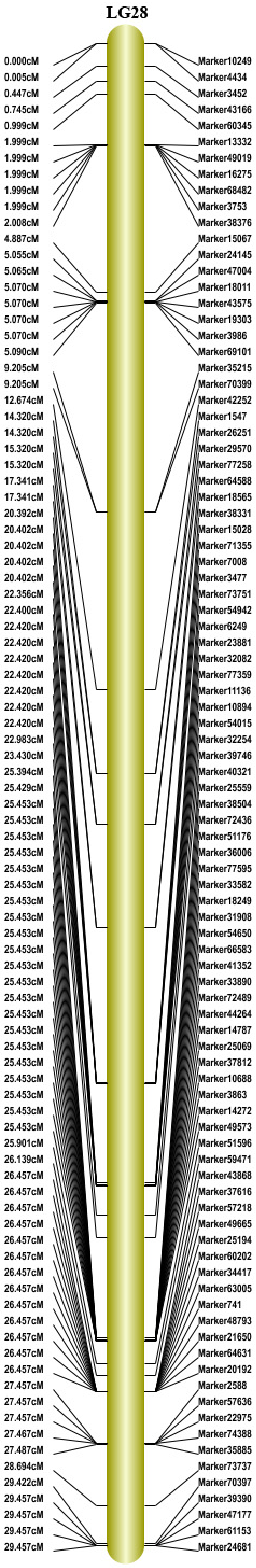
LG28
0.000cM Marker10249
0.005cM Marker4434
0.447cM Marker3452
0.745cM Marker43166
0.999cM Marker60345
1.999cM Marker13332
1.999cM Marker49019
1.999cM Marker16275
1.999cM Marker68482
1.999cM Marker3753
2.008cM Marker38376
4.887cM Marker15067
5.055cM Marker24145
5.065cM Marker47004
5.070cM Marker18011
5.070cM Marker43575
5.070cM Marker19303
5.070cM Marker3986
5.090cM Marker69101
9.205cM Marker35215
9.205cM Marker70399
12.674cM Marker42252
14.320cM Marker1547
14.320cM Marker26251
15.320cM Marker29570
15.320cM Marker77258
17.341cM Marker64588
17.341cM Marker18565
20.392cM Marker38331
20.402cM Marker15028
20.402cM Marker71355
20.402cM Marker7008
20.402cM Marker3477
22.356cM Marker73751
22.400cM Marker54942
22.420cM Marker6249
22.420cM Marker23881
22.420cM Marker32082
22.420cM Marker77359
22.420cM Marker11136
22.420cM Marker10894
22.420cM Marker54015
22.983cM Marker32254
23.430cM Marker39746
25.394cM Marker40321
25.429cM Marker25559
25.453cM Marker38504
25.453cM Marker72436
25.453cM Marker51176
25.453cM Marker36006
25.453cM Marker77595
25.453cM Marker33582
25.453cM Marker18249
25.453cM Marker31908
25.453cM Marker54650
25.453cM Marker66583
25.453cM Marker41352
25.453cM Marker33890
25.453cM Marker72489
25.453cM Marker44264
25.453cM Marker14787
25.453cM Marker25069
25.453cM Marker37812
25.453cM Marker10688
25.453cM Marker3863
25.453cM Marker14272
25.453cM Marker49573
25.901cM Marker51596
26.139cM Marker59471
26.457cM Marker43868
26.457cM Marker37616
26.457cM Marker57218
26.457cM Marker49665
26.457cM Marker25194
26.457cM Marker60202
26.457cM Marker34417
26.457cM Marker63005
26.457cM Marker741
26.457cM Marker48793
26.457cM Marker21650
26.457cM Marker64631
26.457cM Marker20192
27.457cM Marker2588
27.457cM Marker57636
27.457cM Marker22975
27.467cM Marker74388
27.487cM Marker35885
28.694cM Marker73737
29.422cM Marker70397
29.457cM Marker39390
29.457cM Marker47177
29.457cM Marker61153
29.457cM Marker24681

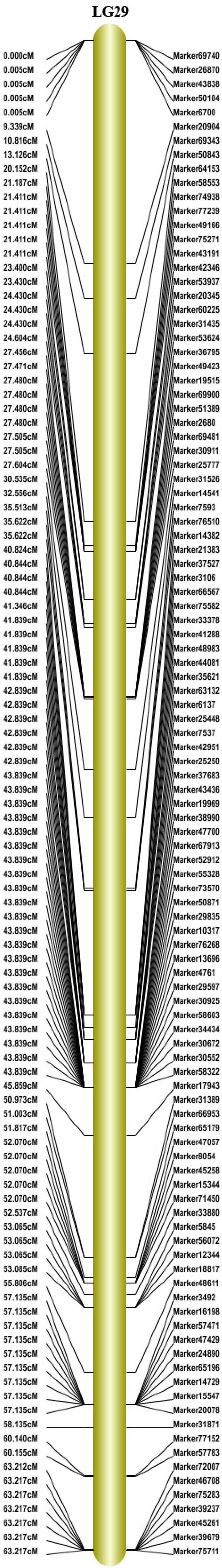
LG29
0.000cM Marker69740
0.005cM Marker26870
0.005cM Marker43838
0.005cM Marker50104
0.005cM Marker6700
9.339cM Marker20904
10.816cM Marker69343
13.126cM Marker50843
20.152cM Marker64153
21.187cM Marker58553
21.411cM Marker74938
21.411cM Marker77239
21.411cM Marker49166
21.411cM Marker75271
21.411cM Marker43191
23.400cM Marker42346
23.430cM Marker53937
24.430cM Marker20345
24.430cM Marker60225
24.430cM Marker31435
24.604cM Marker53624
27.456cM Marker36795
27.471cM Marker49423
27.480cM Marker19515
27.480cM Marker69900
27.480cM Marker51389
27.480cM Marker2680
27.505cM Marker69481
27.505cM Marker30911
27.604cM Marker25777
30.535cM Marker31526
32.556cM Marker14541
35.513cM Marker7593
35.622cM Marker76510
35.622cM Marker14382
40.824cM Marker21383
40.844cM Marker37527
40.844cM Marker3106
40.844cM Marker66567
41.346cM Marker75582
41.839cM Marker33378
41.839cM Marker41288
41.839cM Marker48983
41.839cM Marker44081
41.839cM Marker35621
42.839cM Marker63132
42.839cM Marker6137
42.839cM Marker25448
42.839cM Marker7537
42.839cM Marker42951
42.839cM Marker25250
43.839cM Marker37683
43.839cM Marker43436
43.839cM Marker19969
43.839cM Marker38990
43.839cM Marker47700
43.839cM Marker67913
43.839cM Marker52912
43.839cM Marker55328
43.839cM Marker73570
43.839cM Marker50871
43.839cM Marker29835
43.839cM Marker10317
43.839cM Marker76268
43.839cM Marker13696
43.839cM Marker4761
43.839cM Marker29597
43.839cM Marker30925
43.839cM Marker58603
43.839cM Marker34434
43.839cM Marker30672
43.839cM Marker30552
43.839cM Marker58322
45.859cM Marker17943
50.973cM Marker31389
51.003cM Marker66953
51.817cM Marker65179
52.070cM Marker47057
52.070cM Marker8054
52.070cM Marker45258
52.070cM Marker15344
52.070cM Marker71450
52.537cM Marker33880
53.065cM Marker5845
53.065cM Marker56072
53.065cM Marker12344
53.085cM Marker18817
55.806cM Marker48611
57.135cM Marker3492
57.135cM Marker16198
57.135cM Marker57471
57.135cM Marker47429
57.135cM Marker24890
57.135cM Marker65196
57.135cM Marker14729
57.135cM Marker15547
57.135cM Marker20078
58.135cM Marker31871
60.140cM Marker77152
60.155cM Marker57783
63.212cM Marker72007
63.217cM Marker46708
63.217cM Marker75283
63.217cM Marker39237
63.217cM Marker45261
63.217cM Marker39679
63.217cM Marker75711

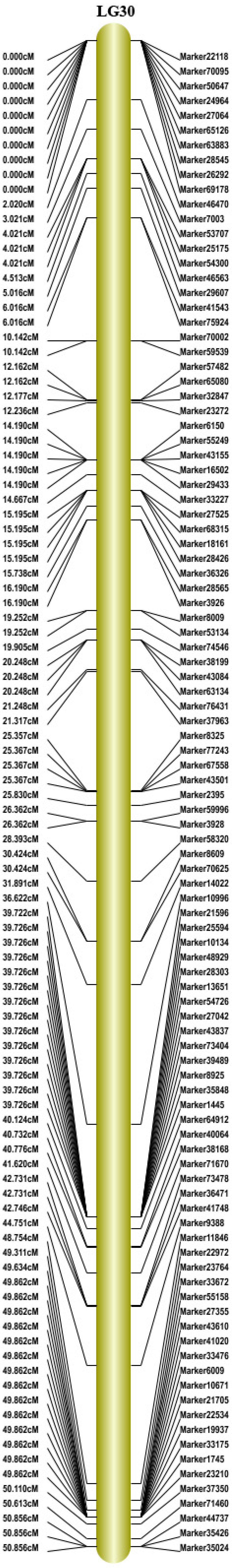
LG30
0.000cM Marker22118
0.000cM Marker70095
0.000cM Marker50647
0.000cM Marker24964
0.000cM Marker27064
0.000cM Marker65126
0.000cM Marker63883
0.000cM Marker28545
0.000cM Marker26292
0.000cM Marker69178
2.020cM Marker46470
3.021cM Marker7003
4.021cM Marker53707
4.021cM Marker25175
4.021cM Marker54300
4.513cM Marker46563
5.016cM Marker29607
6.016cM Marker41543
6.016cM Marker75924
10.142cM Marker70002
10.142cM Marker59539
12.162cM Marker57482
12.162cM Marker65080
12.177cM Marker32847
12.236cM Marker23272
14.190cM Marker6150
14.190cM Marker55249
14.190cM Marker43155
14.190cM Marker16502
14.190cM Marker29433
14.667cM Marker33227
15.195cM Marker27525
15.195cM Marker68315
15.195cM Marker18161
15.195cM Marker28426
15.738cM Marker36326
16.190cM Marker28565
16.190cM Marker3926
19.252cM Marker8009
19.252cM Marker53134
19.905cM Marker74546
20.248cM Marker38199
20.248cM Marker43084
20.248cM Marker63134
21.248cM Marker76431
21.317cM Marker37963
25.357cM Marker8325
25.367cM Marker77243
25.367cM Marker67558
25.367cM Marker43501
25.830cM Marker2395
26.362cM Marker59996
26.362cM Marker3928
28.393cM Marker58320
30.424cM Marker8609
30.424cM Marker70625
31.891cM Marker14022
36.622cM Marker10996
39.722cM Marker21596
39.726cM Marker25594
39.726cM Marker10134
39.726cM Marker48929
39.726cM Marker28303
39.726cM Marker13651
39.726cM Marker54726
39.726cM Marker27042
39.726cM Marker43837
39.726cM Marker73404
39.726cM Marker39489
39.726cM Marker8925
39.726cM Marker35848
39.726cM Marker1445
40.124cM Marker64912
40.732cM Marker40064
40.776cM Marker38168
41.620cM Marker71670
42.731cM Marker73478
42.731cM Marker36471
42.746cM Marker41748
44.751cM Marker9388
48.754cM Marker11846
49.311cM Marker22972
49.634cM Marker23764
49.862cM Marker33672
49.862cM Marker55158
49.862cM Marker27355
49.862cM Marker43610
49.862cM Marker41020
49.862cM Marker33476
49.862cM Marker6009
49.862cM Marker10671
49.862cM Marker21705
49.862cM Marker22534
49.862cM Marker19937
49.862cM Marker33175
49.862cM Marker1745
49.862cM Marker23210
50.110cM Marker37350
50.613cM Marker71460
50.856cM Marker44737
50.856cM Marker35426
50.856cM Marker35024
