# Supplementary material for: Construction of a high-density genetic map and mapping of a sex-linked locus for the brown alga Undaria pinnatifida (Phaeophyceae) based on large scale marker development by specific length amplified fragment (SLAF) sequencing
Source: BMC Genomics. 2015 Nov 5;16:902. doi: 10.1186/s12864-015-2184-y (PMC4635539; doi:10.1186/s12864-015-2184-y)

# LG1

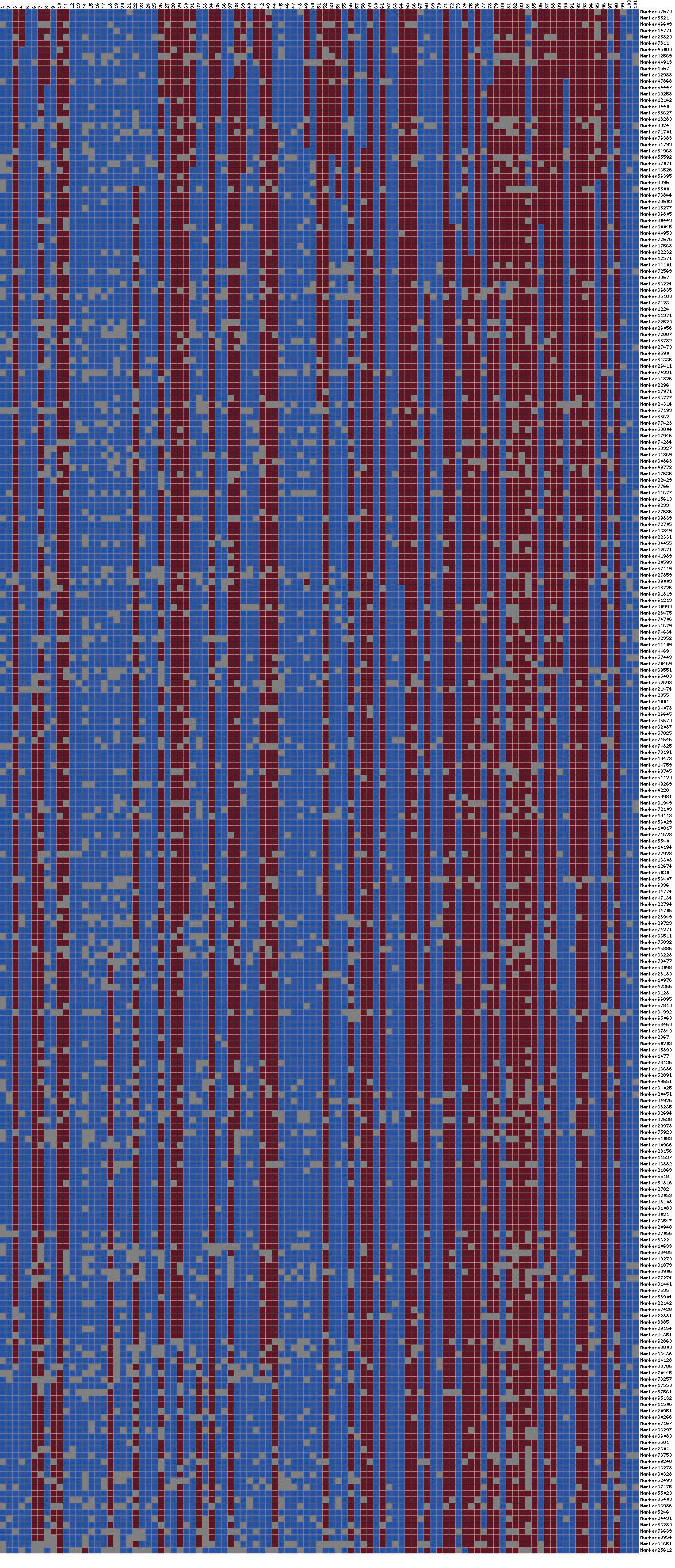

# LG2

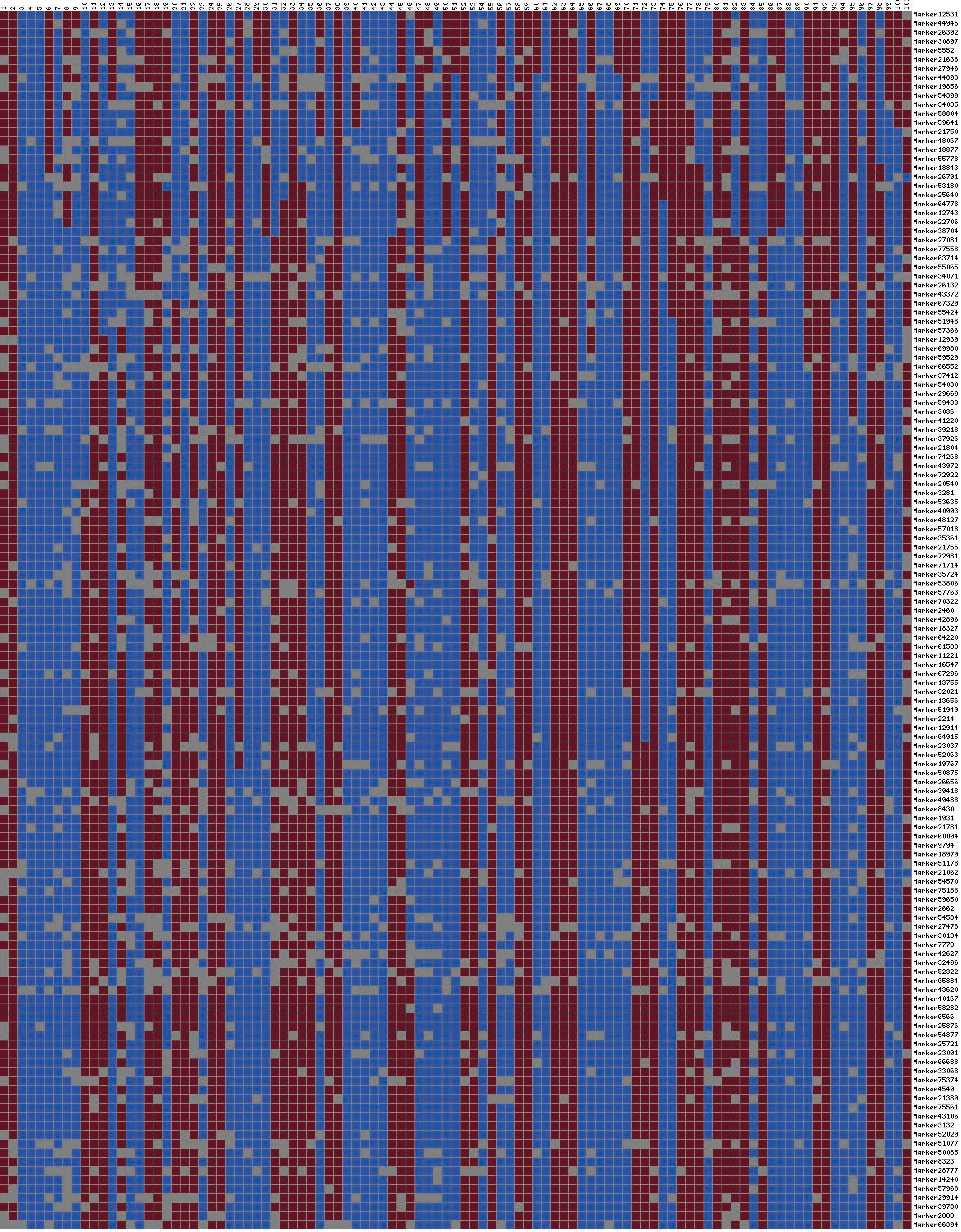

LG3

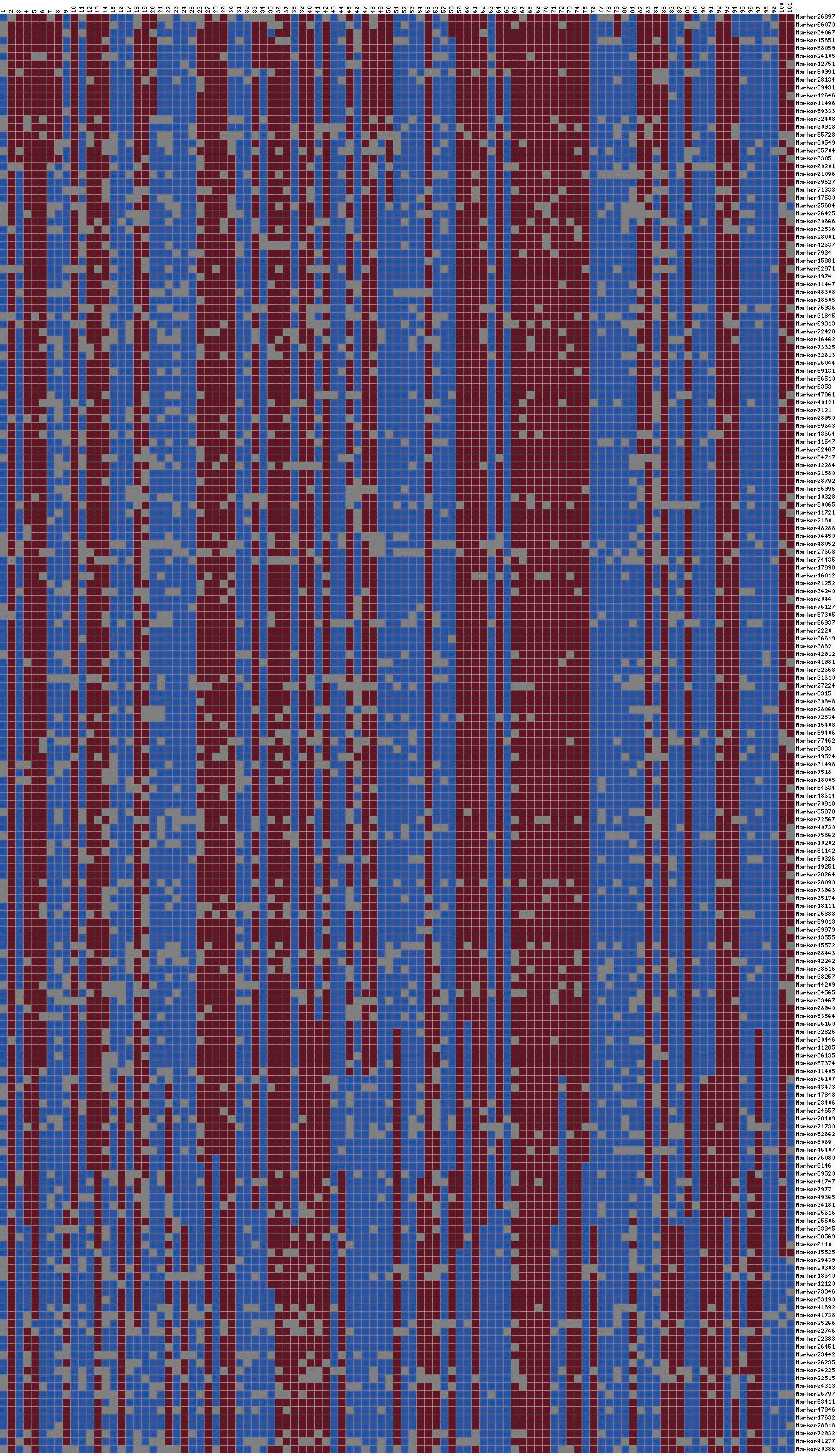

# LG4

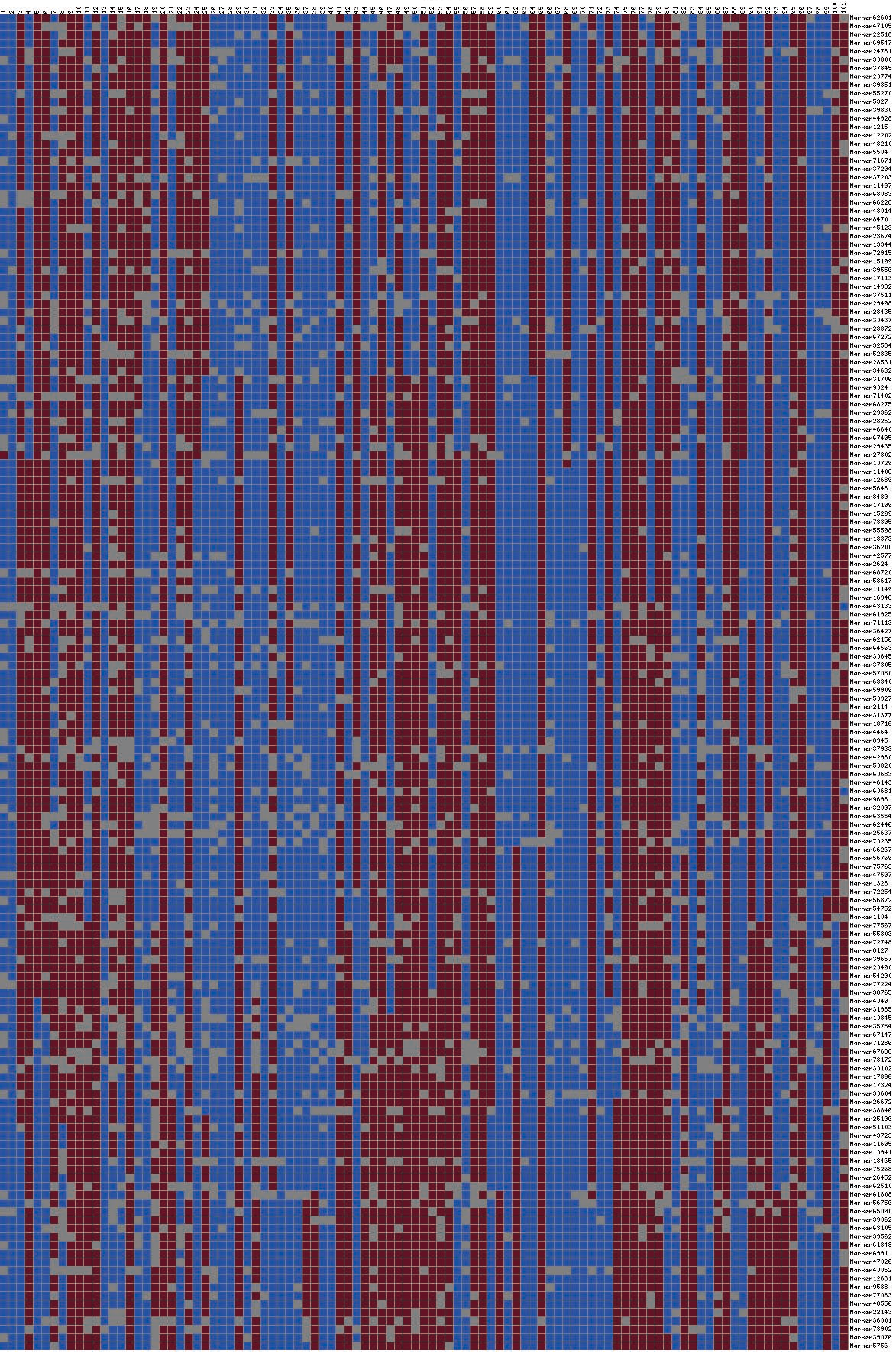

# LG5

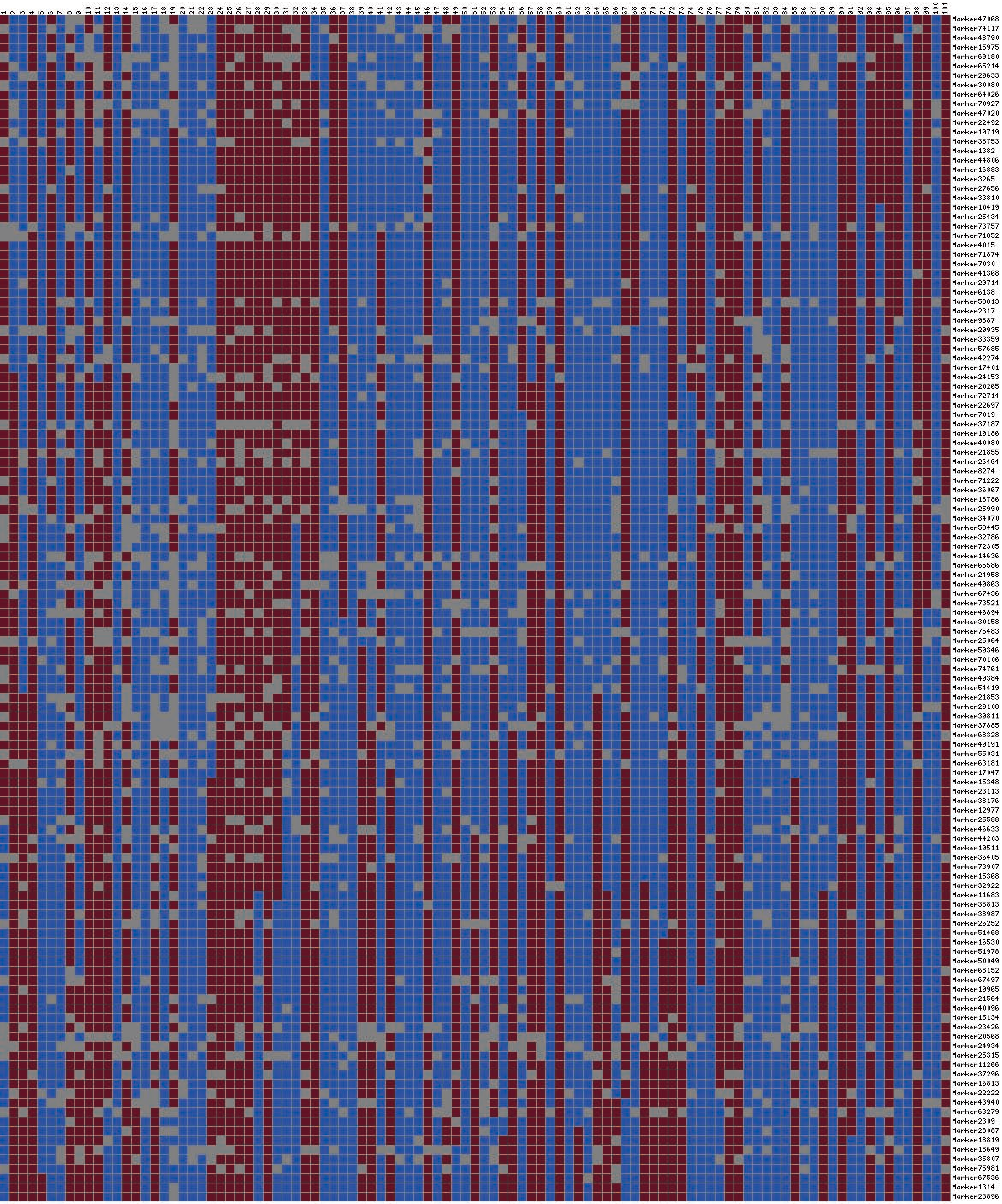

# LG6

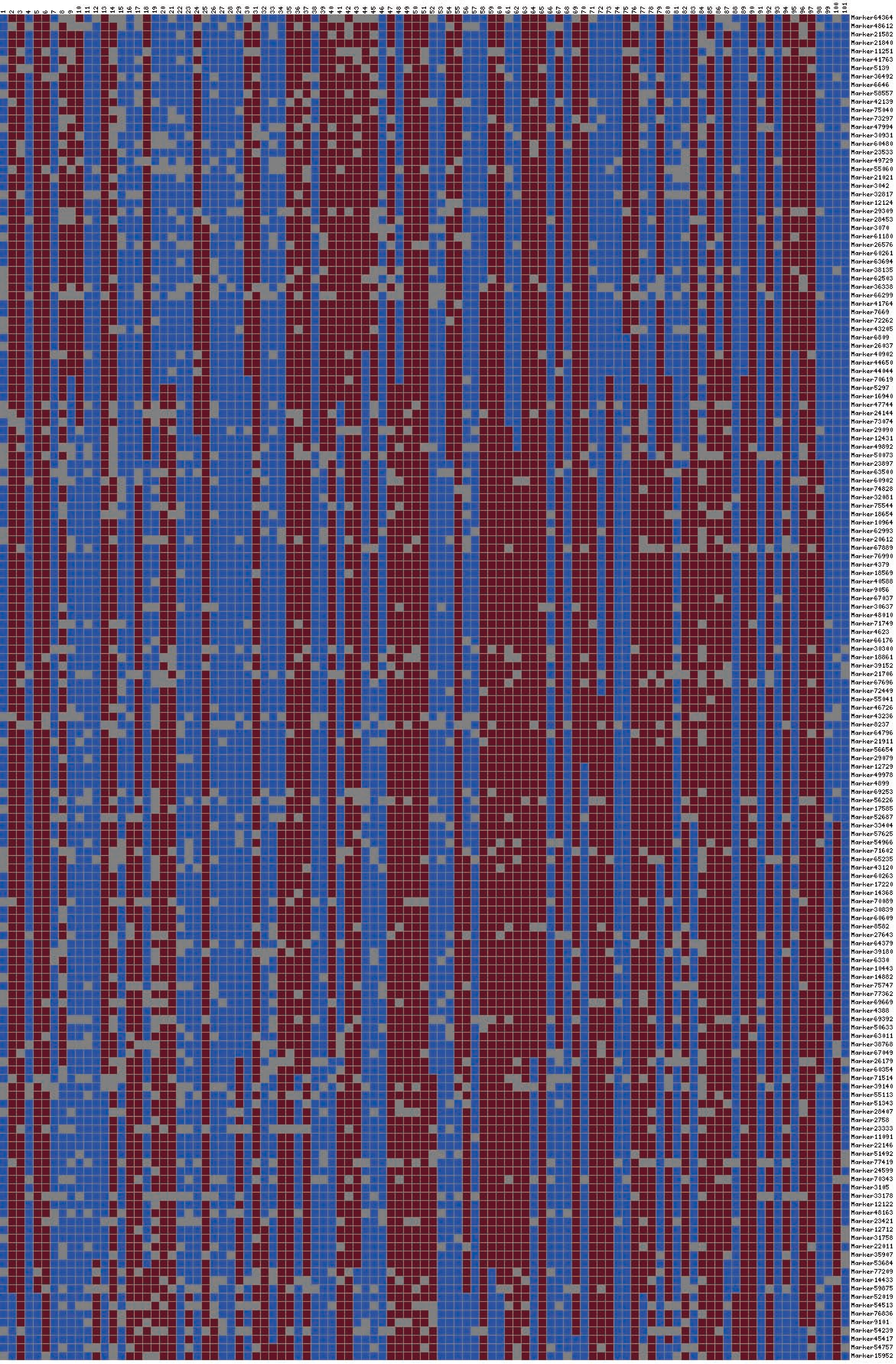

# LG7

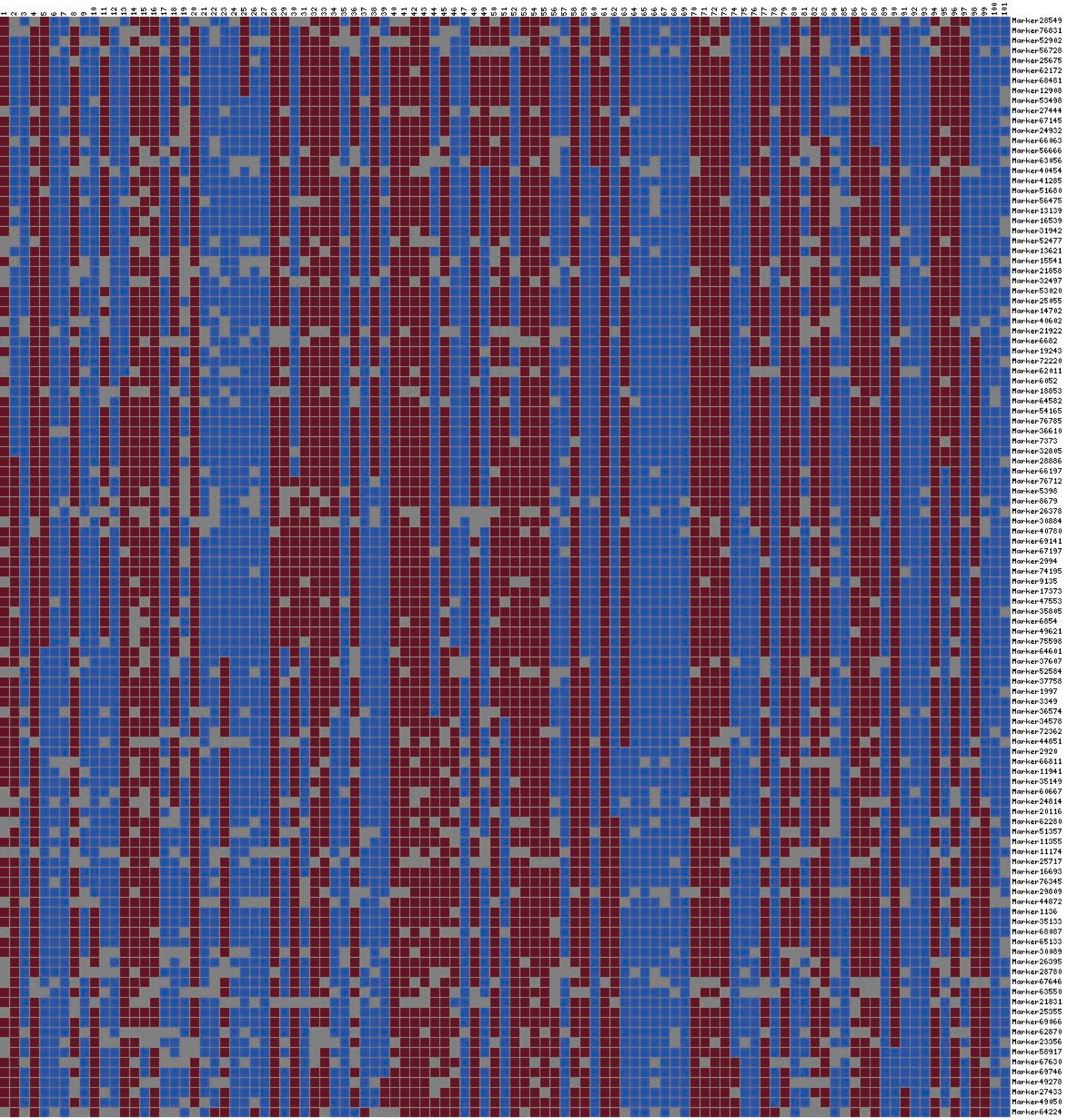

1 2 3 4 5 6 7 8 9 10 11 12 13 14 15 16 17 18 19 20 21 22 23 24 25 26 27 28 29 30 31 32 33 34 35 36 37 38 39 40 41 42 43 44 45 46 47 48 49 50 51 52 53 54 55 56 57 58 59 60 61 62 63 64 65 66 67 68 69 70 71 72 73 74 75 76 77 78 79 80 81 82 83 84 85 86 87 88 89 90 91 92 93 94 95 96 97 98 99 100 101
Marker28549
Marker76831
Marker52902
Marker56728
Marker25675
Marker62172
Marker68481
Marker12908
Marker53498
Marker27444
Marker67145
Marker24932
Marker66063
Marker56666
Marker63056
Marker40454
Marker41285
Marker51680
Marker56475
Marker13139
Marker16539
Marker31942
Marker52477
Marker13621
Marker15541
Marker21858
Marker32497
Marker53020
Marker25055
Marker14702
Marker40602
Marker21922
Marker6682
Marker19243
Marker72220
Marker62011
Marker6052
Marker18853
Marker64582
Marker54165
Marker76785
Marker36610
Marker7373
Marker32805
Marker28886
Marker66197
Marker76712
Marker5398
Marker8679
Marker26378
Marker30884
Marker40780
Marker69141
Marker67197
Marker2994
Marker74195
Marker9135
Marker17373
Marker47553
Marker35805
Marker6854
Marker49621
Marker75598
Marker64601
Marker37607
Marker52584
Marker37758
Marker1997
Marker3349
Marker36574
Marker34578
Marker72362
Marker44851
Marker2920
Marker66811
Marker11941
Marker35149
Marker60667
Marker24814
Marker20116
Marker62280
Marker51357
Marker11355
Marker11174
Marker25717
Marker16693
Marker76345
Marker29809
Marker44872
Marker1136
Marker35133
Marker68087
Marker65133
Marker30089
Marker26395
Marker28780
Marker67646
Marker63550
Marker21831
Marker25355
Marker69066
Marker62870
Marker23356
Marker58917
Marker67630
Marker69746
Marker49278
Marker27433
Marker49050
Marker64224

# LG8

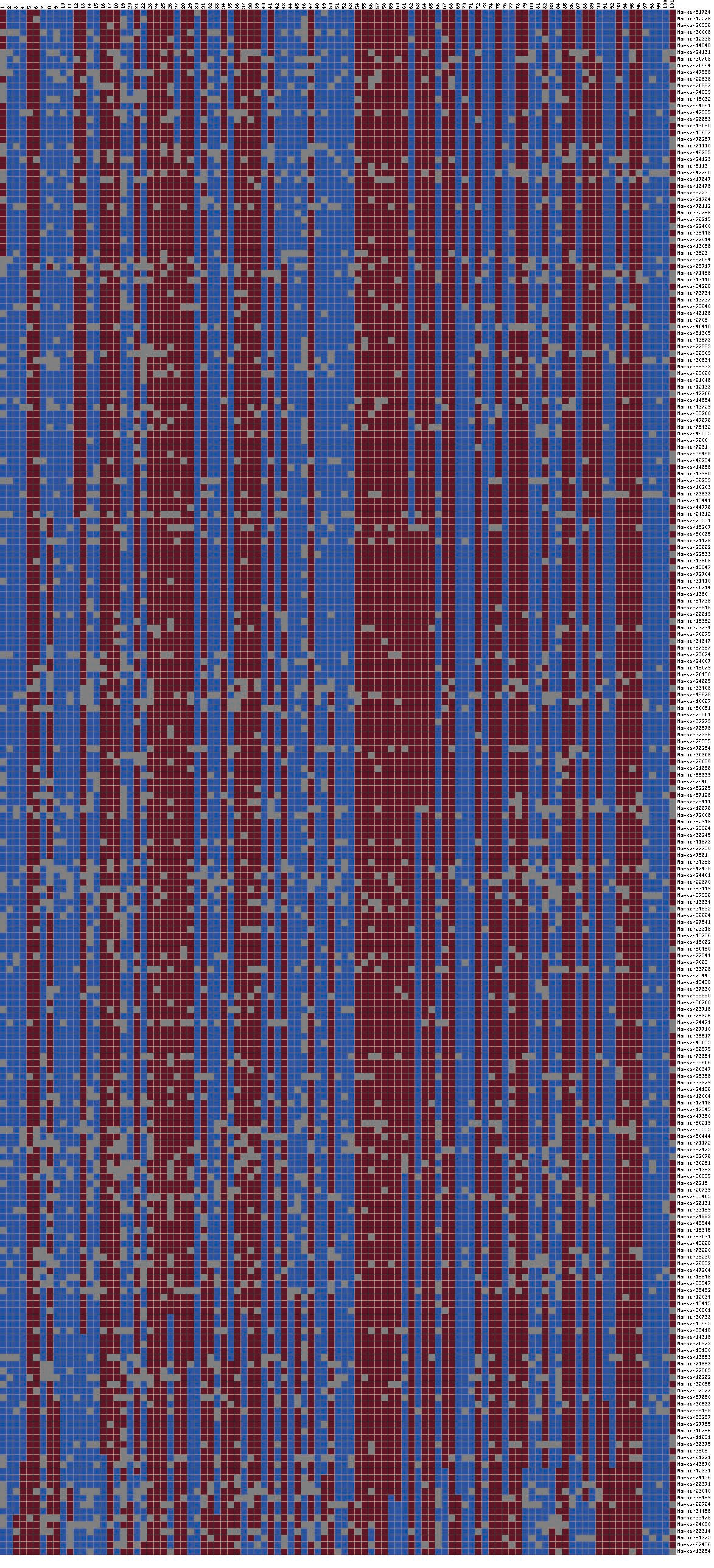

**LG9**

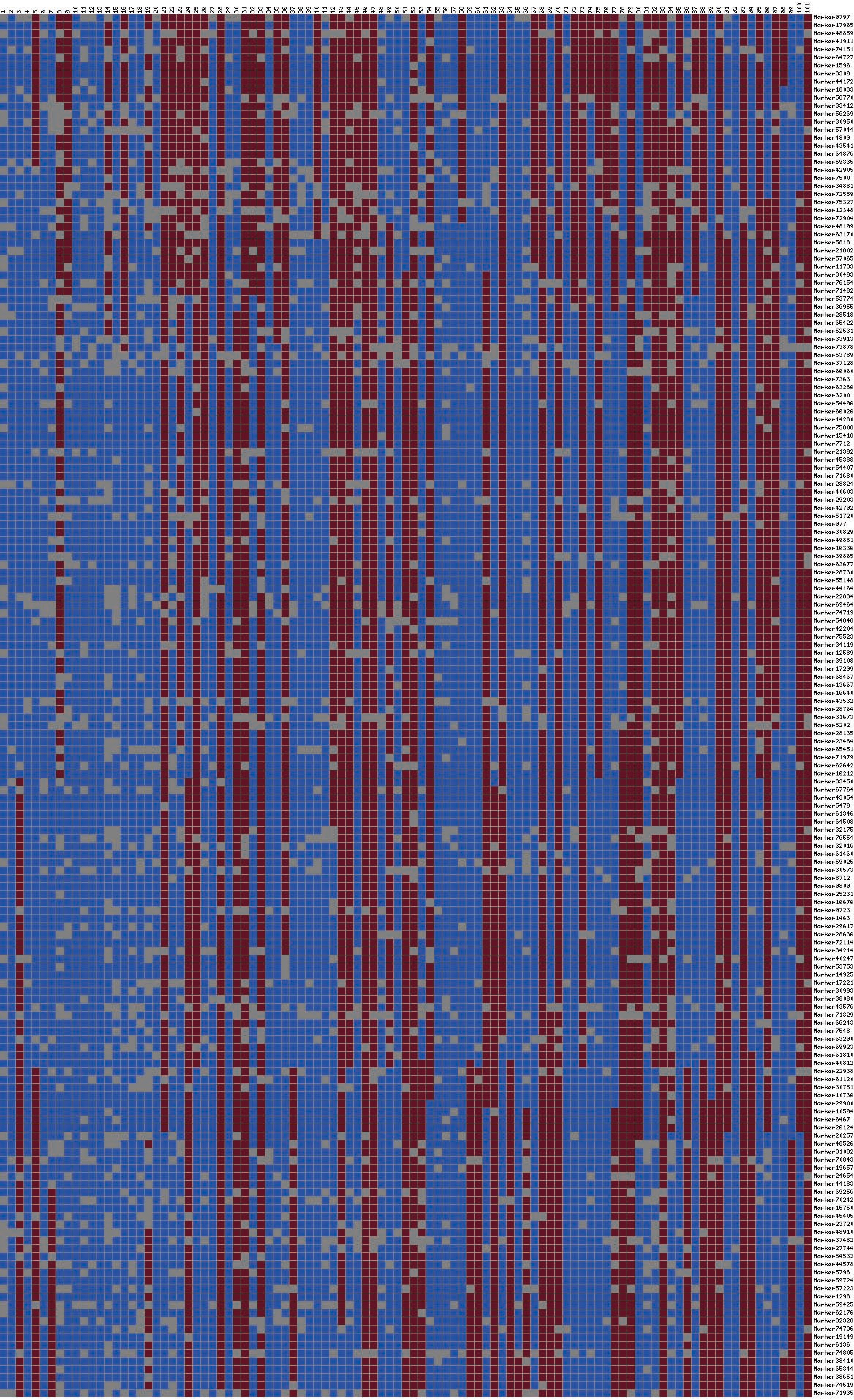

# LG10

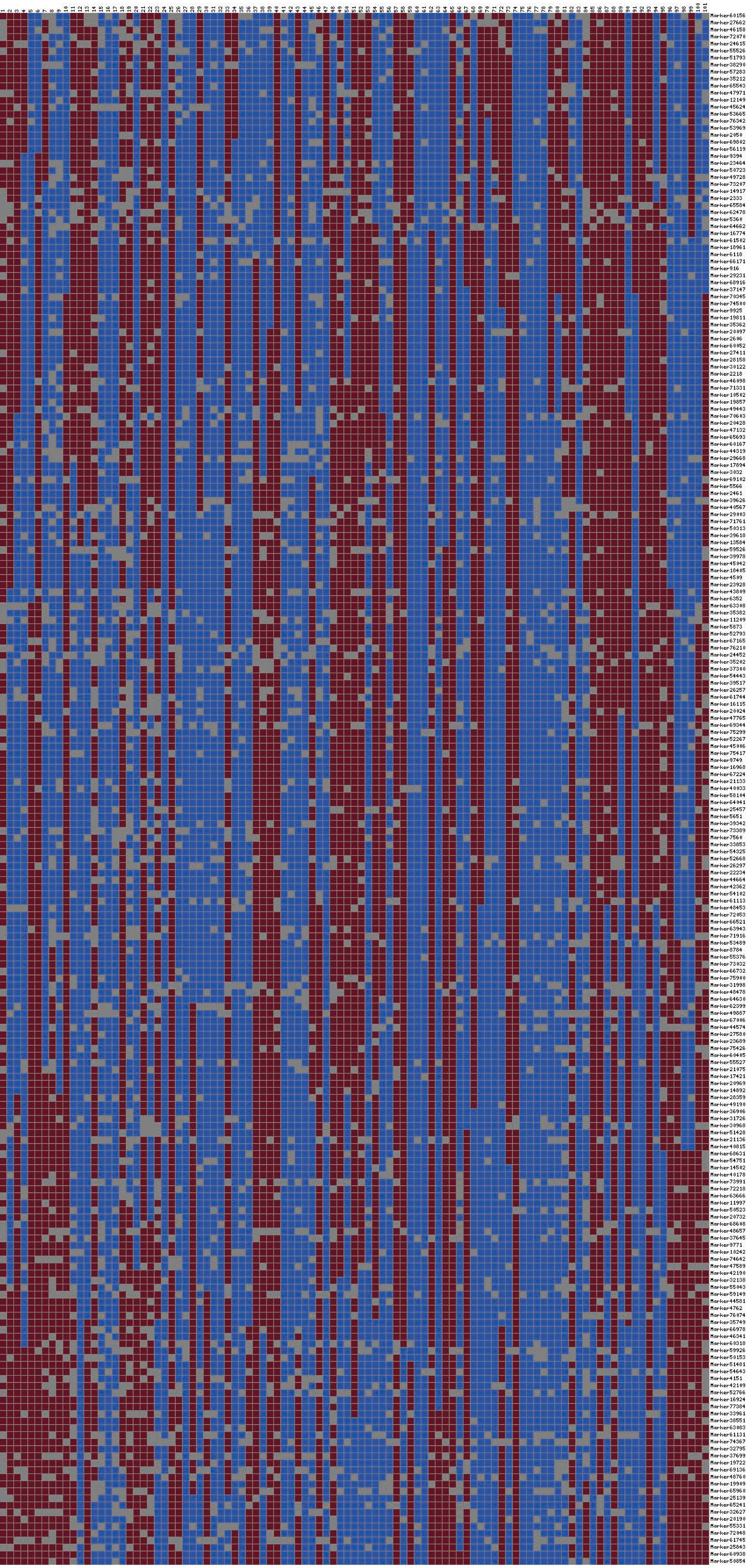

# LG11

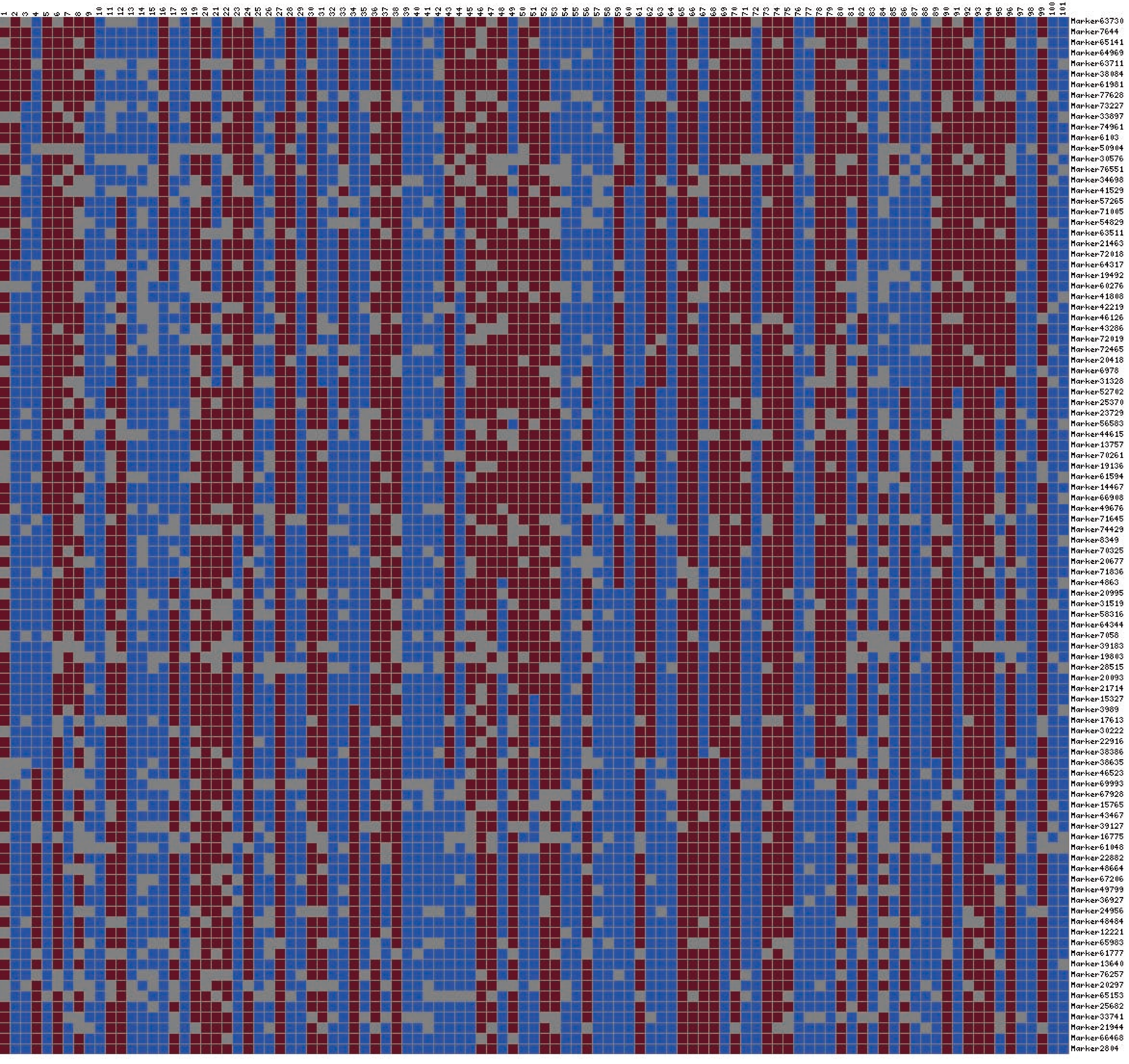

1 2 3 4 5 6 7 8 9 10 11 12 13 14 15 16 17 18 19 20 21 22 23 24 25 26 27 28 29 30 31 32 33 34 35 36 37 38 39 40 41 42 43 44 45 46 47 48 49 50 51 52 53 54 55 56 57 58 59 60 61 62 63 64 65 66 67 68 69 70 71 72 73 74 75 76 77 78 79 80 81 82 83 84 85 86 87 88 89 90 91 92 93 94 95 96 97 98 99 100 101
Marker63730
Marker7644
Marker65141
Marker64969
Marker63711
Marker38084
Marker61981
Marker77628
Marker73227
Marker33897
Marker74961
Marker6103
Marker50904
Marker30576
Marker76551
Marker34698
Marker41529
Marker57265
Marker71005
Marker54829
Marker63511
Marker21463
Marker72018
Marker64317
Marker19492
Marker60276
Marker41808
Marker42219
Marker46126
Marker43286
Marker72019
Marker72465
Marker20418
Marker6978
Marker31328
Marker52702
Marker25370
Marker23729
Marker56583
Marker44615
Marker13757
Marker70261
Marker19136
Marker61594
Marker14467
Marker66908
Marker49676
Marker71645
Marker74429
Marker8349
Marker70325
Marker20677
Marker71836
Marker4863
Marker20995
Marker31519
Marker58316
Marker64344
Marker7058
Marker39183
Marker19803
Marker28515
Marker20093
Marker21714
Marker15327
Marker3989
Marker17613
Marker30222
Marker22916
Marker38386
Marker38635
Marker46523
Marker69993
Marker67928
Marker15765
Marker43467
Marker39127
Marker16775
Marker61048
Marker22882
Marker48664
Marker67206
Marker49799
Marker36927
Marker24956
Marker48484
Marker12221
Marker65983
Marker61777
Marker13640
Marker76257
Marker20297
Marker65153
Marker25682
Marker33741
Marker21944
Marker66468
Marker2804

# LG12

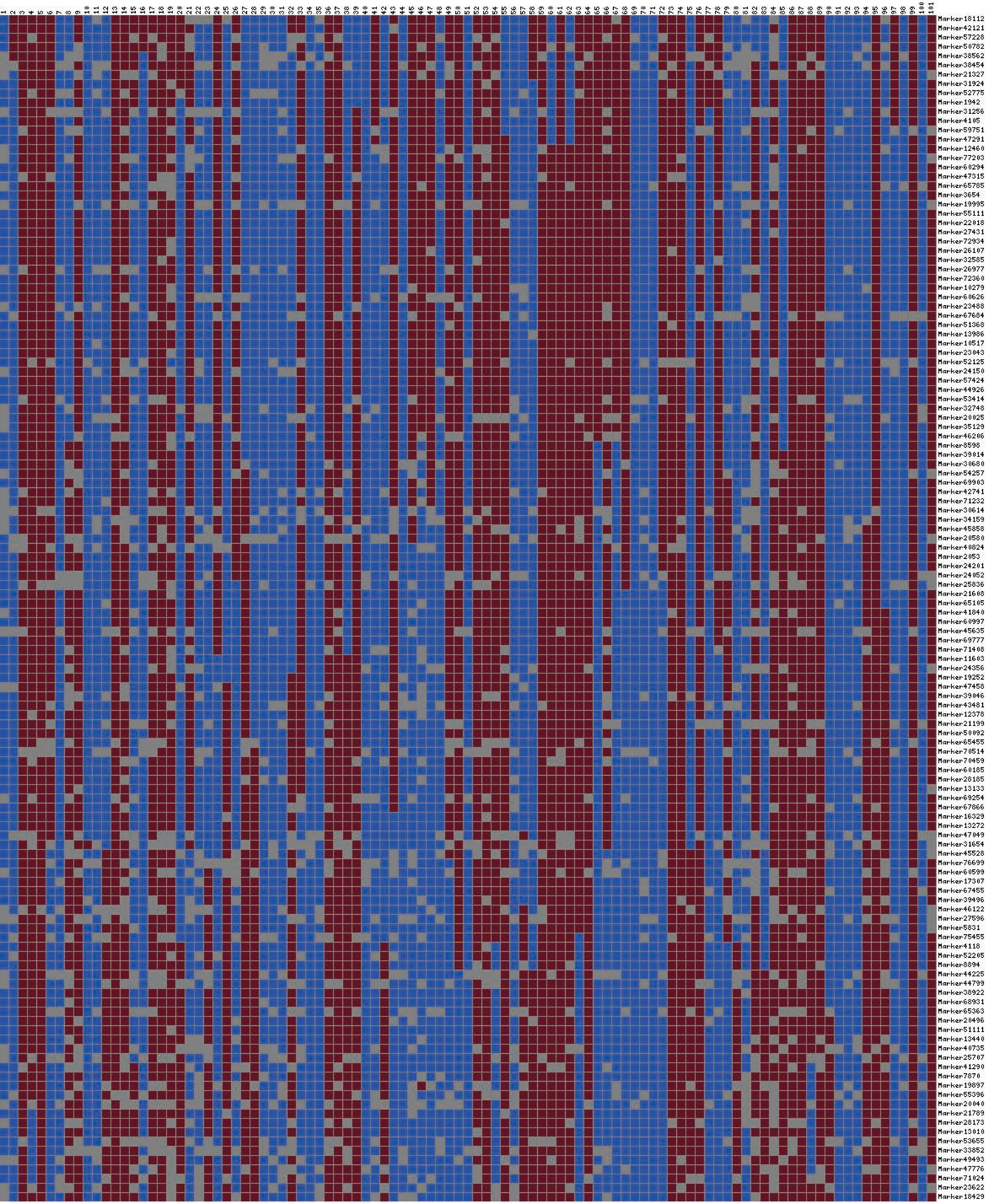
1 2 3 4 5 6 7 8 9 10 11 12 13 14 15 16 17 18 19 20 21 22 23 24 25 26 27 28 29 30 31 32 33 34 35 36 37 38 39 40 41 42 43 44 45 46 47 48 49 50 51 52 53 54 55 56 57 58 59 60 61 62 63 64 65 66 67 68 69 70 71 72 73 74 75 76 77 78 79 80 81 82 83 84 85 86 87 88 89 90 91 92 93 94 95 96 97 98 99 100 101
Marker18112
Marker42121
Marker57228
Marker50782
Marker38562
Marker38454
Marker21327
Marker31924
Marker52775
Marker1942
Marker31256
Marker4105
Marker59751
Marker47291
Marker12460
Marker77203
Marker60294
Marker47315
Marker65785
Marker3654
Marker19995
Marker55111
Marker22018
Marker27431
Marker72934
Marker26107
Marker32585
Marker26977
Marker72360
Marker10279
Marker60626
Marker23488
Marker67684
Marker51368
Marker13986
Marker10517
Marker23043
Marker52125
Marker24150
Marker57424
Marker44926
Marker53414
Marker32748
Marker20025
Marker35129
Marker46206
Marker8598
Marker39014
Marker30680
Marker54257
Marker69903
Marker42741
Marker71232
Marker30614
Marker34159
Marker45858
Marker20580
Marker40824
Marker2053
Marker24201
Marker24052
Marker25836
Marker21608
Marker65105
Marker41840
Marker60997
Marker45635
Marker69777
Marker71408
Marker11603
Marker24356
Marker19252
Marker47458
Marker39046
Marker43481
Marker12378
Marker21199
Marker50092
Marker65455
Marker70514
Marker70459
Marker60185
Marker28185
Marker13133
Marker69254
Marker67866
Marker16329
Marker13272
Marker47049
Marker31654
Marker45528
Marker76699
Marker60599
Marker17307
Marker67455
Marker39496
Marker46122
Marker27596
Marker5831
Marker75455
Marker4118
Marker52205
Marker8894
Marker44225
Marker44799
Marker38922
Marker68931
Marker65363
Marker20496
Marker51111
Marker13440
Marker40735
Marker25707
Marker41290
Marker7870
Marker19897
Marker55396
Marker20040
Marker21789
Marker28173
Marker13010
Marker53655
Marker33852
Marker49493
Marker47776
Marker71024
Marker23622
Marker18429

# LG13

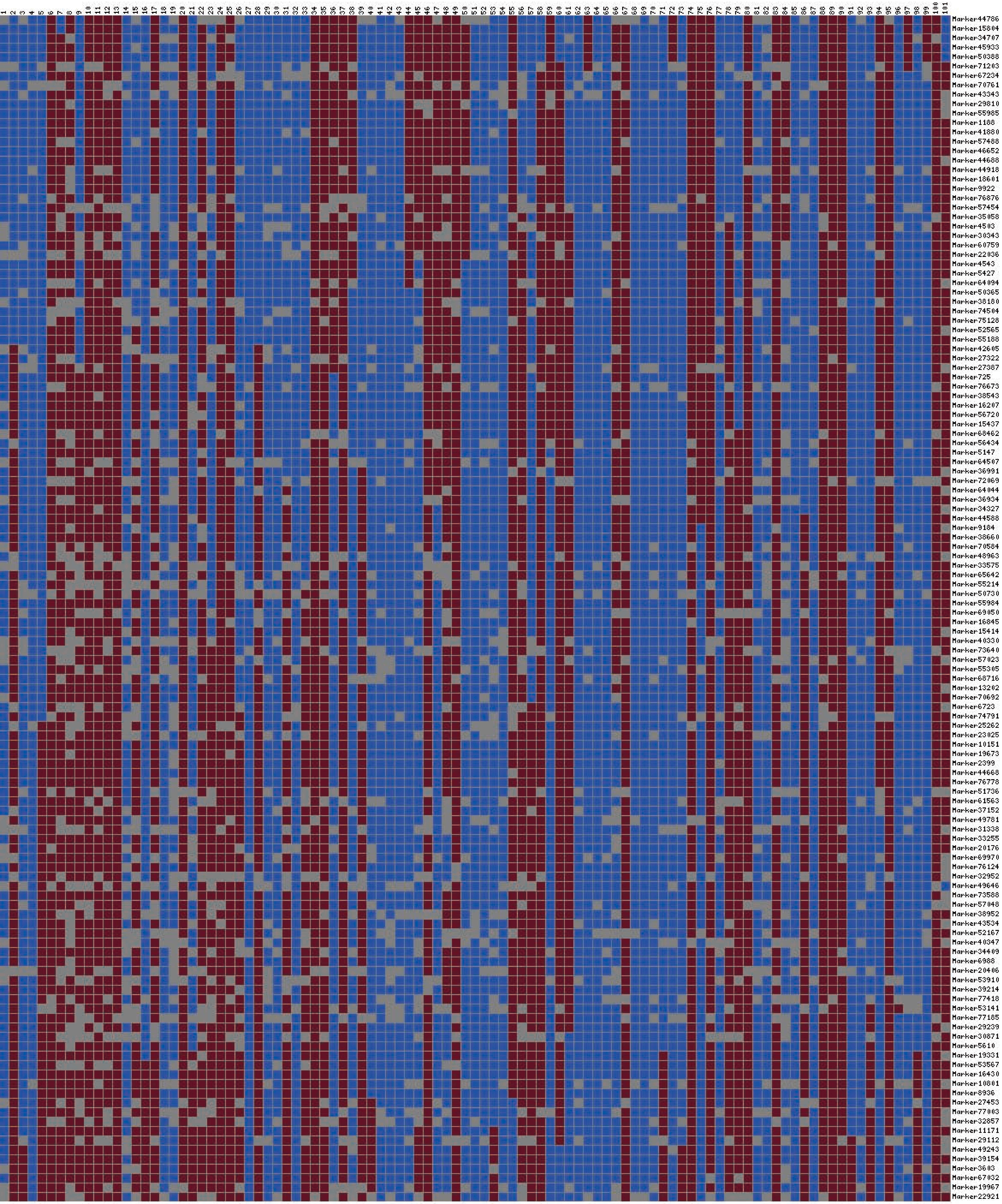

# LG14

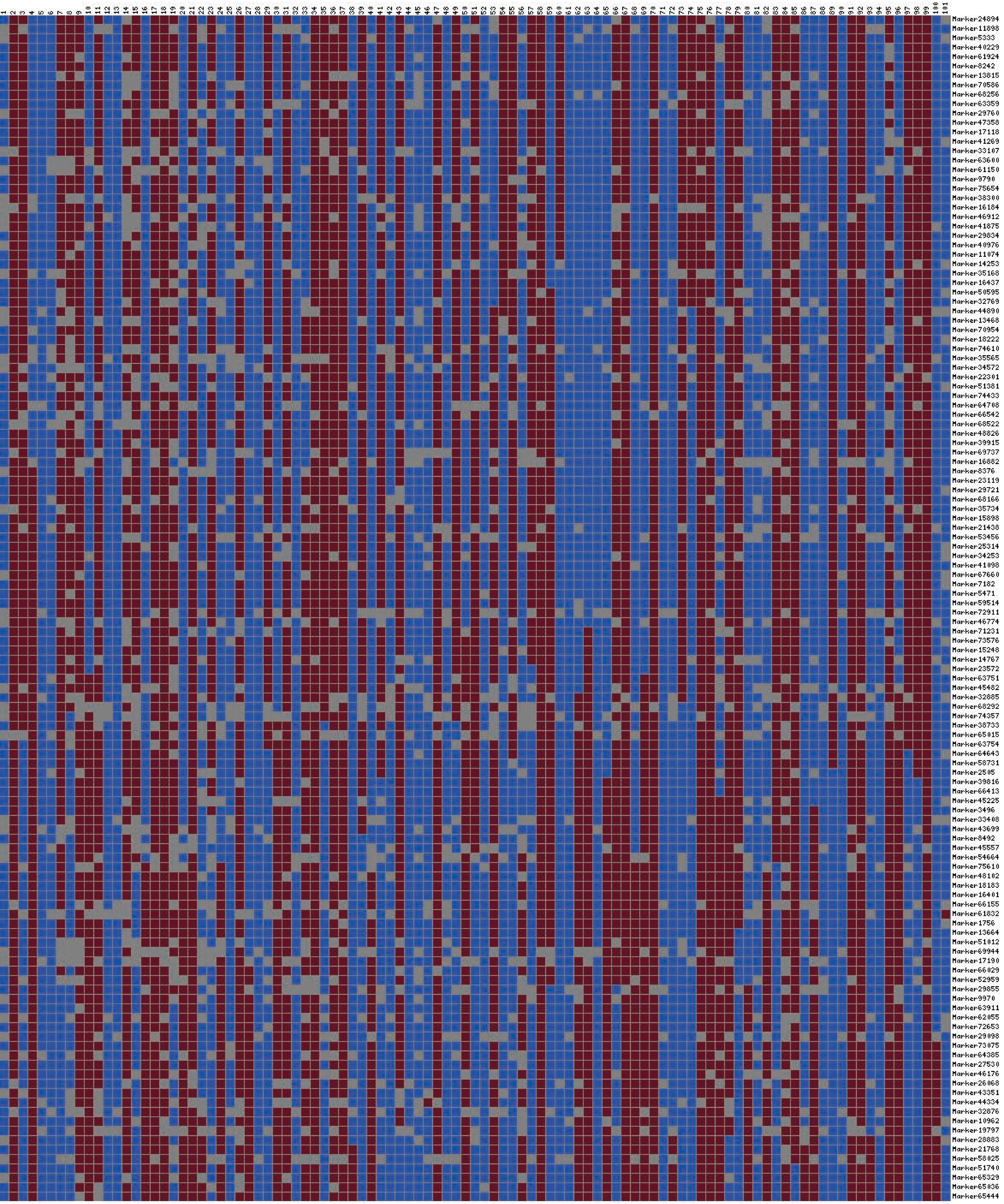
1 2 3 4 5 6 7 8 9 10 11 12 13 14 15 16 17 18 19 20 21 22 23 24 25 26 27 28 29 30 31 32 33 34 35 36 37 38 39 40 41 42 43 44 45 46 47 48 49 50 51 52 53 54 55 56 57 58 59 60 61 62 63 64 65 66 67 68 69 70 71 72 73 74 75 76 77 78 79 80 81 82 83 84 85 86 87 88 89 90 91 92 93 94 95 96 97 98 99 100 101
Marker24894
Marker11898
Marker5333
Marker40229
Marker61924
Marker8242
Marker13815
Marker70586
Marker68256
Marker63359
Marker29760
Marker47358
Marker17118
Marker41269
Marker33107
Marker63600
Marker61150
Marker9790
Marker75654
Marker38300
Marker16184
Marker46912
Marker41875
Marker29834
Marker40976
Marker11074
Marker14253
Marker35168
Marker16437
Marker50595
Marker32769
Marker44890
Marker13468
Marker70954
Marker18222
Marker74610
Marker35565
Marker34572
Marker22301
Marker51381
Marker74433
Marker64708
Marker66542
Marker68522
Marker48826
Marker39915
Marker69737
Marker16882
Marker8376
Marker23119
Marker29721
Marker68166
Marker35734
Marker15898
Marker21438
Marker53456
Marker25314
Marker34253
Marker41098
Marker67660
Marker7182
Marker5471
Marker59514
Marker72911
Marker46774
Marker71231
Marker73576
Marker15248
Marker14767
Marker23572
Marker63751
Marker45482
Marker32885
Marker68292
Marker74357
Marker38733
Marker65015
Marker63754
Marker64643
Marker58731
Marker2505
Marker39816
Marker66413
Marker45225
Marker3496
Marker33408
Marker43699
Marker8492
Marker45557
Marker54664
Marker75610
Marker48102
Marker18183
Marker16401
Marker66155
Marker61832
Marker1756
Marker13664
Marker51012
Marker69944
Marker17190
Marker66029
Marker52959
Marker29855
Marker9970
Marker63911
Marker62055
Marker72653
Marker29098
Marker73075
Marker64385
Marker27530
Marker46176
Marker26068
Marker43351
Marker44334
Marker32876
Marker10962
Marker19797
Marker28883
Marker21768
Marker58025
Marker51740
Marker65329
Marker65036
Marker65444

# LG15

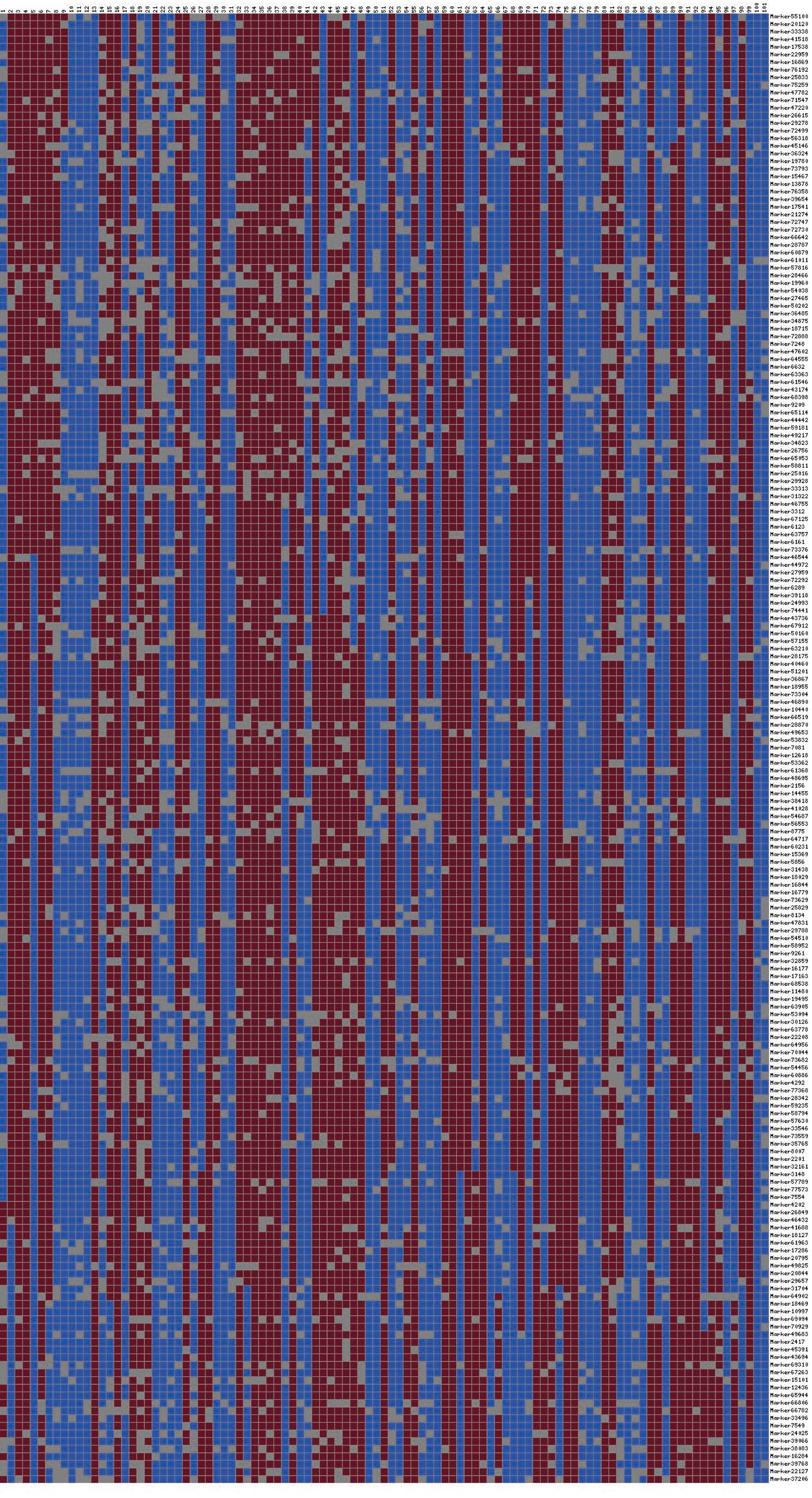

# LG16

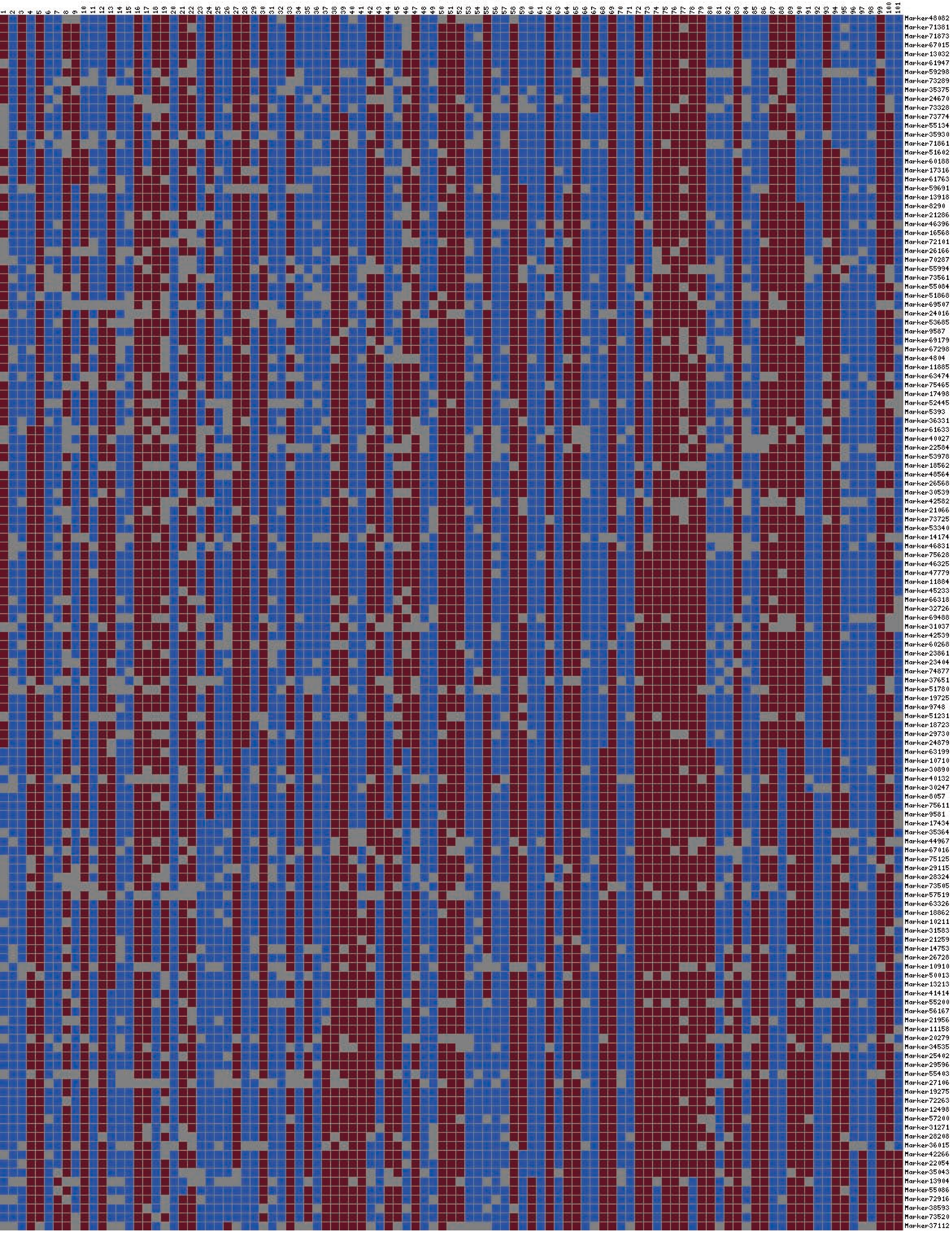

# LG17

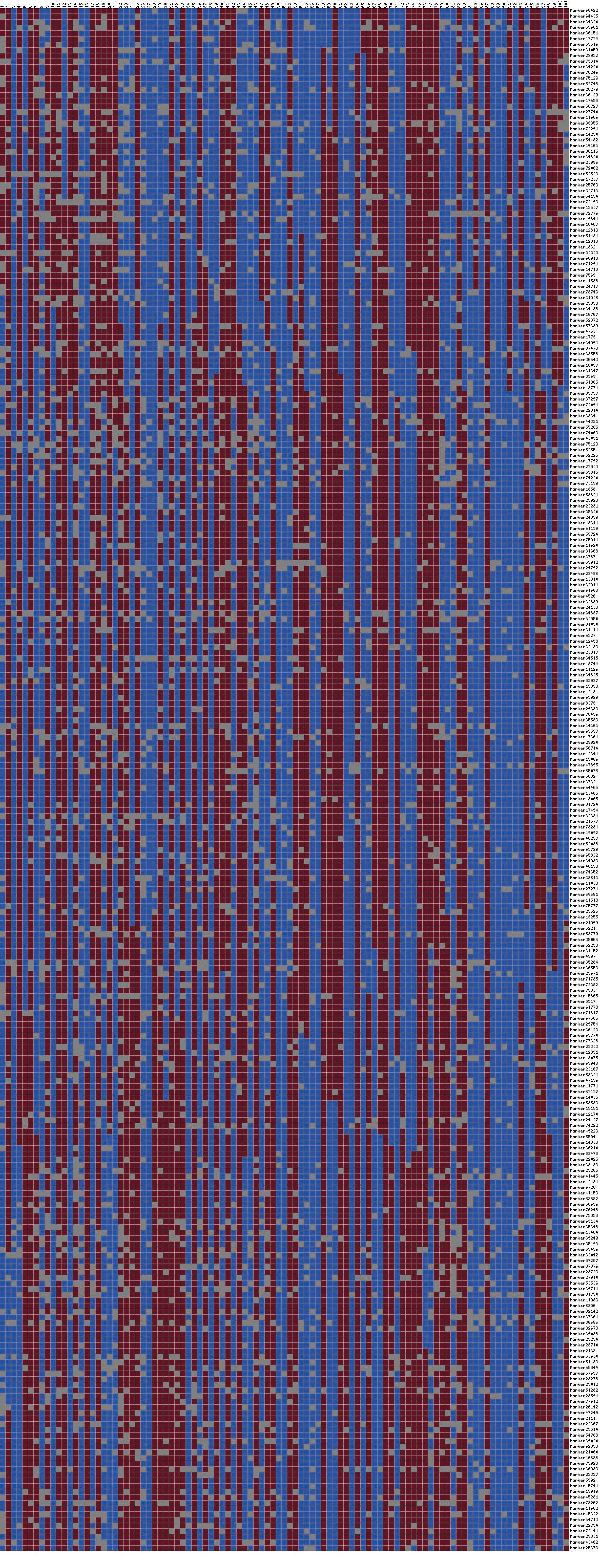

# LG18

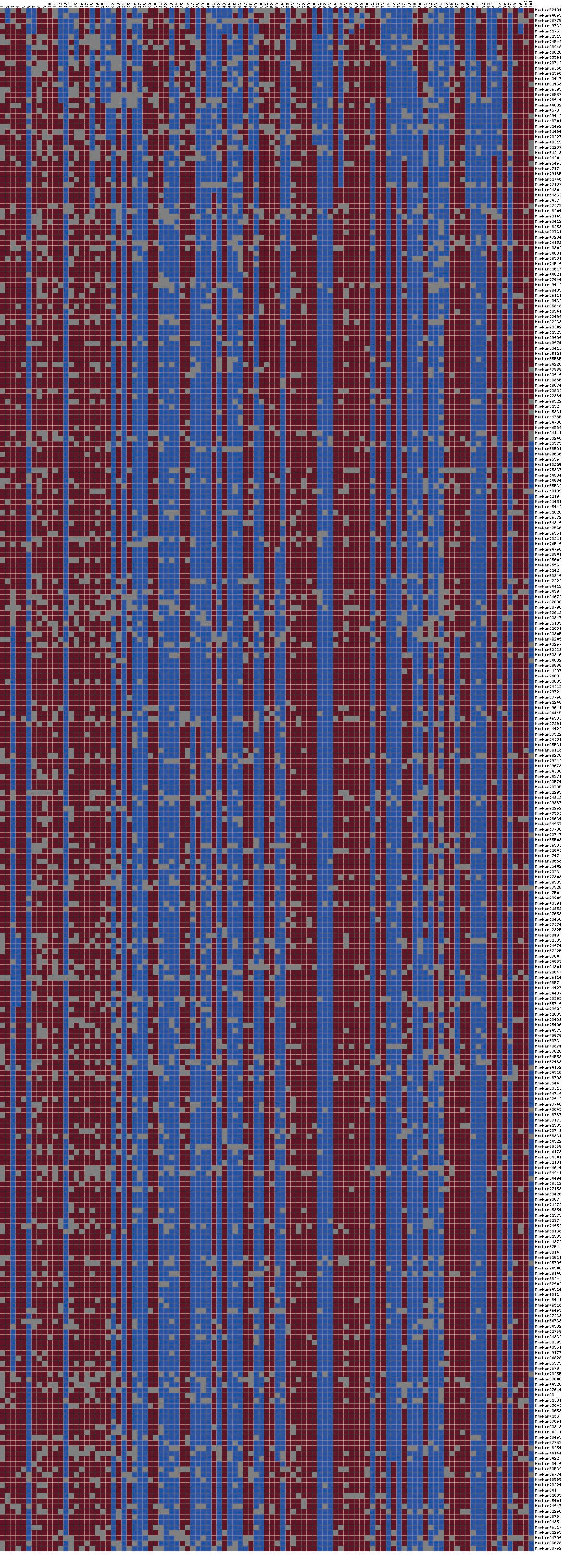

# LG19

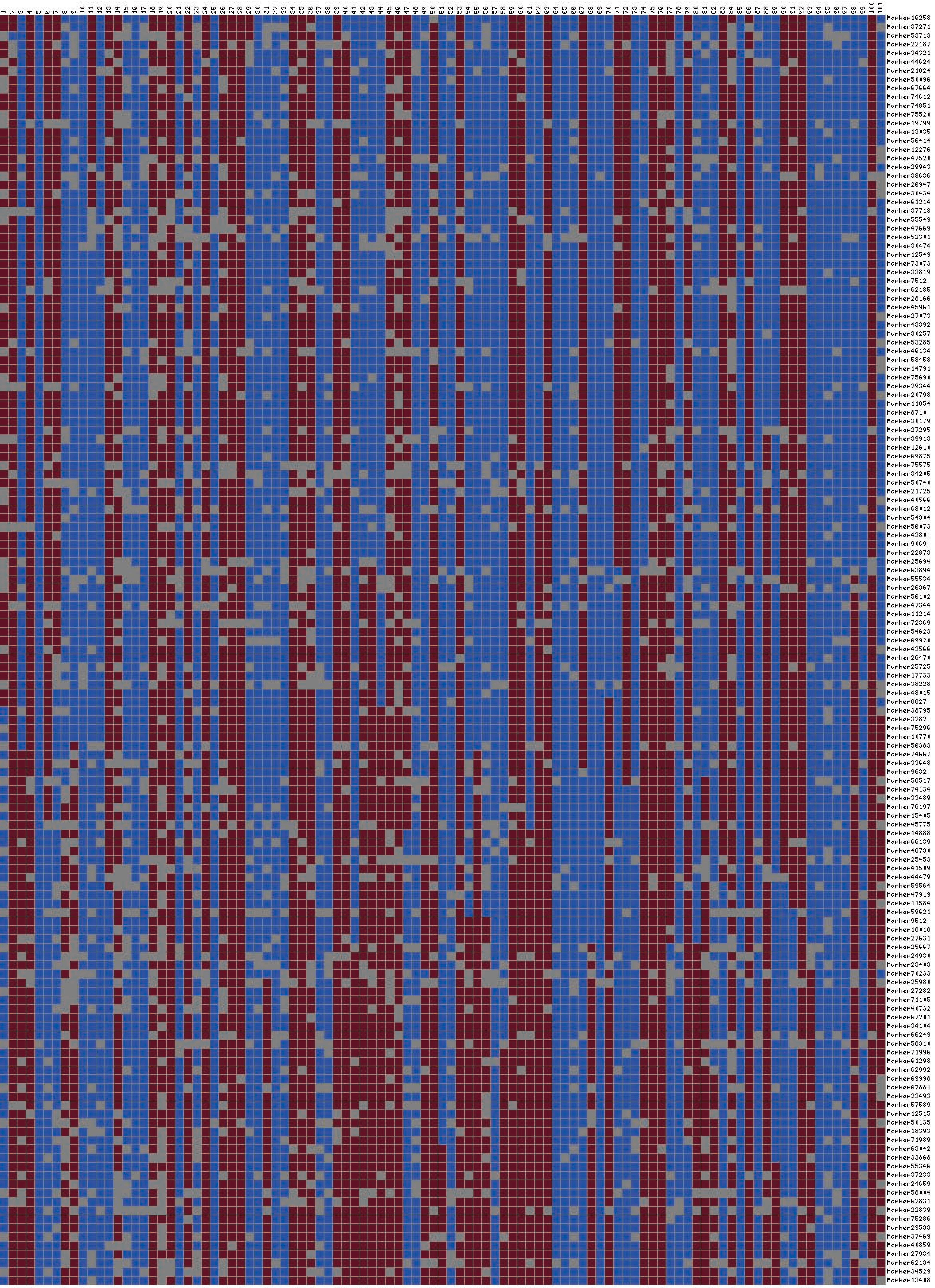

# LG20

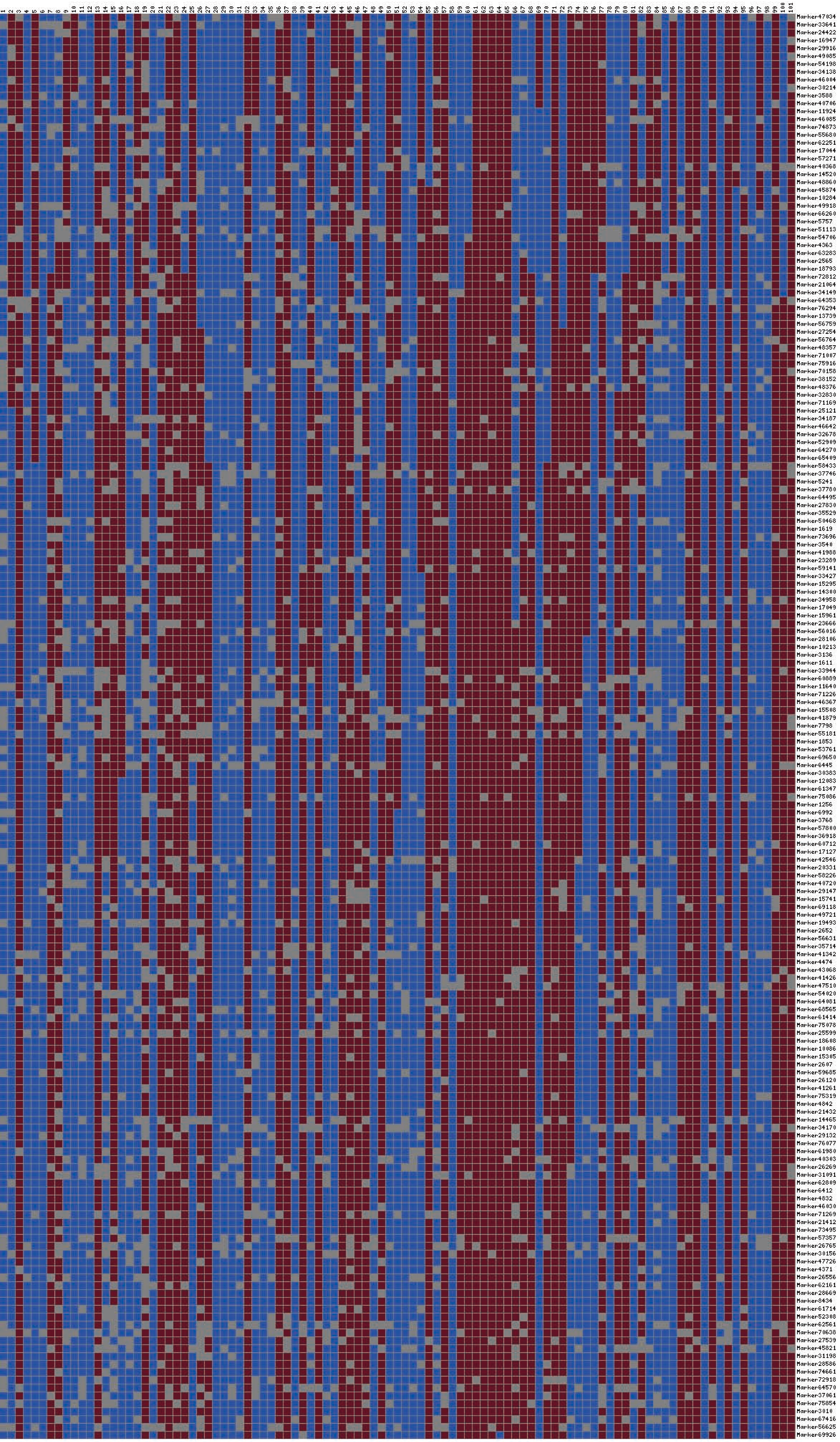

# LG21

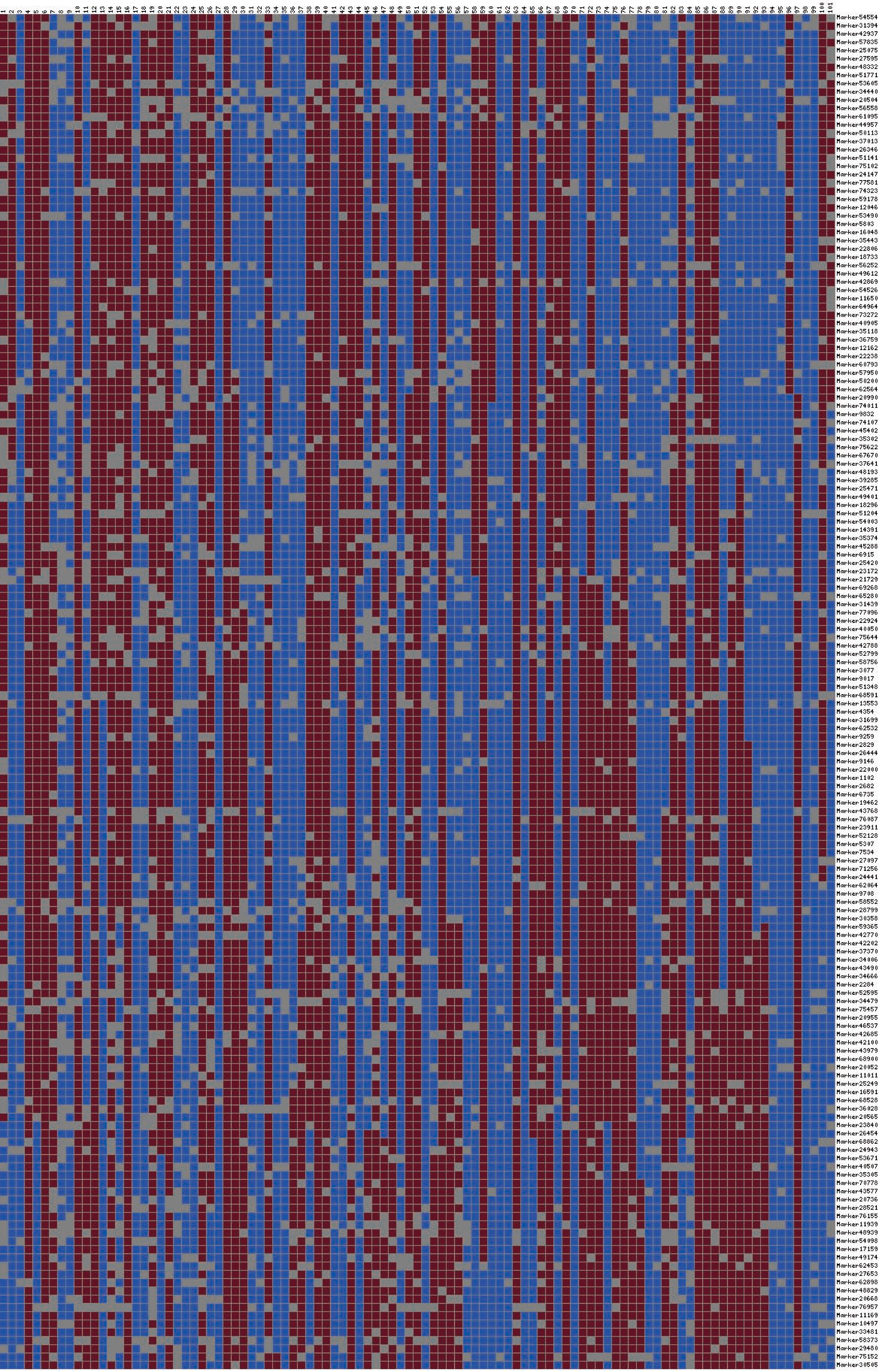

# LG23

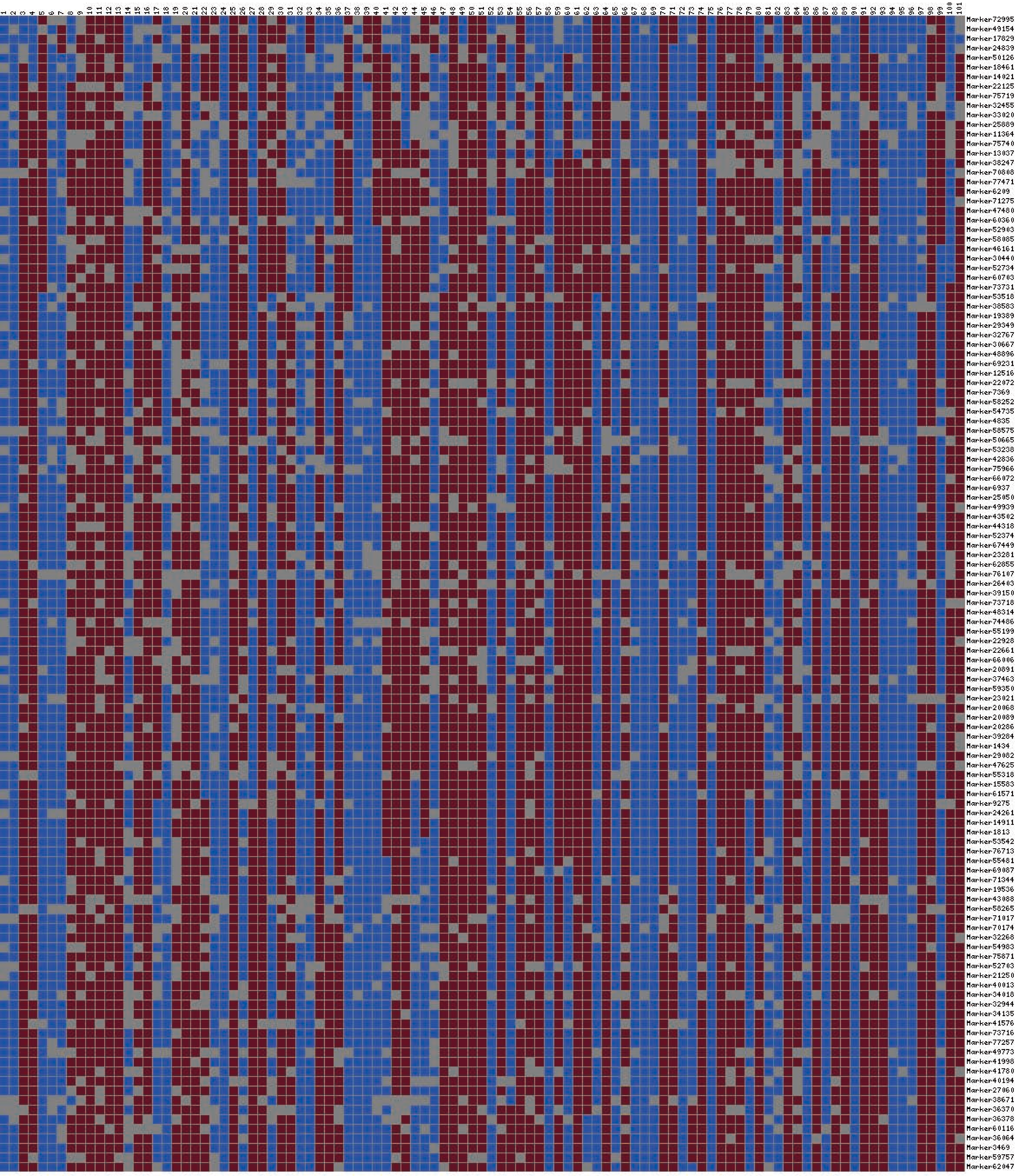

1 2 3 4 5 6 7 8 9 10 11 12 13 14 15 16 17 18 19 20 21 22 23 24 25 26 27 28 29 30 31 32 33 34 35 36 37 38 39 40 41 42 43 44 45 46 47 48 49 50 51 52 53 54 55 56 57 58 59 60 61 62 63 64 65 66 67 68 69 70 71 72 73 74 75 76 77 78 79 80 81 82 83 84 85 86 87 88 89 90 91 92 93 94 95 96 97 98 99 100 101
Marker72995
Marker49154
Marker17829
Marker24839
Marker50126
Marker18461
Marker14021
Marker22125
Marker75719
Marker32455
Marker33020
Marker25889
Marker11364
Marker75740
Marker13037
Marker38247
Marker70808
Marker77471
Marker6209
Marker71275
Marker47480
Marker60360
Marker52903
Marker58085
Marker46161
Marker30440
Marker52734
Marker60703
Marker73731
Marker53518
Marker38583
Marker19389
Marker29349
Marker32767
Marker30667
Marker48896
Marker69231
Marker12516
Marker22072
Marker7369
Marker58252
Marker54735
Marker4835
Marker58575
Marker50665
Marker53238
Marker42836
Marker75966
Marker66072
Marker6937
Marker25050
Marker49939
Marker43502
Marker44318
Marker52374
Marker67449
Marker23281
Marker62855
Marker76107
Marker26403
Marker39150
Marker73718
Marker48314
Marker74486
Marker55199
Marker22928
Marker22661
Marker66006
Marker20891
Marker37463
Marker59350
Marker23021
Marker20068
Marker20089
Marker20286
Marker39284
Marker1434
Marker29082
Marker47625
Marker55318
Marker15583
Marker61571
Marker9275
Marker24261
Marker14911
Marker1813
Marker53542
Marker76713
Marker55481
Marker69087
Marker71344
Marker19536
Marker43088
Marker58265
Marker71017
Marker70174
Marker32268
Marker54983
Marker75871
Marker52703
Marker21250
Marker40013
Marker34018
Marker32944
Marker34135
Marker41576
Marker73716
Marker77257
Marker49773
Marker41998
Marker41780
Marker40194
Marker27060
Marker38671
Marker36370
Marker36378
Marker60116
Marker36064
Marker3469
Marker59757
Marker62047

# LG24

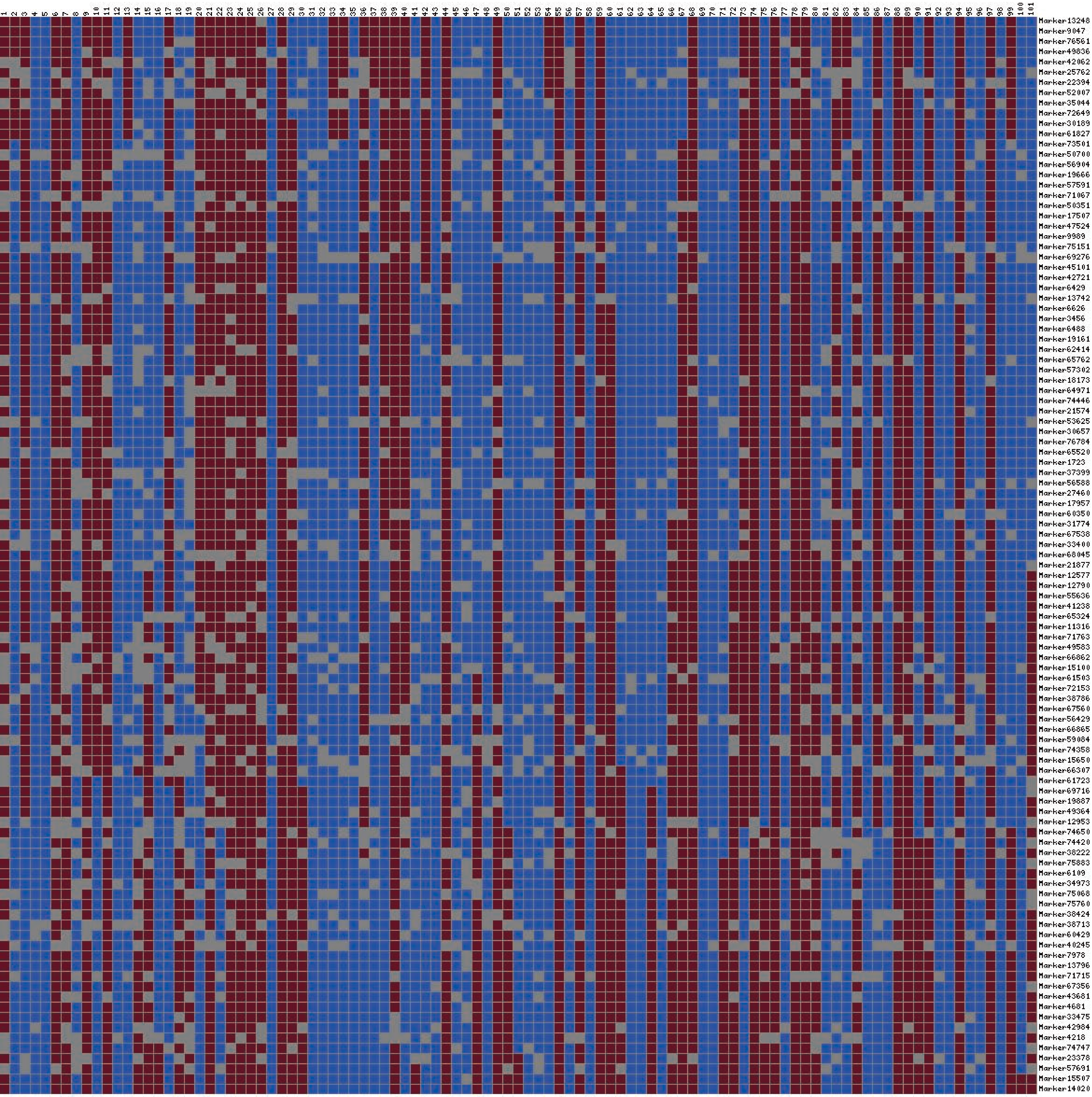
1 2 3 4 5 6 7 8 9 10 11 12 13 14 15 16 17 18 19 20 21 22 23 24 25 26 27 28 29 30 31 32 33 34 35 36 37 38 39 40 41 42 43 44 45 46 47 48 49 50 51 52 53 54 55 56 57 58 59 60 61 62 63 64 65 66 67 68 69 70 71 72 73 74 75 76 77 78 79 80 81 82 83 84 85 86 87 88 89 90 91 92 93 94 95 96 97 98 99 100 101
Marker13248
Marker9047
Marker76561
Marker49836
Marker42062
Marker25762
Marker22394
Marker52007
Marker35044
Marker72649
Marker30189
Marker61827
Marker73501
Marker50700
Marker56904
Marker19666
Marker57591
Marker71067
Marker50351
Marker17507
Marker47524
Marker9989
Marker75151
Marker69276
Marker45101
Marker42721
Marker6429
Marker13742
Marker6626
Marker3456
Marker6488
Marker19161
Marker62414
Marker65762
Marker57302
Marker18173
Marker64971
Marker74446
Marker21574
Marker53625
Marker30657
Marker76784
Marker65520
Marker1723
Marker37399
Marker56588
Marker27460
Marker17957
Marker60350
Marker31774
Marker67538
Marker33400
Marker68045
Marker21877
Marker12577
Marker12790
Marker55636
Marker41238
Marker65324
Marker11316
Marker71763
Marker49583
Marker66862
Marker15100
Marker61503
Marker72153
Marker38786
Marker67560
Marker56429
Marker66865
Marker59084
Marker74358
Marker15650
Marker66307
Marker61723
Marker69716
Marker19887
Marker49364
Marker12953
Marker74650
Marker74420
Marker38222
Marker75883
Marker6109
Marker34973
Marker75068
Marker75760
Marker38424
Marker38713
Marker60429
Marker40245
Marker7978
Marker13796
Marker71715
Marker67356
Marker43681
Marker4681
Marker33475
Marker42984
Marker4218
Marker74747
Marker23378
Marker57691
Marker15507
Marker14020

# LG25

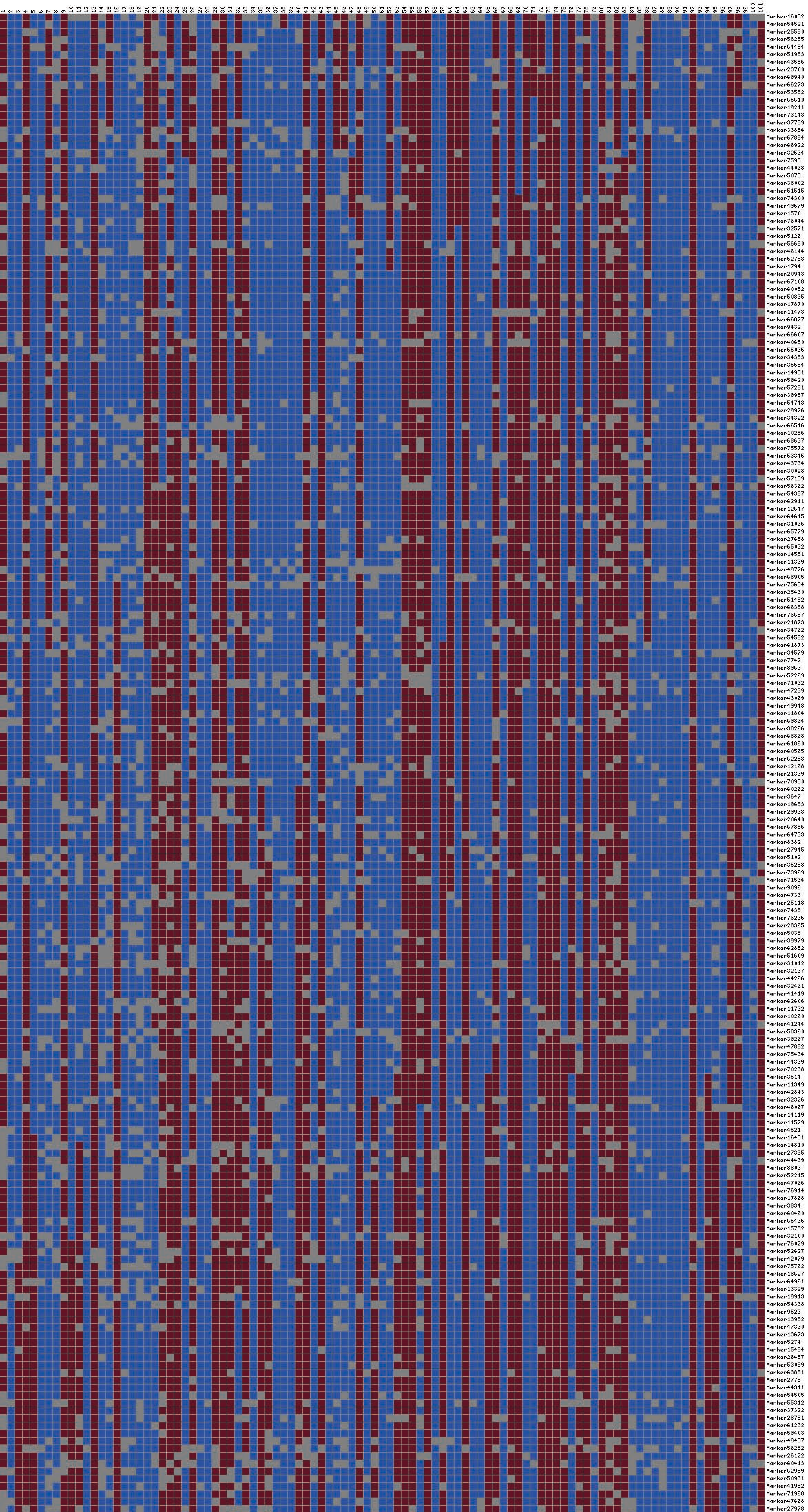

# LG26

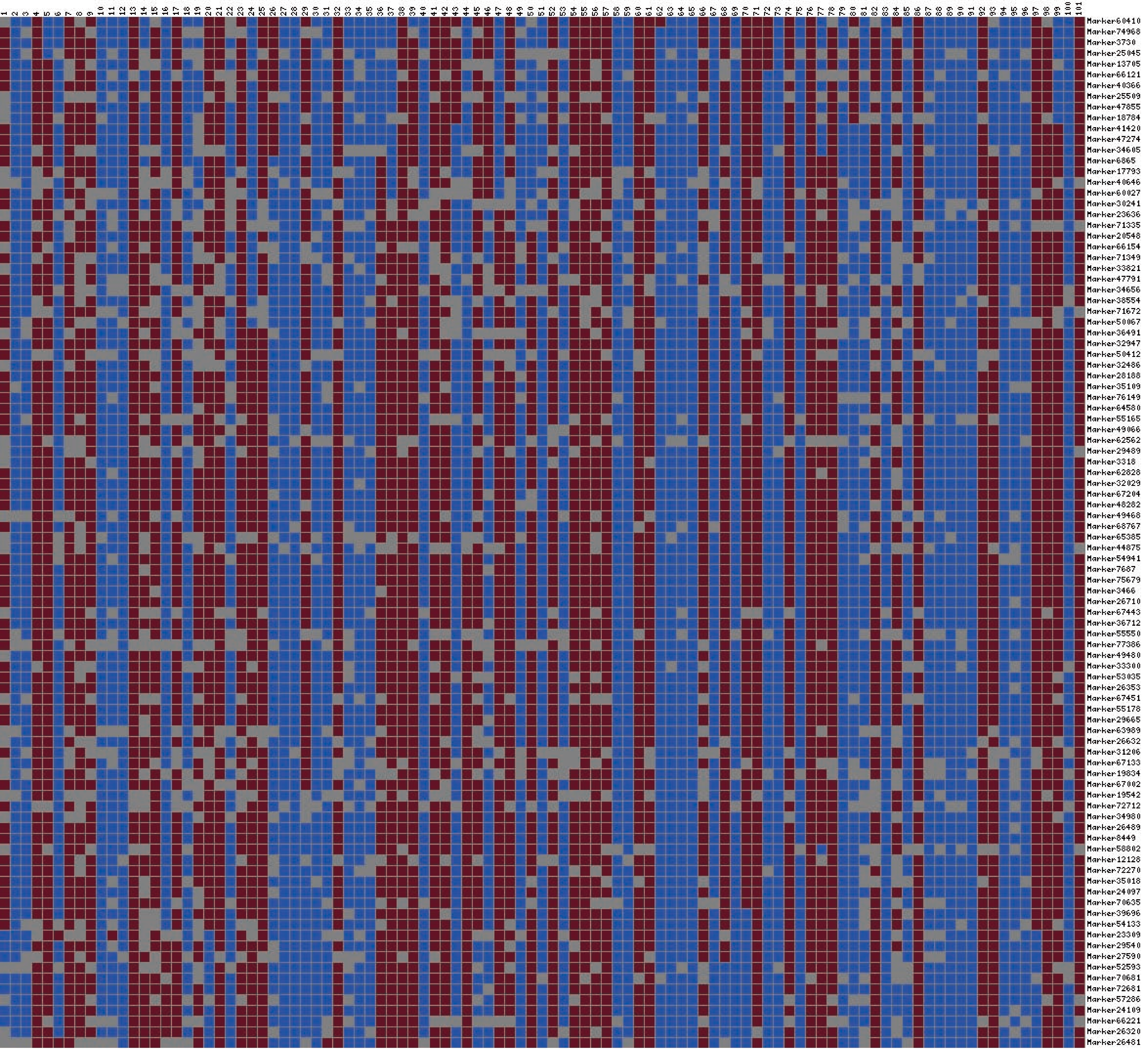

# LG27

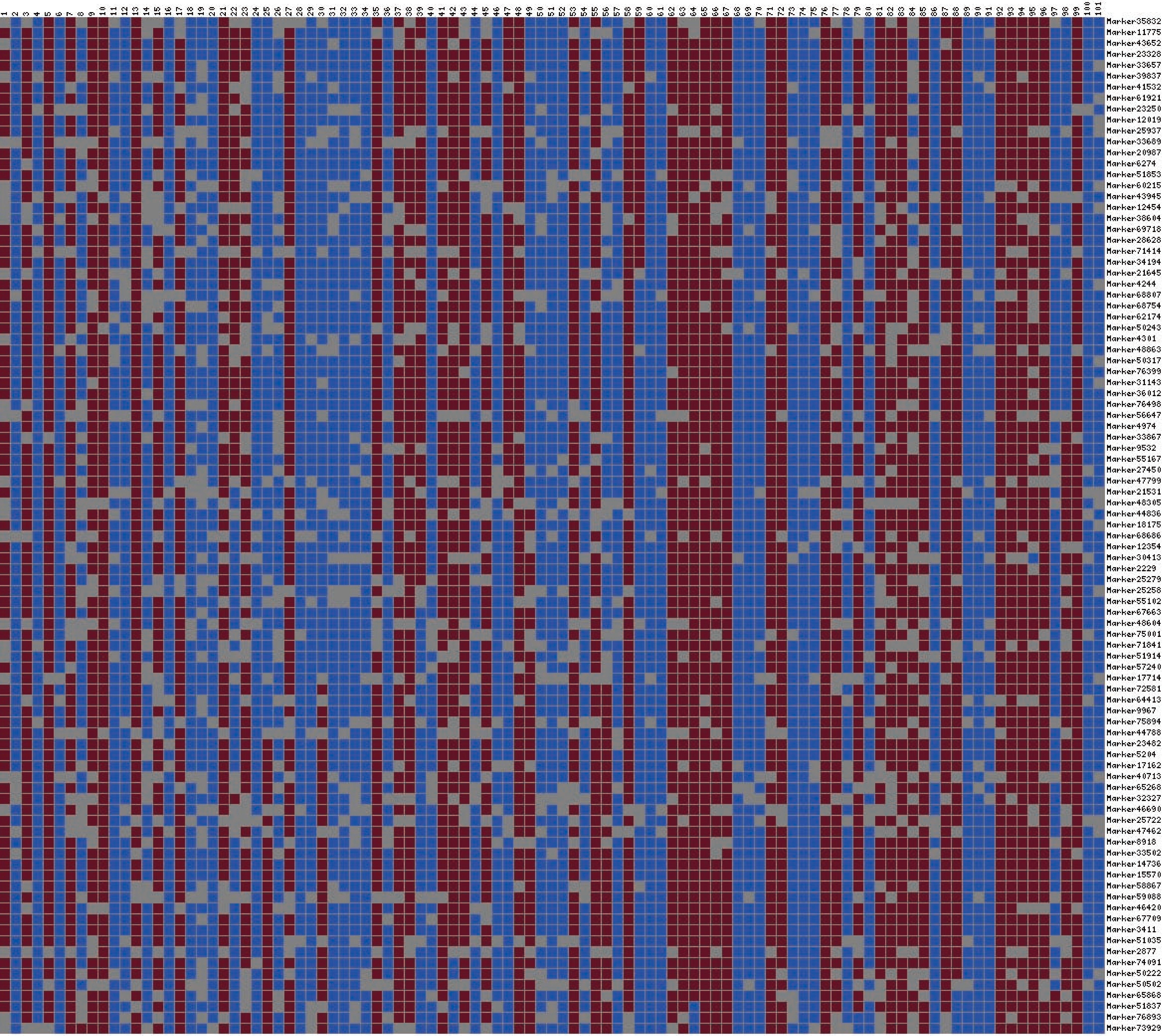

1 2 3 4 5 6 7 8 9 10 11 12 13 14 15 16 17 18 19 20 21 22 23 24 25 26 27 28 29 30 31 32 33 34 35 36 37 38 39 40 41 42 43 44 45 46 47 48 49 50 51 52 53 54 55 56 57 58 59 60 61 62 63 64 65 66 67 68 69 70 71 72 73 74 75 76 77 78 79 80 81 82 83 84 85 86 87 88 89 90 91 92 93 94 95 96 97 98 99 100 101
Marker35832
Marker11775
Marker43652
Marker23328
Marker33657
Marker39837
Marker41532
Marker61921
Marker23250
Marker12019
Marker25937
Marker33689
Marker20987
Marker6274
Marker51853
Marker60215
Marker43945
Marker12454
Marker38604
Marker69718
Marker28628
Marker71414
Marker34194
Marker21645
Marker4244
Marker68807
Marker68754
Marker62174
Marker50243
Marker4301
Marker48863
Marker50317
Marker76399
Marker31143
Marker36012
Marker76498
Marker56647
Marker4974
Marker33867
Marker9532
Marker55167
Marker27450
Marker47799
Marker21531
Marker48305
Marker44836
Marker18175
Marker68686
Marker12354
Marker30413
Marker2229
Marker25279
Marker25258
Marker55102
Marker67663
Marker48604
Marker75001
Marker71841
Marker51914
Marker57240
Marker17714
Marker72581
Marker64413
Marker9967
Marker75894
Marker44788
Marker23482
Marker5204
Marker17162
Marker40713
Marker65268
Marker32327
Marker46690
Marker25722
Marker47462
Marker8918
Marker33502
Marker14736
Marker15570
Marker58867
Marker59088
Marker46420
Marker67709
Marker3411
Marker51035
Marker2877
Marker74091
Marker50222
Marker50502
Marker65868
Marker51837
Marker76893
Marker73929

# LG28

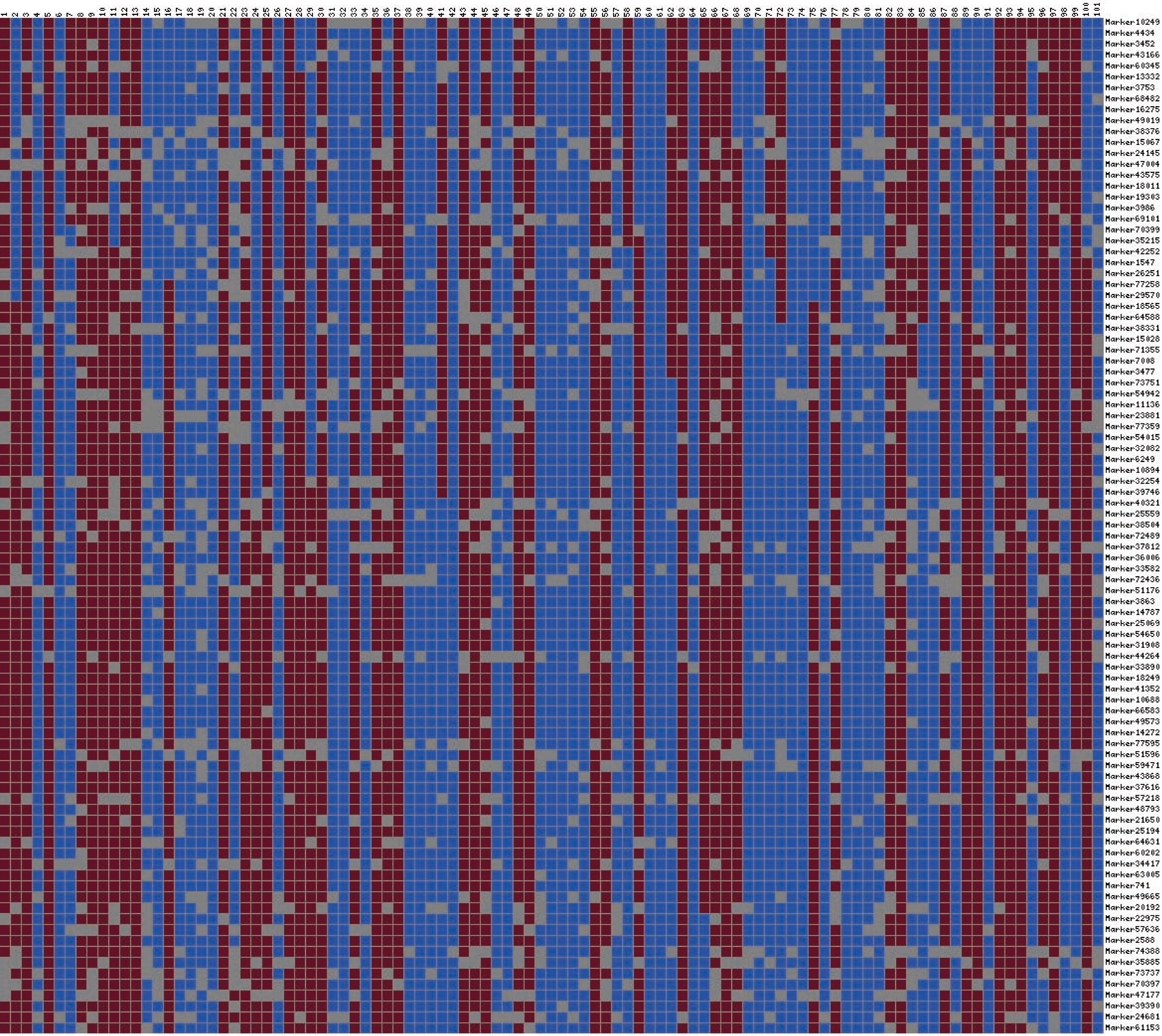
1 2 3 4 5 6 7 8 9 10 11 12 13 14 15 16 17 18 19 20 21 22 23 24 25 26 27 28 29 30 31 32 33 34 35 36 37 38 39 40 41 42 43 44 45 46 47 48 49 50 51 52 53 54 55 56 57 58 59 60 61 62 63 64 65 66 67 68 69 70 71 72 73 74 75 76 77 78 79 80 81 82 83 84 85 86 87 88 89 90 91 92 93 94 95 96 97 98 99 100 101
Marker10249
Marker4434
Marker3452
Marker43166
Marker60345
Marker13332
Marker3753
Marker68482
Marker16275
Marker49019
Marker38376
Marker15067
Marker24145
Marker47004
Marker43575
Marker18011
Marker19303
Marker3986
Marker69101
Marker70399
Marker35215
Marker42252
Marker1547
Marker26251
Marker77258
Marker29570
Marker18565
Marker64588
Marker38331
Marker15028
Marker71355
Marker7008
Marker3477
Marker73751
Marker54942
Marker11136
Marker23881
Marker77359
Marker54015
Marker32082
Marker6249
Marker10894
Marker32254
Marker39746
Marker40321
Marker25559
Marker38504
Marker72489
Marker37812
Marker36006
Marker33582
Marker72436
Marker51176
Marker3863
Marker14787
Marker25069
Marker54650
Marker31908
Marker44264
Marker33890
Marker18249
Marker41352
Marker10688
Marker66583
Marker49573
Marker14272
Marker77595
Marker51596
Marker59471
Marker43868
Marker37616
Marker57218
Marker48793
Marker21650
Marker25194
Marker64631
Marker60202
Marker34417
Marker63005
Marker741
Marker49665
Marker20192
Marker22975
Marker57636
Marker2588
Marker74388
Marker35885
Marker73737
Marker70397
Marker47177
Marker39390
Marker24681
Marker61153

# LG29

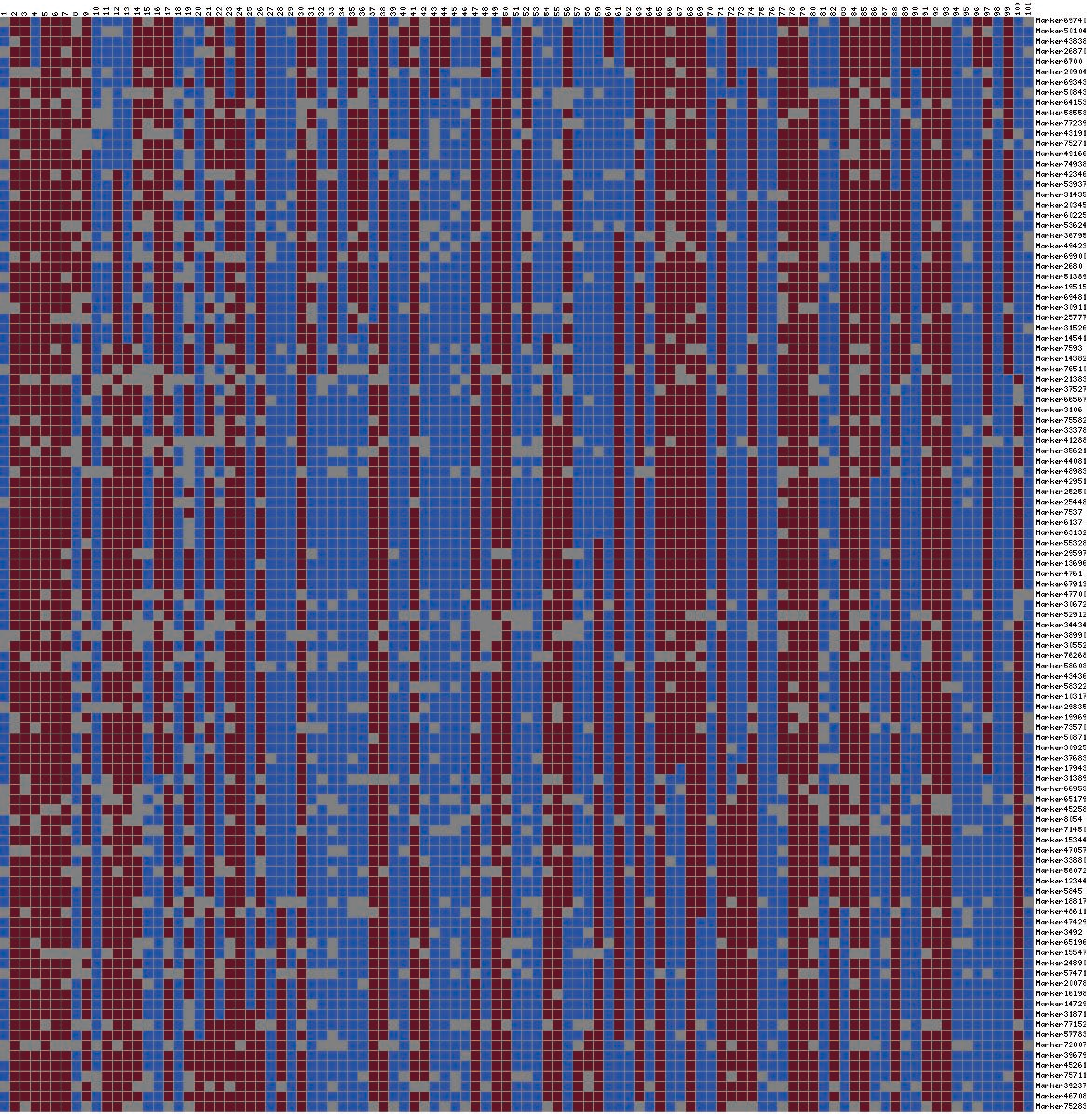

1 2 3 4 5 6 7 8 9 10 11 12 13 14 15 16 17 18 19 20 21 22 23 24 25 26 27 28 29 30 31 32 33 34 35 36 37 38 39 40 41 42 43 44 45 46 47 48 49 50 51 52 53 54 55 56 57 58 59 60 61 62 63 64 65 66 67 68 69 70 71 72 73 74 75 76 77 78 79 80 81 82 83 84 85 86 87 88 89 90 91 92 93 94 95 96 97 98 99 100 101
Marker69740
Marker50104
Marker43838
Marker26870
Marker6700
Marker20904
Marker69343
Marker50843
Marker64153
Marker58553
Marker77239
Marker43191
Marker75271
Marker49166
Marker74938
Marker42346
Marker53937
Marker31435
Marker20345
Marker60225
Marker53624
Marker36795
Marker49423
Marker69900
Marker2680
Marker51389
Marker19515
Marker69481
Marker30911
Marker25777
Marker31526
Marker14541
Marker7593
Marker14382
Marker76510
Marker21383
Marker37527
Marker66567
Marker3106
Marker75582
Marker33378
Marker41288
Marker35621
Marker44081
Marker48983
Marker42951
Marker25250
Marker25448
Marker7537
Marker6137
Marker63132
Marker55328
Marker29597
Marker13696
Marker4761
Marker67913
Marker47700
Marker30672
Marker52912
Marker34434
Marker38990
Marker30552
Marker76268
Marker58603
Marker43436
Marker58322
Marker10317
Marker29835
Marker19969
Marker73570
Marker50871
Marker30925
Marker37683
Marker17943
Marker31389
Marker66953
Marker65179
Marker45258
Marker8054
Marker71450
Marker15344
Marker47057
Marker33880
Marker56072
Marker12344
Marker5845
Marker18817
Marker48611
Marker47429
Marker3492
Marker65196
Marker15547
Marker24890
Marker57471
Marker20078
Marker16198
Marker14729
Marker31871
Marker77152
Marker57783
Marker72007
Marker39679
Marker45261
Marker75711
Marker39237
Marker46708
Marker75283

LG30

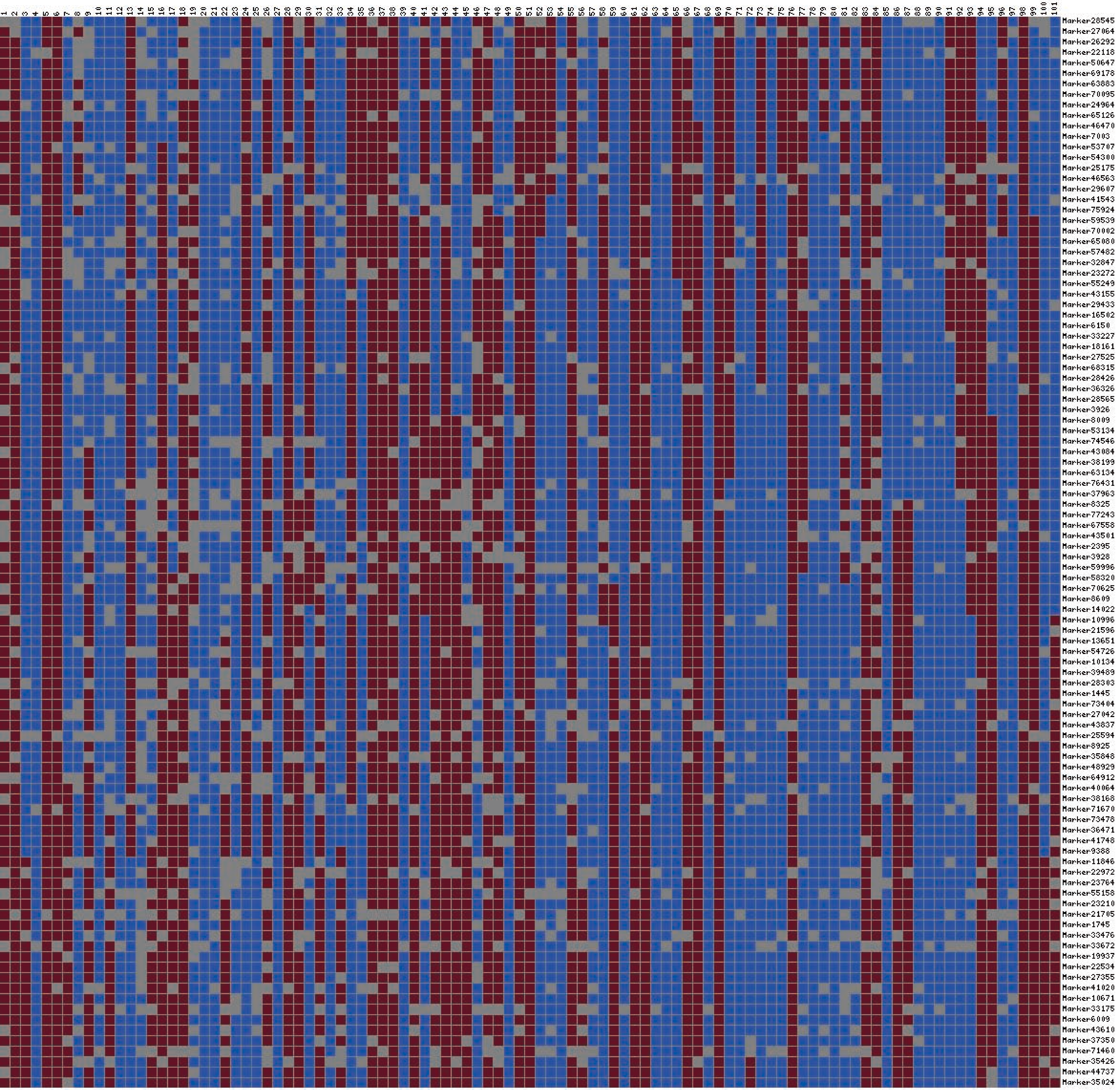

Supplement: Additional file 3: — Haplotype map of the genetic map. Blue and red represent markers originating from maternal and paternal parent, respectively. Gray represents missing data. Rows indicate the markers on the linkage group and columns the genotype of an individual. (PDF 27195 kb) [file 12864_2015_2184_MOESM3_ESM.pdf]
