# Supplementary material for: Construction of a high-density genetic map and mapping of a sex-linked locus for the brown alga Undaria pinnatifida (Phaeophyceae) based on large scale marker development by specific length amplified fragment (SLAF) sequencing
Source: BMC Genomics. 2015 Nov 5;16:902. doi: 10.1186/s12864-015-2184-y (PMC4635539; doi:10.1186/s12864-015-2184-y)
Supplement: Additional file 4: — Heat map of the genetic map: Each cell represents the recombination rate between two markers. The color change from yellow through red to purple indicates the change of recombination rate from low to high. (PDF 23911 kb) [file 12864_2015_2184_MOESM4_ESM.pdf]

LG1

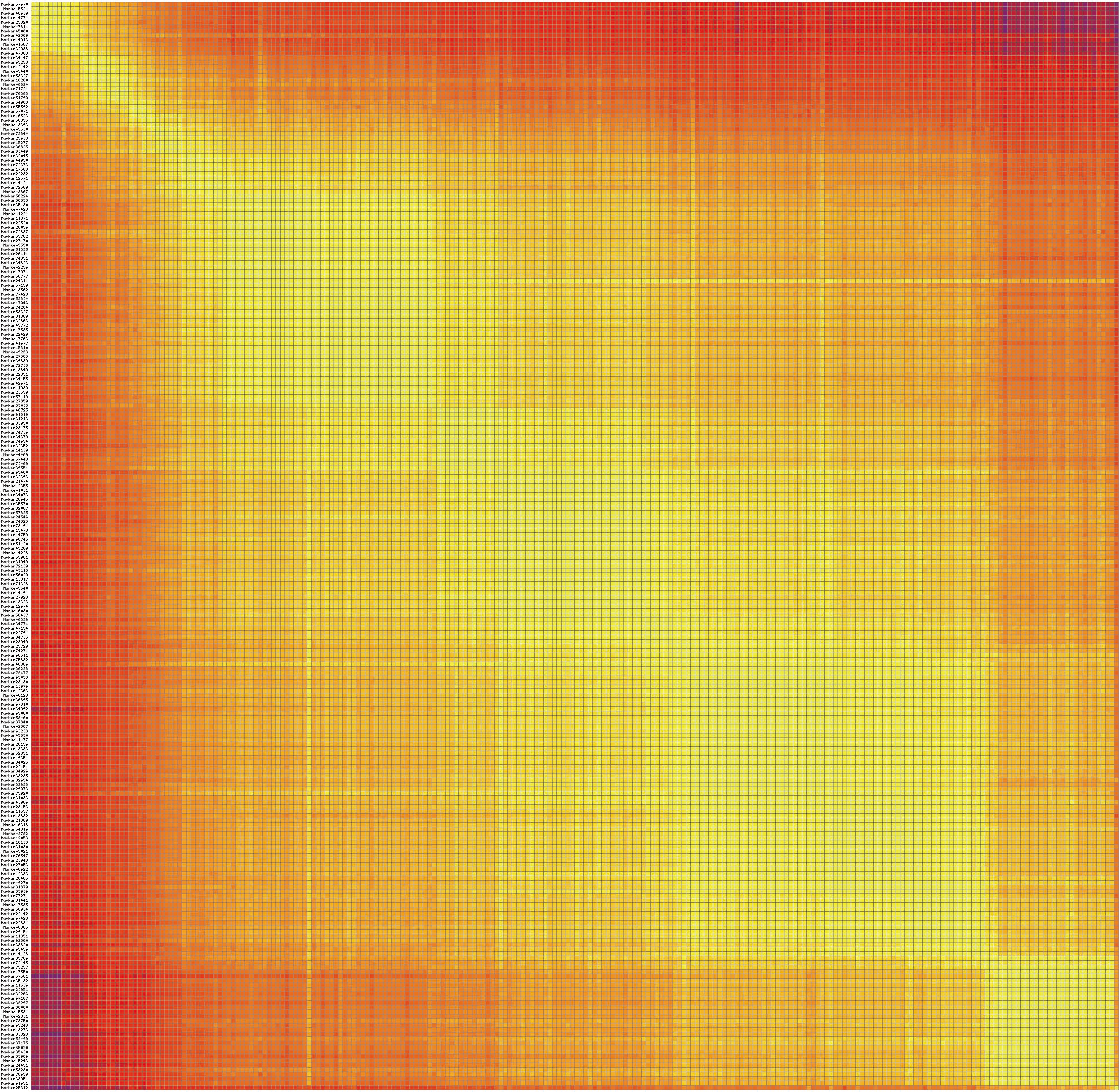

LG2

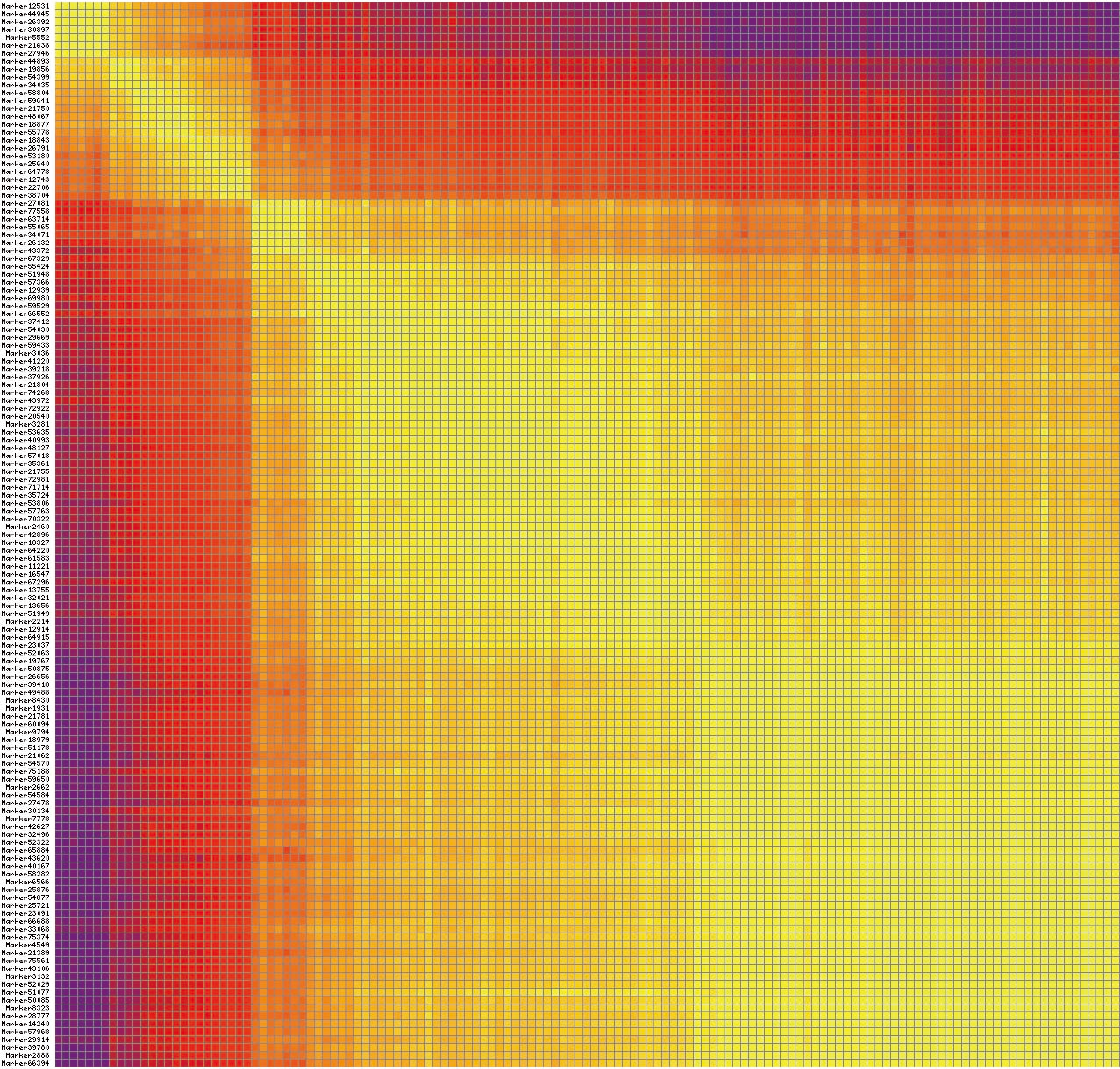
Marker12531
Marker44945
Marker26392
Marker30897
Marker5552
Marker21638
Marker27946
Marker44893
Marker19856
Marker54399
Marker34035
Marker58804
Marker59641
Marker21750
Marker48067
Marker18877
Marker55778
Marker18843
Marker26791
Marker53180
Marker25640
Marker64778
Marker12743
Marker22706
Marker38704
Marker27081
Marker77558
Marker63714
Marker55065
Marker34071
Marker26132
Marker43372
Marker67329
Marker55424
Marker51948
Marker57366
Marker12939
Marker69980
Marker59529
Marker66552
Marker37412
Marker54030
Marker29669
Marker59433
Marker3036
Marker41220
Marker39218
Marker37926
Marker21804
Marker74268
Marker43972
Marker72922
Marker20540
Marker3281
Marker53635
Marker40993
Marker48127
Marker57018
Marker35361
Marker21755
Marker72981
Marker71714
Marker35724
Marker53806
Marker57763
Marker70322
Marker2460
Marker42896
Marker18327
Marker64220
Marker61583
Marker11221
Marker16547
Marker67296
Marker13755
Marker32021
Marker13656
Marker51949
Marker2214
Marker12914
Marker64915
Marker23037
Marker52063
Marker19767
Marker50875
Marker26656
Marker39418
Marker49488
Marker8430
Marker1931
Marker21781
Marker60094
Marker9794
Marker18979
Marker51178
Marker21062
Marker54570
Marker75188
Marker59650
Marker2662
Marker54584
Marker27478
Marker30134
Marker7778
Marker42627
Marker32496
Marker52322
Marker65884
Marker43620
Marker40167
Marker58282
Marker6566
Marker25876
Marker54877
Marker25721
Marker23091
Marker66688
Marker33068
Marker75374
Marker4549
Marker21389
Marker75561
Marker43106
Marker3132
Marker52029
Marker51077
Marker50085
Marker8323
Marker28777
Marker14240
Marker57968
Marker29914
Marker39780
Marker2888
Marker66394

LG3

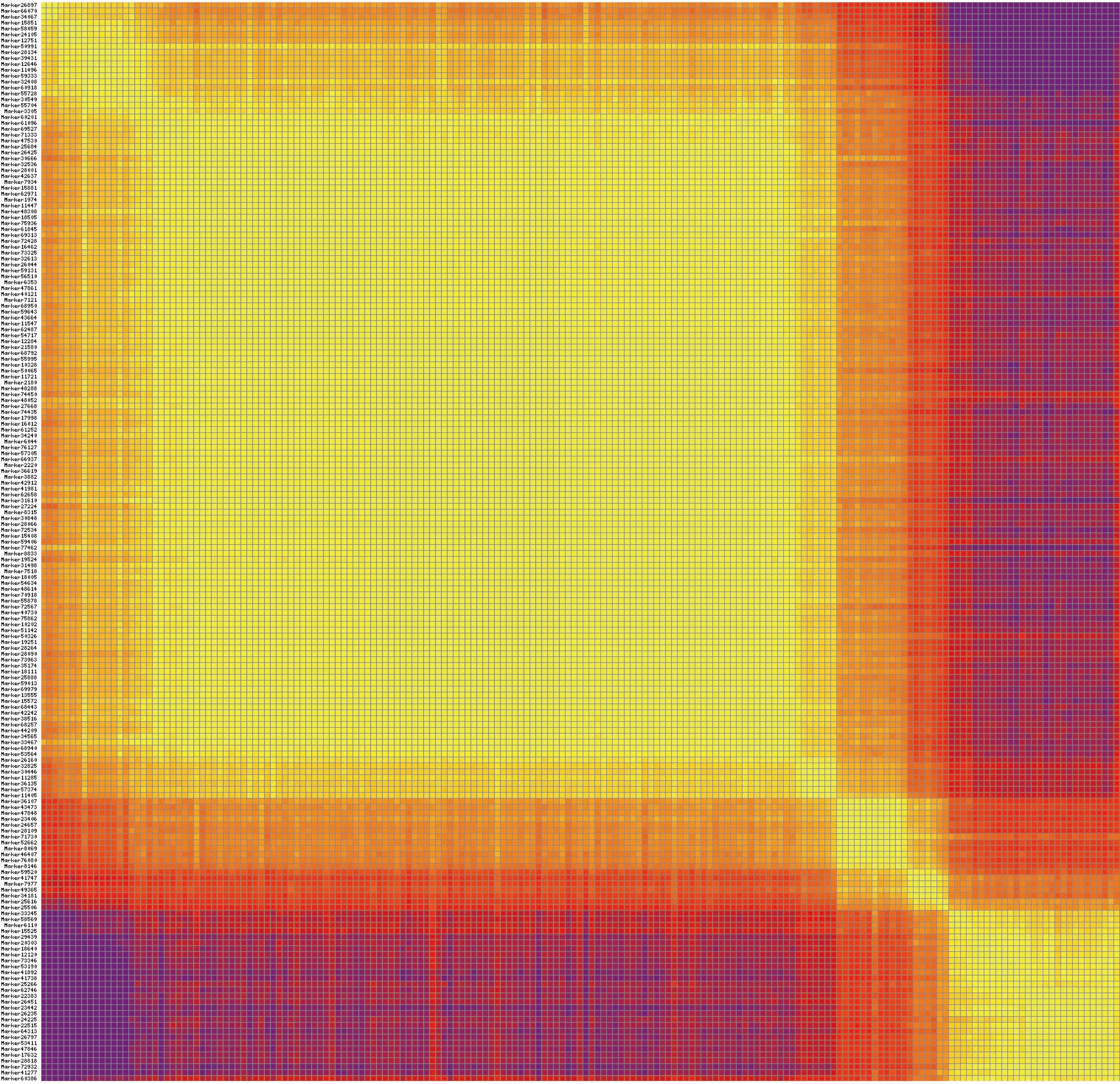

LG4

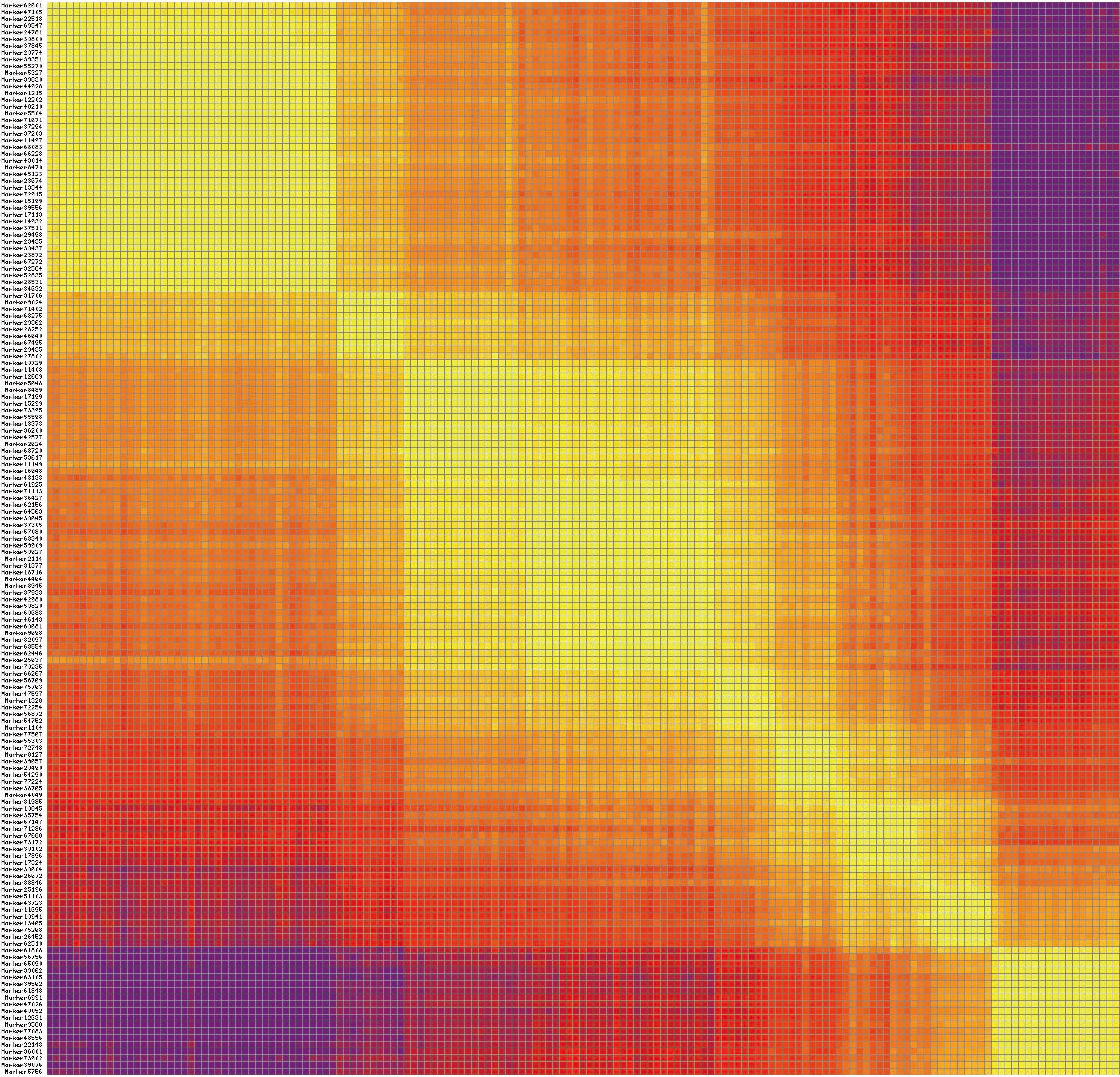

LG5

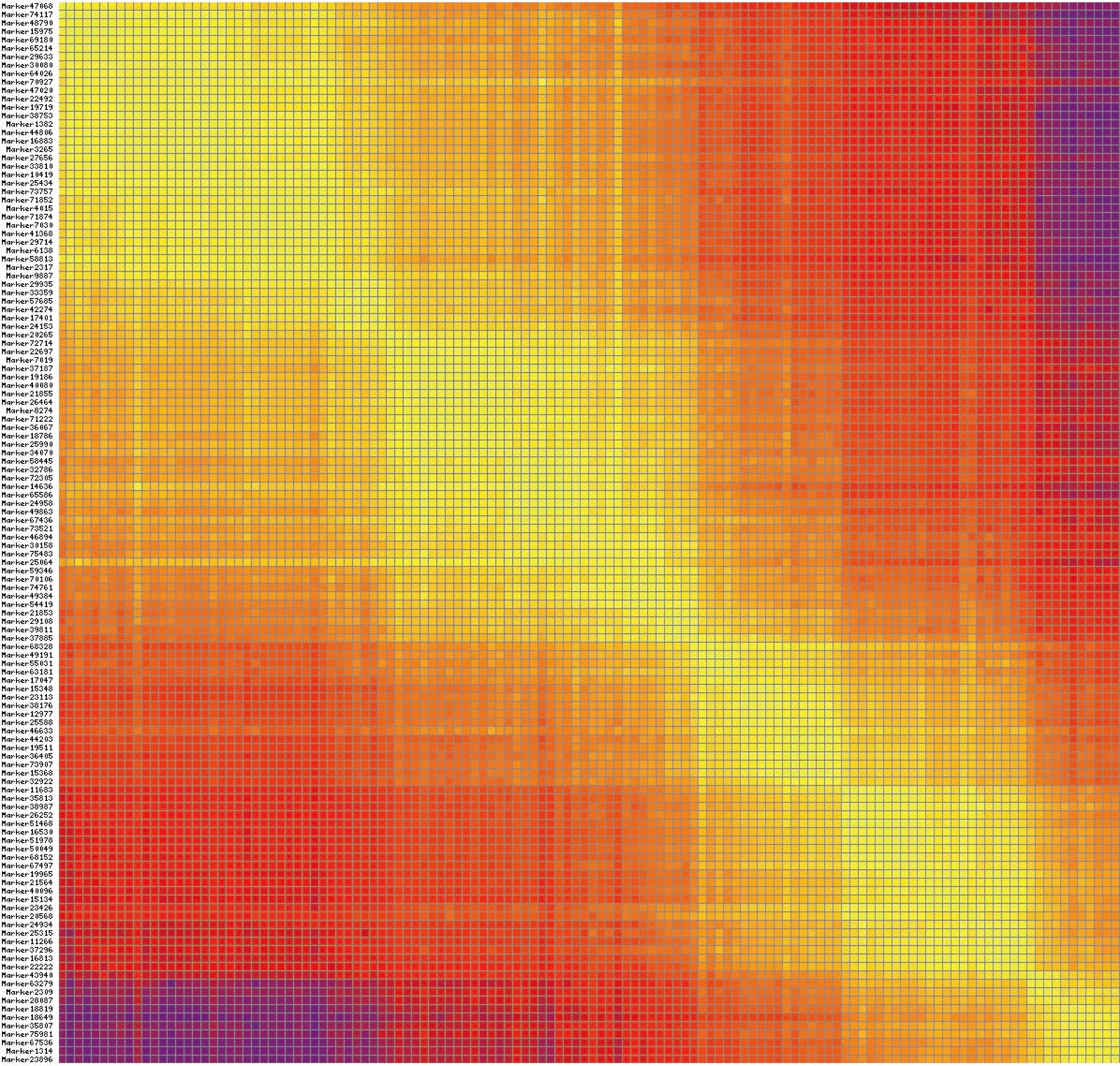

Marker47068
Marker74117
Marker48790
Marker15975
Marker69180
Marker65214
Marker29633
Marker30080
Marker64026
Marker70927
Marker47020
Marker22492
Marker19719
Marker38753
Marker1382
Marker44806
Marker16883
Marker3265
Marker27656
Marker33810
Marker10419
Marker25434
Marker73757
Marker71852
Marker4015
Marker71874
Marker7030
Marker41368
Marker29714
Marker6138
Marker58813
Marker2317
Marker9887
Marker29935
Marker33359
Marker57685
Marker42274
Marker17401
Marker24153
Marker20265
Marker72714
Marker22697
Marker7019
Marker37187
Marker19186
Marker40080
Marker21855
Marker26464
Marker8274
Marker71222
Marker36067
Marker18786
Marker25990
Marker34070
Marker58445
Marker32786
Marker72305
Marker14636
Marker65586
Marker24958
Marker49863
Marker67436
Marker73521
Marker46894
Marker30158
Marker75483
Marker25064
Marker59346
Marker70106
Marker74761
Marker49384
Marker54419
Marker21853
Marker29108
Marker39811
Marker37885
Marker68328
Marker49191
Marker55031
Marker63181
Marker17047
Marker15348
Marker23113
Marker38176
Marker12977
Marker25588
Marker46633
Marker44203
Marker19511
Marker36405
Marker73907
Marker15368
Marker32922
Marker11683
Marker35813
Marker38987
Marker26252
Marker51468
Marker16530
Marker51978
Marker50049
Marker68152
Marker67497
Marker19965
Marker21564
Marker40096
Marker15134
Marker23426
Marker20568
Marker24934
Marker25315
Marker11266
Marker37296
Marker16813
Marker22222
Marker43940
Marker63279
Marker2309
Marker28087
Marker18819
Marker18649
Marker35807
Marker75981
Marker67536
Marker1314
Marker23896

LG6

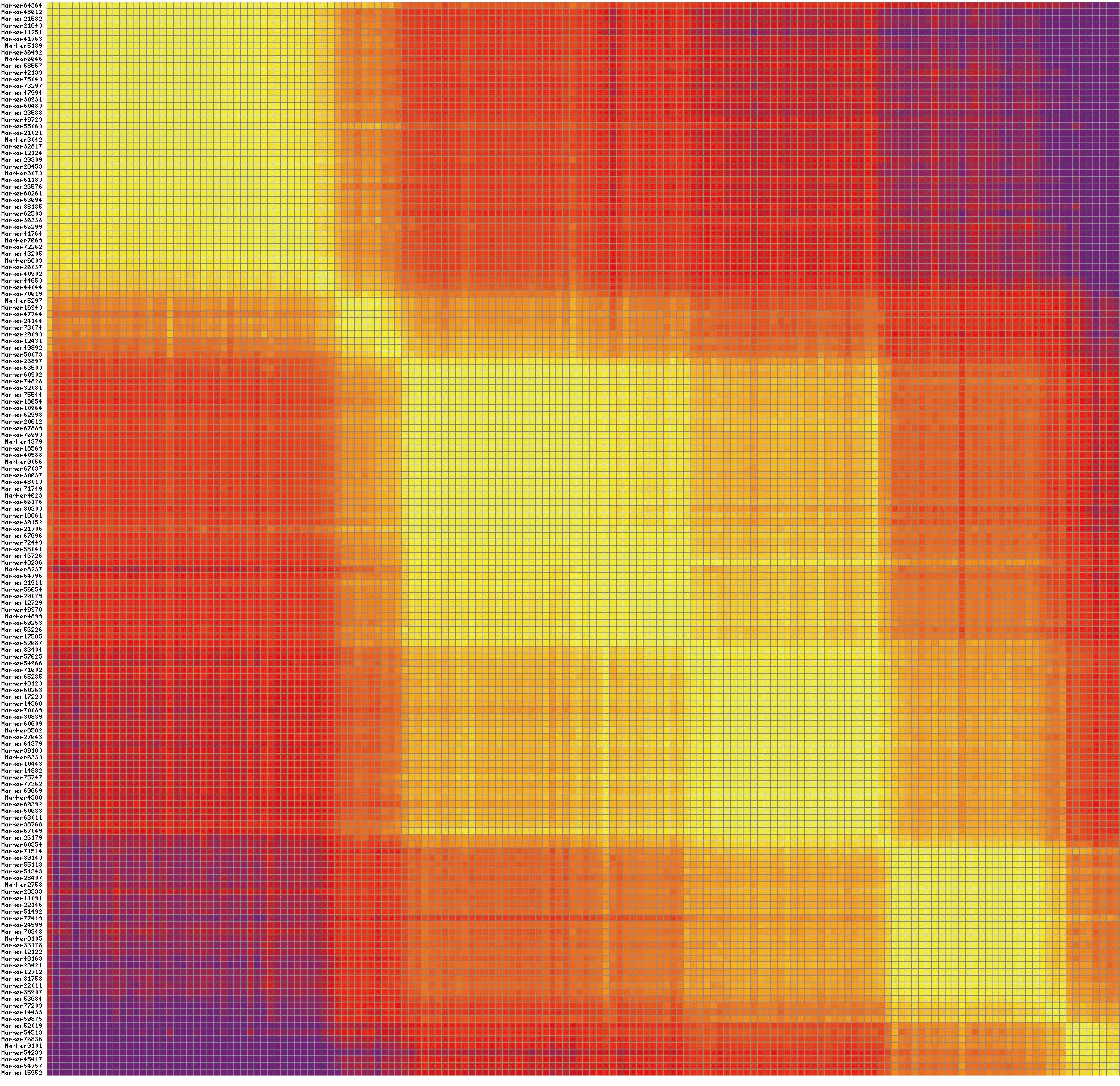

LG7

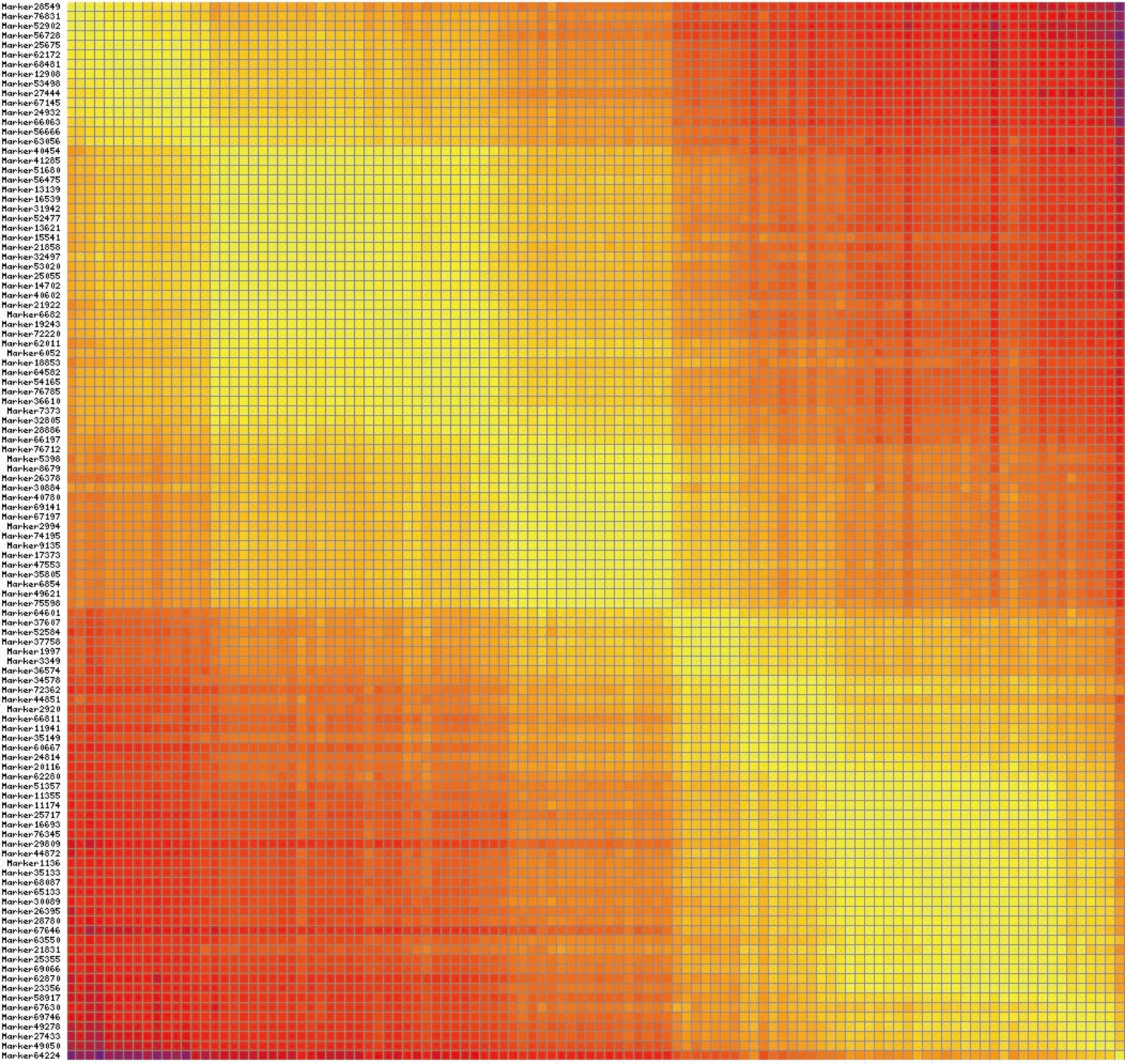
Marker28549
Marker76831
Marker52902
Marker56728
Marker25675
Marker62172
Marker68481
Marker12908
Marker53498
Marker27444
Marker67145
Marker24932
Marker66063
Marker56666
Marker63056
Marker40454
Marker41285
Marker51680
Marker56475
Marker13139
Marker16539
Marker31942
Marker52477
Marker13621
Marker15541
Marker21858
Marker32497
Marker53020
Marker25055
Marker14702
Marker40602
Marker21922
Marker6682
Marker19243
Marker72220
Marker62011
Marker6052
Marker18853
Marker64582
Marker54165
Marker76785
Marker36610
Marker7373
Marker32805
Marker28886
Marker66197
Marker76712
Marker5398
Marker8679
Marker26378
Marker30884
Marker40780
Marker69141
Marker67197
Marker2994
Marker74195
Marker9135
Marker17373
Marker47553
Marker35805
Marker6854
Marker49621
Marker75598
Marker64601
Marker37607
Marker52584
Marker37758
Marker1997
Marker3349
Marker36574
Marker34578
Marker72362
Marker44851
Marker2920
Marker66811
Marker11941
Marker35149
Marker60667
Marker24814
Marker20116
Marker62280
Marker51357
Marker11355
Marker11174
Marker25717
Marker16693
Marker76345
Marker29809
Marker44872
Marker1136
Marker35133
Marker68087
Marker65133
Marker30089
Marker26395
Marker28780
Marker67646
Marker63550
Marker21831
Marker25355
Marker69066
Marker62870
Marker23356
Marker58917
Marker67630
Marker69746
Marker49278
Marker27433
Marker49050
Marker64224

LG8

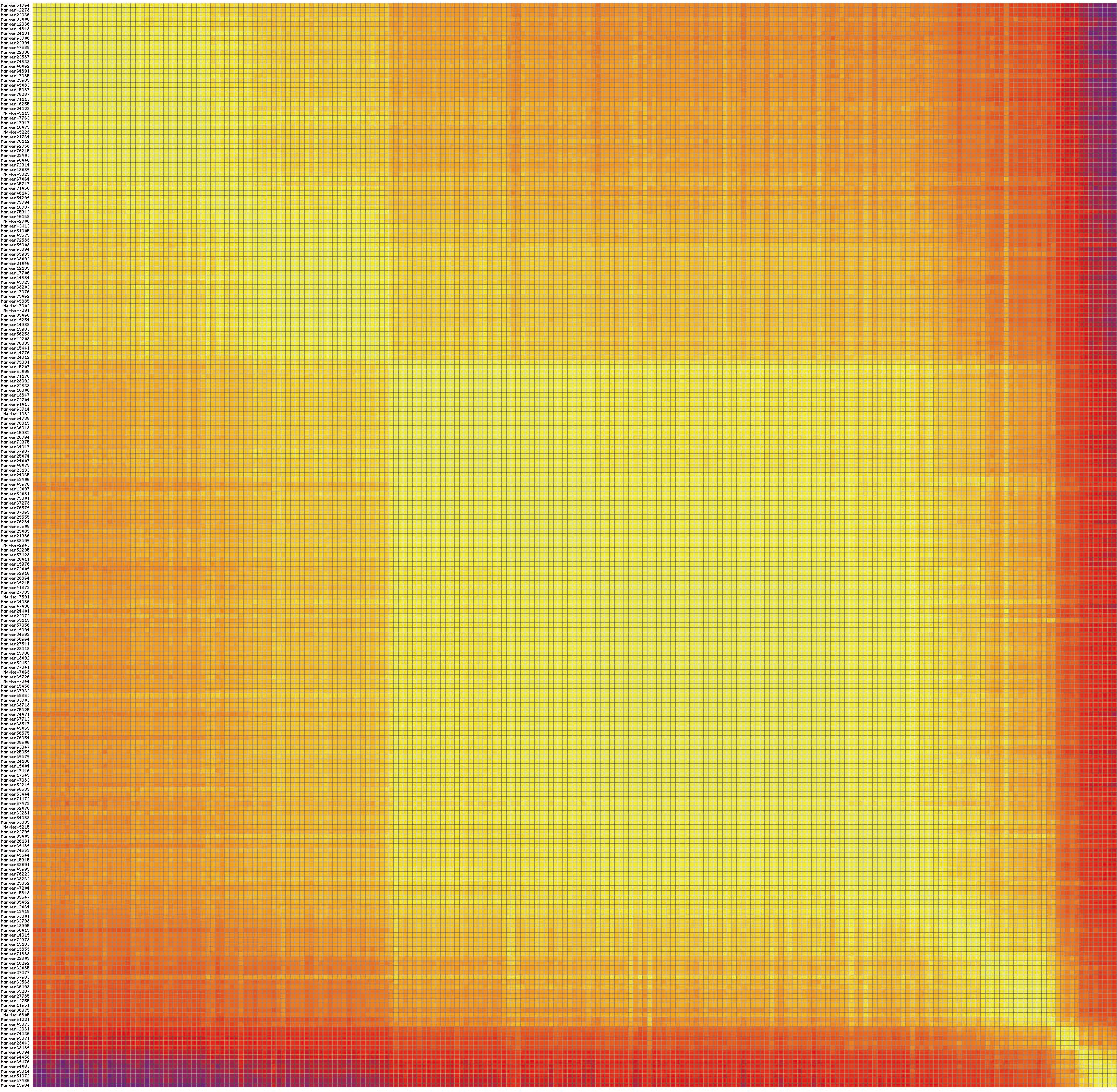

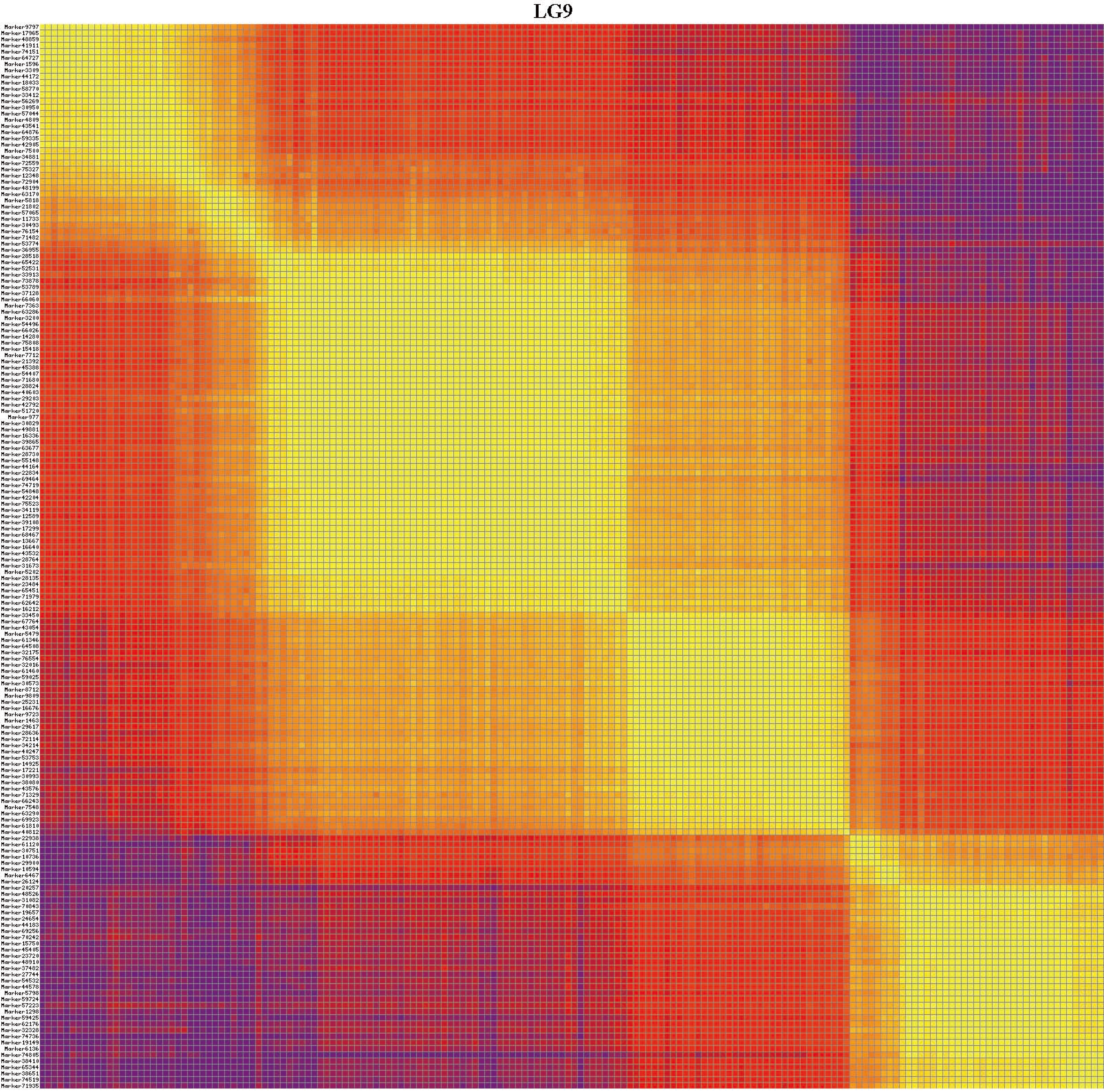
LG9

LG10

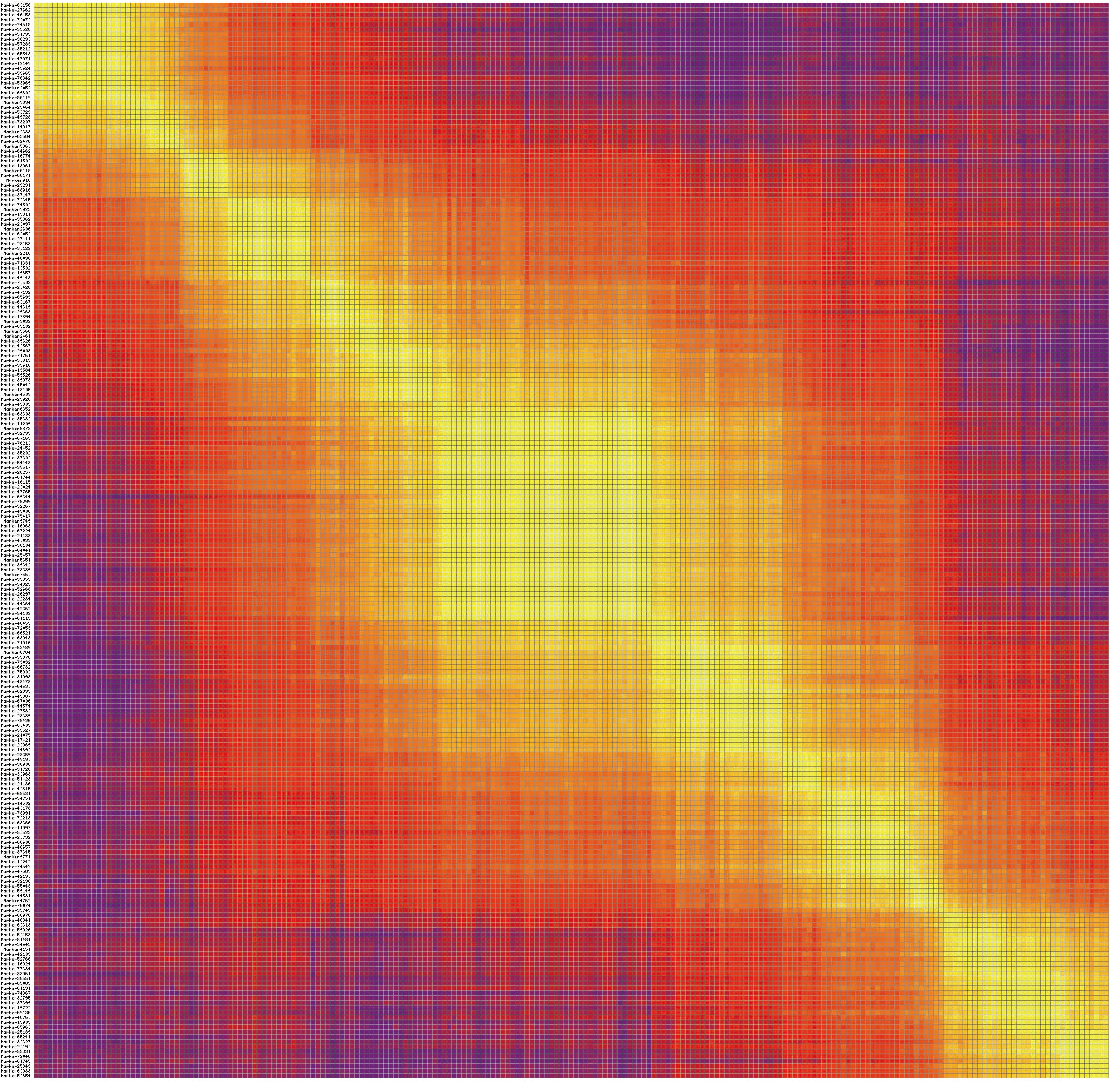

LG11

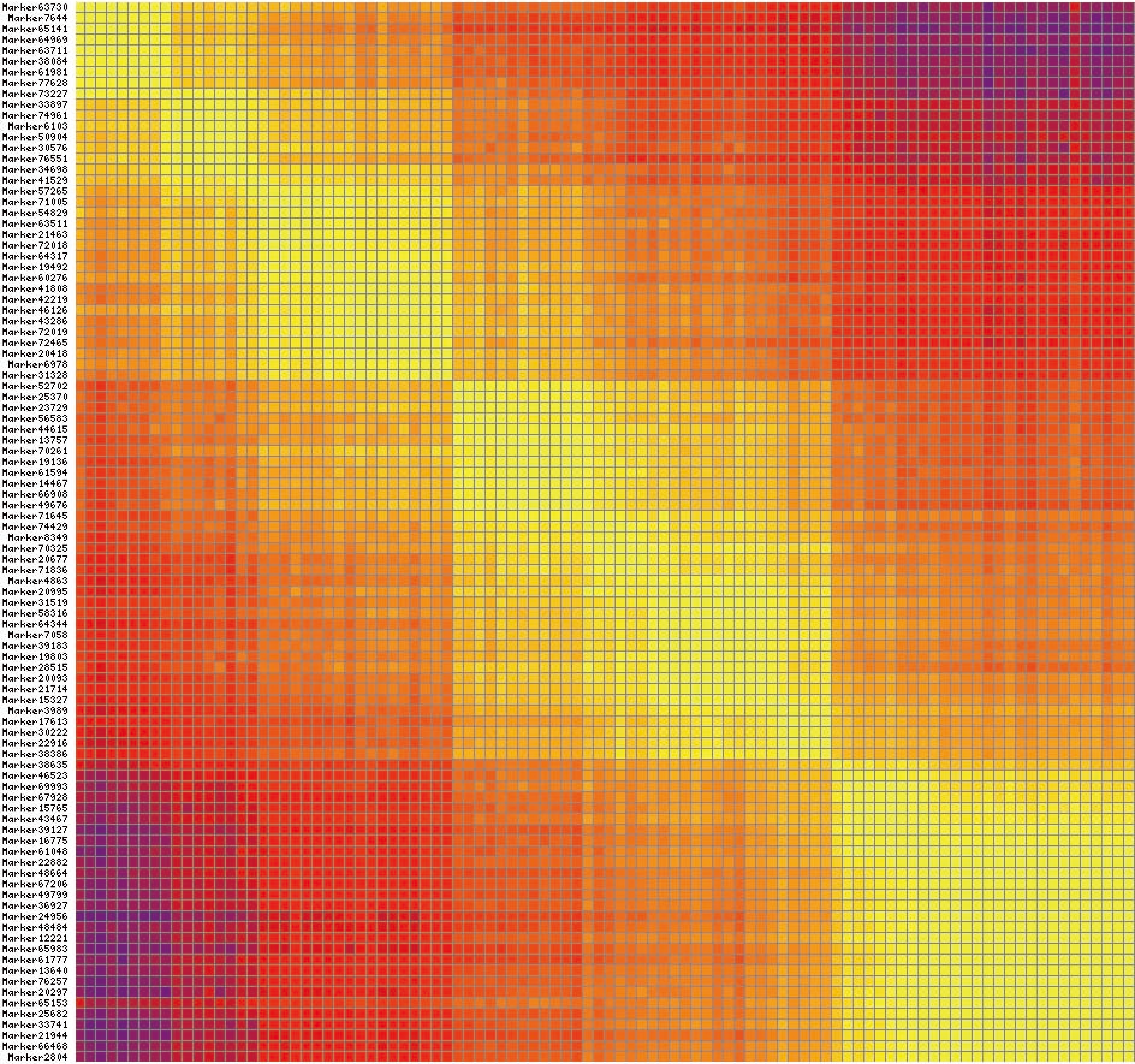
Marker63730
Marker7644
Marker65141
Marker64969
Marker63711
Marker38084
Marker61981
Marker77628
Marker73227
Marker33897
Marker74961
Marker6103
Marker50904
Marker30576
Marker76551
Marker34698
Marker41529
Marker57265
Marker71005
Marker54829
Marker63511
Marker21463
Marker72018
Marker64317
Marker19492
Marker60276
Marker41808
Marker42219
Marker46126
Marker43286
Marker72019
Marker72465
Marker20418
Marker6978
Marker31328
Marker52702
Marker25370
Marker23729
Marker56583
Marker44615
Marker13757
Marker70261
Marker19136
Marker61594
Marker14467
Marker66908
Marker49676
Marker71645
Marker74429
Marker8349
Marker70325
Marker20677
Marker71836
Marker4863
Marker20995
Marker31519
Marker58316
Marker64344
Marker7058
Marker39183
Marker19803
Marker28515
Marker20093
Marker21714
Marker15327
Marker3989
Marker17613
Marker30222
Marker22916
Marker38386
Marker38635
Marker46523
Marker69993
Marker67928
Marker15765
Marker43467
Marker39127
Marker16775
Marker61048
Marker22882
Marker48664
Marker67206
Marker49799
Marker36927
Marker24956
Marker48484
Marker12221
Marker65983
Marker61777
Marker13640
Marker76257
Marker20297
Marker65153
Marker25682
Marker33741
Marker21944
Marker66468
Marker2804

LG12

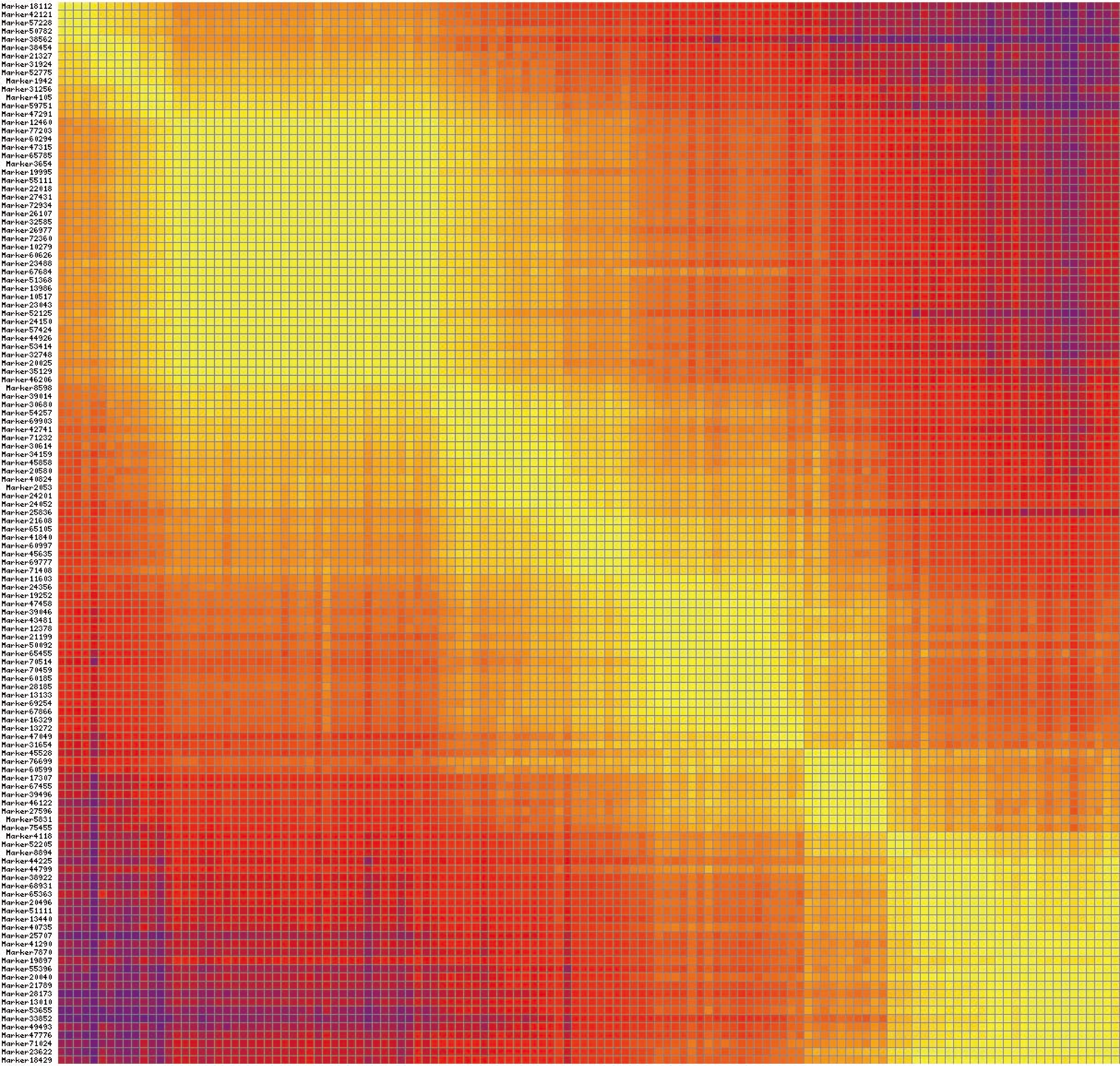

Marker18112
Marker42121
Marker57228
Marker50782
Marker38562
Marker38454
Marker21327
Marker31924
Marker52775
Marker1942
Marker31256
Marker4105
Marker59751
Marker47291
Marker12460
Marker77203
Marker60294
Marker47315
Marker65785
Marker3654
Marker19995
Marker55111
Marker22018
Marker27431
Marker72934
Marker26107
Marker32585
Marker26977
Marker72360
Marker10279
Marker60626
Marker23488
Marker67684
Marker51368
Marker13986
Marker10517
Marker23043
Marker52125
Marker24150
Marker57424
Marker44926
Marker53414
Marker32748
Marker20025
Marker35129
Marker46206
Marker8598
Marker39014
Marker30680
Marker54257
Marker69903
Marker42741
Marker71232
Marker30614
Marker34159
Marker45858
Marker20580
Marker40824
Marker2053
Marker24201
Marker24052
Marker25836
Marker21608
Marker65105
Marker41840
Marker60997
Marker45635
Marker69777
Marker71408
Marker11603
Marker24356
Marker19252
Marker47458
Marker39046
Marker43481
Marker12378
Marker21199
Marker50092
Marker65455
Marker70514
Marker70459
Marker60185
Marker28185
Marker13133
Marker69254
Marker67866
Marker16329
Marker13272
Marker47049
Marker31654
Marker45528
Marker76699
Marker60599
Marker17307
Marker67455
Marker39496
Marker46122
Marker27596
Marker5831
Marker75455
Marker4118
Marker52205
Marker8894
Marker44225
Marker44799
Marker38922
Marker68931
Marker65363
Marker20496
Marker51111
Marker13440
Marker40735
Marker25707
Marker41290
Marker7870
Marker19897
Marker55396
Marker20040
Marker21789
Marker28173
Marker13010
Marker53655
Marker33852
Marker49493
Marker47776
Marker71024
Marker23622
Marker18429

LG13

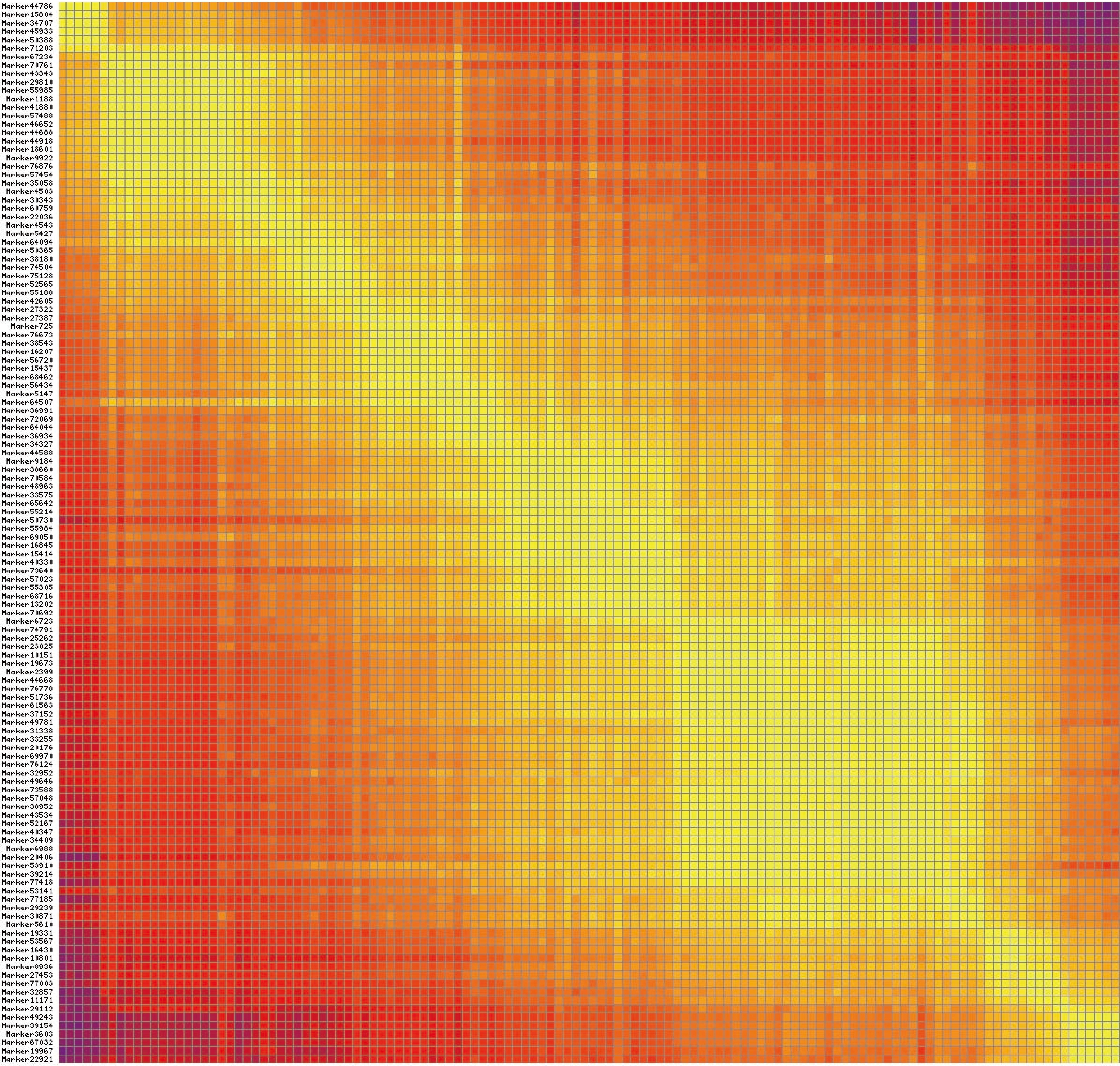
Marker44786
Marker15804
Marker34707
Marker45933
Marker50388
Marker71203
Marker67234
Marker70761
Marker43343
Marker29810
Marker55985
Marker1188
Marker41880
Marker57488
Marker46652
Marker44688
Marker44918
Marker18601
Marker9922
Marker76876
Marker57454
Marker35058
Marker4503
Marker30343
Marker60759
Marker22036
Marker4543
Marker5427
Marker64094
Marker50365
Marker38180
Marker74504
Marker75128
Marker52565
Marker55188
Marker42605
Marker27322
Marker27387
Marker725
Marker76673
Marker38543
Marker16207
Marker56720
Marker15437
Marker68462
Marker56434
Marker5147
Marker64507
Marker36991
Marker72069
Marker64044
Marker36934
Marker34327
Marker44588
Marker9184
Marker38660
Marker70584
Marker48963
Marker33575
Marker65642
Marker55214
Marker50730
Marker55984
Marker69050
Marker16845
Marker15414
Marker40330
Marker73640
Marker57023
Marker55305
Marker68716
Marker13202
Marker70692
Marker6723
Marker74791
Marker25262
Marker23025
Marker10151
Marker19673
Marker2399
Marker44668
Marker76778
Marker51736
Marker61563
Marker37152
Marker49781
Marker31338
Marker33255
Marker20176
Marker69970
Marker76124
Marker32952
Marker49646
Marker73588
Marker57048
Marker38952
Marker43534
Marker52167
Marker40347
Marker34409
Marker6988
Marker20406
Marker53910
Marker39214
Marker77418
Marker53141
Marker77185
Marker29239
Marker30871
Marker5610
Marker19331
Marker53567
Marker16430
Marker10801
Marker8936
Marker27453
Marker77003
Marker32857
Marker11171
Marker29112
Marker49243
Marker39154
Marker3603
Marker67032
Marker19967
Marker22921

LG14

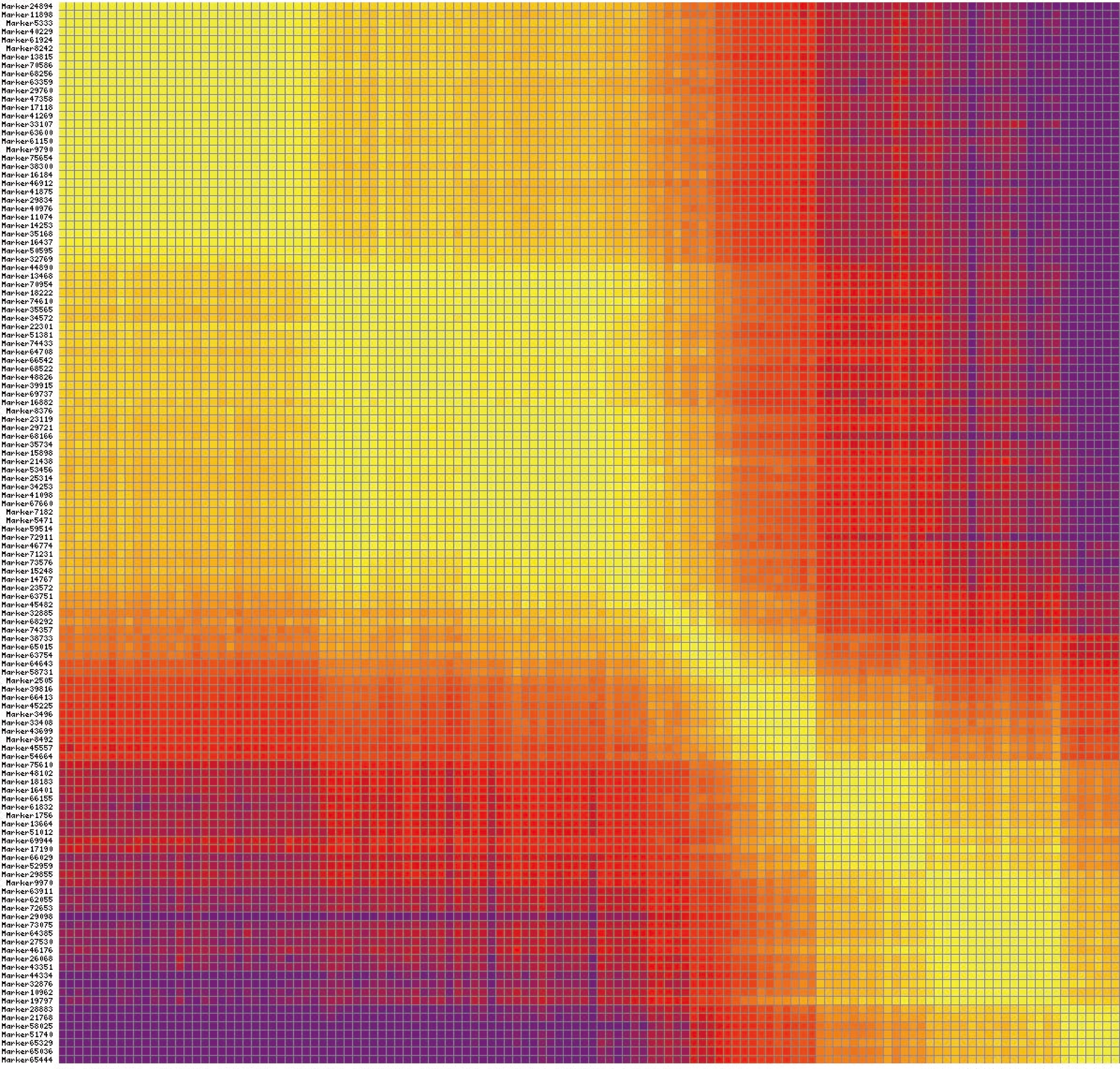

Marker24894
Marker11898
Marker5333
Marker40229
Marker61924
Marker8242
Marker13815
Marker70586
Marker68256
Marker63359
Marker29760
Marker47358
Marker17118
Marker41269
Marker33107
Marker63600
Marker61150
Marker9790
Marker75654
Marker38300
Marker16184
Marker46912
Marker41875
Marker29834
Marker40976
Marker11074
Marker14253
Marker35168
Marker16437
Marker50595
Marker32769
Marker44890
Marker13468
Marker70954
Marker18222
Marker74610
Marker35565
Marker34572
Marker22301
Marker51381
Marker74433
Marker64708
Marker66542
Marker68522
Marker48826
Marker39915
Marker69737
Marker16882
Marker8376
Marker23119
Marker29721
Marker68166
Marker35734
Marker15898
Marker21438
Marker53456
Marker25314
Marker34253
Marker41098
Marker67660
Marker7182
Marker5471
Marker59514
Marker72911
Marker46774
Marker71231
Marker73576
Marker15248
Marker14767
Marker23572
Marker63751
Marker45482
Marker32885
Marker68292
Marker74357
Marker38733
Marker65015
Marker63754
Marker64643
Marker58731
Marker2505
Marker39816
Marker66413
Marker45225
Marker3496
Marker33408
Marker43699
Marker8492
Marker45557
Marker54664
Marker75610
Marker48102
Marker18183
Marker16401
Marker66155
Marker61832
Marker1756
Marker13664
Marker51012
Marker69944
Marker17190
Marker66029
Marker52959
Marker29855
Marker9970
Marker63911
Marker62055
Marker72653
Marker29098
Marker73075
Marker64385
Marker27530
Marker46176
Marker26068
Marker43351
Marker44334
Marker32876
Marker10962
Marker19797
Marker28883
Marker21768
Marker58025
Marker51740
Marker65329
Marker65036
Marker65444

LG15

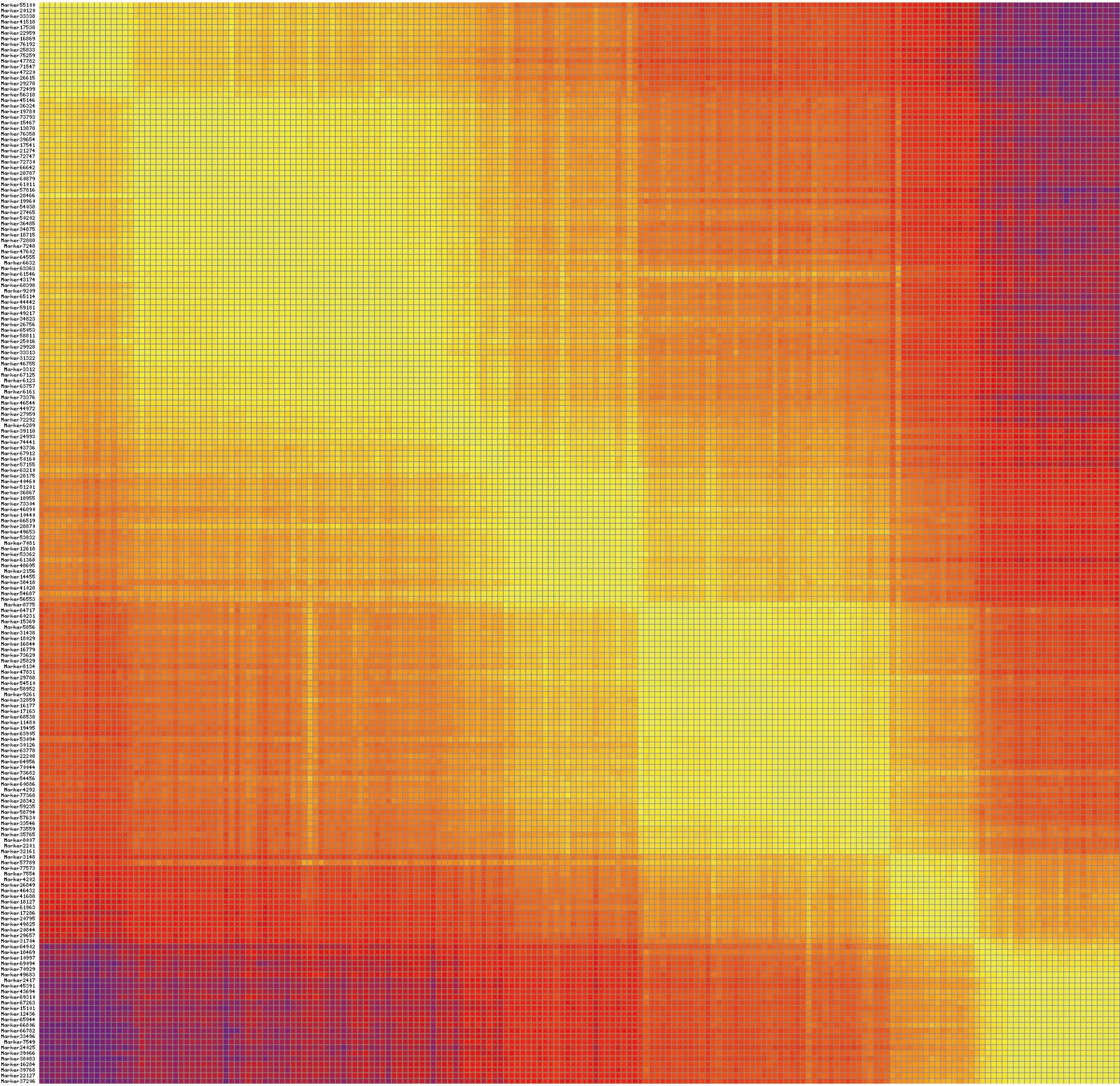

LG16

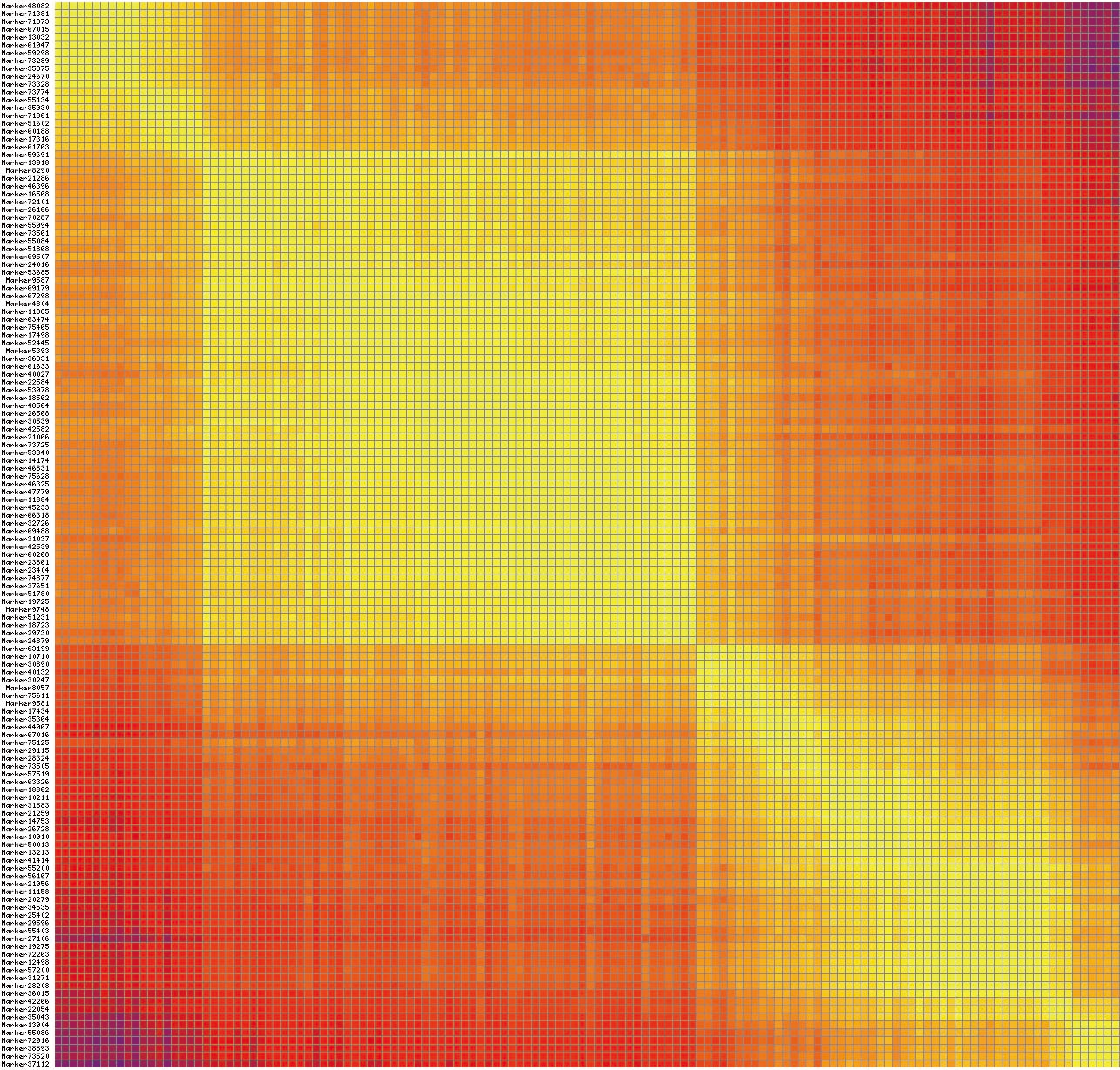
Marker48082
Marker71381
Marker71873
Marker67015
Marker13032
Marker61947
Marker59298
Marker73289
Marker35375
Marker24670
Marker73328
Marker73774
Marker55134
Marker35930
Marker71861
Marker51602
Marker60188
Marker17316
Marker61763
Marker59691
Marker13918
Marker8290
Marker21286
Marker46396
Marker16568
Marker72101
Marker26166
Marker70287
Marker55994
Marker73561
Marker55084
Marker51868
Marker69507
Marker24016
Marker53685
Marker9587
Marker69179
Marker67298
Marker4804
Marker11885
Marker63474
Marker75465
Marker17498
Marker52445
Marker5393
Marker36331
Marker61633
Marker40027
Marker22584
Marker53978
Marker18562
Marker48564
Marker26568
Marker30539
Marker42582
Marker21066
Marker73725
Marker53340
Marker14174
Marker46831
Marker75628
Marker46325
Marker47779
Marker11884
Marker45233
Marker66318
Marker32726
Marker69488
Marker31037
Marker42539
Marker60268
Marker23861
Marker23404
Marker74877
Marker37651
Marker51780
Marker19725
Marker9748
Marker51231
Marker18723
Marker29730
Marker24879
Marker63199
Marker10710
Marker30890
Marker40132
Marker30247
Marker8057
Marker75611
Marker9581
Marker17434
Marker35364
Marker44967
Marker67016
Marker75125
Marker29115
Marker28324
Marker73505
Marker57519
Marker63326
Marker18862
Marker10211
Marker31583
Marker21259
Marker14753
Marker26728
Marker10910
Marker50013
Marker13213
Marker41414
Marker55200
Marker56167
Marker21956
Marker11158
Marker20279
Marker34535
Marker25402
Marker29596
Marker55403
Marker27106
Marker19275
Marker72263
Marker12498
Marker57200
Marker31271
Marker28208
Marker36015
Marker42266
Marker22054
Marker35043
Marker13904
Marker55086
Marker72916
Marker38593
Marker73520
Marker37112

LG17

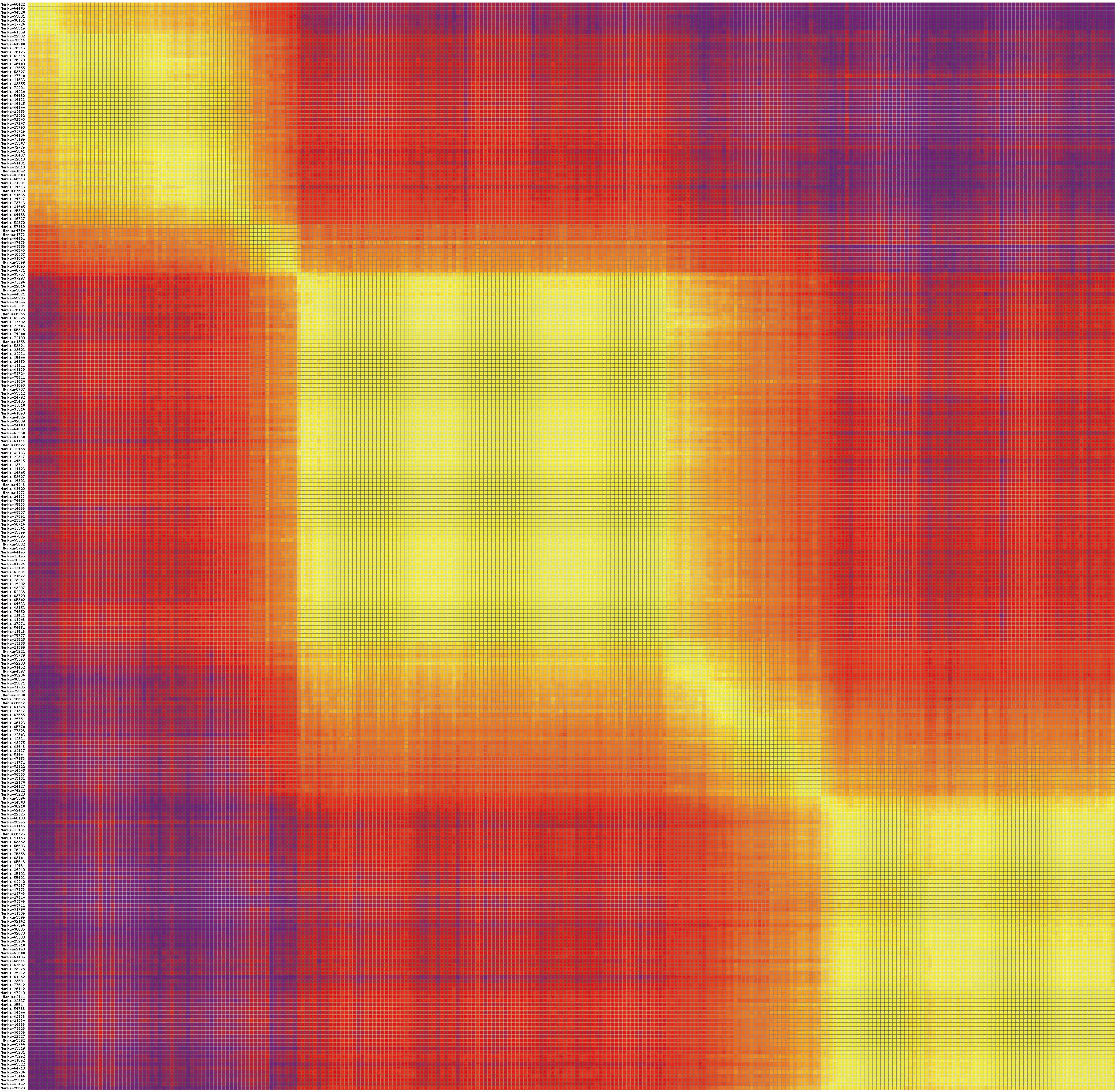

LG18

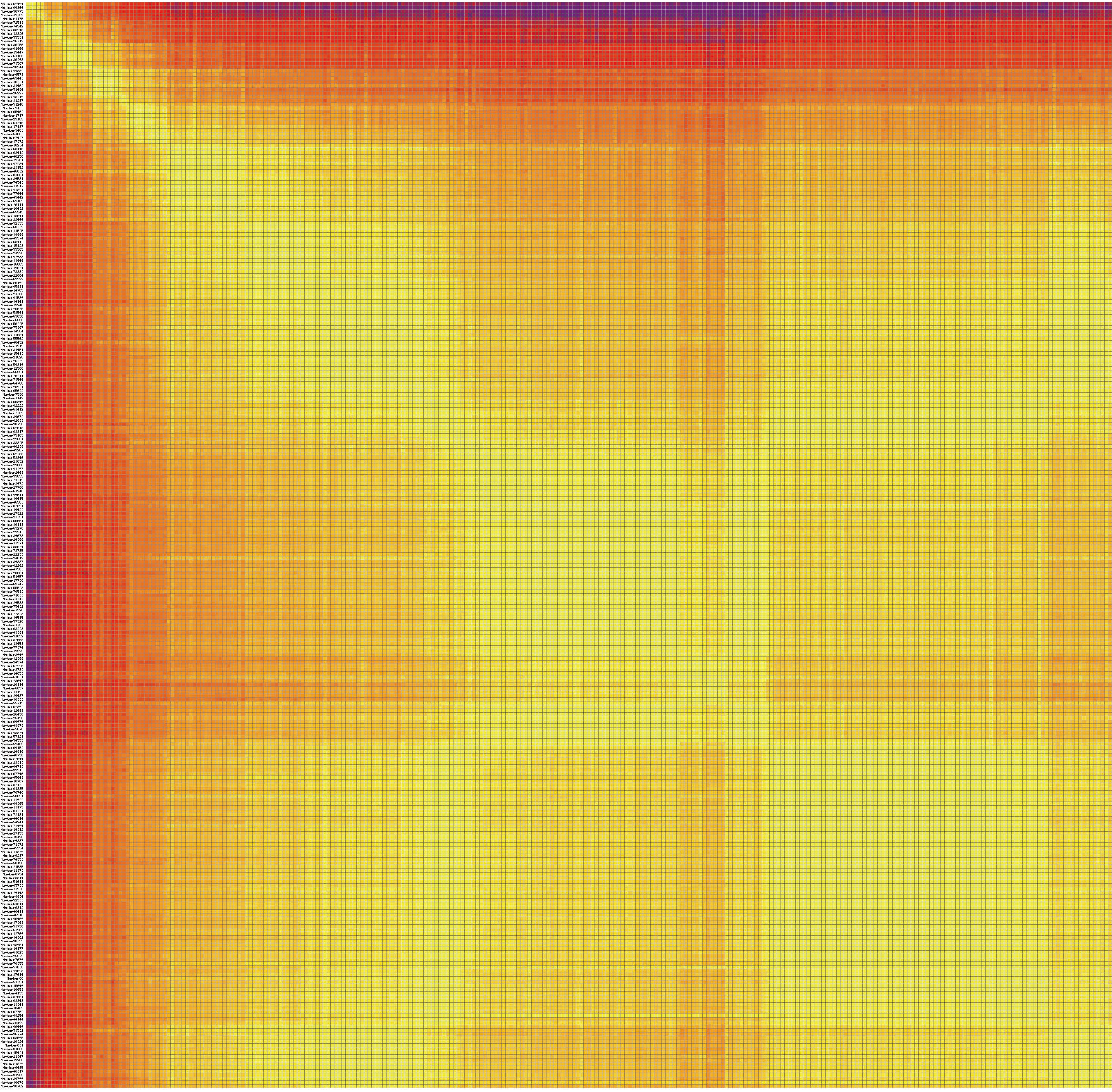

LG19

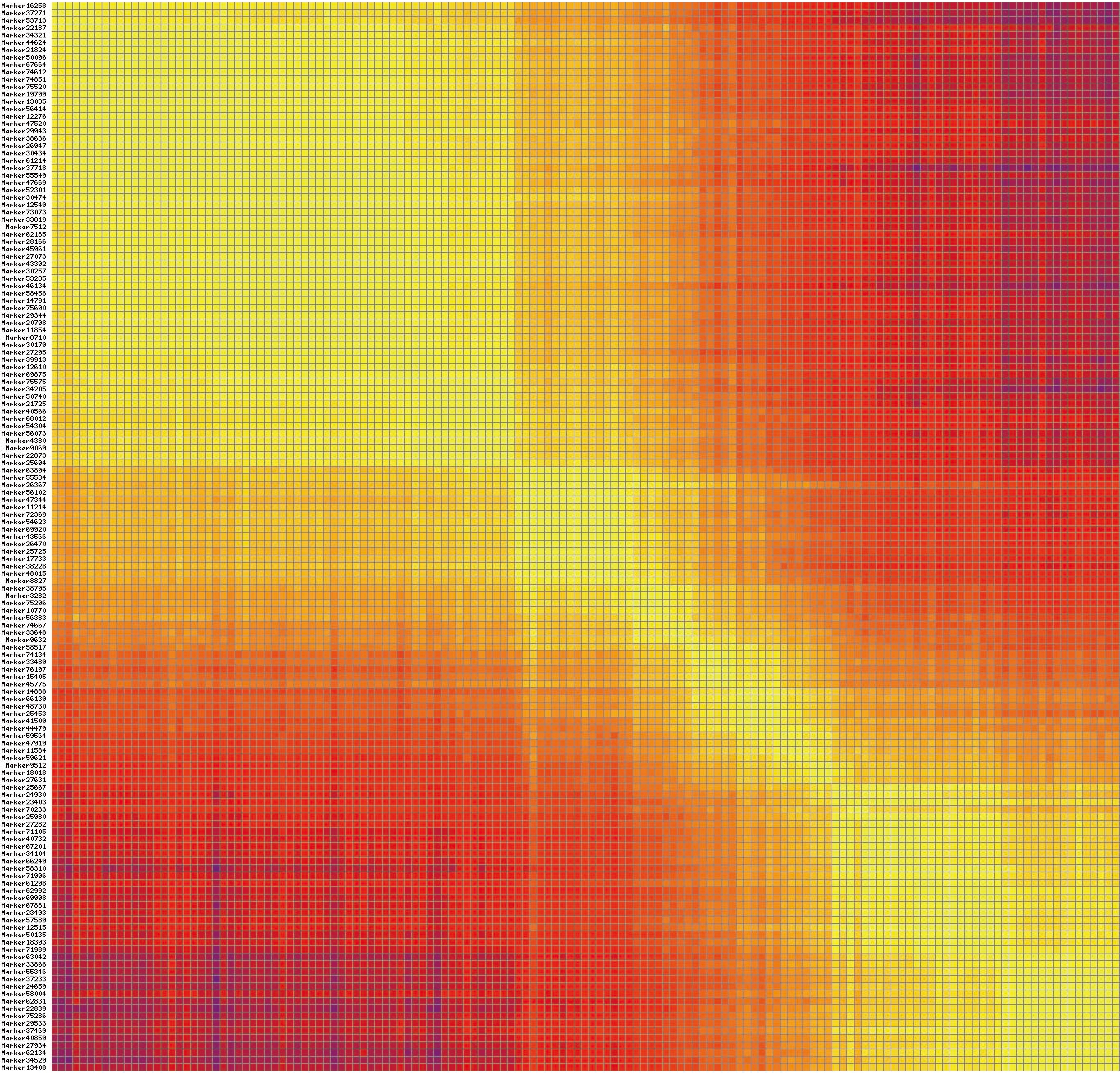

Marker16258
Marker37271
Marker53713
Marker22187
Marker34321
Marker44624
Marker21824
Marker50096
Marker67664
Marker74612
Marker74851
Marker75520
Marker19799
Marker13035
Marker56414
Marker12276
Marker47520
Marker29943
Marker38636
Marker26947
Marker30434
Marker61214
Marker37718
Marker55549
Marker47669
Marker52301
Marker30474
Marker12549
Marker73073
Marker33819
Marker7512
Marker62185
Marker28166
Marker45961
Marker27073
Marker43392
Marker30257
Marker53285
Marker46134
Marker58458
Marker14791
Marker75690
Marker29344
Marker20798
Marker11854
Marker8710
Marker30179
Marker27295
Marker39913
Marker12610
Marker69875
Marker75575
Marker34205
Marker50740
Marker21725
Marker40566
Marker68012
Marker54304
Marker56073
Marker4380
Marker9069
Marker22873
Marker25694
Marker63894
Marker55534
Marker26367
Marker56102
Marker47344
Marker11214
Marker72369
Marker54623
Marker69920
Marker43566
Marker26470
Marker25725
Marker17733
Marker38228
Marker48015
Marker8827
Marker38795
Marker3282
Marker75296
Marker10770
Marker56383
Marker74667
Marker33648
Marker9632
Marker58517
Marker74134
Marker33489
Marker76197
Marker15405
Marker45775
Marker14888
Marker66139
Marker48730
Marker25453
Marker41509
Marker44479
Marker59564
Marker47919
Marker11584
Marker59621
Marker9512
Marker18018
Marker27631
Marker25667
Marker24930
Marker23403
Marker70233
Marker25980
Marker27282
Marker71105
Marker40732
Marker67201
Marker34104
Marker66249
Marker58310
Marker71996
Marker61298
Marker62992
Marker69998
Marker67881
Marker23493
Marker57589
Marker12515
Marker50135
Marker18393
Marker71989
Marker63042
Marker33868
Marker55346
Marker37233
Marker24659
Marker58004
Marker62831
Marker22839
Marker75286
Marker29533
Marker37469
Marker40859
Marker27934
Marker62134
Marker34529
Marker13408

LG20

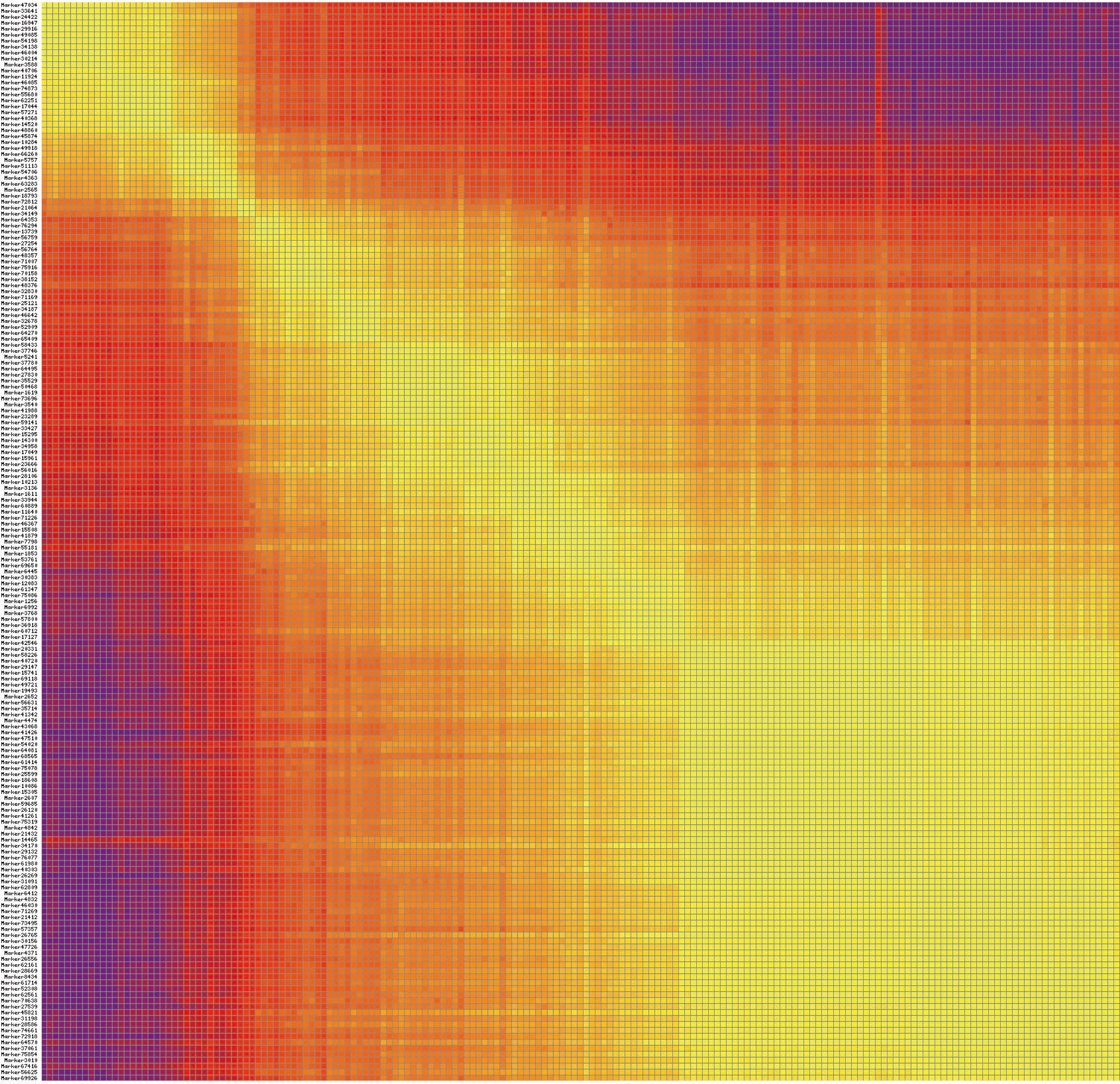

LG21

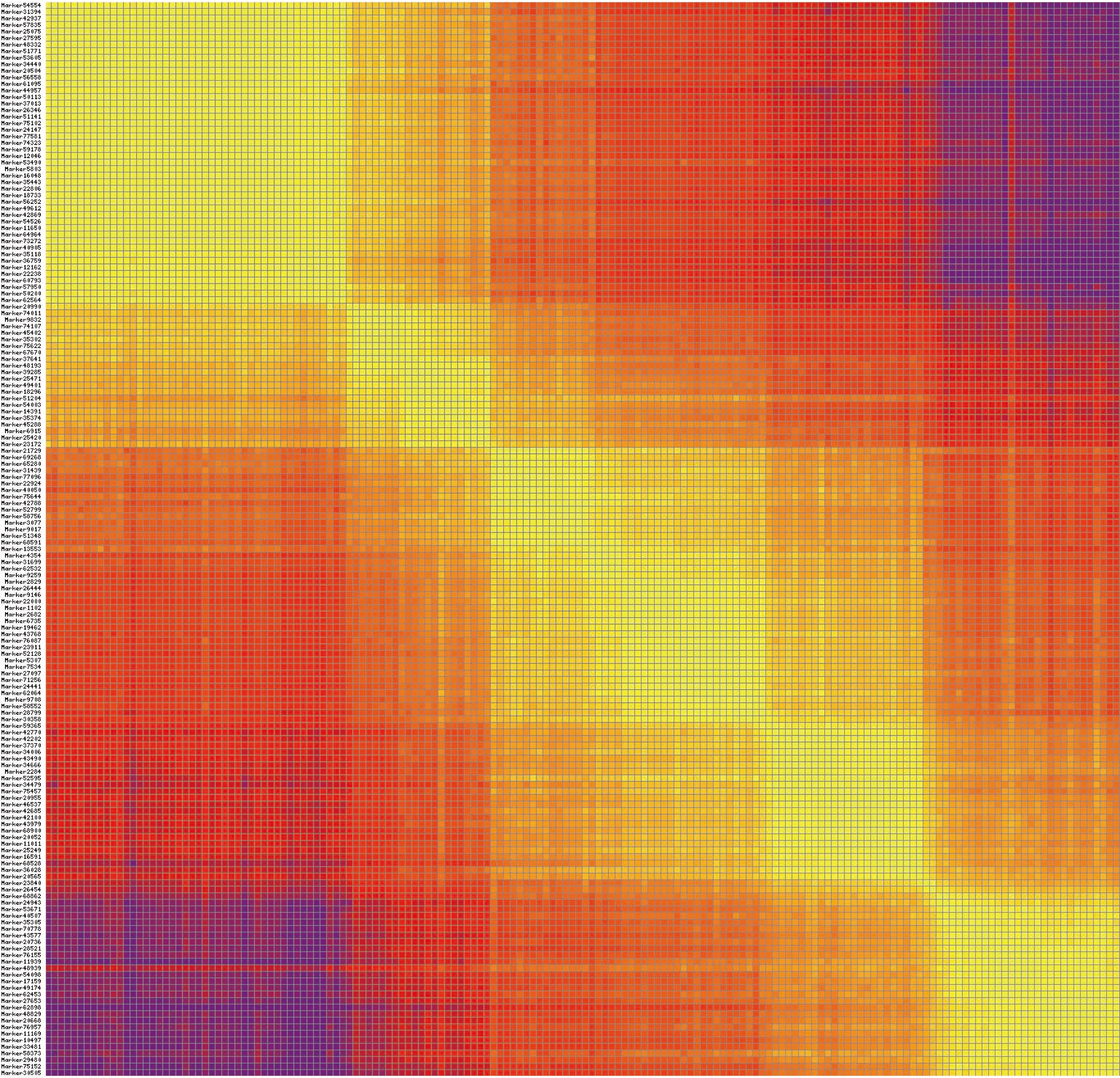

LG22

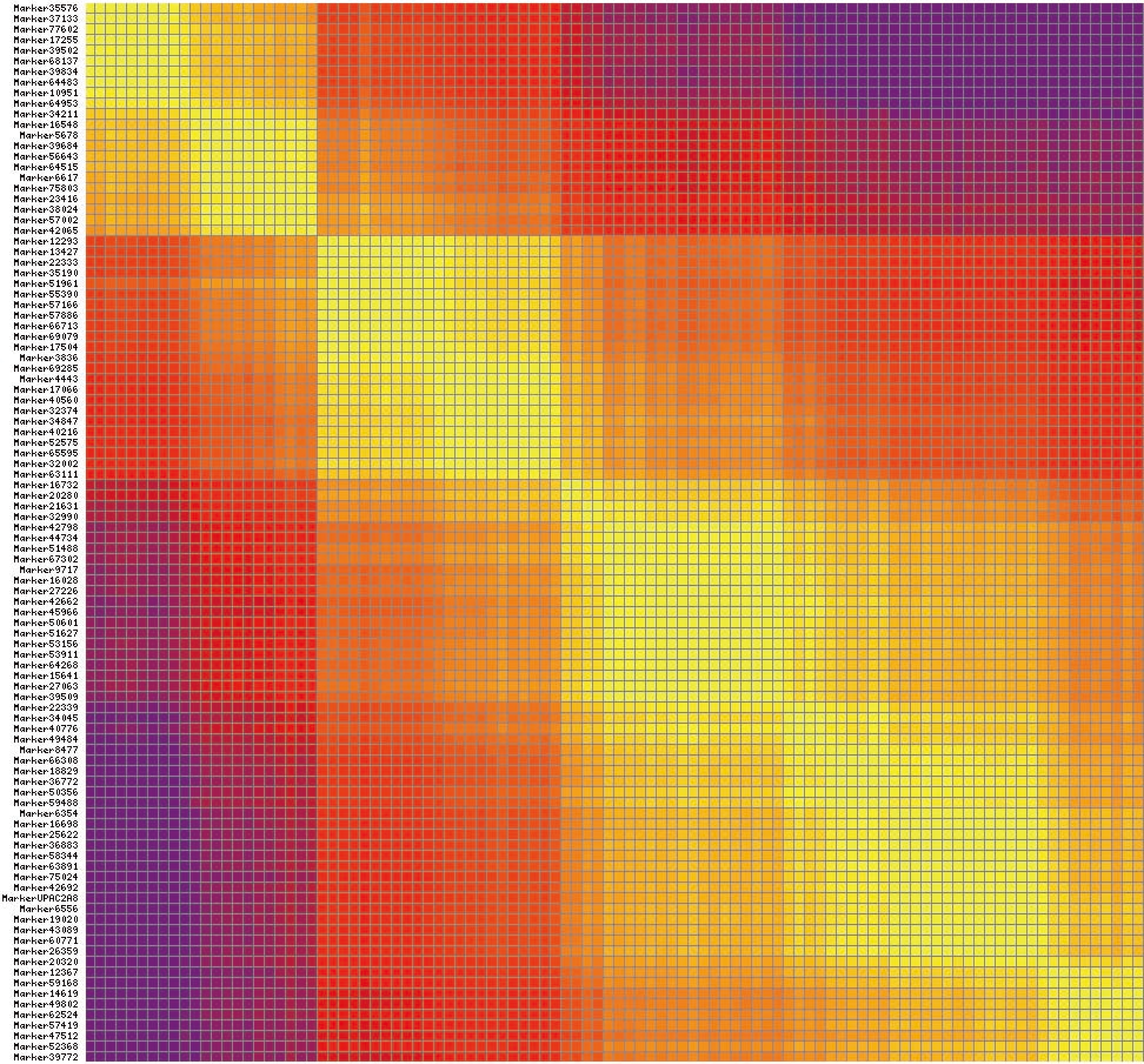
Marker35576
Marker37133
Marker77602
Marker17255
Marker39502
Marker68137
Marker39834
Marker64483
Marker10951
Marker64953
Marker34211
Marker16548
Marker5678
Marker39684
Marker56643
Marker64515
Marker6617
Marker75803
Marker23416
Marker38024
Marker57002
Marker42065
Marker12293
Marker13427
Marker22333
Marker35190
Marker51961
Marker55390
Marker57166
Marker57886
Marker66713
Marker69079
Marker17504
Marker3836
Marker69285
Marker4443
Marker17066
Marker40560
Marker32374
Marker34847
Marker40216
Marker52575
Marker65595
Marker32002
Marker63111
Marker16732
Marker20280
Marker21631
Marker32990
Marker42798
Marker44734
Marker51488
Marker67302
Marker9717
Marker16028
Marker27226
Marker42662
Marker45966
Marker50601
Marker51627
Marker53156
Marker53911
Marker64268
Marker15641
Marker27063
Marker39509
Marker22339
Marker34045
Marker40776
Marker49484
Marker8477
Marker66308
Marker18829
Marker36772
Marker50356
Marker59488
Marker6354
Marker16698
Marker25622
Marker36883
Marker58344
Marker63891
Marker75024
Marker42692
MarkerUPAC2A8
Marker6556
Marker19020
Marker43089
Marker60771
Marker26359
Marker20320
Marker12367
Marker59168
Marker14619
Marker49802
Marker62524
Marker57419
Marker47512
Marker52368
Marker39772

LG23

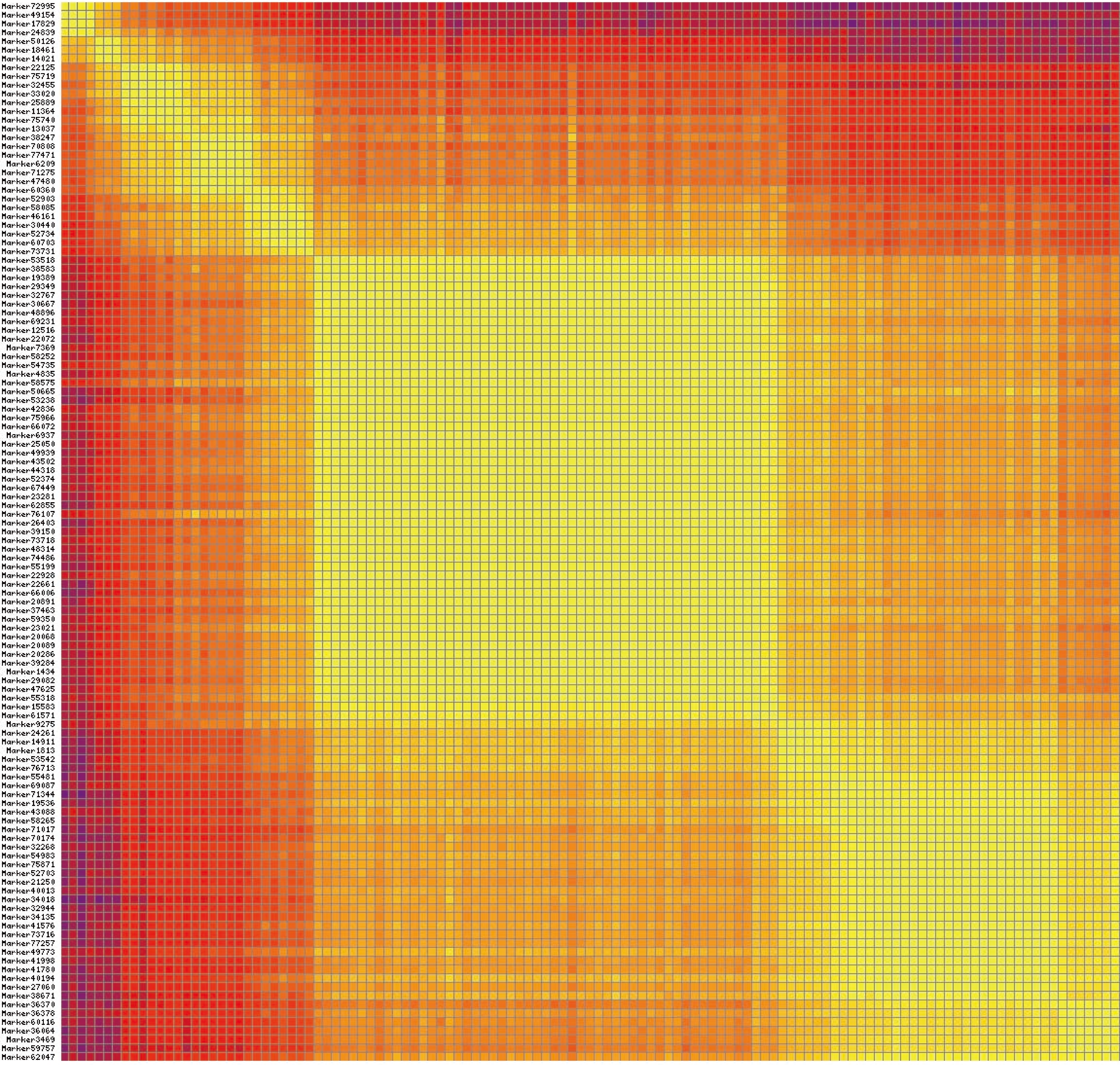
Marker72995
Marker49154
Marker17829
Marker24839
Marker50126
Marker18461
Marker14021
Marker22125
Marker75719
Marker32455
Marker33020
Marker25889
Marker11364
Marker75740
Marker13037
Marker38247
Marker70808
Marker77471
Marker6209
Marker71275
Marker47480
Marker60360
Marker52903
Marker58085
Marker46161
Marker30440
Marker52734
Marker60703
Marker73731
Marker53518
Marker38583
Marker19389
Marker29349
Marker32767
Marker30667
Marker48896
Marker69231
Marker12516
Marker22072
Marker7369
Marker58252
Marker54735
Marker4835
Marker58575
Marker50665
Marker53238
Marker42836
Marker75966
Marker66072
Marker6937
Marker25050
Marker49939
Marker43502
Marker44318
Marker52374
Marker67449
Marker23281
Marker62855
Marker76107
Marker26403
Marker39150
Marker73718
Marker48314
Marker74486
Marker55199
Marker22928
Marker22661
Marker66006
Marker20891
Marker37463
Marker59350
Marker23021
Marker20068
Marker20089
Marker20286
Marker39284
Marker1434
Marker29082
Marker47625
Marker55318
Marker15583
Marker61571
Marker9275
Marker24261
Marker14911
Marker1813
Marker53542
Marker76713
Marker55481
Marker69087
Marker71344
Marker19536
Marker43088
Marker58265
Marker71017
Marker70174
Marker32268
Marker54983
Marker75871
Marker52703
Marker21250
Marker40013
Marker34018
Marker32944
Marker34135
Marker41576
Marker73716
Marker77257
Marker49773
Marker41998
Marker41780
Marker40194
Marker27060
Marker38671
Marker36370
Marker36378
Marker60116
Marker36064
Marker3469
Marker59757
Marker62047

LG24

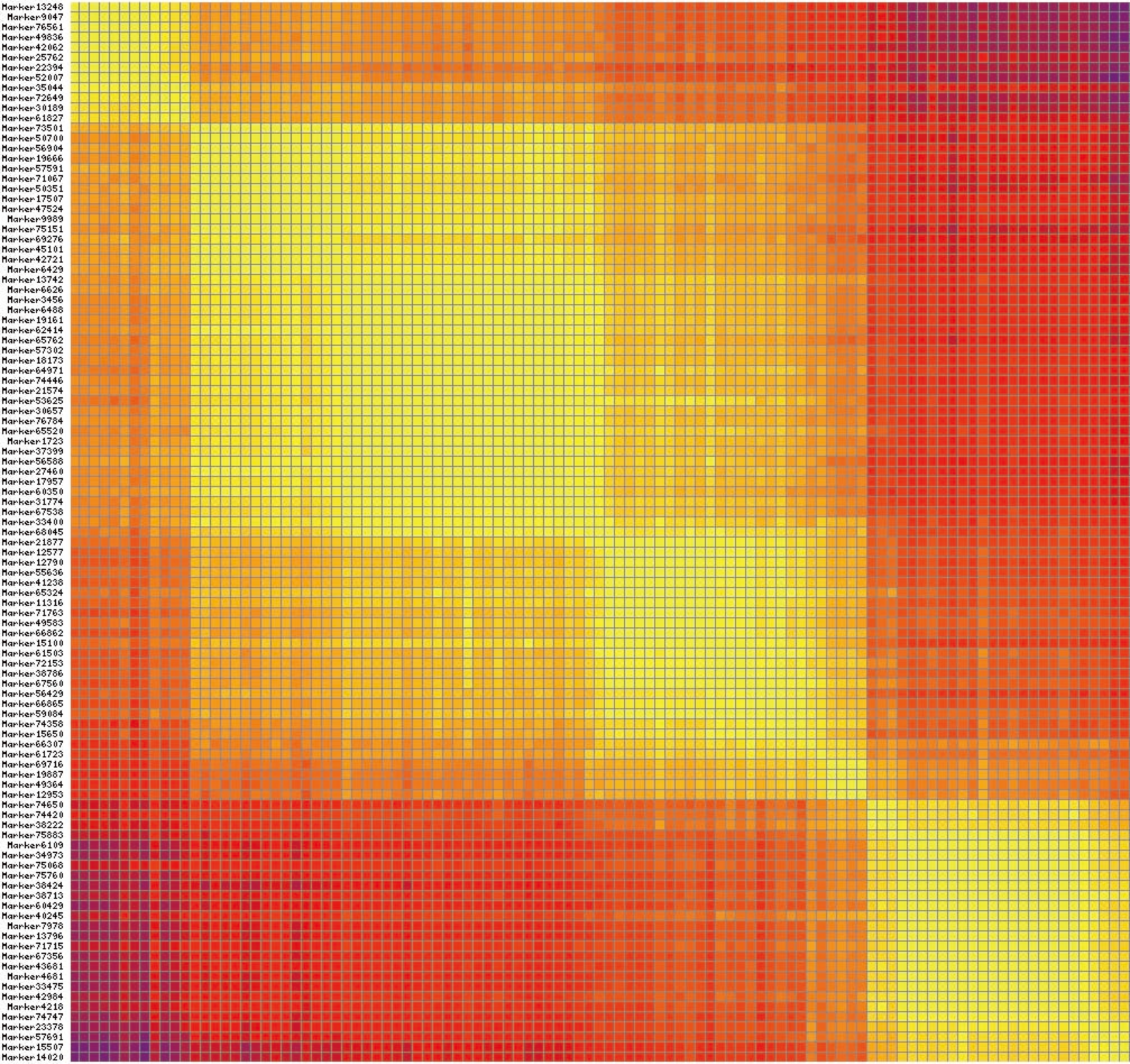
Marker13248
Marker9047
Marker76561
Marker49836
Marker42062
Marker25762
Marker22394
Marker52007
Marker35044
Marker72649
Marker30189
Marker61827
Marker73501
Marker50700
Marker56904
Marker19666
Marker57591
Marker71067
Marker50351
Marker17507
Marker47524
Marker9989
Marker75151
Marker69276
Marker45101
Marker42721
Marker6429
Marker13742
Marker6626
Marker3456
Marker6488
Marker19161
Marker62414
Marker65762
Marker57302
Marker18173
Marker64971
Marker74446
Marker21574
Marker53625
Marker30657
Marker76784
Marker65520
Marker1723
Marker37399
Marker56588
Marker27460
Marker17957
Marker60350
Marker31774
Marker67538
Marker33400
Marker68045
Marker21877
Marker12577
Marker12790
Marker55636
Marker41238
Marker65324
Marker11316
Marker71763
Marker49583
Marker66862
Marker15100
Marker61503
Marker72153
Marker38786
Marker67560
Marker56429
Marker66865
Marker59084
Marker74358
Marker15650
Marker66307
Marker61723
Marker69716
Marker19887
Marker49364
Marker12953
Marker74650
Marker74420
Marker38222
Marker75883
Marker6109
Marker34973
Marker75068
Marker75760
Marker38424
Marker38713
Marker60429
Marker40245
Marker7978
Marker13796
Marker71715
Marker67356
Marker43681
Marker4681
Marker33475
Marker42984
Marker4218
Marker74747
Marker23378
Marker57691
Marker15507
Marker14020

LG25

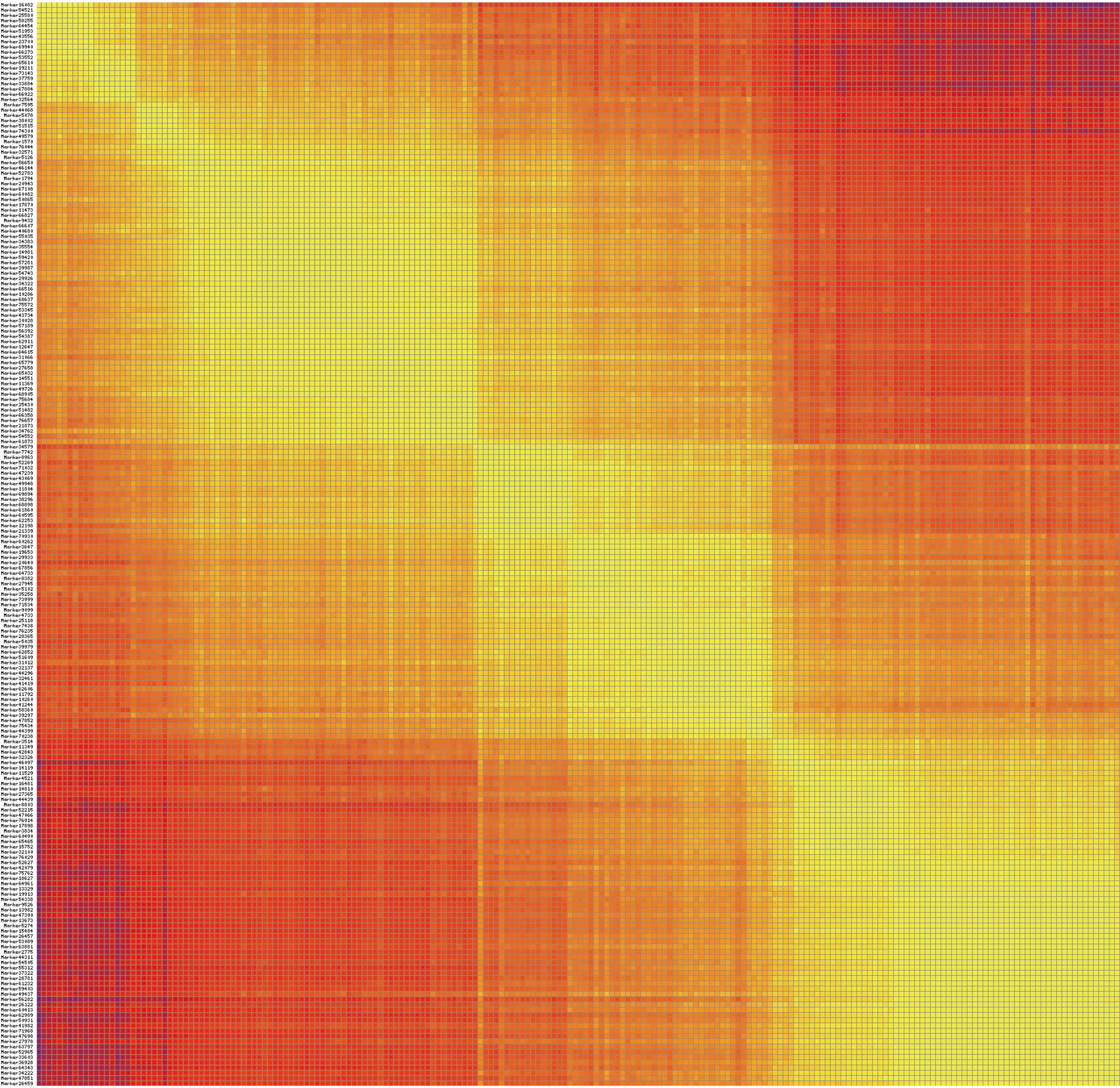

LG26

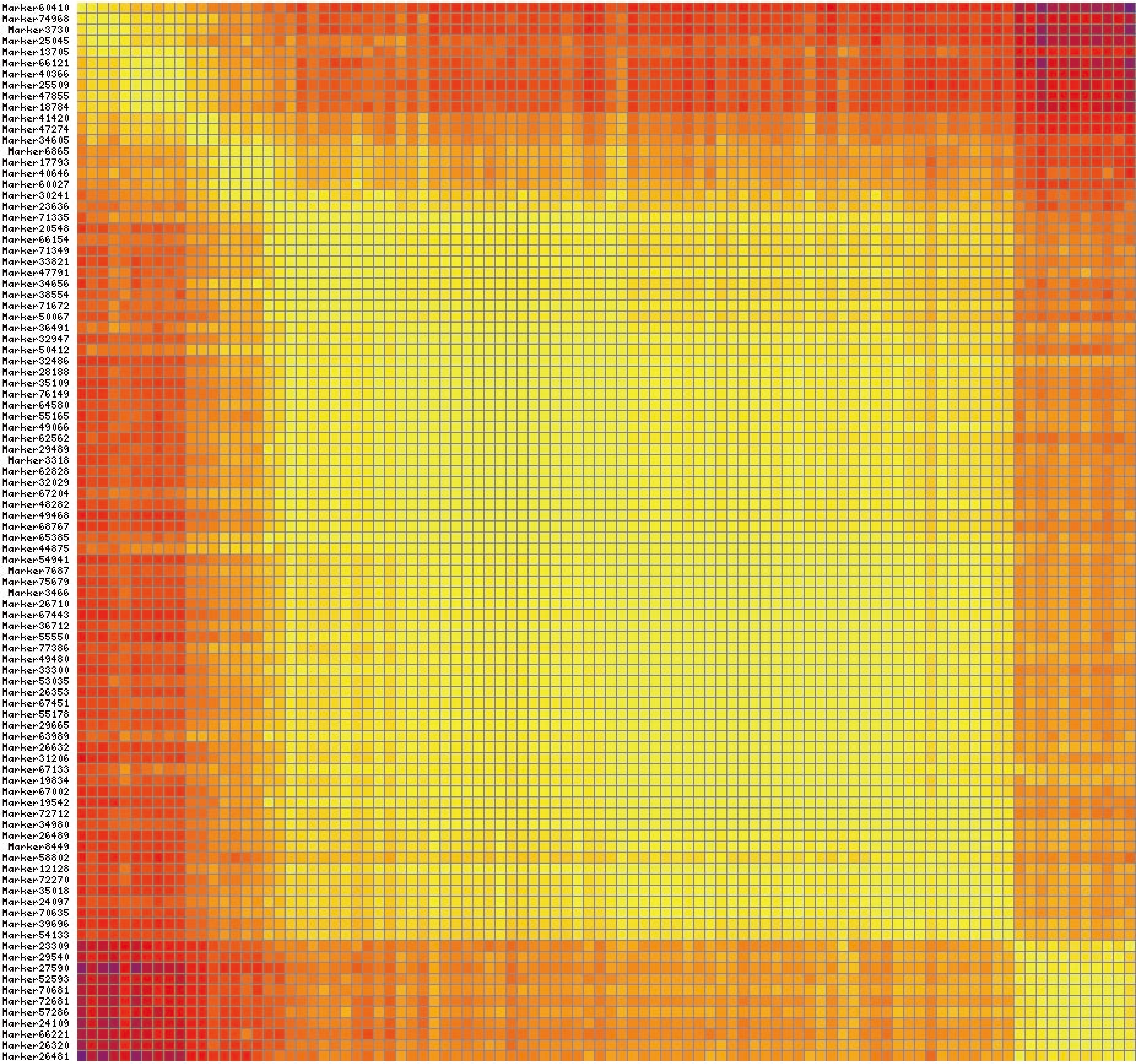
Marker60410
Marker74968
Marker3730
Marker25045
Marker13705
Marker66121
Marker40366
Marker25509
Marker47855
Marker18784
Marker41420
Marker47274
Marker34605
Marker6865
Marker17793
Marker40646
Marker60027
Marker30241
Marker23636
Marker71335
Marker20548
Marker66154
Marker71349
Marker33821
Marker47791
Marker34656
Marker38554
Marker71672
Marker50067
Marker36491
Marker32947
Marker50412
Marker32486
Marker28188
Marker35109
Marker76149
Marker64580
Marker55165
Marker49066
Marker62562
Marker29489
Marker3318
Marker62828
Marker32029
Marker67204
Marker48282
Marker49468
Marker68767
Marker65385
Marker44875
Marker54941
Marker7687
Marker75679
Marker3466
Marker26710
Marker67443
Marker36712
Marker55550
Marker77386
Marker49480
Marker33300
Marker53035
Marker26353
Marker67451
Marker55178
Marker29665
Marker63989
Marker26632
Marker31206
Marker67133
Marker19834
Marker67002
Marker19542
Marker72712
Marker34980
Marker26489
Marker8449
Marker58802
Marker12128
Marker72270
Marker35018
Marker24097
Marker70635
Marker39696
Marker54133
Marker23309
Marker29540
Marker27590
Marker52593
Marker70681
Marker72681
Marker57286
Marker24109
Marker66221
Marker26320
Marker26481

LG27

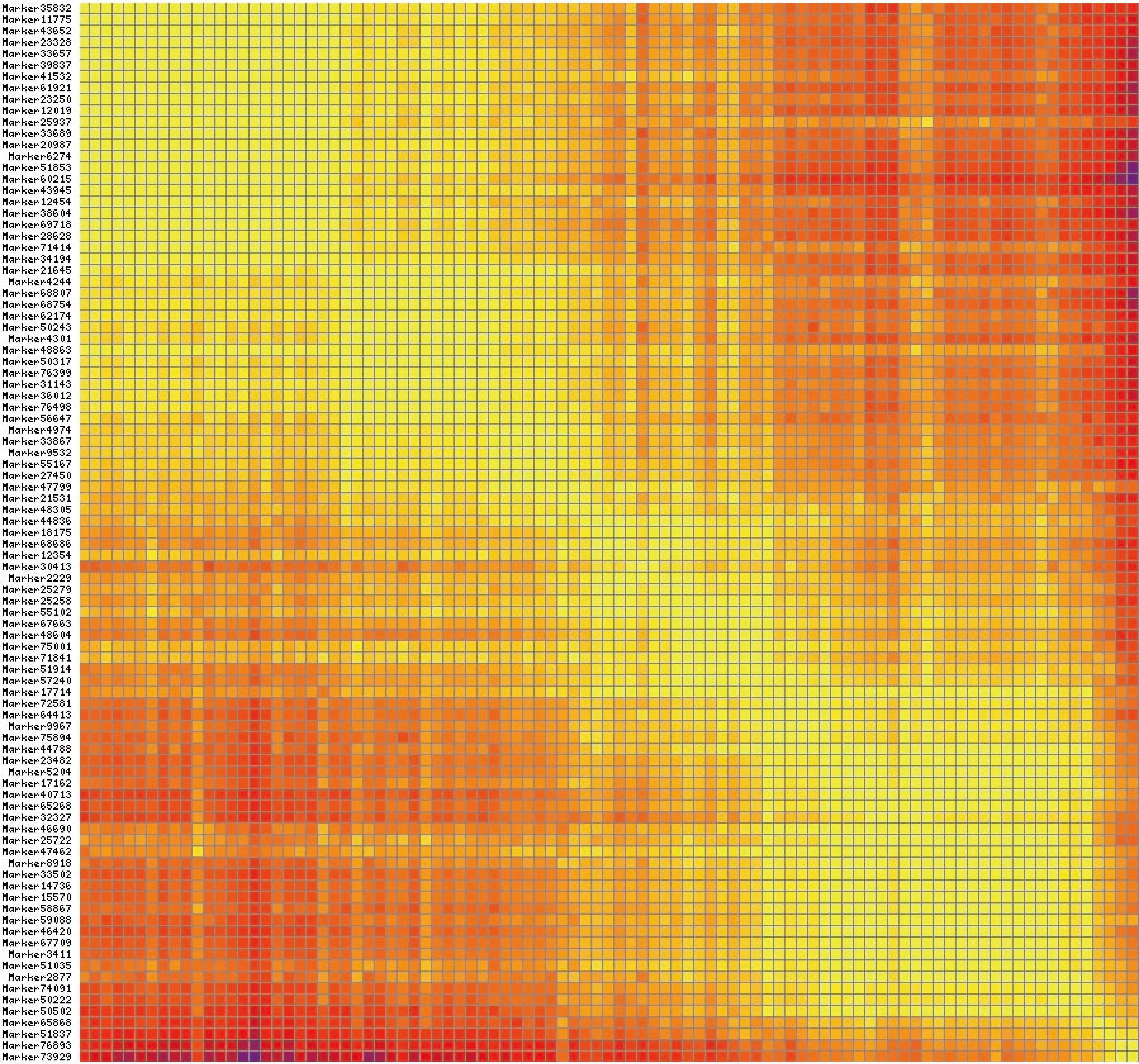
Marker35832
Marker11775
Marker43652
Marker23328
Marker33657
Marker39837
Marker41532
Marker61921
Marker23250
Marker12019
Marker25937
Marker33689
Marker20987
Marker6274
Marker51853
Marker60215
Marker43945
Marker12454
Marker38604
Marker69718
Marker28628
Marker71414
Marker34194
Marker21645
Marker4244
Marker68807
Marker68754
Marker62174
Marker50243
Marker4301
Marker48863
Marker50317
Marker76399
Marker31143
Marker36012
Marker76498
Marker56647
Marker4974
Marker33867
Marker9532
Marker55167
Marker27450
Marker47799
Marker21531
Marker48305
Marker44836
Marker18175
Marker68686
Marker12354
Marker30413
Marker2229
Marker25279
Marker25258
Marker55102
Marker67663
Marker48604
Marker75001
Marker71841
Marker51914
Marker57240
Marker17714
Marker72581
Marker64413
Marker9967
Marker75894
Marker44788
Marker23482
Marker5204
Marker17162
Marker40713
Marker65268
Marker32327
Marker46690
Marker25722
Marker47462
Marker8918
Marker33502
Marker14736
Marker15570
Marker58867
Marker59088
Marker46420
Marker67709
Marker3411
Marker51035
Marker2877
Marker74091
Marker50222
Marker50502
Marker65868
Marker51837
Marker76893
Marker73929

LG28

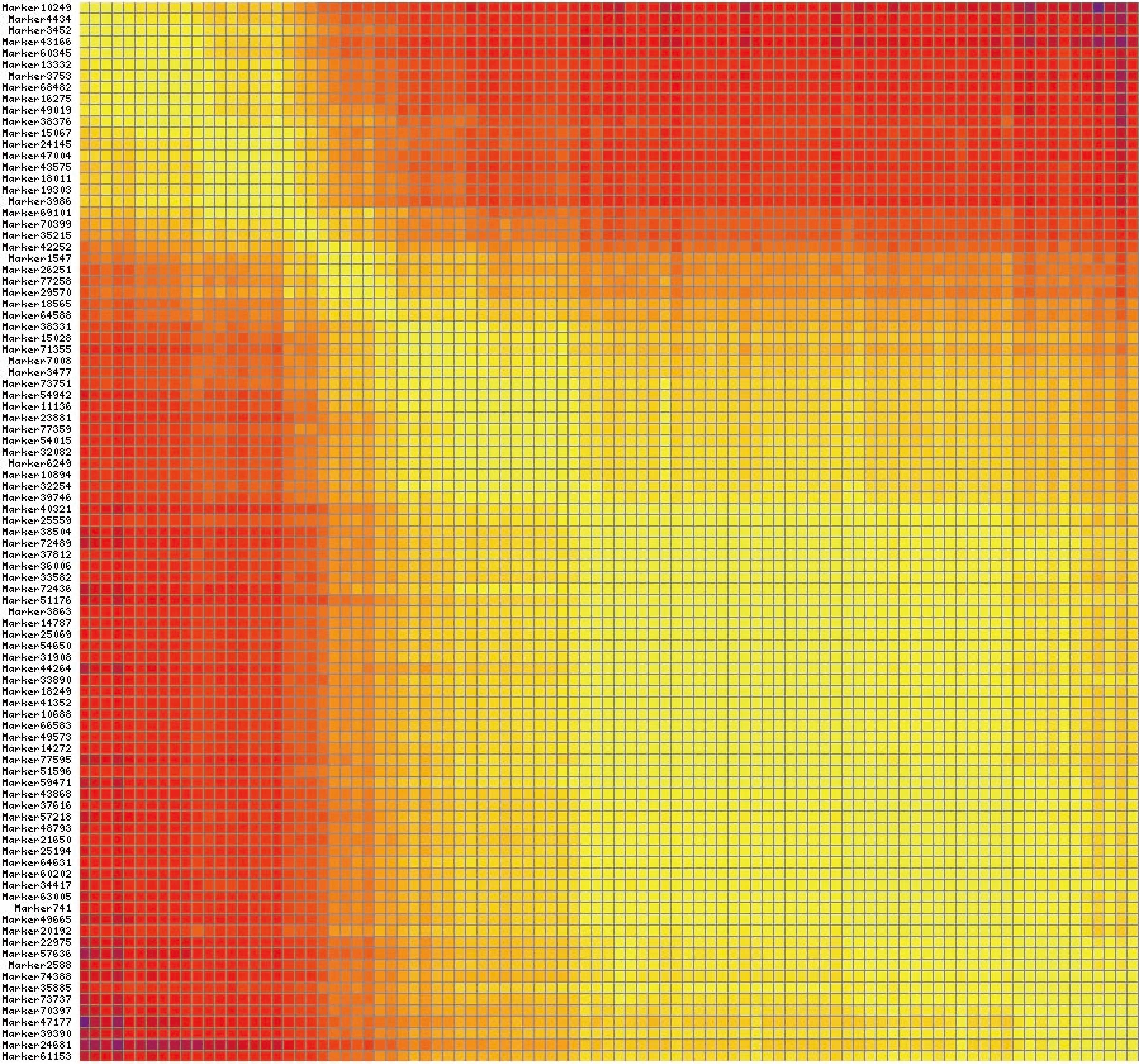

Marker10249
Marker4434
Marker3452
Marker43166
Marker60345
Marker13332
Marker3753
Marker68482
Marker16275
Marker49019
Marker38376
Marker15067
Marker24145
Marker47004
Marker43575
Marker18011
Marker19303
Marker3986
Marker69101
Marker70399
Marker35215
Marker42252
Marker1547
Marker26251
Marker77258
Marker29570
Marker18565
Marker64588
Marker38331
Marker15028
Marker71355
Marker7008
Marker3477
Marker73751
Marker54942
Marker11136
Marker23881
Marker77359
Marker54015
Marker32082
Marker6249
Marker10894
Marker32254
Marker39746
Marker40321
Marker25559
Marker38504
Marker72489
Marker37812
Marker36006
Marker33582
Marker72436
Marker51176
Marker3863
Marker14787
Marker25069
Marker54650
Marker31908
Marker44264
Marker33890
Marker18249
Marker41352
Marker10688
Marker66583
Marker49573
Marker14272
Marker77595
Marker51596
Marker59471
Marker43868
Marker37616
Marker57218
Marker48793
Marker21650
Marker25194
Marker64631
Marker60202
Marker34417
Marker63005
Marker741
Marker49665
Marker20192
Marker22975
Marker57636
Marker2588
Marker74388
Marker35885
Marker73737
Marker70397
Marker47177
Marker39390
Marker24681
Marker61153

LG29

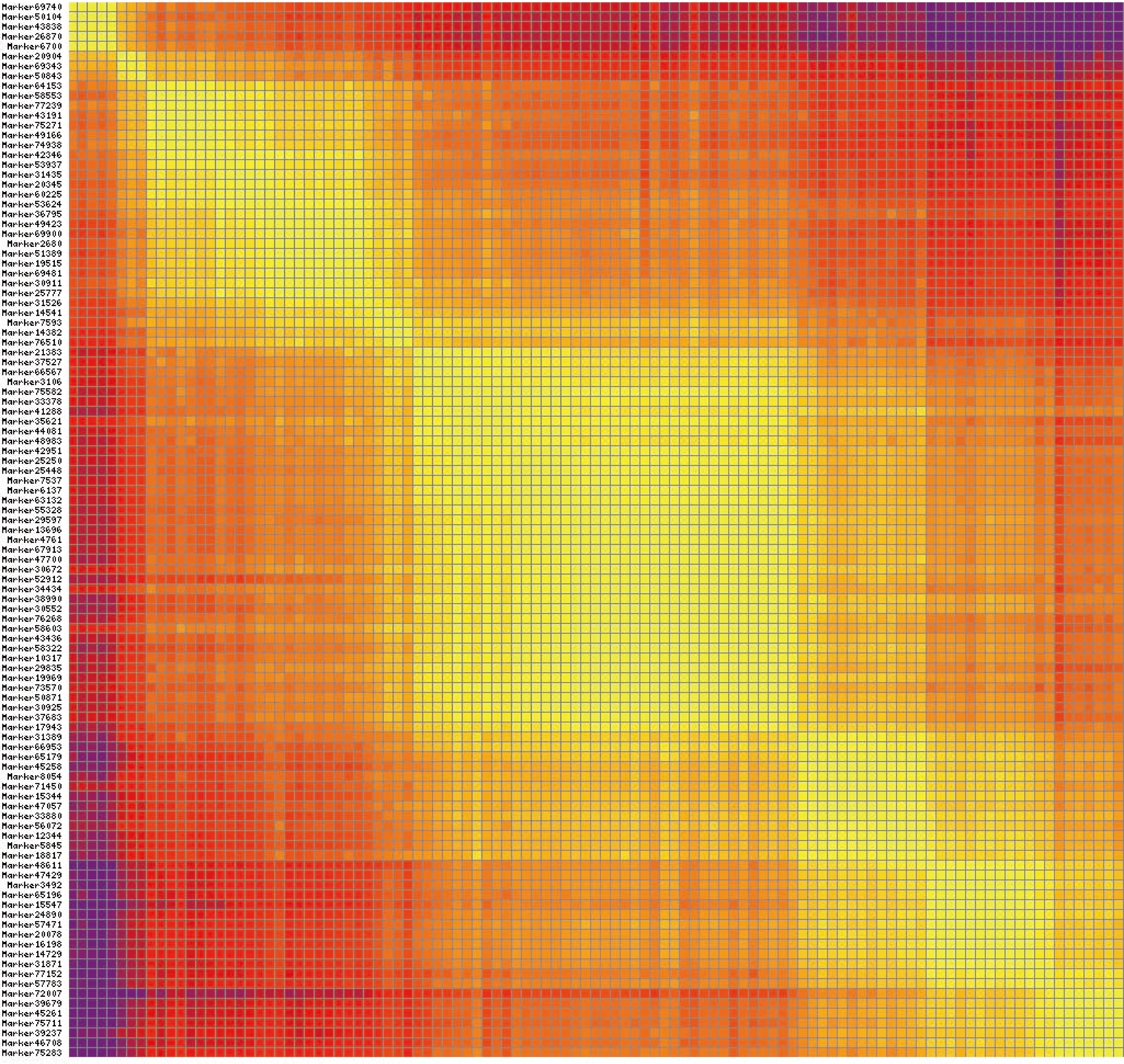
Marker69740
Marker50104
Marker43838
Marker26870
Marker6700
Marker20904
Marker69343
Marker50843
Marker64153
Marker58553
Marker77239
Marker43191
Marker75271
Marker49166
Marker74938
Marker42346
Marker53937
Marker31435
Marker20345
Marker60225
Marker53624
Marker36795
Marker49423
Marker69900
Marker2680
Marker51389
Marker19515
Marker69481
Marker30911
Marker25777
Marker31526
Marker14541
Marker7593
Marker14382
Marker76510
Marker21383
Marker37527
Marker66567
Marker3106
Marker75582
Marker33378
Marker41288
Marker35621
Marker44081
Marker48983
Marker42951
Marker25250
Marker25448
Marker7537
Marker6137
Marker63132
Marker55328
Marker29597
Marker13696
Marker4761
Marker67913
Marker47700
Marker30672
Marker52912
Marker34434
Marker38990
Marker30552
Marker76268
Marker58603
Marker43436
Marker58322
Marker10317
Marker29835
Marker19969
Marker73570
Marker50871
Marker30925
Marker37683
Marker17943
Marker31389
Marker66953
Marker65179
Marker45258
Marker8054
Marker71450
Marker15344
Marker47057
Marker33880
Marker56072
Marker12344
Marker5845
Marker18817
Marker48611
Marker47429
Marker3492
Marker65196
Marker15547
Marker24890
Marker57471
Marker20078
Marker16198
Marker14729
Marker31871
Marker77152
Marker57783
Marker72007
Marker39679
Marker45261
Marker75711
Marker39237
Marker46708
Marker75283

LG30

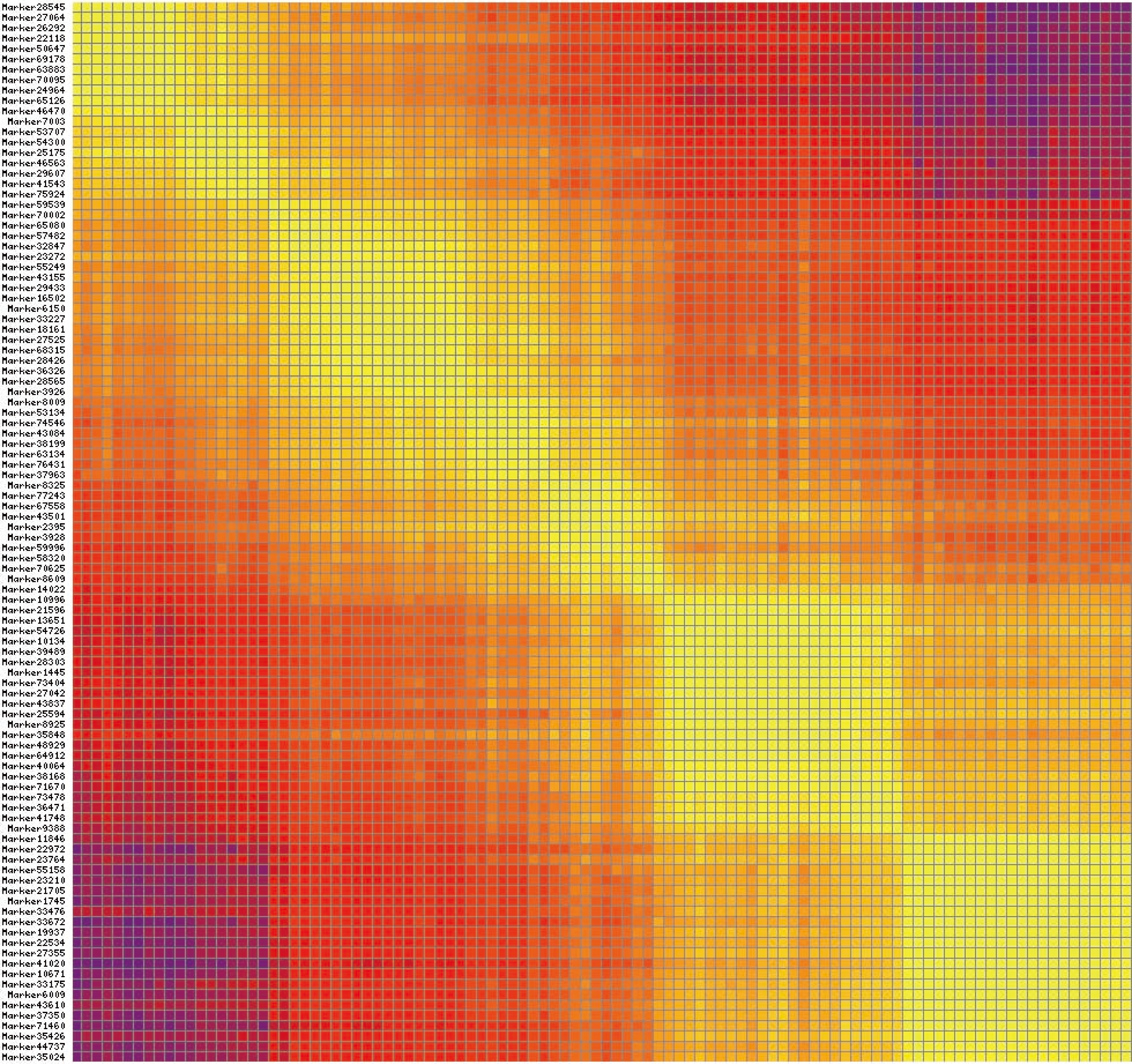
Marker28545
Marker27064
Marker26292
Marker22118
Marker50647
Marker69178
Marker63883
Marker70095
Marker24964
Marker65126
Marker46470
Marker7003
Marker53707
Marker54300
Marker25175
Marker46563
Marker29607
Marker41543
Marker75924
Marker59539
Marker70002
Marker65080
Marker57482
Marker32847
Marker23272
Marker55249
Marker43155
Marker29433
Marker16502
Marker6150
Marker33227
Marker18161
Marker27525
Marker68315
Marker28426
Marker36326
Marker28565
Marker3926
Marker8009
Marker53134
Marker74546
Marker43084
Marker38199
Marker63134
Marker76431
Marker37963
Marker8325
Marker77243
Marker67558
Marker43501
Marker2395
Marker3928
Marker59996
Marker58320
Marker70625
Marker8609
Marker14022
Marker10996
Marker21596
Marker13651
Marker54726
Marker10134
Marker39489
Marker28303
Marker1445
Marker73404
Marker27042
Marker43837
Marker25594
Marker8925
Marker35848
Marker48929
Marker64912
Marker40064
Marker38168
Marker71670
Marker73478
Marker36471
Marker41748
Marker9388
Marker11846
Marker22972
Marker23764
Marker55158
Marker23210
Marker21705
Marker1745
Marker33476
Marker33672
Marker19937
Marker22534
Marker27355
Marker41020
Marker10671
Marker33175
Marker6009
Marker43610
Marker37350
Marker71460
Marker35426
Marker44737
Marker35024
