# Supplementary material for: Construction of a high-density genetic map and mapping of a sex-linked locus for the brown alga Undaria pinnatifida (Phaeophyceae) based on large scale marker development by specific length amplified fragment (SLAF) sequencing
Source: BMC Genomics. 2015 Nov 5;16:902. doi: 10.1186/s12864-015-2184-y (PMC4635539; doi:10.1186/s12864-015-2184-y)
Supplement: Additional file 6: — Result of the blast analysis of the tightly sex-linked markers against the unigene database of Undaria pinntifida . (DOCX 18 kb) [file 12864_2015_2184_MOESM6_ESM.docx]

BLASTN 2.2.26 [Sep-21-2011]

Reference: Altschul, Stephen F., Thomas L. Madden, Alejandro A. Schaffer,

Jinghui Zhang, Zheng Zhang, Webb Miller, and David J. Lipman (1997),

"Gapped BLAST and PSI-BLAST: a new generation of protein database search

programs", Nucleic Acids Res. 25:3389-3402.

Query= Marker43089

(210 letters)

Database: Wakame.Unigene.fa

44,264 sequences; 58,493,649 total letters

Searching..................................................done

Score E

Sequences producing significant alignments: (bits) Value

CL134Contig1 115 2e-25

CL1827Contig1 115 2e-25

>CL134Contig1

Length = 10725

Score = 115 bits (58), Expect = 2e-25

Identities = 70/74 (94%)

Strand = Plus / Plus

Query: 112 taagattcaactgtgtatcttattcgtgcgaatagactgcgcattatattagatttgaat 171

||||||||||||| ||||||||||||||||||||||||||||||| ||||||||||||

Sbjct: 4993 taagattcaactgcatatcttattcgtgcgaatagactgcgcattaacttagatttgaat 5052

Query: 172 ccaaggttcatact 185

||||||||||||||

Sbjct: 5053 ccaaggttcatact 5066

>CL1827Contig1

Length = 3276

Score = 115 bits (58), Expect = 2e-25

Identities = 70/74 (94%)

Strand = Plus / Plus

Query: 112 taagattcaactgtgtatcttattcgtgcgaatagactgcgcattatattagatttgaat 171

||||||||||||| ||||||||||||||||||||||||||||||| ||||||||||||

Sbjct: 480 taagattcaactgcatatcttattcgtgcgaatagactgcgcattaacttagatttgaat 539

Query: 172 ccaaggttcatact 185

||||||||||||||

Sbjct: 540 ccaaggttcatact 553

BLASTN 2.2.26 [Sep-21-2011]

Reference: Altschul, Stephen F., Thomas L. Madden, Alejandro A. Schaffer,

Jinghui Zhang, Zheng Zhang, Webb Miller, and David J. Lipman (1997),

"Gapped BLAST and PSI-BLAST: a new generation of protein database search

programs", Nucleic Acids Res. 25:3389-3402.

Query= Marker19020

(210 letters)

Database: Wakame.Unigene.fa

44,264 sequences; 58,493,649 total letters

Searching..................................................done

***** No hits found ******

BLASTN 2.2.26 [Sep-21-2011]

Reference: Altschul, Stephen F., Thomas L. Madden, Alejandro A. Schaffer,

Jinghui Zhang, Zheng Zhang, Webb Miller, and David J. Lipman (1997),

"Gapped BLAST and PSI-BLAST: a new generation of protein database search

programs", Nucleic Acids Res. 25:3389-3402.

Query= Marker6556

(210 letters)

Database: Wakame.Unigene.fa

44,264 sequences; 58,493,649 total letters

Searching..................................................done

***** No hits found ******

BLASTN 2.2.26 [Sep-21-2011]

Reference: Altschul, Stephen F., Thomas L. Madden, Alejandro A. Schaffer,

Jinghui Zhang, Zheng Zhang, Webb Miller, and David J. Lipman (1997),

"Gapped BLAST and PSI-BLAST: a new generation of protein database search

programs", Nucleic Acids Res. 25:3389-3402.

Query= Marker60771

(210 letters)

Database: Wakame.Unigene.fa

44,264 sequences; 58,493,649 total letters

Searching..................................................done

Score E

Sequences producing significant alignments: (bits) Value

CL11961Contig1 139 1e-32

>CL11961Contig1

Length = 773

Score = 139 bits (70), Expect = 1e-32

Identities = 91/98 (92%)

Strand = Plus / Plus

Query: 2 cctaggaaatcggatggtgctcctgacgtgaaatcatcggggaagaaaccatctgcatcg 61

|||||||| ||||||||||||||||||||||||||||||||||| | ||||| |||||

Sbjct: 396 cctaggaagtcggatggtgctcctgacgtgaaatcatcggggaacgagtcatctacatcg 455

Query: 62 gctggggtgtccttagcatgtggcagtcgtgacagggc 99

||||||||||||||||||||||||||||||| ||||||

Sbjct: 456 gctggggtgtccttagcatgtggcagtcgtggcagggc 493

Score = 113 bits (57), Expect = 8e-25

Identities = 78/85 (91%)

Strand = Plus / Minus

Query: 125 acccacagaacgggaatatatgggagtgctgtgggcagtagatcactttcgaccctattt 184

|||||| ||||| |||| |||||||||||||| ||||||||||||||||||||||| |||

Sbjct: 713 acccaccgaacgagaatgtatgggagtgctgtaggcagtagatcactttcgacccttttt 654

Query: 185 agctggtatgcagttcaaggtagtg 209

|||||||| |||||| |||||||||

Sbjct: 653 agctggtaggcagtttaaggtagtg 629

BLASTN 2.2.26 [Sep-21-2011]

Reference: Altschul, Stephen F., Thomas L. Madden, Alejandro A. Schaffer,

Jinghui Zhang, Zheng Zhang, Webb Miller, and David J. Lipman (1997),

"Gapped BLAST and PSI-BLAST: a new generation of protein database search

programs", Nucleic Acids Res. 25:3389-3402.

Query= Marker26359

(210 letters)

Database: Wakame.Unigene.fa

44,264 sequences; 58,493,649 total letters

Searching..................................................done

***** No hits found ******

Database: Wakame.Unigene.fa

Posted date: Sep 8, 2015 6:50 PM

Number of letters in database: 58,493,649

Number of sequences in database: 44,264

Lambda K H

1.37 0.711 1.31

Gapped

Lambda K H

1.37 0.711 1.31

Matrix: blastn matrix:1 -3

Gap Penalties: Existence: 5, Extension: 2

Number of Sequences: 44264

Number of Hits to DB: 399,712

Number of extensions: 4967

Number of successful extensions: 4967

Number of sequences better than 1.0e-20: 3

Number of HSP's gapped: 4967

Number of HSP's successfully gapped: 4

Length of database: 58,493,649

X1: 11 (21.8 bits)

X2: 15 (29.7 bits)

X3: 50 (99.1 bits)

S1: 12 (24.3 bits)

S2: 51 (101.6 bits)
